# Characterization of the *E. coli* proteome and its modifications during growth and ethanol stress

**DOI:** 10.3389/fmicb.2015.00103

**Published:** 2015-02-18

**Authors:** Boumediene Soufi, Karsten Krug, Andreas Harst, Boris Macek

**Affiliations:** Proteome Center Tuebingen, University of TuebingenTuebingen, Germany

**Keywords:** Super-SILAC, quantitative proteomics, absolute quantitation, stress response, *E. coli*, post translational modifications

## Abstract

We set out to provide a resource to the microbiology community especially with respect to systems biology based endeavors. To this end, we generated a comprehensive dataset monitoring the changes in protein expression, copy number, and post translational modifications in a systematic fashion during growth and ethanol stress in *E. coli*. We utilized high-resolution mass spectrometry (MS) combined with the Super-SILAC approach. In a single experiment, we have identified over 2300 proteins, which represent approximately 88% of the estimated expressed proteome of *E. coli* and estimated protein copy numbers using the Intensity Based Absolute Quantitation (iBAQ). The dynamic range of protein expression spanned up to six orders of magnitude, with the highest protein copy per cell estimated at approximately 300,000. We focused on the proteome dynamics involved during stationary phase growth. A global up-regulation of proteins related to stress response was detected in later stages of growth. We observed the down-regulation of the methyl directed mismatch repair system containing MutS and MutL of *E. coli* growing in long term growth cultures, confirming that higher incidence of mutations presents an important mechanism in the increase in genetic diversity and stationary phase survival in *E. coli*. During ethanol stress, known markers such as alcohol dehydrogenase and aldehyde dehydrogenase were induced, further validating the dataset. Finally, we performed unbiased protein modification detection and revealed changes of many known and unknown protein modifications in both experimental conditions. Data are available via ProteomeXchange with identifier PXD001648.

## Introduction

*E. coli* serves as an excellent model to study general features of prokaryotic proteome, such as its dynamics under various physiological conditions, its dynamic range of expression and its modifications. Despite the significant progress made toward the understanding of bacterial regulatory processes, the scale and dynamics of the global protein expression during bacterial growth and stress response has not been addressed systematically. During growth in batch culture, bacteria have to constantly monitor changes and make adjustments on the molecular level during different stages of bacterial growth (Nystrom, 2004). For example, long term growing *E. coli* in stationary phase have developed specific mechanisms encouraging certain genetic mutations to occur in order to cope with the numerous stresses encountered during stationary phase, better known as the growth advantage in stationary phase (GASP) phenotype (Finkel, 2006). *E. coli* is an excellent organism to use when studying ethanol stress response, as it is a commonly used industrial strain in many processes including bio-ethanol production (Woodruff et al., 2013). Ethanol stress in *E. coli* is known to cause a variety of different physiological responses such as the inhibition of peptidoglycan biosynthesis (Buttke and Ingram, 1978), and fatty acid biosynthesis (Clark and Beard, 1979). Despite the fact that previous studies monitored bacterial proteome changes in response to various stresses and growth conditions (Bernhardt et al., 2003; Lee et al., 2006; Soufi et al., 2010; Soares et al., 2013), a systematic and comprehensive analysis of proteome changes during growth, and ethanol stress in *E. coli* has not been performed. Monitoring these changes and processes on the level of the proteome, will allow for a much better understanding into the adaptive mechanisms bacteria undertake during changes in their environment.

Quantitative mass spectrometry (MS) based proteomics has become an invaluable tool utilized to study protein expression and dynamics in a global fashion (Aebersold and Mann, 2003). Technologies developed in this field have evolved quite dramatically within the last decade, especially in context of advanced methodologies in the metabolic labeling of proteins using stable isotopes such as ^15^N labeling (Gouw et al., 2011) and Stable isotope labeling of amino acids in cell culture (SILAC) (Ong et al., 2002). SILAC has been used before in studies of bacterial growth; however, its classical application allows for comparison of only three conditions at a time (Soares et al., 2013). One extension of SILAC, known as the Super-SILAC approach, involves mixing samples from different experimental conditions labeled with the same SILAC-label to obtain an internal standard. This labeled standard can then be added into several samples and used for their indirect quantitative comparison. This approach can be used to produce a quantitative analysis of a wide range of biological and environmental samples (Geiger et al., 2010) and has been applied primarily in eukaryotic systems for quantification of many different types of cancer and tumor cell lines (Deeb et al., 2012; Geiger et al., 2012; Lund et al., 2012; Boersema et al., 2013; Schweppe et al., 2013); only one application of this approach has been reported in prokaryotic systems so far (Berghoff et al., 2013).

In this study, we employ the Super-SILAC approach to study proteome dynamics in bacteria during growth and ethanol stress. We investigate the absolute and relative proteome dynamics on the global scale at seven distinct growth phases in *E. coli* cultured in minimal medium. We identify 2303 proteins with 1604 proteins being absolutely quantified in all seven growth phases, and achieve good reproducibility between biological replicates. We extend this approach to monitor ethanol stress response in *E. coli* at two distinct time points leading to the identification of 2251 and quantification of 1804 proteins. Distinct global proteome changes were observed in both analyzed conditions with good correlation between biological replicates in all experiments. In terms of an unbiased detection of protein post translational modification changes, we detected numerous types of modifications depending on the growth state employed.

## Materials and methods

### Bacterial strain

The *E. coli BW25113* strain was employed in all experiments conducted in this study.

### Bacterial cell culture and SILAC labeling

Each experiment was performed in two biological replicates. *E. coli* cells were grown in a 2L flask containing 500 ml of minimal M9 media with stable isotope-labeled (heavy) L-Lysine-13C6, 15N2 in batch culture. Aliquots of batch culture were removed at each specific growth stage (7 time points) in the growth experiment or collected at pre stress, 10 min, and 2 h post ethanol stress in the ethanol stress experiment (Supplementary Figure 1). Separately, 1.25L of cells were grown in a 5L flask with M9 medium containing unlabeled (light) L-Lysine-12C6, 14N2 at the exact seven different phases of growth or collected at 10 min and 2 h post ethanol stress. For ethanol stress experiments, 4% v/v of ethanol was added when cells had reached an OD600 of 0.4.

### Protein extraction and mixing

The cells were harvested by centrifugation at 4000 g for 10 min, media was removed and cells were snap-frozen in liquid nitrogen and stored at −80°C. The cell pellets were resuspended in the appropriate amount of the commercially available YPER lysis buffer (Thermo Scientific), plus 50 μg/ml lysozyme. Cell wall lysis was performed at 37°C for 20 min, followed by brief sonication on ice (1 min at 40% amplitude) to remove DNA. Cellular debris was removed by centrifugation at 13000 g for 30 min. The crude protein extract was precipitated via the methanol/chloroform approach and the proteins were resuspended in denaturation buffer containing 6 M urea/2 M thiourea in 10 mM Tris. Protein concentration was measured by Bradford assay (Bio-Rad, Munich, Germany). Protein extracts obtained from L-Lysine-13C6, 15N2 labeled cells were mixed 1:1:1:1:1:1:1 for growth, and 1:1:1 for ethanol stress to obtain the Super-SILAC standard (SSS) for each respective experiment. Proteins labeled with L-Lysine-12C6, 14N2 (50 μg) were then mixed in an equimolar ratio with the SSS (50 μg), resulting in 7 protein samples per biological replicate.

### In-solution digestion

A total of 100 μg (50 μg SSS and 50 μg light) of crude protein extract in denaturation buffer were digested via in-solution prior to peptide separation. Briefly, proteins were reduced with 1 mM dithiothreitol for 1 h shaking at room temperature, alkylated with 5.5 mM iodoacetamide for 1 h shaking at room temperature in the dark, predigested with 1:100 (w/w) endoproteinase Lys-C for 3 h at room temperature, diluted with 4 volumes of 20 mM ammonium bicarbonate followed by an overnight digestion step 1:100 (w/w) endoproteinase Lys-C at room temperature. The protein digest was acidified using trifluoroacetic acid (TFA) to a final concentration of 0.1% (v/v) to stop the protein digestion. In the case of samples fractionated using Off-Gel fractionation no TFA was added. For the intensity-based absolute quantification of proteins (iBAQ), the UPS2 Proteomics Dynamic Range Standard set (Sigma-Aldrich) was spiked in prior to digestion.

### Isoelectric focusing

Hundred microgram of peptides were separated using the OFFGEL 3100 Fractionator (Agilent) following the experimental steps as previously reported (Hubner et al., 2008). Separation was performed on a 13 cm Immobiline DryStrip (GE Healthcare) with a PH separation gradient of 3–13 to a maximum of 50 μA for 20 kVh. Peptides were acidified using an acidic solution (30% ACN, 5% Acetic acid, 10%TFA), followed by a cleanup step using Stage-Tips loaded with C18 material (Rappsilber et al., 2007).

### SDS-PAGE and In-Gel digestion

Protein separation via SDS-PAGE followed by an In-Gel digestion was used for one biological replicate from each Super-SILAC experiment. Briefly, 100 μg (50 μg SSS and 50 μg light) of crude protein extracts were separated on a NuPage Bis-Tris 4–12% gradient gel (Invitrogen). After staining the gel with Coomassie Blue, protein lanes with cut into 12 equal slices. Coomassie Blue stain was subsequently removed from each gel slice via washing with 10 mM ammonium bicarbonate (ABC) and acetonitrile (ACN) (1:1, v/v). Protein slices were reduced with 10 mM dithiothreitol (DTT) in 20 mM (ABC) for 45 min at 56°C and alkylated with 55 mM iodoacetamide IAA in 20 mM ABC for 30 min in the dark at room temperature. Gel slices were washed twice with 5 mM ABC, followed by a dehydration step by incubating with 100% ACN at room temperature. Proteins were digested with Lys-C (Wako) (12.5 ng/μL in 20 mM ABC) at 37°C overnight. Digested peptides were recovered from each gel slice using three consecutive extraction steps: (I) 3% TFA in 30% ACN (II) 0.5% acetic acid in 80% ACN (III) 100% ACN. Resulting peptides were desalted using StageTips (Ishihama et al., 2006).

### LC-MS/MS analysis

All samples were measured on an EASY-nLC II nano-LC (Proxeon Biosystems) coupled to an Orbitrap Elite mass spectrometer (Thermo Fisher Scientific). Peptides were further separated chromatographically using a 15 cm PicoTip fused silica emitter with an inner diameter of 75 μm (New Objective) packed in-house with reversed-phase ReproSil-Pur C18-AQ 3 μm resin (Dr. Maisch GmbH). A total of 2 μg of peptides were injected into the column, using the intelliflow technology with solvent A (0.5% acetic acid) at a rate of 200 nL/min to a maximum pressure of 280 Bar. Peptides were then eluted using a 90 min segmented gradient of 5–50% solvent B (80% ACN in 0.5% acetic acid). The mass spectrometer was operated on a data-dependent positive ion mode. Peptide fragmentation (MS/MS) was induced using either collision induced dissociation (CID) or higher-energy collisional dissociation (HCD). Survey spectral full-scans were recorded between 300 and 2000 Thomson at a resolution of 120,000 with a target value of 1E6 charges in the LTQ mass analyzer. The 20 most intense peaks from the survey scans were selected for fragmentation using CID with a normalized collision energy of 35% at a target value of 5000 charges. The dynamic exclusion window was set at 90 s. For operation via HCD, normalized collision energy of 40 eV requiring a minimum signal of 1000 and a target value of 4E4 charges was employed. The spectra were acquired in the Orbitrap mass analyzer with a resolution of 7500. The 15 most intense peaks from the survey scans were selected for fragmentation using HCD.

### Data processing

All acquired MS data was processed with the MaxQuant software suite (Cox and Mann, 2008) version 1.2.2.9. Briefly, the SILAC labeling parameter was set to a multiplicity of Two (Lys0, Lys8). After all peptides were quantified, a database search was performed using the MaxQuant internal search engine Andromeda (Cox et al., 2011). Full enzyme specificity was required, with an allowance of up to two missed cleavages. Lys-C was specified as the protease. MS/MS spectra were searched against the Uniprot *Escherichia coli* K12 database (taxonomy reference: 833333), complete proteome set containing 4303 protein entries, downloaded Dec 24, 2012. For absolute protein quantification analysis, MS/MS spectra were also searched against another FASTA file containing the Proteomics Dynamic Range Standard (UPS2, Sigma) with a total of 48 entries. Methionine oxidation and protein N-terminal acetylation were defined as variable modifications. Cysteine carbamidomethylation was defined as a fixed modification. MS scan mass tolerance was set to 6 ppm. For CID fragmentation, the MS/MS tolerance was 0.5 Da, whereas for HCD fragmentation the mass tolerance was set to 20 ppm. Peptides and assembled proteins were searched at a false discovery rate (FDR) of 1%. A minimum of two quantified peptides per protein were required for protein quantitation.

### Relative protein quantitation

SILAC ratios of proteins (“light” to “heavy”) were transformed to log2 scale and only proteins that were quantified in all seven growth phases (growth) or all three time points (ethanol stress) were considered for further quantitative analysis. The magnitude of fluctuation was expressed by calculating the standard deviation of the log-transformed ratios across the growth phases or time points. The resulting quartiles of the distribution were used to bin proteins according to the extent of fluctuation. Those belonging to the quartile with the highest standard deviation (75–100%) were defined as “fluctuating” or dynamic, whereas those belonging to the quartile with the lowest standard deviation (0–25%) were defined as “non-fluctuating” or static.

### Bacterial cell counting

As part of the protein copy number /cell calculation, the total number of *E. coli* cells were counted. Briefly, *E. coli* cells were grown in minimal M9 media supplemented with 0.5% glucose at an OD600 = 0.5 (TP3) and 1.0 (TP5) were grown in minimal media. Experiments were performed in biological triplicates and replicates at an OD600 of 0.5 and 1.0, respectively. 500 μl of cells were mixed with 50 μl 25% para-formaldehyde (20 mM MOPS PS = 7) and 50 μl 2.5% glutaraldehyde, followed by an incubation period of 15 min at room temperature. Cells were counted using Fluorescence-activated Cell Sorting (FACS). In brief, the BD LSR Fortesa FACS instrument was used. Standard settings were employed (log scale acquisition, Threshold: 400 FCS, Acquisition speed: low). Gating settings were set according to cell size in order to remove background noise in the form of cellular aggregates and debris. *E. coli* cells were diluted 1:20 for more accurate counts (5 μl cells, 95 μl water). Each biological replicate was measured 3 times (technical replicates) and averaged in order to obtain the final cell count at each growth phase. The exact number of cells was derived from the total volume of cells taken for protein extraction, and calculated for the amount of input protein material that was used for digestion (50 μg).

### Absolute protein quantitation

The absolute protein copy number per cell was calculated for the Super-SILAC growth experiment in two stages of growth: logarithmic and early stationary. To this end, the intensity based absolute quantitation approach (iBAQ) supported by the MaxQuant Software suite was employed as previously shown. The absolute amount of protein is proportional to the absolute molar amounts of the UPS2 protein standard spiked into the protein sample. All acquired iBAQ values were divided by 4, due to the fact that ¼ of the UPS2 protein was spiked in the log and early stationary Super-SILAC growth experimental samples. The total number of cells counted via FACS (N_cells_), was used to calculate the absolute protein copy numbers (CN_protein_) by utilizing the calculation as previously described (Carpy et al., 2014):

$$CN_{protein}=\frac{N_{A}\left(iBAQ_{protein}\;*\;10^{-15}\right)}{N_{cells}}$$

N_A_ represents Avogadro's number. The absolute protein amounts calculated for the super-SILAC standard together with corresponding SILAC ratios were used to calculate the absolute amount for each protein in all seven stages of growth in the growth analysis experiment.

### Peak time index calculation

For each protein we determined the experimental condition of its highest expression (“peak time index”) as described in Olsen et al. (2010). Briefly, the SILAC ratios for each protein were normalized to the maximal change across the experiment. We then calculated the weighted mean of the expression ratio in a particular condition with respect to the normalized ratios of adjacent conditions, i.e., adjacent growth phases or time points upon ethanol stress. We slightly modified the peak time index calculation as described in Olsen et al. (2010) to account for the acyclicity of our experiments. To assign the resulting “peak time index” of every protein to a specific growth phase respective time point, we applied hierarchical clustering on these values using the Euclidian distance and a defined cluster numbers of seven (growth phase) or three (ethanol stress).

### Functional enrichment analysis

We retrieved Gene Ontology (GO) annotation of *E. coli* from the UniProt-GOA database (downloaded on April 18, 2012). To test whether specific annotation terms are enriched or depleted within a set of proteins of interest we applied Fisher's exact test using the theoretical *E. coli* proteome as background. Derived *p*-values were further adjusted to address multiple hypothesis testing using the method proposed by Benjamini and Hochberg (1995).

### Unbiased detection of protein modifications

All acquired MS data was processed with the MaxQuant software suite (Cox and Mann, 2008) version 1.2.2.9. Briefly, the SILAC labeling parameter was set to a multiplicity of Two (Lys0, Lys8), or as unlabeled (in order to serve as quality control for dependent peptide (DP) function). All other parameters were the same as described above, except that the DP function was enabled in order to search for modified peptides that were not identified. Briefly, MaxQuant compares all identified MS/MS to all unidentified MS/MS spectra. The precursor mass differences are calculated between identified spectra (“base peptides”) and unidentified spectra with similar MS/MS features (“dependent peptides”) and corresponding possible modifications are reported.

## Results and discussion

### Absolute quantification of *E. coli* proteome

In total, we identified 2303 proteins at a false discovery rate (FDR) of 1% of which 1604 were quantified in all seven stages of growth (Table 1, Table S1). We applied the iBAQ MS based strategy (Schwanhausser et al., 2011) to estimate copy numbers of proteins in the analyzed conditions. To this end, we used an internal standard containing absolute molar amount of proteins (standard) spiked into the protein samples. The iBAQ standard was spiked in the *E. coli* Super-SILAC experiment at two time points (TP3 & TP5). This led to the estimates of protein copy numbers for 1587 proteins during growth in minimal medium (Table S2). Moreover, we applied the protein copy numbers from the growth dataset (T3 time point) to the proteins quantified in order to assess protein abundance changes during ethanol stress, which lead to protein copy number estimates for 1620 proteins (Table S7).

**Table 1:** **Total number of identified and quantified proteins**.

	**Growth**	**Ethanol Stress**
Identified	2303	2260
Quantified (all phases)	1604	1984
Absolutely quantified	1587	1620

Despite the fact that MS based strategies have been increasingly used in determining protein copy numbers in living organisms, there is a large discrepancy between previous studies that have estimated protein copy numbers from bacteria to eukaryotes. Possible reasons have been recently discussed in detail (Milo, 2013) and include loss of material during sample preparation (cell lysis, protein digestion etc), the inherent detection limits of mass spectrometers toward low abundant proteins, and errors in MS quantitation methodologies. Furthermore, in bacteria, the total amount of protein moieties per cell and the number of cells can differ greatly depending on the strain and growth conditions employed. Therefore, cell counting was recently identified as one of the main reasons for inaccuracies in estimation of protein copy numbers. To address this, we carefully determined the number of cells that existed in the exact experimental conditions that were employed. To this end, we performed multiple biological and technical measurements of the total *E. coli* cell counts using FACS which showed excellent reproducibility (Table S4, Supplementary Figure 12). We also employed orthogonal methods of determining the number of *E. coli* cells, such as CFU counting and microscopy-based methods, however with much lower level of reproducibility and accuracy then FACS (data not shown). The FACS-derived total cell count was then used to derive the absolute protein copies per cell.

**Figure 1:**
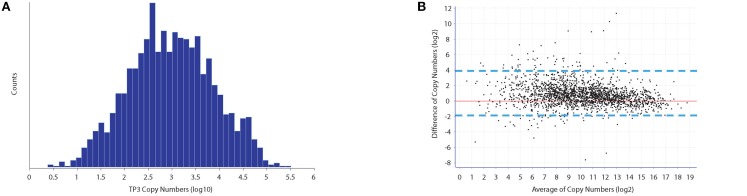
**Protein copy number estimates of *E. coli*. (A)** Dynamic range of protein copy number estimates which span across less than six orders of magnitude from approximately 1–300,000 protein copies per cell. **(B)** Biological reproducibility of Copy number estimates between time points T3 and T5 as indicated through Bland-Altman statistical analysis, indicate a good level of correlation. Outliers between copy number estimates are usually associated with those proteins that are of a general low abundance.

Bland-Altman analysis of replicate UPS standard measurements (difference of the copy numbers between TP3 and TP5 vs the average), revealed a small standard deviation (Figure 1B). Therefore, the average of protein copy numbers between TP3 and TP5 was used to obtain copy numbers for all time points in both growth and ethanol stress datasets. We observed a good level of reproducibility between the protein intensities derived from both the UPS2 internal standard as well as their iBAQ intensities for all proteins between TP3 and TP5 (Supplementary Figure 2). We deduced no significant difference between the total protein moieties/cell in different growth stages (Table S5), which demonstrates an overall balance of protein synthesis and degradation at different points during growth to maintain an overall level of cellular homeostasis. Our results showed that an *E. coli* cell under tested conditions has an average of 10,761,042 protein molecules with a dynamic range of protein copy number within 6 orders of magnitude (Figure 1A). Many of the higher abundant proteins were ribosomal, membrane and carbohydrate metabolism related proteins. For example, previous studies have shown that the most abundant lipomembrane protein in *E. coli* is the Braun lipoprotein found in the outer membrane with approximately 200,000 copies per cell (Braun, 1975). Our studies revealed similar protein copy number estimates of the Braun lipoprotein at 167,568/118,072 copies per cell at TP3 and TP5 respectively which is within the same range of magnitude as previously reported. Elongation factor Tu1 was found to be the most abundant protein with 327,934 and 301,731copies per cell at TP3 and TP5, respectively. The least abundant classes of proteins (<500 copies/cell) for both TP3 and TP5 were enriched toward membrane and membrane associated proteins (inner,outer, plasma etc). This is due to the fact that membrane proteins typically pose problems during a typical cell lysis procedure without specific membrane protein enrichment, thus difficult to accurately detect.

The economics of protein synthesis becomes especially important during extended periods of growth as the bacterial cells needs to prioritize and conserve their energy toward those biological processes that are essential in order to sustain the growth and survival when facing conditions of nutrient starvation and harsh stress conditions. For example, we observed that most Glycolytic/Gluconeogenic enzymes (essential proteins required in environmental growth conditions employed) stay the same or increase in abundance whereas non-essential proteins such as cold shock proteins (respond to specific stress conditions) decrease during the later stages of growth as their synthesis would be a waste or precious energy in which the bacterial cell cannot afford (Supplementary Figures 3A, B). Furthermore, we estimated protein copy numbers for all 299 gene products of *E. coli* that are considered essential which showed a high level of correlation between TP3 and TP5. This is as expected, as essential proteins need to be constantly present in order to maintain cellular viability.

In order to validate the absolute quantification data, we investigated known protein complexes in *E. coli.* Protein complexes serve as a very important role as they act together in order to implement numerous biological functions ultimately regulating complex metabolic pathways. Recently, a study analyzed the synthetic rates of a total of 64 well characterized (in terms of stoichiometry) *E. coli* multiprotein complexes (Li et al., 2014). We took the protein stoichiometries reported from this study to compare with our copy number estimates. We would expect that those protein complexes with similar stoichiometries should have similar copy numbers. We observed that absolute levels of proteins with predicted high copy numbers/cell (>10,000) are more accurate (with respect to their stoichiometry) than those with low copy numbers (<500). This is to be expected as the errors tend to become larger with low copy number proteins due to their lower intensities during MS analysis. All stoichiometries and their copy numbers of the 64 protein complexes (that were identified/quantified) can be seen in Supplementary File 1.

### Relative dynamics of *E. coli* proteome during growth

**Figure 2:**
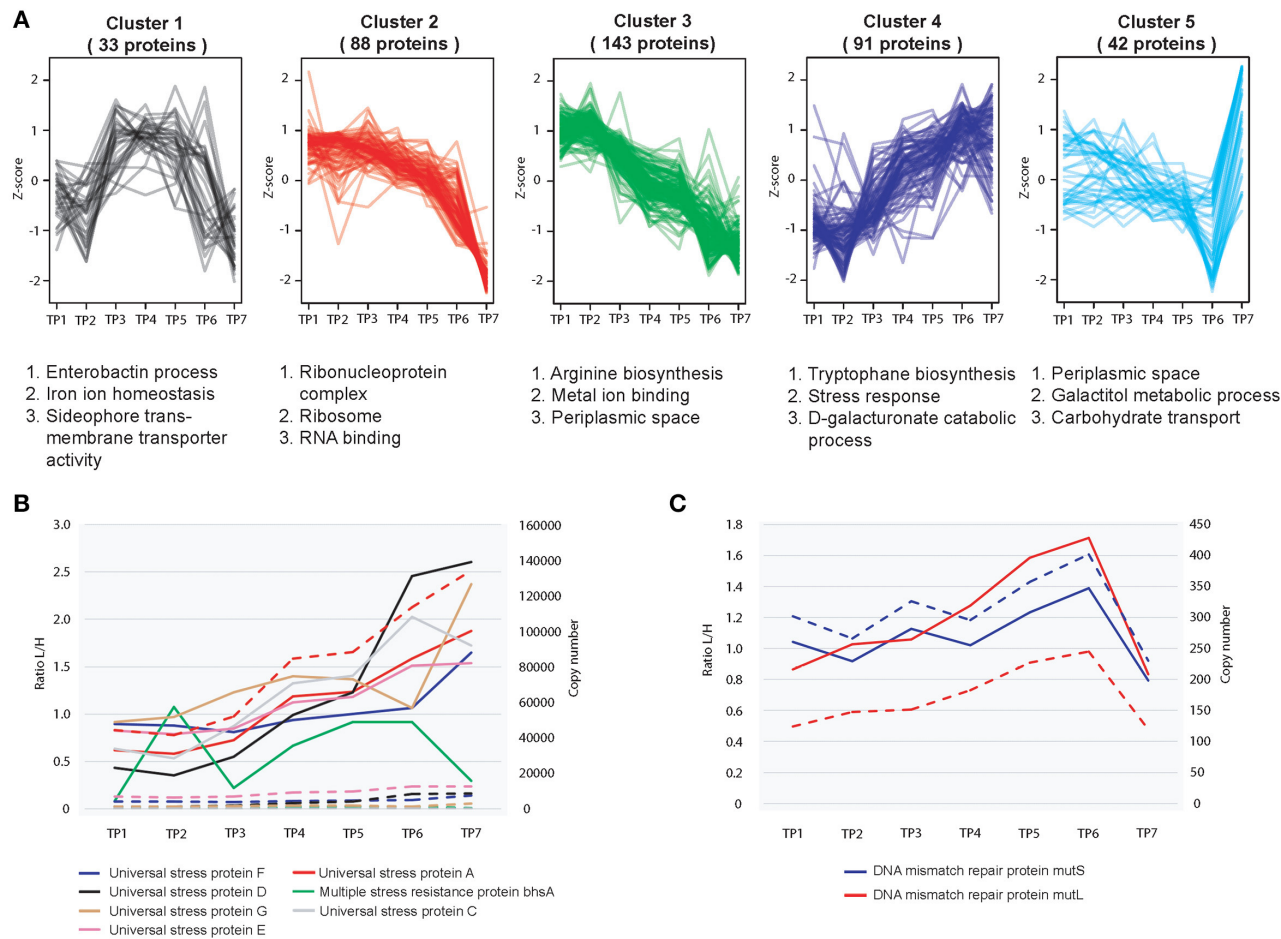
**Relative proteome dynamics during growth in *E. coli*. (A)** Hierarchical clustering analysis of fluctuating proteins reveals distinct growth stage specific changes, resulting in five distinct clusters. Examples of representative classes of proteins based on GO enrichments are depicted below each cluster. **(B)** Relative (solid line) and absolute dynamics (dashed line) of detected universal stress response proteins reveals an increased level of expression during later stages of stationary phase (TP7) **(C)** Relative (solid line) and absolute dynamics (dashed line) of detected mismatch repair proteins mutS and mutL reveals a decreased level of expression during later stages of growth.

For relative quantitation, we assessed the extent of protein fluctuation during different stages of growth and ethanol stress, using the standard deviation of SILAC ratios as described previously (Carpy et al., 2014). To this end we calculated the standard deviations of the log2-transformed protein ratios measured across the analyzed points and binned the proteins into a total of four quartiles (See Experimental Procedures for details). We then performed a functional enrichment analysis for the proteome data (GO analysis) in order to gain insights into which classes of proteins are dynamic and which are static (Supplementary Figure 4). Hierarchical clustering was performed in order to gain a better understanding on significantly changing protein profiles (quartile with top 25% changing proteins) cluster together during the seven different stages of growth (Figure 2A). Enrichment analysis of GO terms revealed association of certain protein clusters with distinct cellular processes (Supplementary Figure 5). For example, during later stages of stationary phase growth in batch culture, an increased expression of multiple universal stress proteins and other proteins involved in stress response occurred (Figure 2B). These proteins continue to increase into the later stages of stationary phase (TP7) despite the fact that the cells are under an enormous amount of growth perturbations, *E. coli* attempts to maintain cellular homeostasis by expressing high levels of stress response proteins. The same is true for protein peak time index (Supplementary Figure 6A), which indicates that proteins associated with stress response are peaking during stationary phase (Supplementary Figure 6B). Furthermore, during stationary phase we observed an increase in the pspA protein is which helps maintain cellular growth during alkaline and nutrient depleted environmental conditions typically associated with stationary phase growth (Weiner and Model, 1994).

*E. coli* as well as other bacterial species have the ability to maintain viability during extended periods of growth during stationary phase. This phenomena is known as the GASP phenotype (Zambrano and Kolter, 1996). This occurs in many Gram negative bacterial species including *E. coli*, and is normally defined as growth in culture for 10 days or longer (Zambrano and Kolter, 1996). As the bacterial cells are encountering several stresses and starvation conditions during extended periods of growth, the bacterial cells are developing increased tolerance toward these harsh environmental conditions through mutations on certain genes, thereby increasing the cellular fitness of these mutants thus enabling a small proportion of the cellular population to survive. Although we did not sample *E. coli* cells growing for a period longer than 10 days, there was evidence of protein activity associated with the GASP phenotype at TP7 (4 days growth). For example, MutS and MutL act as part of the *E. coli* methyl directed mismatch repair system and remove wrongly incorporated bases to ensure both a high fidelity and a low mutation rate of the global protein pool. In the GASP phenotype, lowering the levels of these proteins will increase the gene mutation rate of the bacteria (Finkel, 2006). This promotes a higher level of genetic diversity leading to a greater chance of survival for the bacteria. To this end, expression levels of MutS, MutL decreased during extended growth periods in stationary phase (TP7) (Figure 2C). It has also previously been reported that Dps plays an important role during starvation conditions *in E. coli*, has a significant increase in its synthetic rate during long term growth (6–7 days) (Farrell and Finkel, 2003). Interestingly we observed an approximate 4 fold increase in the levels of Dps between 30 h and 4 days of stationary phase growth, which suggests that this protein could also play an important role in maintaining the cellular integrity of *E. coli* at much earlier periods of stationary phase than previously reported.

Dps is induced by the gene product of the σ^s^ RNA polymerase better known as the *rpoS* gene. *RpoS* serves as a major regulator of multiple genes associated with stress response typically associated with nutrient starvation (Lacour and Landini, 2004). Moreover, many of these Sigma-S dependent genes were found to be specifically induced during the initial onset of stationary phase growth (Lacour and Landini, 2004). Since we did not observe an increase of Dps synthesis (induced by RpoS) until TP7, this prompted us to look for other RpoS regulated targets and observe their proteome dynamics during growth. We observed an increased level of the tnaA encoded tryptophanase enzyme, which acts as an important signaling molecule during stationary phase growth and is also regulated by RpoS (Lacour and Landini, 2004). These observations could suggest that these RpoS regulated proteins have a more important role in extended periods of stationary phase growth rather than at the onset of stationary phase. It is important to note that not all RpoS regulated targets showed this profile type which could be due to the fact that these proteins are specifically induced and regulated by RpoS under different environmental or growth conditions.

Interestingly, we also noticed a dramatic increase in the abundance of the novel aldo-keto reductase protein yghZ (Grant et al., 2003). It is known to assist with methylglyoxal toxicity, a product that is produced in *E. coli* by methylglyoxal synthase (mgsA) which assists to prevent the accumulation of phosphorylated sugars. Despite that fact that we do see a steady increase of mgsA production from TP1-TP7, since its dynamics are similar to those of tnaA and DPs could suggest that it might play an important role during later stages of stationary growth.

### Relative proteome dynamics during ethanol stress

**Figure 3:**
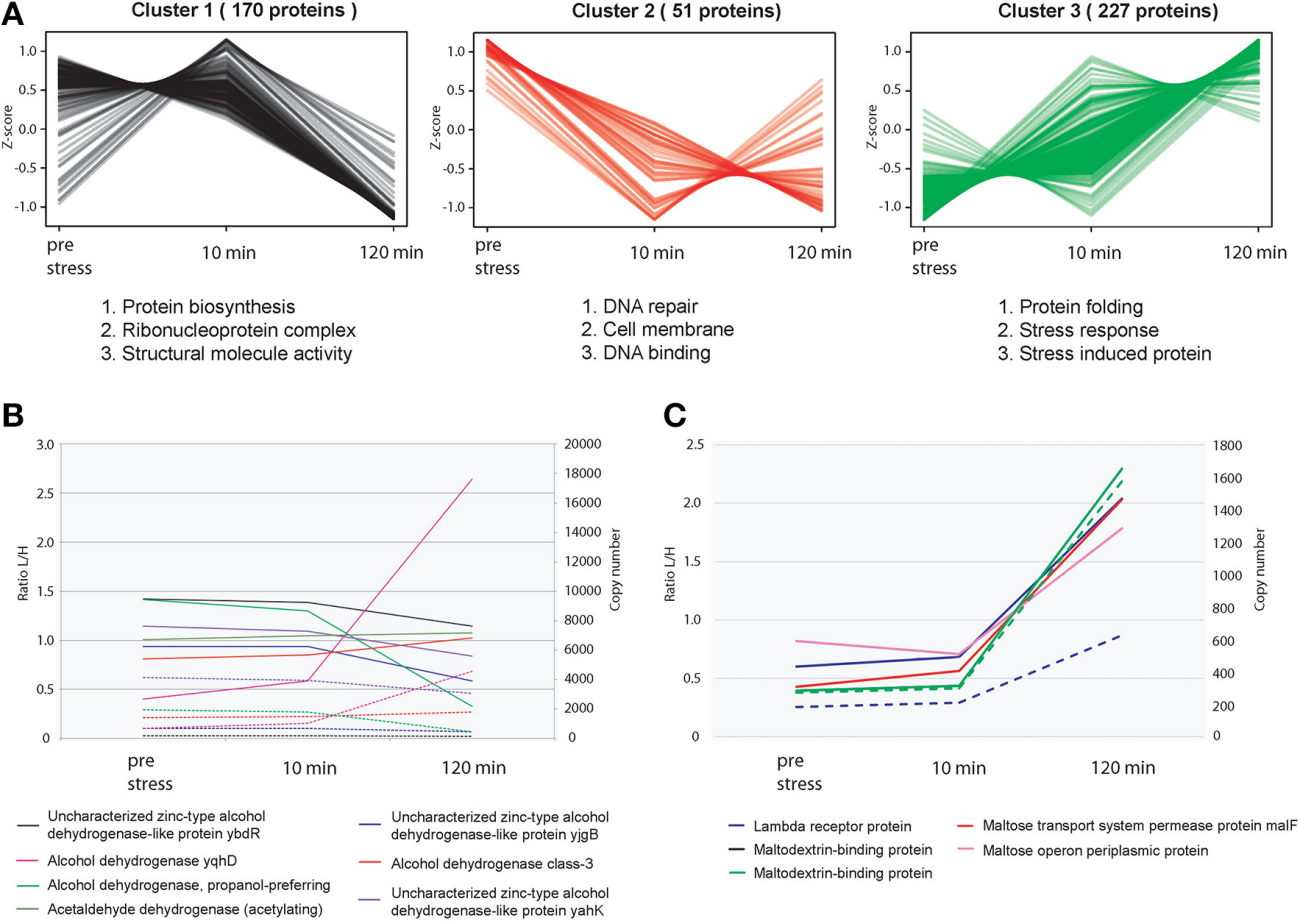
**Relative proteome dynamics during ethanol stress in *E. coli*. (A)** Hierarchical clustering analysis of fluctuating proteins reveals distinct specific changes at each time point, resulting in three distinct clusters. Examples of representative classes of proteins based on GO enrichments are depicted below each cluster. **(B)** Relative (solid line) and absolute dynamics (dashed line) of detected alcohol dehydrogenases confirms that upon ethanol stress, the alcohol dehydrogenase of *E. coli* (yqhD) has the highest level of expression compared to other alcohol dehydrogenases. **(C)** Relative (solid line) and absolute dynamics (dashed line) of detected proteins associated with the binding and transport of maltose.

In response to alcohol stress, bacteria undergo many changes on both the physical and physiological levels. This includes but is not limited to changes in fatty acid composition, loss of ions from membrane leakage, an increase in membrane fluidity, and a decreased level of translation (Ingram, 1990). We extended the Super-SILAC approach to study the global changes of the proteome upon stress with ethanol. Hierarchal clustering analysis reveals very distinct changes on the proteome upon 10 min and 2 h post ethanol stress as opposed to pre stress conditions (Figure 3A). This is as expected, as with an acute and rapid perturbation such as ethanol stress, many physiological changes will be employed by *E. coli* in a rapid fashion. Peak time index analysis of the top 25% most changing proteins revealed that those proteins belonging to general stress response and heat shock response had the highest increase in expression levels during extended periods of ethanol stress (2 h) in both biological replicates (Supplementary Figure 7). Heat shock proteins are commonly known to be up-regulated during many different types of stresses including but not limited to ethanol stress. Supplementary Figure 8A shows many heat shock proteins are induced upon ethanol stress.

The alcohol dehydrogenase YqhD and aldehyde dehydrogenase AldB were highly induced upon ethanol stress as expected. In mesophilic bacteria, it has been previously reported that when submitted to alcohol stresses, they attempt to make a balance between stress response and growth (Huffer et al., 2011). In this dataset, we observed that many stress response pathways such as the universal stress proteins (Supplementary Figure 8B), and multiple stress resistance proteins (ex BhsA, RpoH etc) were induced. However, key pathways critical for growth such as the glycolysis/gluconeogenesis pathways overall remained at a constant level. Figure 3B depicts all alcohol dehydrogenases identified in this study. As expected the main alcohol dehydrogenase yqhD is highly induced upon the addition of ethanol, however it appears that once yqhD is activated other alcohol dehydrogenases such as the propanol preferring alcohol dehydrogenase are down-regulated suggesting a type of a highly controlled network by different alcohol dehydrogenase members.

Many proteins involved in carbohydrate synthesis and transport were also found to be significantly regulated during exposure to ethanol. Interestingly, many of these regulated genes were also previously found to be over expressed during response to butanol (Rutherford et al., 2010) suggesting that many of these proteins play a role in responding to general solvent stress. For example, the manXYZ system is known to play a role in tolerating solvent stress in *E. coli* (Okochi et al., 2007). To this end, we also observed an up-regulation of manX and manZ upon ethanol stress at virtually identical levels which is expected since they are expressed from the same operon, and fall within the same range in terms of protein copies/cell. Unfortunately no quantification data could be obtained for ManY. We also detected an up-regulation of the maltose-binding protein MalE as well as other proteins related to the transport of Maltose into the cell (Figure 3C). Maltose is known to exhibit certain properties similar to that of chaperones, in an attempt to prevent the aggregation of proteins during heat shock stress (Richarme and Caldas, 1997).

Another noteworthy observation is with regards to the YehU/YehT system in *E. coli.* This two component signaling transduction system was never characterized until very recently where this system was reported to be involved in the stationary phase control network of *E. coli* (Kraxenberger et al., 2012). Moreover, it showed that currently the only known and exclusive substrate of YehT is YjiY, an inner membrane protein that is a member of the CstA transporter superfamily (Kraxenberger et al., 2012). This superfamily is typically associated with the transport of peptides and amino acids. The authors reported that upon the onset of stationary phase, YjiY is strongly induced. An up regulation of YjiY was observed during late stationary phase growth and ethanol stress in both biological replicates, however YjiY was the most strongly induced protein after 10 min post ethanol stress. Currently the exact function of YjiY is not known. Therefore, from these observations one could speculate that perhaps YjiY (an inner membrane transporter) acts as a general stress response protein or perhaps also very specific to ethanol stress itself. Upon stress it could be feasible that it removes peptides and amino acids caused from misfolded proteins and degraded proteins via proteases. Further experimental validation on the potential role and function of YjiY during ethanol stress is required in order to understand the exact function of this protein.

How well bacteria can maintain their membrane integrity is a critical survival factor during ethanol stress. Upon ethanol stress, a common phenomenon is that the bacterial membrane increases in fluidity. This results in a sudden loss in the control of solute transportation (Huffer et al., 2011). This can cause a detrimental effect on the viability and stability of bacteria due to the rapid loss of many important ions such as magnesium (Ingram, 1976). In *E. coli*, membrane fluidity increases approximately 15% upon ethanol treatment (Huffer et al., 2011). One strategy that many bacteria employ to circumvent this increase in fluidity is known as lipid mixing. The bacteria replace the lipid content with stronger more complex unsaturated fatty acids in an attempt to maintain cell membrane rigidity. In *E. coli*, the key genes involved in unsaturated fatty acid synthesis are *faba* and *fabb* (Feng and Cronan, 2009). We observed that these proteins as well as many other members of the fab protein family exhibited minor changes during ethanol stress (Supplementary Figure 8C). This observation could be one of the reasons why the *E. coli* membrane increases in fluidity hence ceasing the growth of WT *E. coli* strains upon ethanol stress. For further insights with respect to this section, please refer to the Supplemental text section.

### Unbiased detection of protein modifications during growth and ethanol stress

To perform a global screen of protein modifications during growth and ethanol stress we employed the “DP” algorithm which is a part of the MaxQuant software suite. This algorithm can identify co-translational and post translational modifications, modifications encountered during sample preparation, unknown modifications, as well as amino acid substitutions in a systematic and unbiased fashion. To this end, MaxQuant uses all MS/MS spectra that were identified during the regular database search and compares them to all unidentified MS/MS spectra. It then calculates precursor mass differences between identified spectra (“base peptides”) and unidentified spectra with similar MS/MS features (“dependent peptides”). Therefore, the algorithm reports mass differences that correspond to possible modifications on dependent peptides. This can be used as a powerful approach to detect various modifications without specifically searching for them, and thus has great potential for discovering novel protein modifications. This algorithm works in a similar way as previously reported (Savitski et al., 2006).

Since our dataset contained SILAC label we first tested the accuracy of the algorithm by processing the data without defining any SILAC labels. As expected, DP analysis revealed high number of dependent peptides that contained a Lys8 label (Supplementary Files 2, 3). We then implemented this feature in our experiments in order to elucidate which modifications are present during growth and ethanol stress, and whether these modifications are growth state dependent or ethanol stress dependent.

In all experiments, we observed more than 50 distinct mass differences that were detected in more than 50 MS/MS spectra. Most of them corresponded to known modifications that most likely result from sample preparation (e.g., deamidation, methionine oxidation, carbamidomethylation, etc.), as well as other known and unknown PTMs that could potentially have a biological or regulatory relevance (Tables S8, S9). For both growth and ethanol stress datasets, we compiled the 10 annotated modifications that occurred with highest frequency (Table 2). Utilizing a pairwise comparison of each growth stage relative to TP3 (Supplementary File 2), and each ethanol stress time point relative to pre stress (Supplementary File 3). We did observe subtle changes of many different PTMs, which could be due to both the specific environmental/biological growth condition, or technical variations due to sample preparation (Supplementary Files 2, 3). There was very little detection of some known regulatory modifications, such as protein phosphorylation, stressing the importance of enrichment steps prior to MS analysis for those PTMs that are of low abundance and stoichiometry.

**Table 2:** **Frequent annotated modifications observed during growth and ethanol stress**.

**Modification (Growth)**	**Average mass shift (Da)**	**Most frequent amino acid**	**Max# dependent peptides (growth)**	**Max# dependent peptides (stress)**
Deamidation	0.984	Asparginine	2031	1675
Carbamidomethyl DTT	151.995	n-terminal	814	475
di-Oxidation	31.988	Tryptophan	1851	1023
Sulfide	31.971	Cysteine	401	586
Acetylation	42.010	n-terminal	1116	1289
Oxidation	15.994	Tyrosine	3951	2172
Loss of ammonia	−17.026	n-terminal	999	795
Loss of water	−18.010	n-terminal	1044	1046
Formylation	27.994	n-terminal	319	123
Acetaldehyde	26.015	n-terminal	392	795

**Figure 4:**
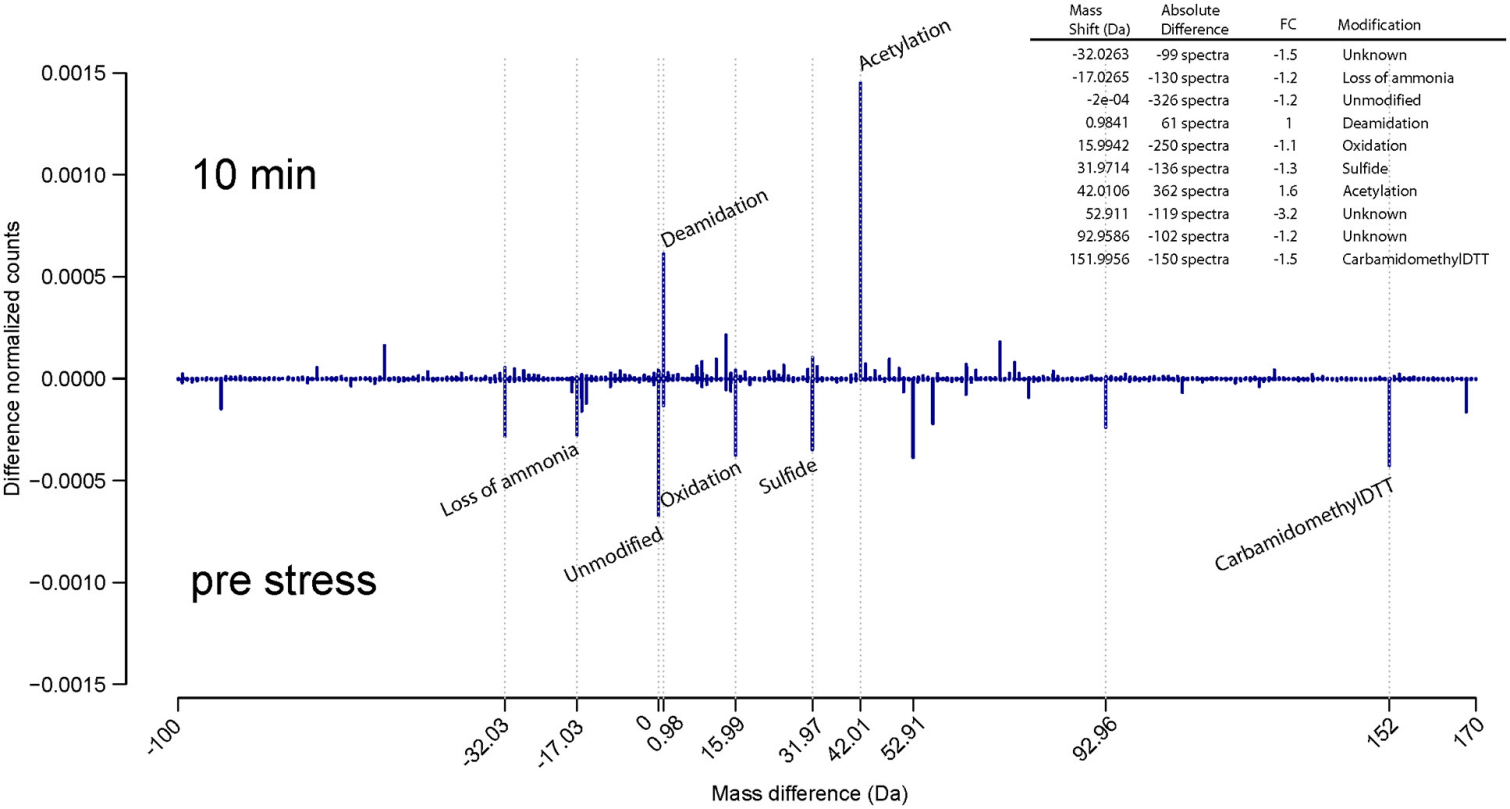
**Unbiased detection of protein modifications during ethanol stress**. Comparison of protein modification that occur in pre-stress vs. 10 min ethanol stress reveals the presence of many post translational modifications associated with sample preparation, but also the presence of an increased amount of acetylation likely due to the production of acetate associated with ethanol metabolism. The Y axis represents the difference between the normalized counts (absolute counts divided by the number of sequenced precursors) of each modification. The legend lists the absolute difference and fold change (FC).

Interestingly, during ethanol stress, already after 10 min post stress, a strong increase in the level of acetylation was encountered relative to pre stressed *E. coli* cells (Figure 4). A similar pattern was detected after 2 h post ethanol stress treatment. This increased level of protein acetylation during ethanol stress in a bacteria is a novel observation, likely resulting from conversion of ethanol into acetate, which can then bind randomly to amino acid residues of the protein in a non-enzymatic fashion. However, further experiments will be needed to confirm this.

## Conclusions and future perspectives

In this study, we used the Super-SILAC approach to investigate global relative proteome dynamics in *E. coli* during growth and ethanol stress grown in minimal medium. Moreover, we estimated the absolute protein copy number per cell in *E. coli* using the iBAQ. To this end, we identified 2303 proteins which represents approximately 88% of the estimated expressed proteome of *E. coli.* The estimates of protein copy numbers of over 2000 proteins were derived. Unbiased detection of protein modifications revealed an increased level of protein acetylation after only 10 min post ethanol stress. Overall, this dataset represents the most comprehensive relative and absolute quantitative analysis of bacterial growth during normal and ethanol stress conditions at the proteome level and implicates both known as well as novel observations of *E. coli* proteome dynamics during growth and ethanol stress in minimal medium.

## Author contributions

BS and BM designed the experiments. BS and AH performed bacterial cell culture/ counting, MS measurements, proteome analysis and data processing. BS and KK performed bioinformatics analysis. BS and BM wrote the manuscript.

### Conflict of interest statement

The Guest Associate Editor Ivan Mijakovic declares that, despite having coauthored an article with the authors Boris Macek and Karsten Krug in 2014, the review process was handled objectively. The authors declare that the research was conducted in the absence of any commercial or financial relationships that could be construed as a potential conflict of interest.
