# Supplementary material for: Characterization of the E. coli proteome and its modifications during growth and ethanol stress
Source: Front Microbiol. 2015 Feb 18;6:103. doi: 10.3389/fmicb.2015.00103 (PMC4332353; doi:10.3389/fmicb.2015.00103)
Supplement: Supplementary file 1 [file DataSheet1.ZIP › Supplementary Figures 1-12.pptx]

## Slide 1
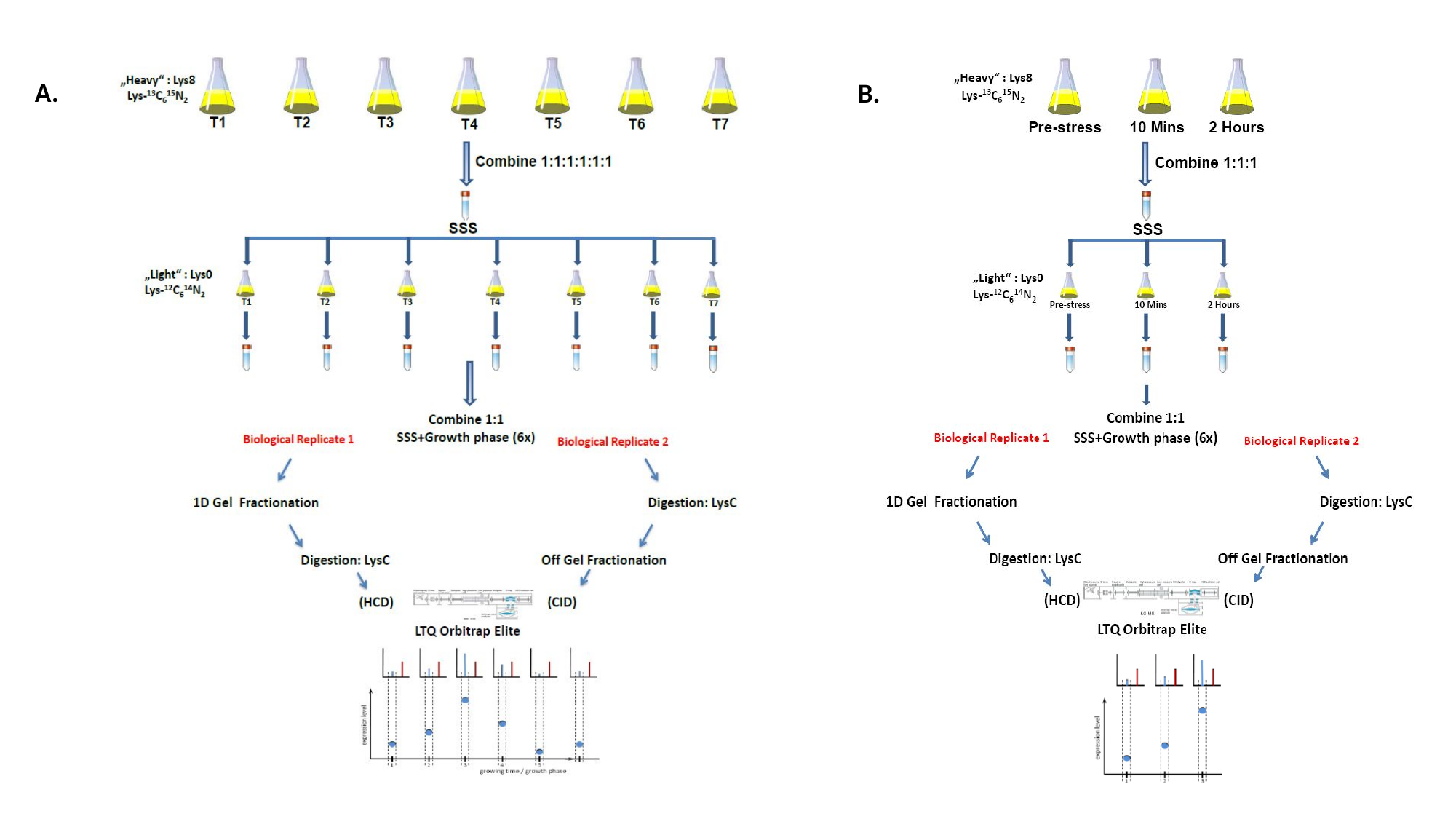

A.
B.

## Slide 2
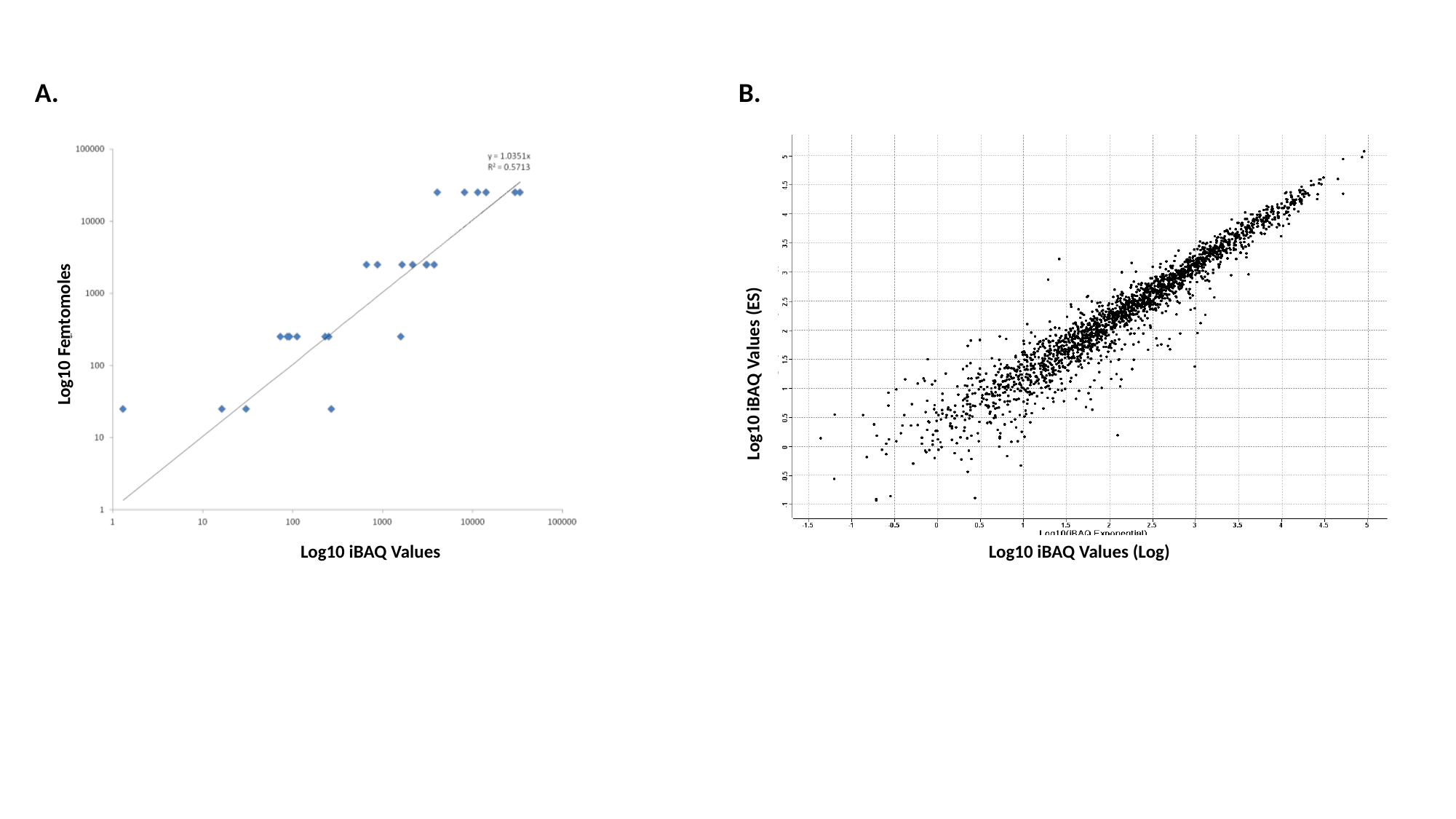

B.
A.
Log10 Femtomoles
Log10 iBAQ Values
Log10 iBAQ Values (ES)
Log10 iBAQ Values (Log)

## Slide 3
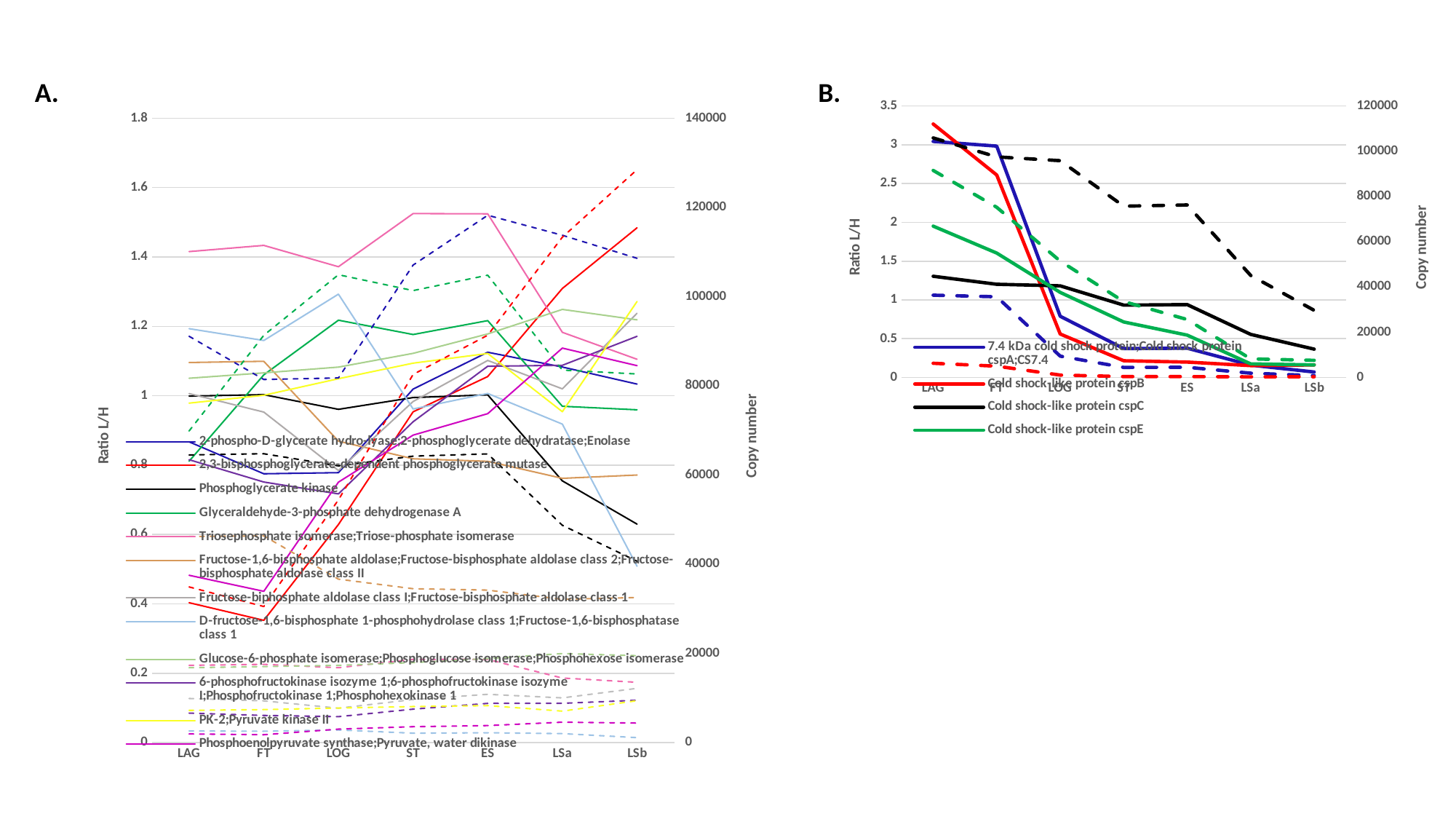

B.
A.
### Chart
| Category | 7.4 kDa cold shock protein;Cold shock protein cspA;CS7.4 | Cold shock-like protein cspB | Cold shock-like protein cspC | Cold shock-like protein cspE | | | | |
|---|---|---|---|---|---|---|---|---|
| LAG | 3.043584 | 3.268828 | 1.304921 | 1.952019 | 36387.24620734475 | 6270.937538073362 | 105902.29994427382 | 91546.9639599119 |
| FT | 2.983917 | 2.611511 | 1.201865 | 1.604518 | 35673.90370736656 | 5009.937005248212 | 97538.67684137558 | 75249.65254899155 |
| LOG | 0.7893283 | 0.5607267 | 1.181251 | 1.09737 | 9436.730903607353 | 1075.7011722947798 | 95865.72498371426 | 51465.119878796526 |
| ST | 0.3724811 | 0.2158802 | 0.9332712 | 0.7158197 | 4453.158346634298 | 414.1457580229932 | 75740.65138943458 | 33570.94386770567 |
| ES | 0.377829 | 0.1986808 | 0.9399379 | 0.5464182 | 4517.094598760824 | 381.15033486449755 | 76281.69476526997 | 25626.250186314748 |
| LSa | 0.1594591 | 0.1530573 | 0.555679 | 0.1751498 | 1906.396383901876 | 293.6259625915331 | 45096.74082242077 | 8214.280920516541 |
| LSb | 0.07069635 | 0.1620588 | 0.3655773 | 0.162064 | 845.2027259344961 | 310.89448949137835 | 29668.828133977284 | 7600.575182515725 |
### Chart
| Category | 2-phospho-D-glycerate hydro-lyase;2-phosphoglycerate dehydratase;Enolase | 2,3-bisphosphoglycerate-dependent phosphoglycerate mutase | Phosphoglycerate kinase | Glyceraldehyde-3-phosphate dehydrogenase A | Triosephosphate isomerase;Triose-phosphate isomerase | Fructose-1,6-bisphosphate aldolase;Fructose-bisphosphate aldolase class 2;Fructose-bisphosphate aldolase class II | Fructose-biphosphate aldolase class I;Fructose-bisphosphate aldolase class 1 | D-fructose-1,6-bisphosphate 1-phosphohydrolase class 1;Fructose-1,6-bisphosphatase class 1 | Glucose-6-phosphate isomerase;Phosphoglucose isomerase;Phosphohexose isomerase | 6-phosphofructokinase isozyme 1;6-phosphofructokinase isozyme I;Phosphofructokinase 1;Phosphohexokinase 1 | PK-2;Pyruvate kinase II | Phosphoenolpyruvate synthase;Pyruvate, water dikinase | | | | | | | | | | | | |
|---|---|---|---|---|---|---|---|---|---|---|---|---|---|---|---|---|---|---|---|---|---|---|---|---|
| LAG | 0.8670771 | 0.4031771 | 0.9989012 | 0.8111616 | 1.415448 | 1.095758 | 1.006158 | 1.193289 | 1.050365 | 0.8153946 | 0.9786651 | 0.4822764 | 91103.53902831611 | 34901.27139142979 | 64500.389482273175 | 69881.50098730373 | 17318.84206546495 | 46203.430942799874 | 9882.230183580656 | 2611.2789911537143 | 16789.64026162229 | 6603.518974826281 | 7228.365584392828 | 1940.146764681762 |
| FT | 0.7748934 | 0.3524229 | 1.003019 | 1.060131 | 1.433363 | 1.099227 | 0.9528347 | 1.159152 | 1.065825 | 0.751089 | 1.001302 | 0.4361099 | 81417.82444685086 | 30507.703134564737 | 64766.281348065415 | 91330.19305052322 | 17538.042668809474 | 46349.70384424396 | 9358.502175903803 | 2536.5768603865536 | 17036.761822646014 | 6082.736460706628 | 7395.560459225232 | 1754.4238356483686 |
| LOG | 0.7781495 | 0.6286541 | 0.9607994 | 1.21776 | 1.371836 | 0.8691118 | 0.7843137 | 1.292791 | 1.08245 | 0.7168973 | 1.049043 | 0.7507507 | 81759.94192801847 | 54419.82532101908 | 62040.10518190825 | 104909.91763207108 | 16785.223493706002 | 36646.72950858903 | 7703.3314047454 | 2829.0196073646885 | 17302.505415920226 | 5805.833057456756 | 7748.1728098286185 | 3020.1903756592033 |
| ST | 1.019119 | 0.9536525 | 0.9947279 | 1.176415 | 1.525111 | 0.8180628 | 0.9832842 | 0.9617234 | 1.12212 | 0.9249838 | 1.093876 | 0.8863676 | 107078.53729616258 | 82553.50989829407 | 64230.91390708478 | 101348.04949344117 | 18660.633623632457 | 34494.21139218103 | 9657.569487374703 | 2104.543081953257 | 17936.61358705936 | 7491.033267459606 | 8079.306835395776 | 3565.7627689406704 |
| ES | 1.125366 | 1.055186 | 1.002496 | 1.216486 | 1.52432 | 0.8108327 | 1.10171 | 1.006573 | 1.178134 | 1.084987 | 1.121781 | 0.9482268 | 118241.87872351834 | 91342.81920882221 | 64732.51053699898 | 104800.16264335143 | 18650.955271567396 | 34189.34898090086 | 10820.717835123949 | 2202.6876372467755 | 18831.973685324738 | 8786.828171218993 | 8285.41160160485 | 3814.6157643304555 |
| LSa | 1.082685 | 1.30914 | 0.7544892 | 0.969556 | 1.18276 | 0.7617887 | 1.019753 | 0.918105 | 1.24886 | 1.087642 | 0.9540164 | 1.137255 | 113757.3984515015 | 113326.50199968301 | 48718.37901503041 | 83527.16471199605 | 14471.766989214242 | 32121.373143938068 | 10015.756845740849 | 2009.0927664406365 | 19962.498880988624 | 8808.329837869918 | 7046.311667501316 | 4575.056148026644 |
| LSb | 1.033496 | 1.484054 | 0.6298022 | 0.9594167 | 1.105009 | 0.7713074 | 1.237823 | 0.5082592 | 1.218799 | 1.171207 | 1.27165 | 1.086744 | 108589.12450992948 | 128468.03901694056 | 40667.17228570003 | 82653.66490263554 | 13520.43759425804 | 32522.736034389454 | 12157.585401627135 | 1112.225597504539 | 19481.986510617724 | 9485.085684832062 | 9392.335636974425 | 4371.855756651822 |

## Slide 4
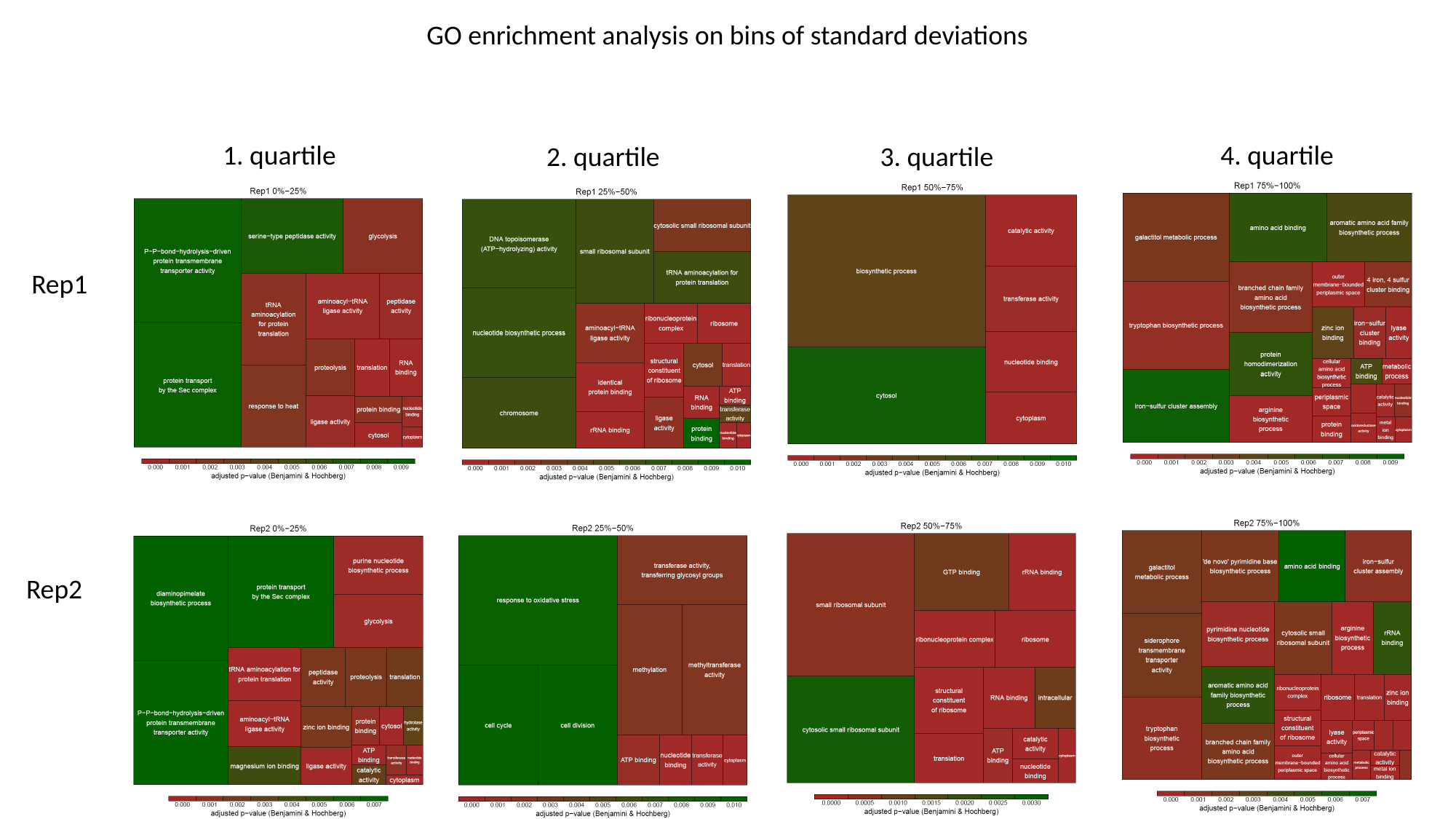

GO enrichment analysis on bins of standard deviations
1. quartile
4. quartile
2. quartile
3. quartile
Rep1
Rep2

## Slide 5
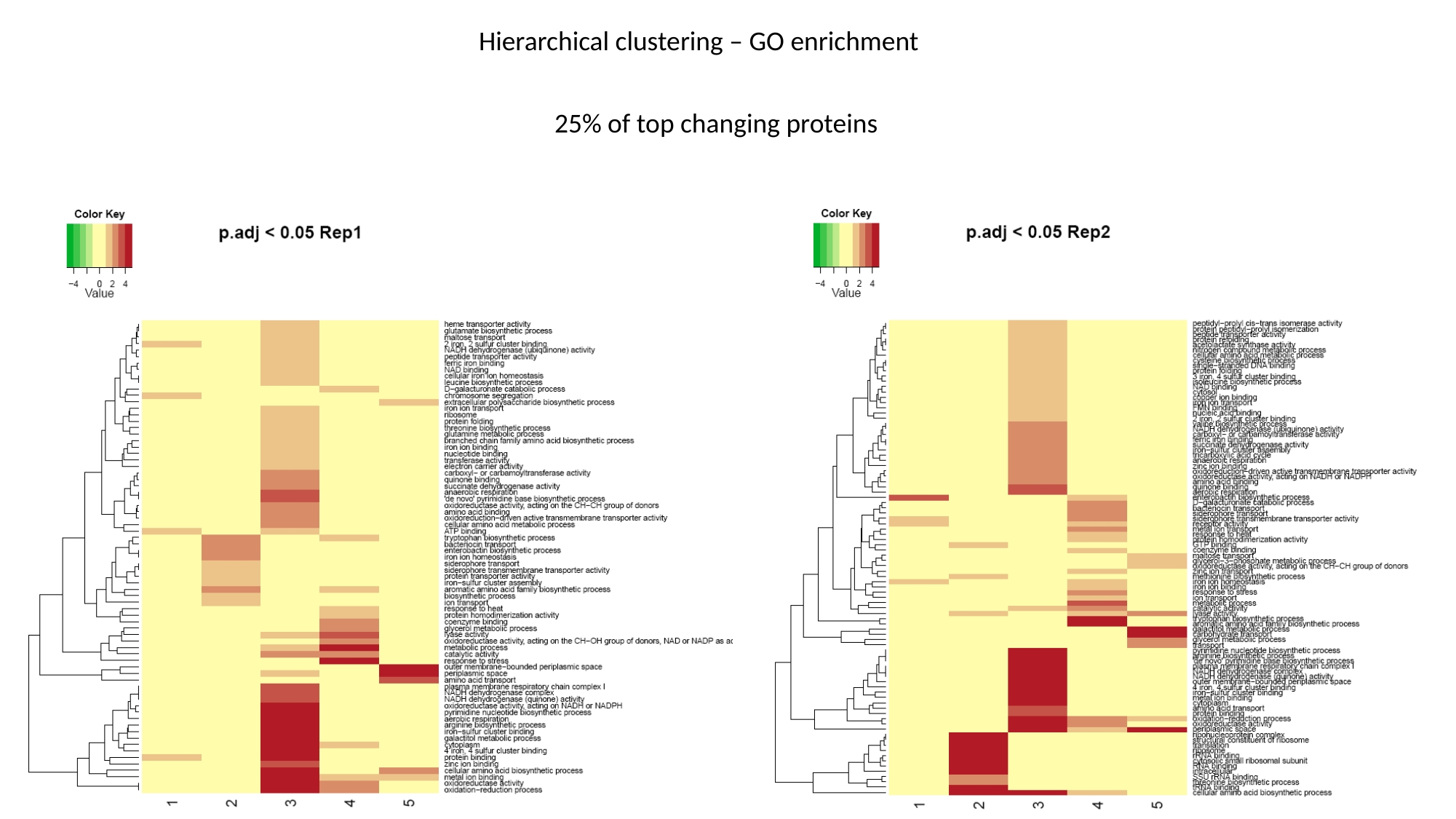

Hierarchical clustering – GO enrichment
25% of top changing proteins

## Slide 6
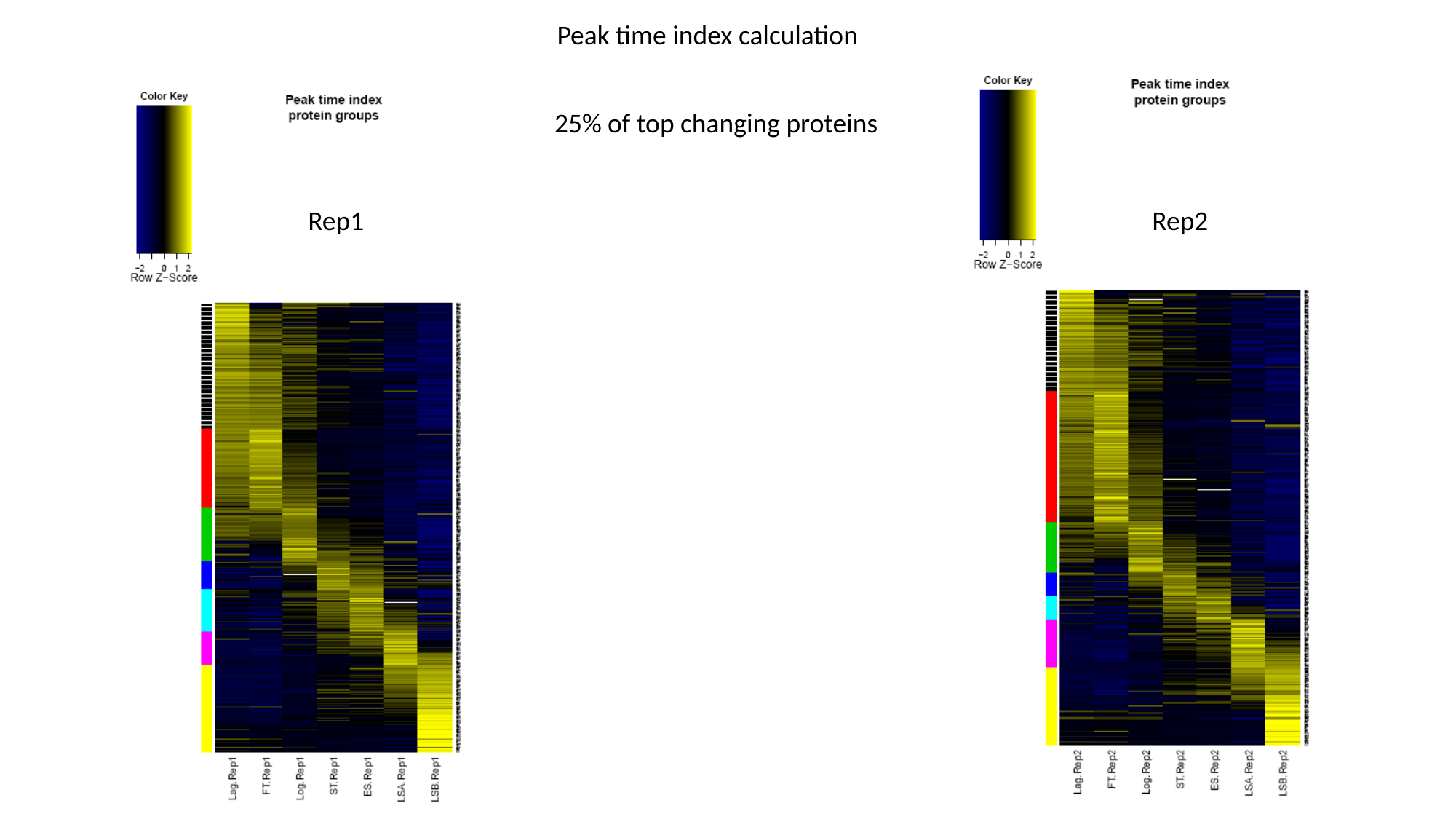

Peak time index calculation
25% of top changing proteins
Rep1
Rep2

## Slide 7
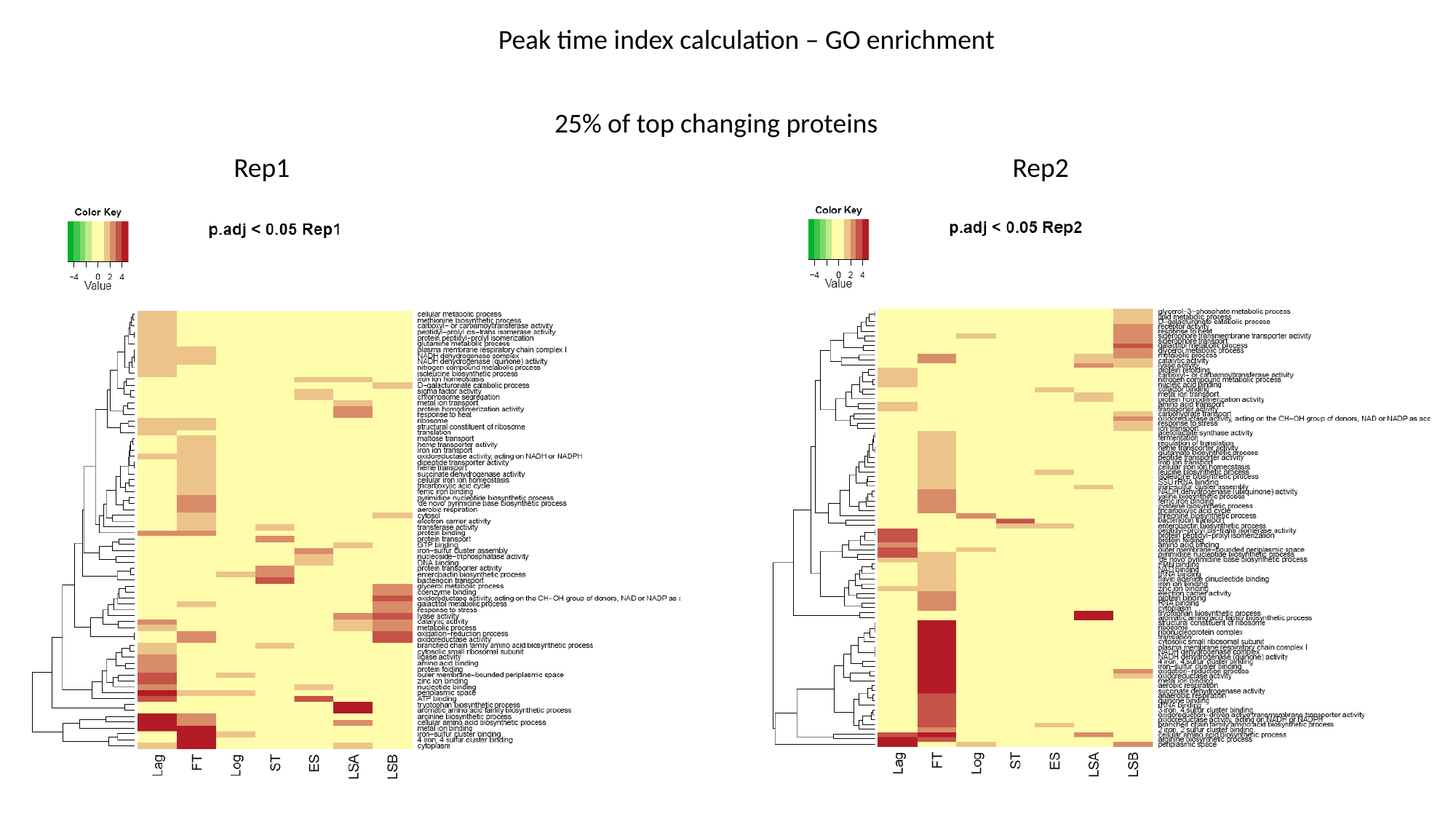

Peak time index calculation – GO enrichment
25% of top changing proteins
Rep1
Rep2

## Slide 8
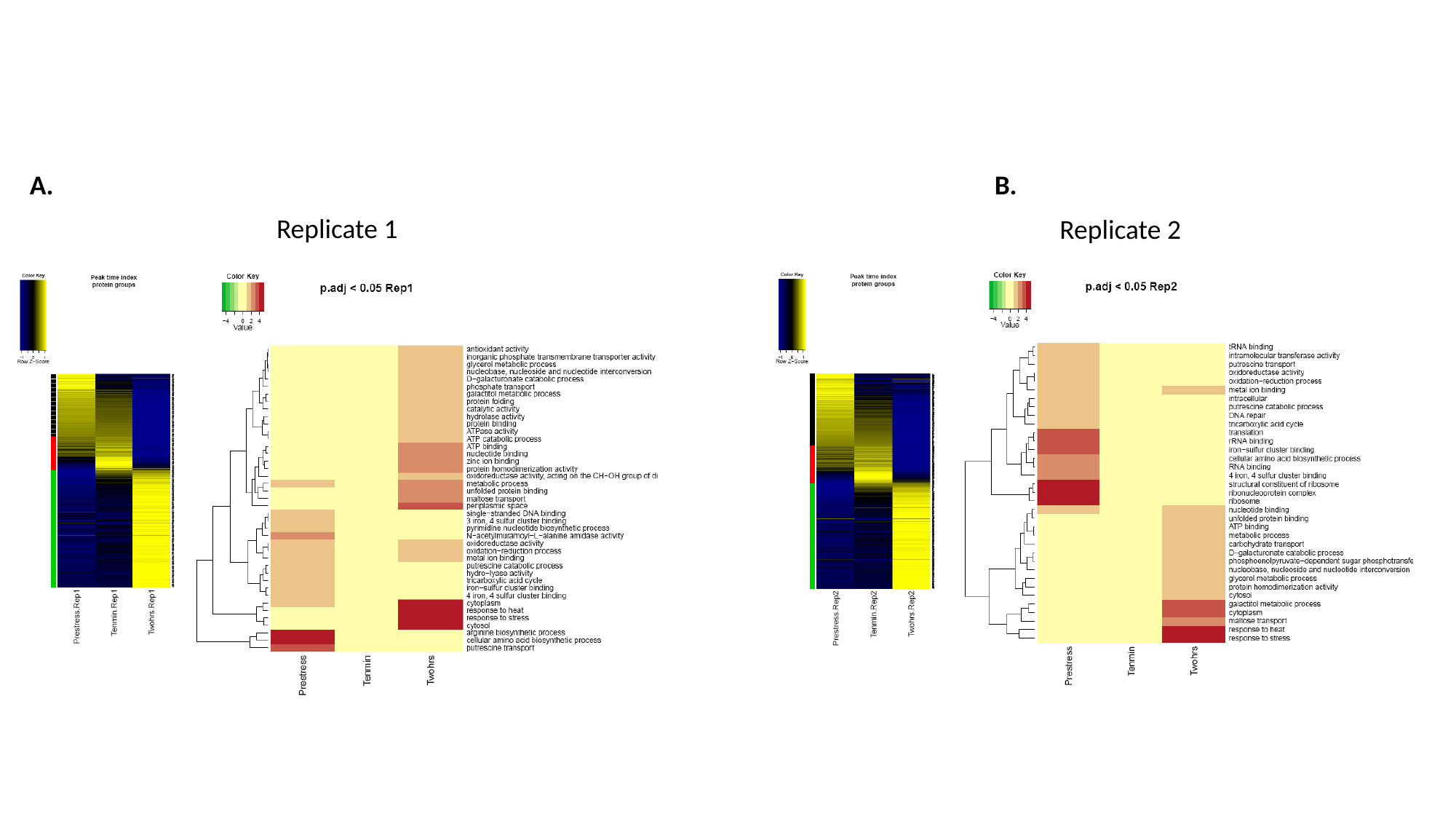

A.
B.
Replicate 1
Replicate 2

## Slide 9
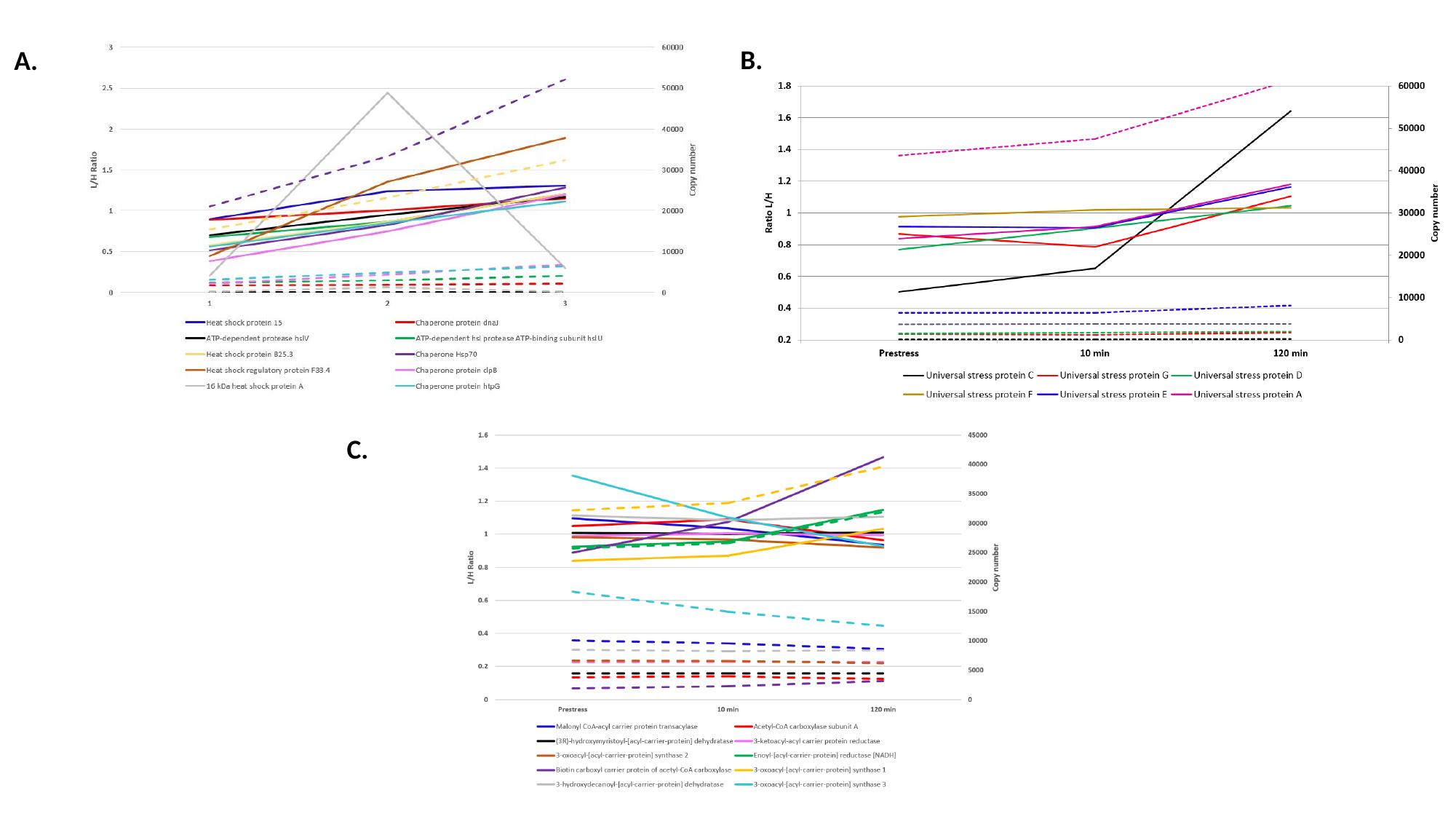

B.
A.
C.

## Slide 10
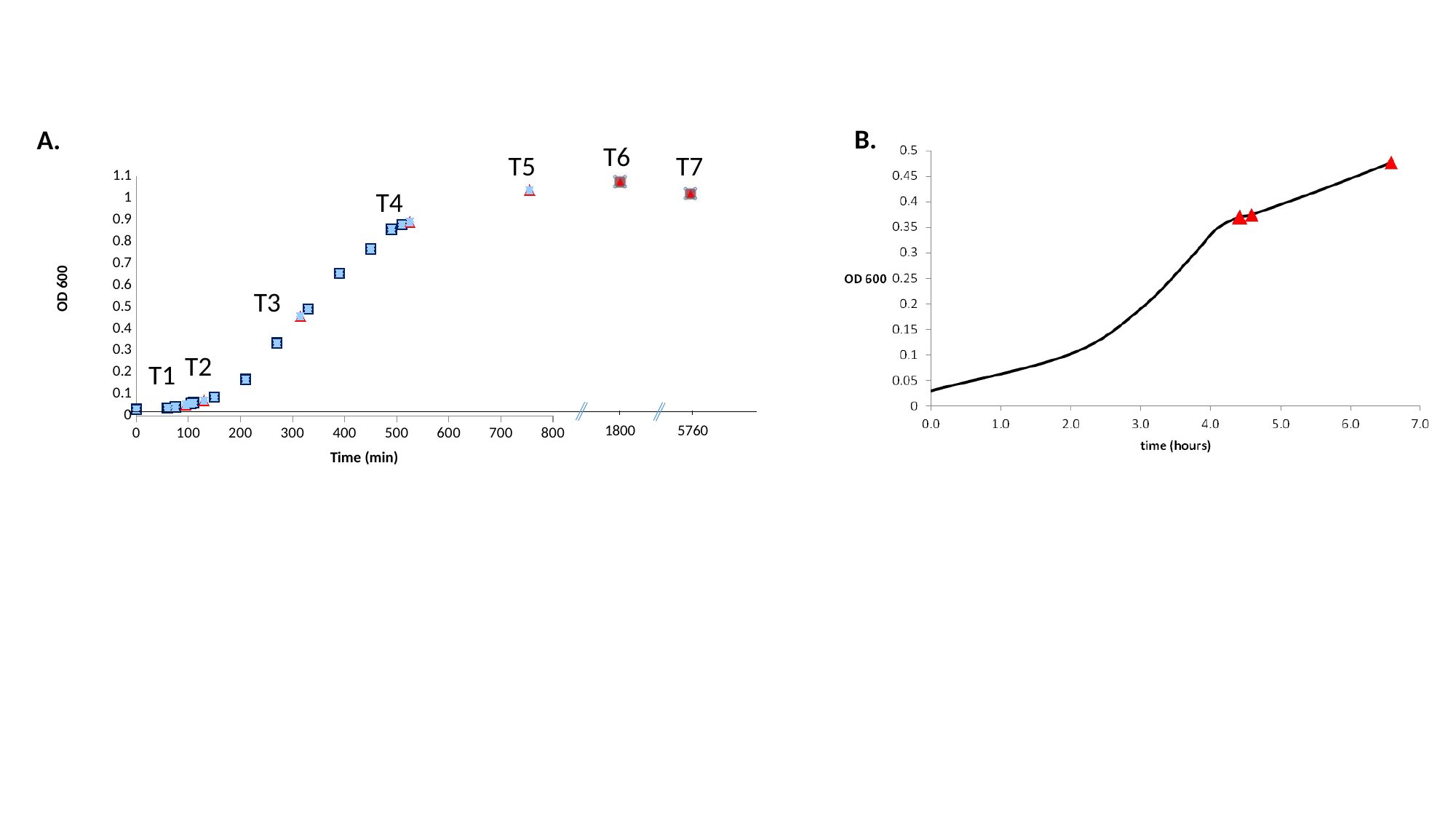

B.
A.
T6
T5
T7
### Chart
| Category | Light Culture | Light Culture | Light Culture | Light Culture |
|---|---|---|---|---|
T4
OD 600
T3
T2
T1
1800
5760
Time (min)

## Slide 11
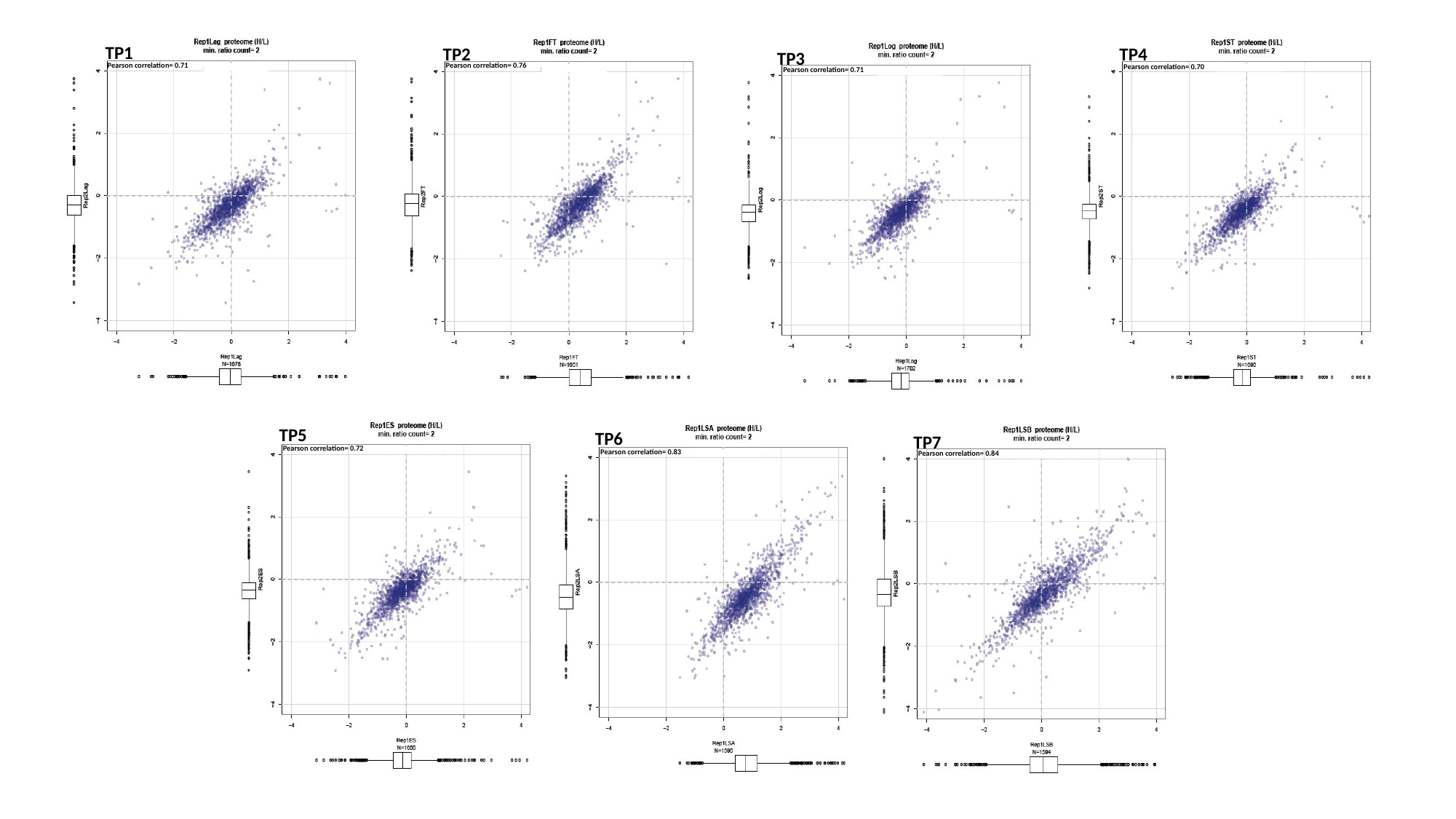

Pearson correlation= 0.76
Pearson correlation= 0.71
Pearson correlation= 0.70
Pearson correlation= 0.71
Pearson correlation= 0.72
Pearson correlation= 0.83
Pearson correlation= 0.84
TP1
TP2
TP4
TP3
TP5
TP6
TP7

## Slide 12
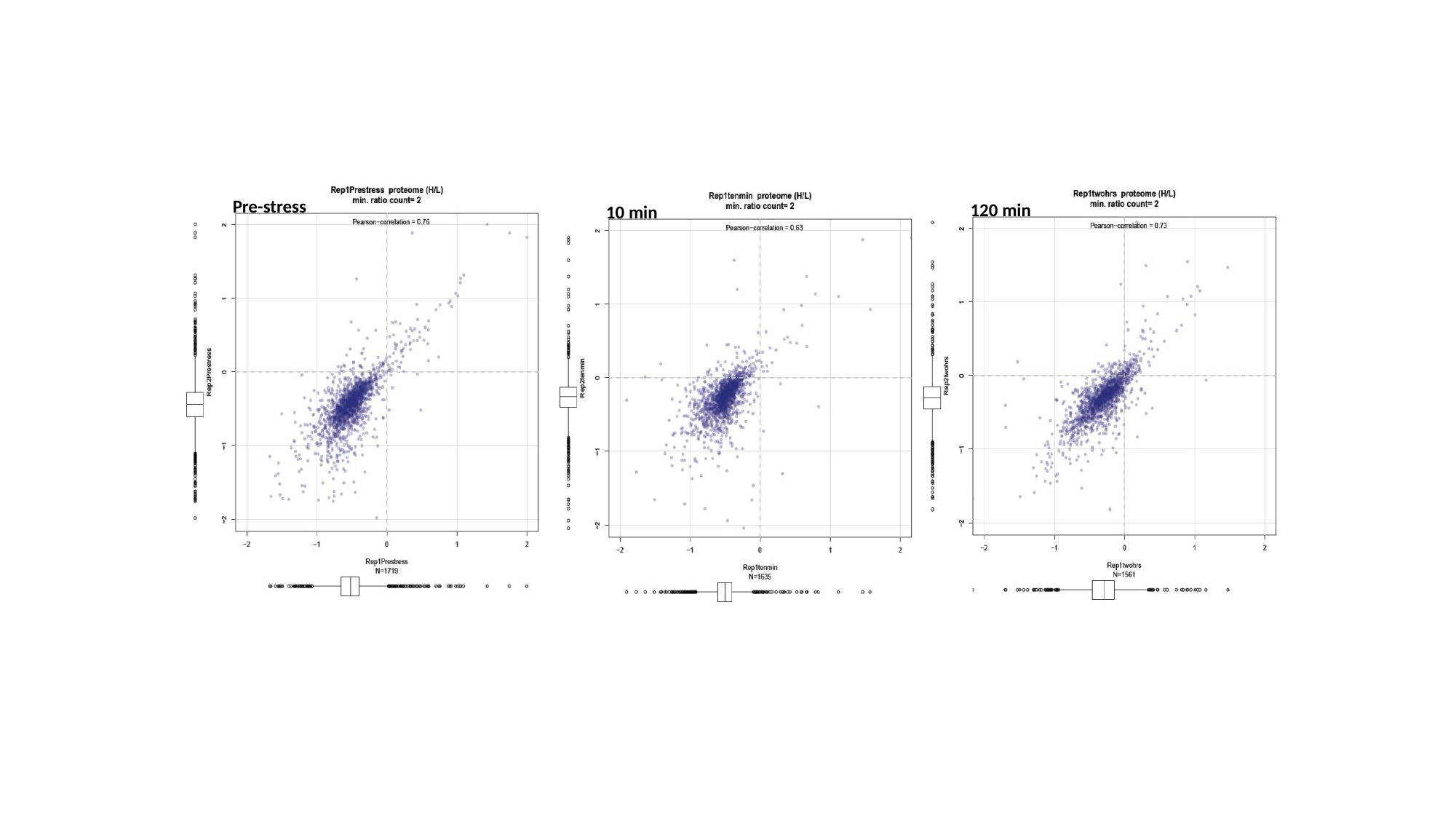

Pre-stress
120 min
10 min

## Slide 13
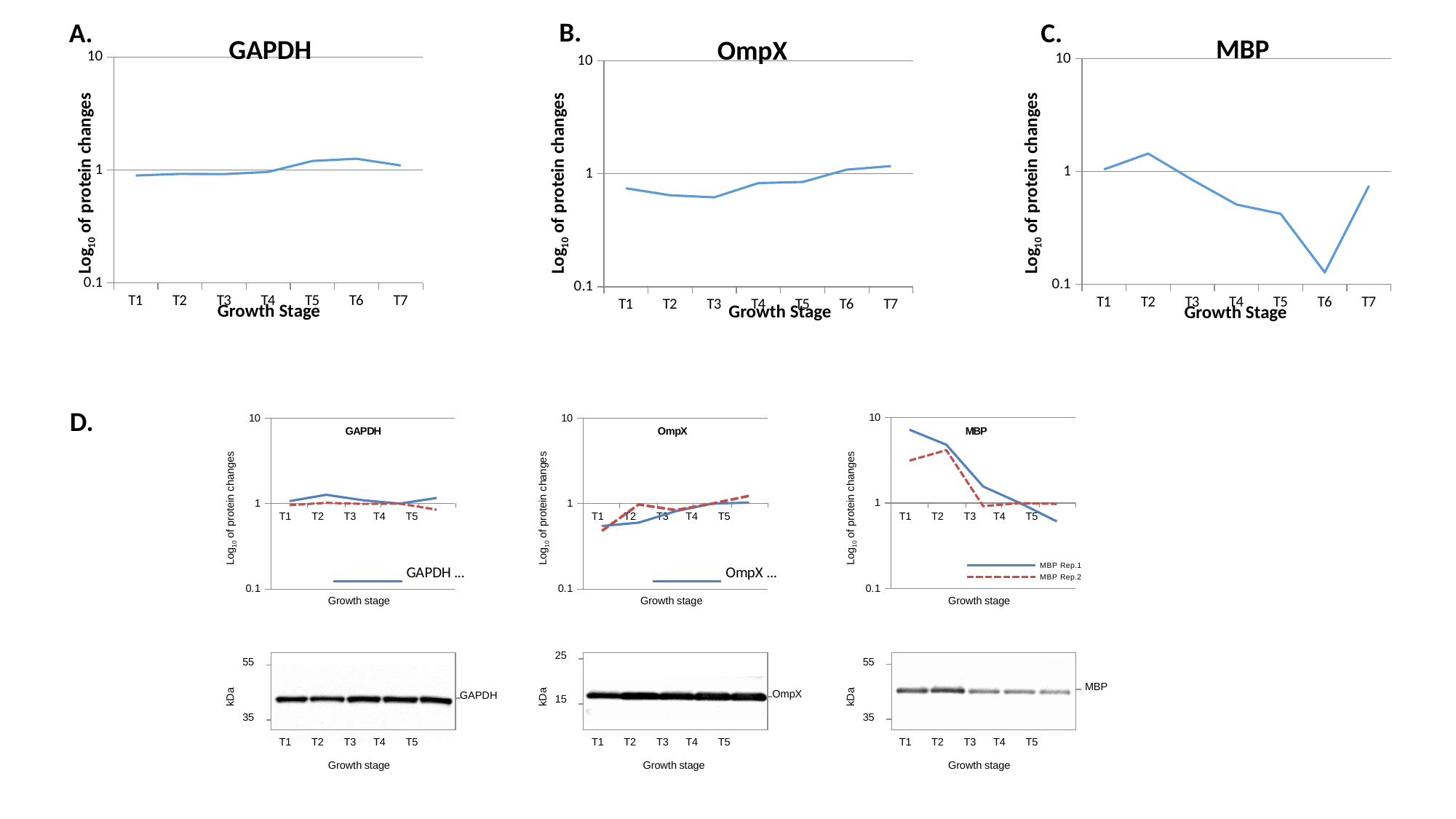

B.
C.
A.
MBP
GAPDH
OmpX
### Chart
| Category | |
|---|---|
| T1 | 0.8954956568460642 |
| T2 | 0.9248127254231019 |
| T3 | 0.921234454168586 |
| T4 | 0.9629272989889264 |
| T5 | 1.2037605479518014 |
| T6 | 1.2605889471561114 |
| T7 | 1.0985389432055366 |
### Chart
| Category | |
|---|---|
| T1 | 1.0465396167571923 |
| T2 | 1.446445360526506 |
| T3 | 0.8457374830852504 |
| T4 | 0.5116136293870869 |
| T5 | 0.42320876888569137 |
| T6 | 0.1280639295136132 |
| T7 | 0.7483349547257352 |
### Chart
| Category | |
|---|---|
| T1 | 0.7449344457687723 |
| T2 | 0.6461200491051237 |
| T3 | 0.619463544570402 |
| T4 | 0.8290499088045101 |
| T5 | 0.8481764206955046 |
| T6 | 1.0902509757746233 |
| T7 | 1.173392159393591 |Log10 of protein changes
Log10 of protein changes
Log10 of protein changes
Growth Stage
Growth Stage
Growth Stage
### Chart: GAPDH
| Category | | |
|---|---|---|
| T1 | 1.0692349326626418 | 0.9581441041488457 |
| Log | 1.2685540124595818 | 1.021115093985471 |
| T2 | 1.0947 | 0.9915750000000001 |
| ES | 1.0 | 1.0 |
| LS | 1.1647500000000026 | 0.8480050000000015 |Log10 of protein changes
Growth stage
55
kDa
GAPDH
35
T1 T2 T3 T4 T5
Growth stage
T1 T2 T3 T4 T5
### Chart: OmpX
| Category | | |
|---|---|---|
| T1 | 0.5464468719503903 | 0.47856049394258293 |
| Log | 0.5978782869468061 | 0.9770284691204829 |
| T2 | 0.8129450000000018 | 0.8387400000000018 |
| ES | 1.0 | 1.0 |
| LS | 1.026899999999997 | 1.2325999999999973 |Log10 of protein changes
Growth stage
25
kDa
OmpX
15
Growth stage
T1 T2 T3 T4 T5
T1 T2 T3 T4 T5
### Chart: MBP
| Category | | |
|---|---|---|
| T1 | 7.206569459931951 | 3.1423378140502285 |
| Log | 4.811166115331483 | 4.163401033193857 |
| T2 | 1.55975 | 0.9198250000000014 |
| ES | 1.0 | 1.0 |
| LS | 0.6097150000000015 | 0.9758400000000012 |Log10 of protein changes
Growth stage
55
MBP
kDa
35
Growth stage
T1 T2 T3 T4 T5
T1 T2 T3 T4 T5
D.

## Slide 14
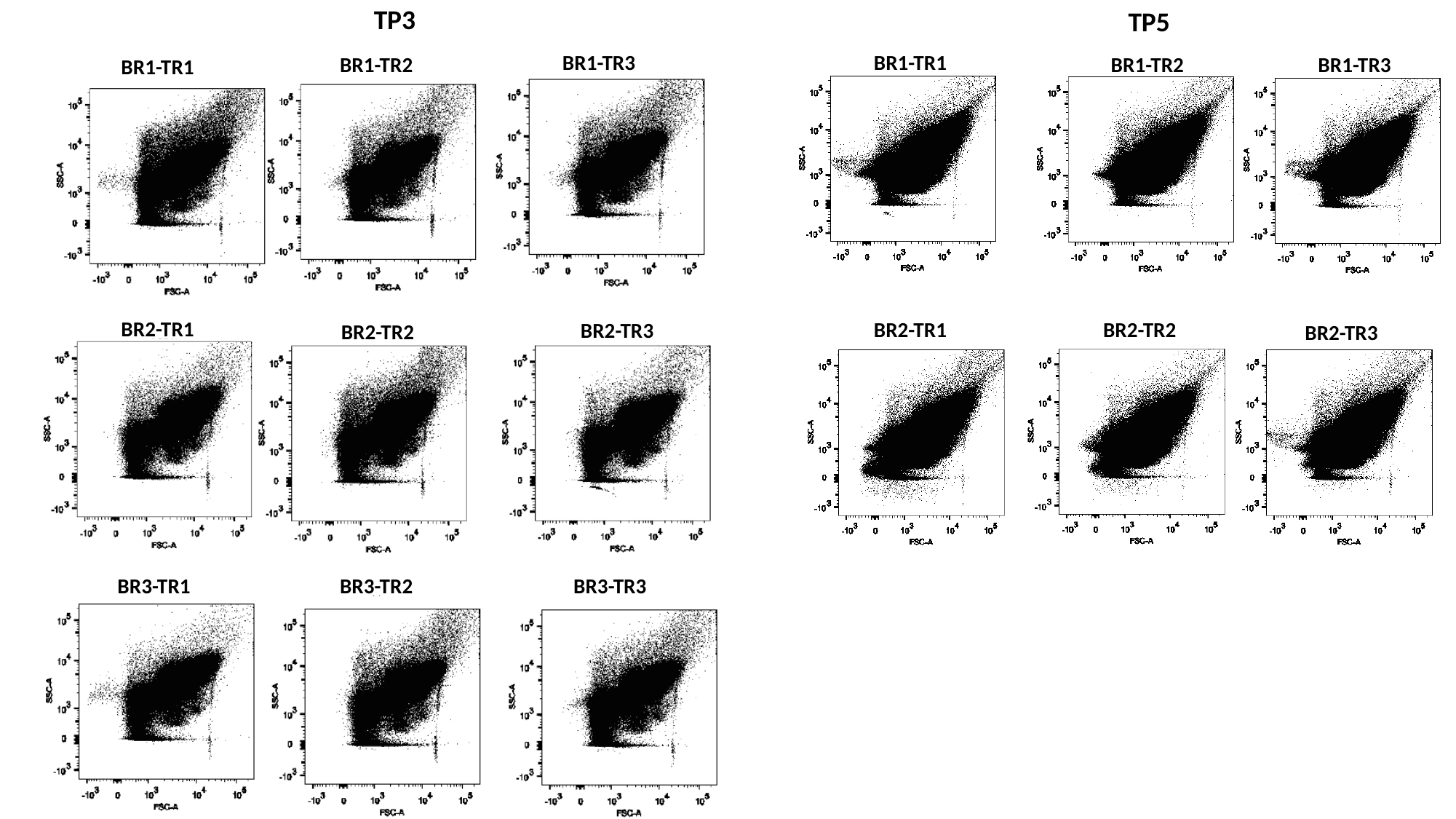

TP3
TP5
BR1-TR3
BR1-TR1
BR1-TR3
BR1-TR2
BR1-TR2
BR1-TR1
BR2-TR1
BR2-TR2
BR2-TR1
BR2-TR3
BR2-TR2
BR2-TR3
BR3-TR1
BR3-TR2
BR3-TR3
