# Supplementary material for: Characterization of the E. coli proteome and its modifications during growth and ethanol stress
Source: Front Microbiol. 2015 Feb 18;6:103. doi: 10.3389/fmicb.2015.00103 (PMC4332353; doi:10.3389/fmicb.2015.00103)
Supplement: Supplementary file 1 [file DataSheet1.ZIP › Supplementary File 1- protein complex stoichiometries.pdf]

Please note the following

Lag = TP1

FT = TP2

Log= TP3

ST= TP4

ES= TP5

LSA= TP6

LSB = TP7

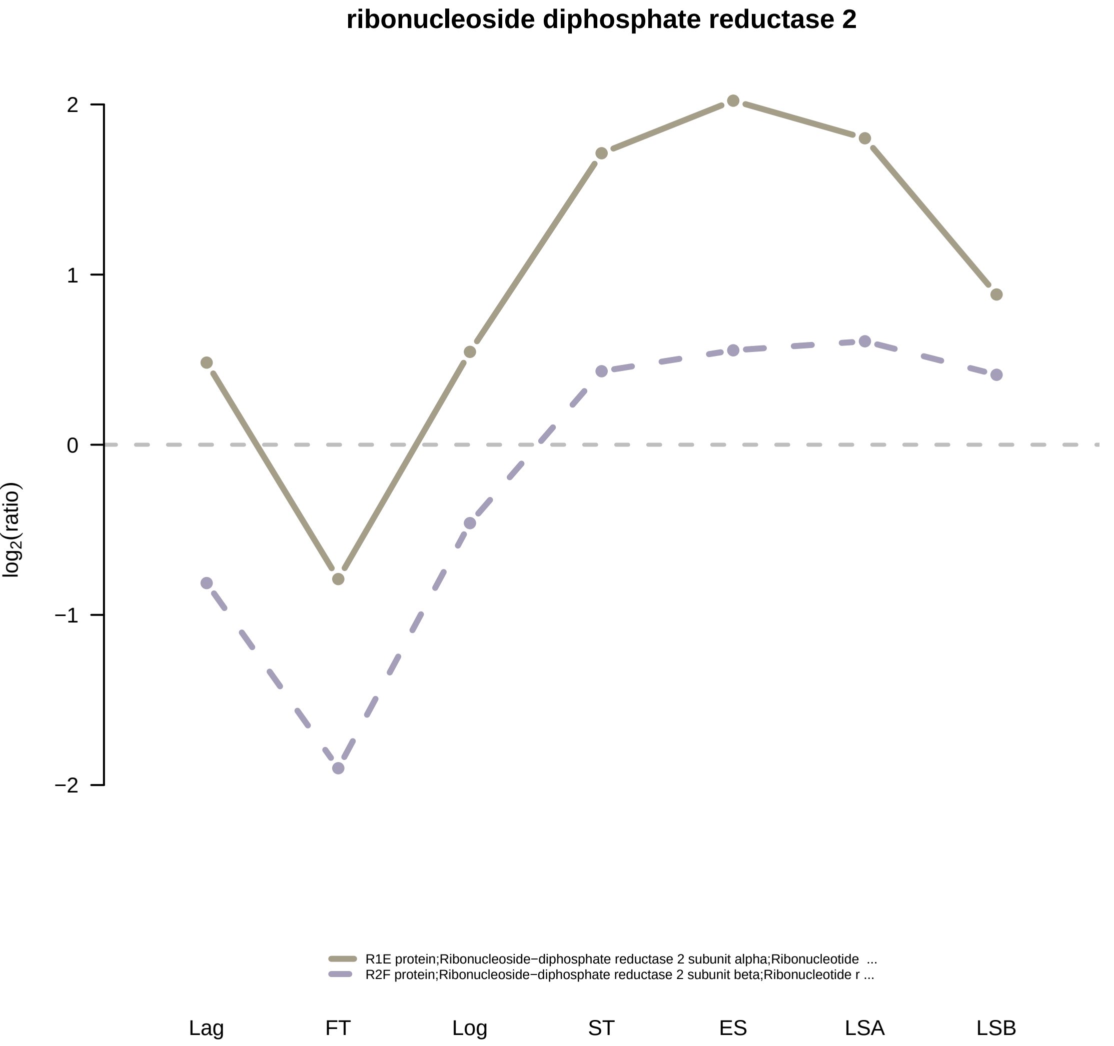

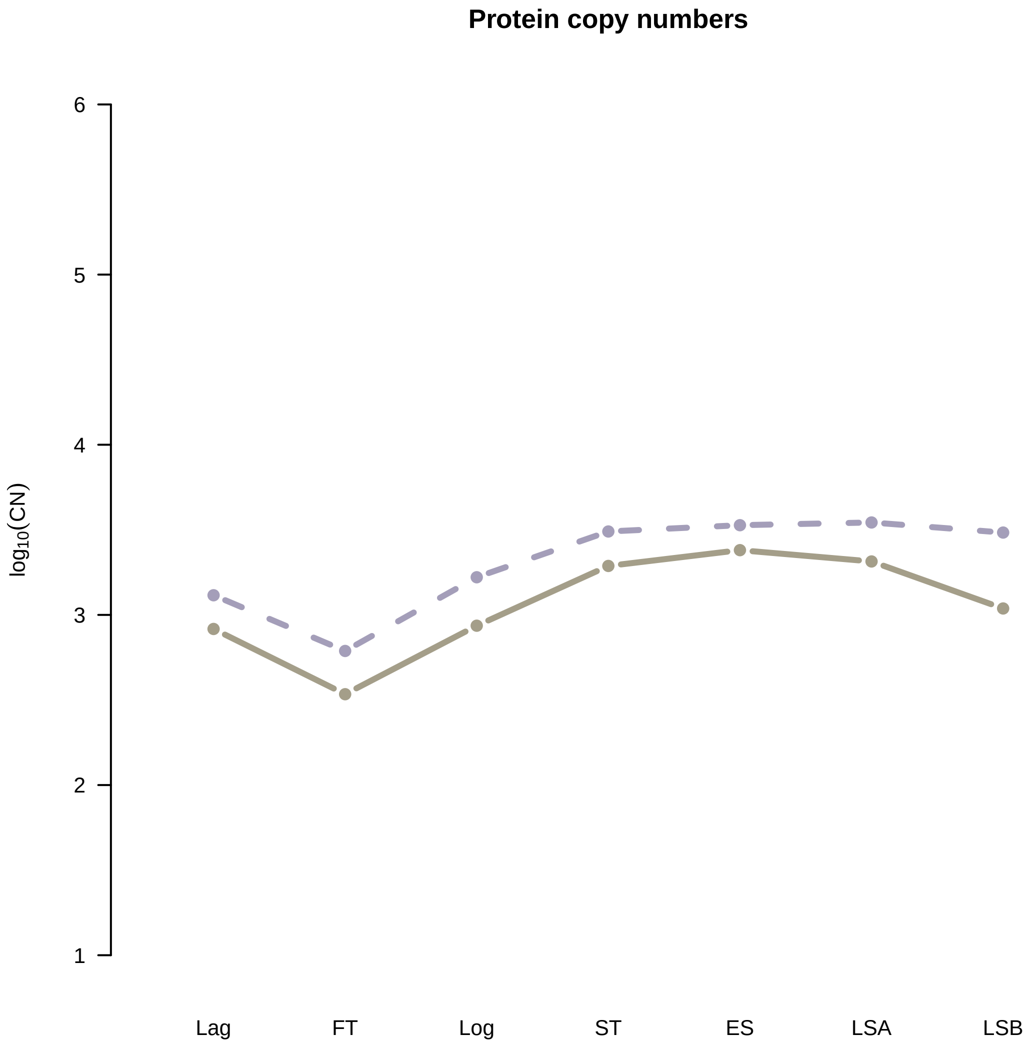

nrdE	P39452	R1E protein;Ribonucleoside−dip ...
nrdF	P37146	R2F protein;Ribonucleoside−dip ...

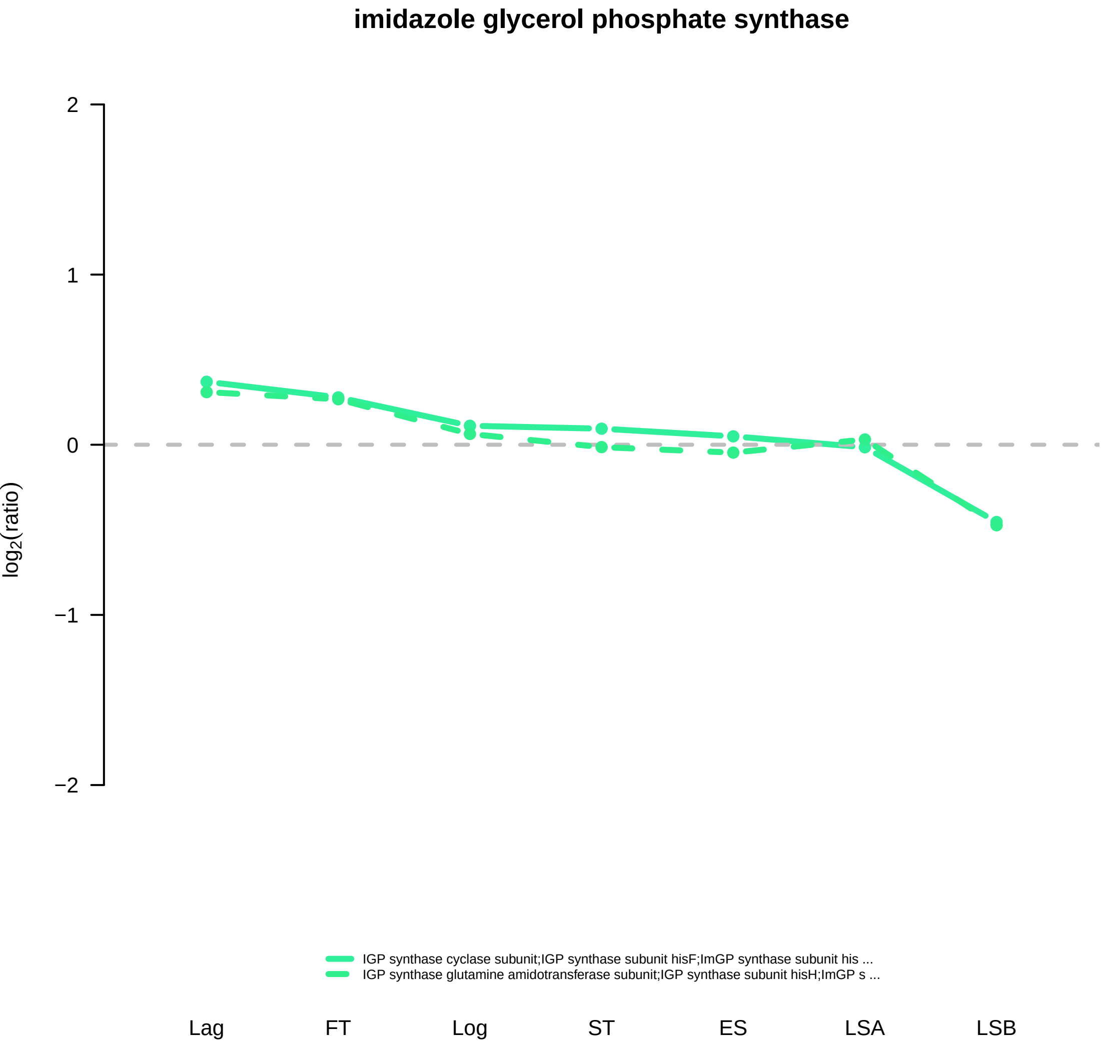

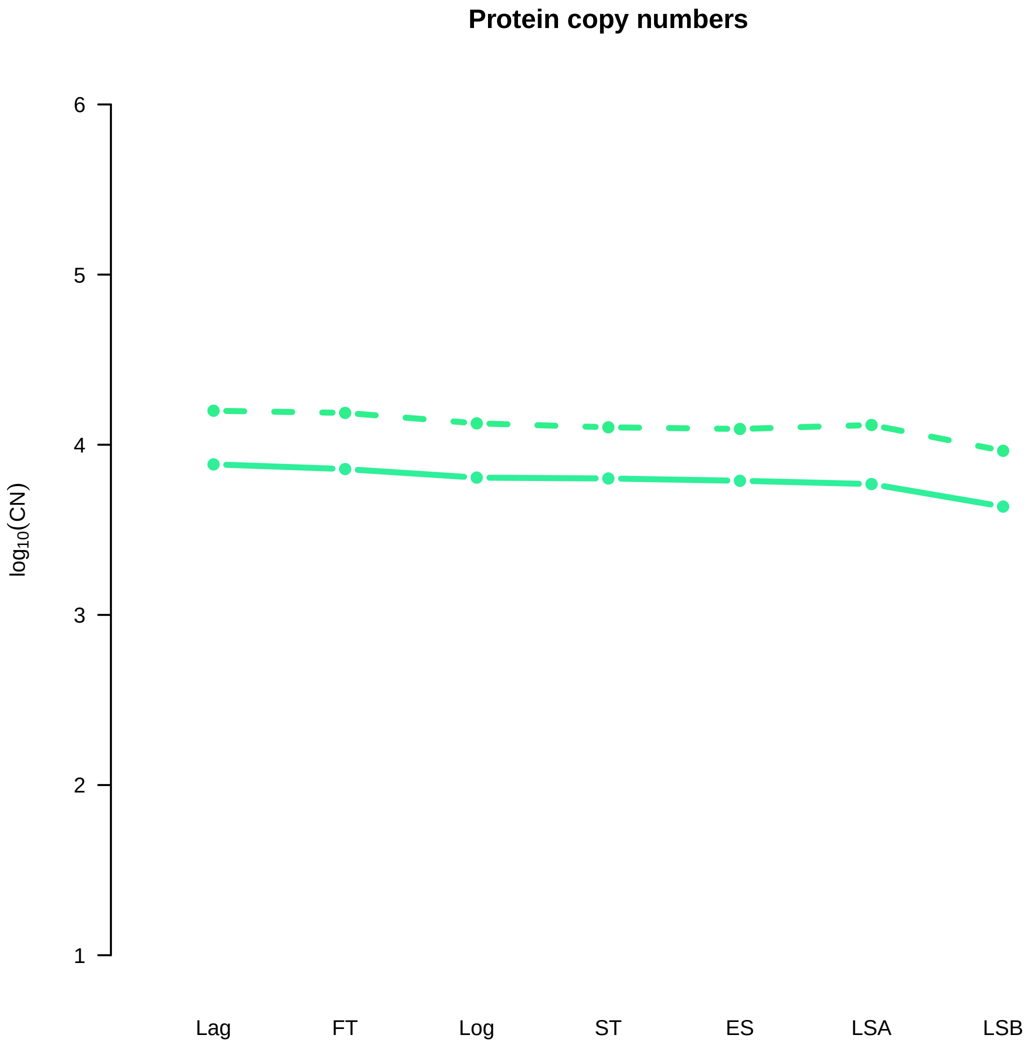

hisF	P60664	IGP synthase cyclase subunit;I ...
hisH	P60595	IGP synthase glutamine amidotr ...

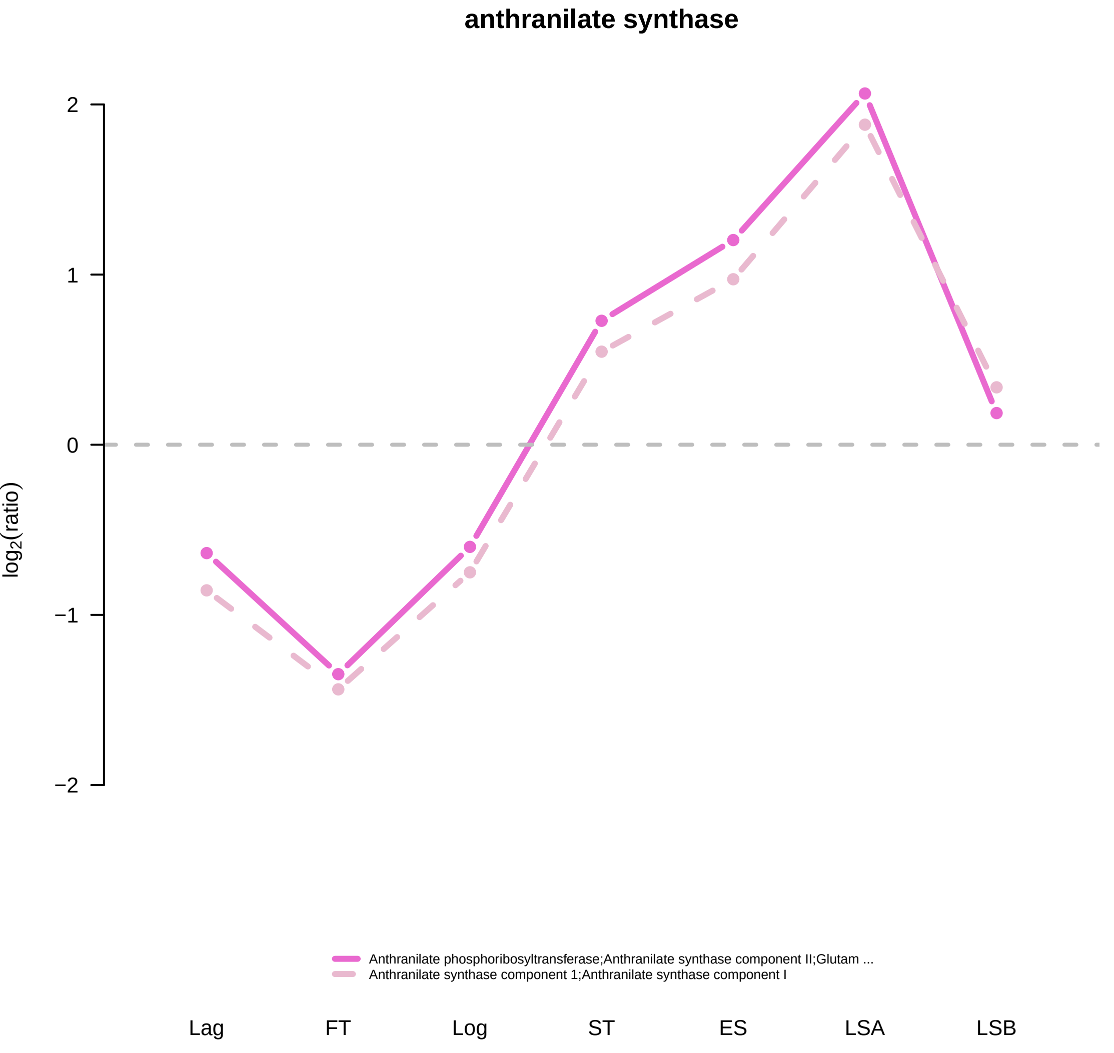

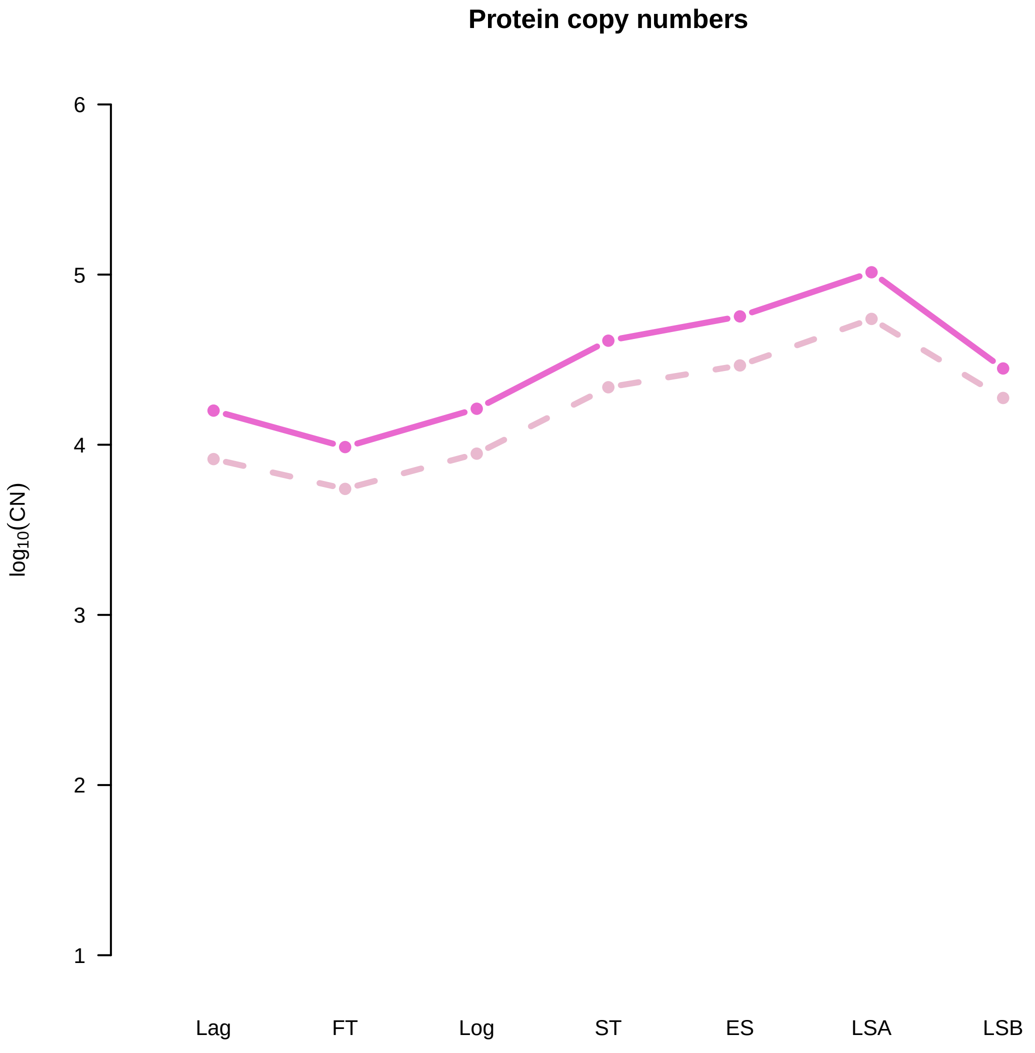

trpD	P00904	Anthranilate phosphoribosyltra ...
trpE	P00895	Anthranilate synthase componen ...

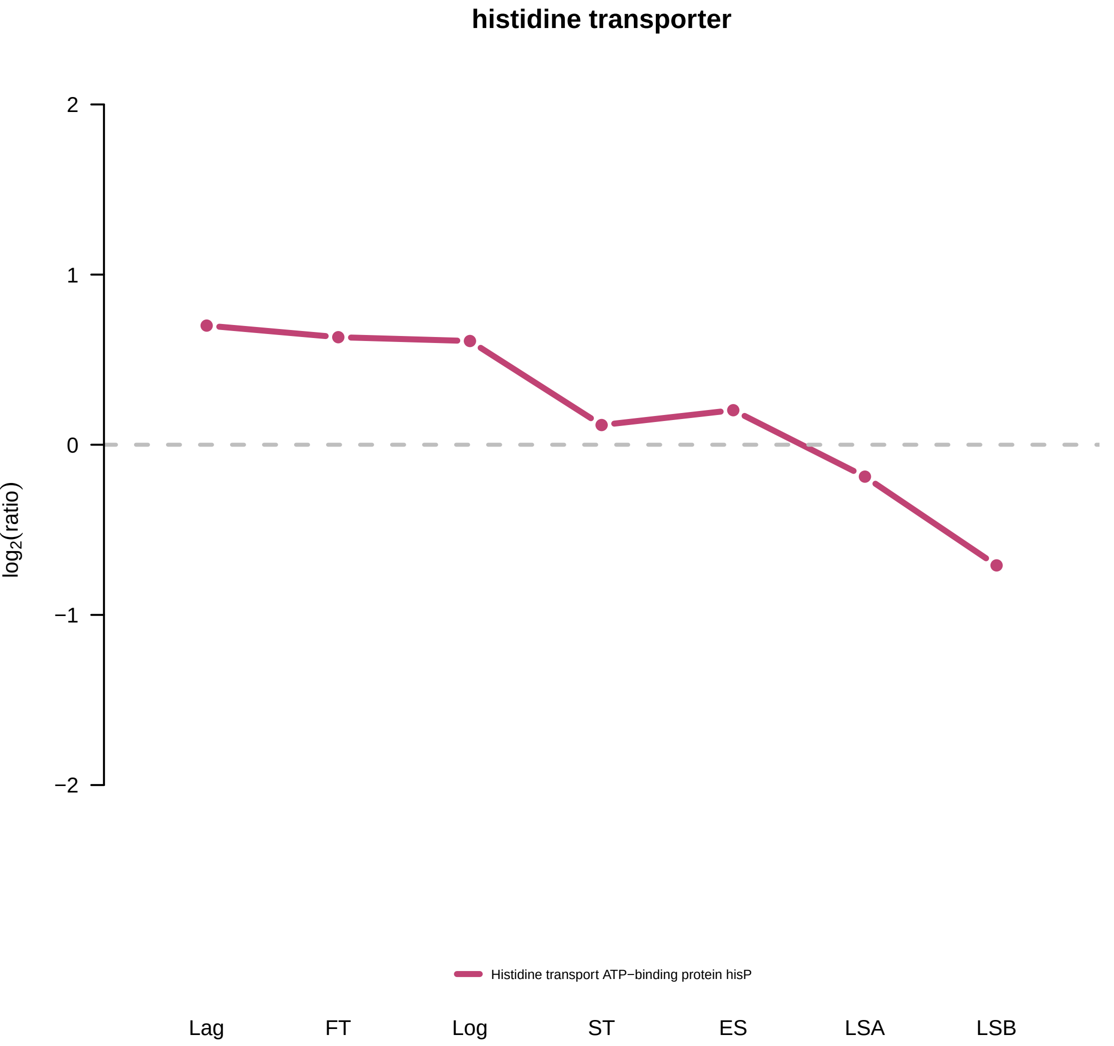

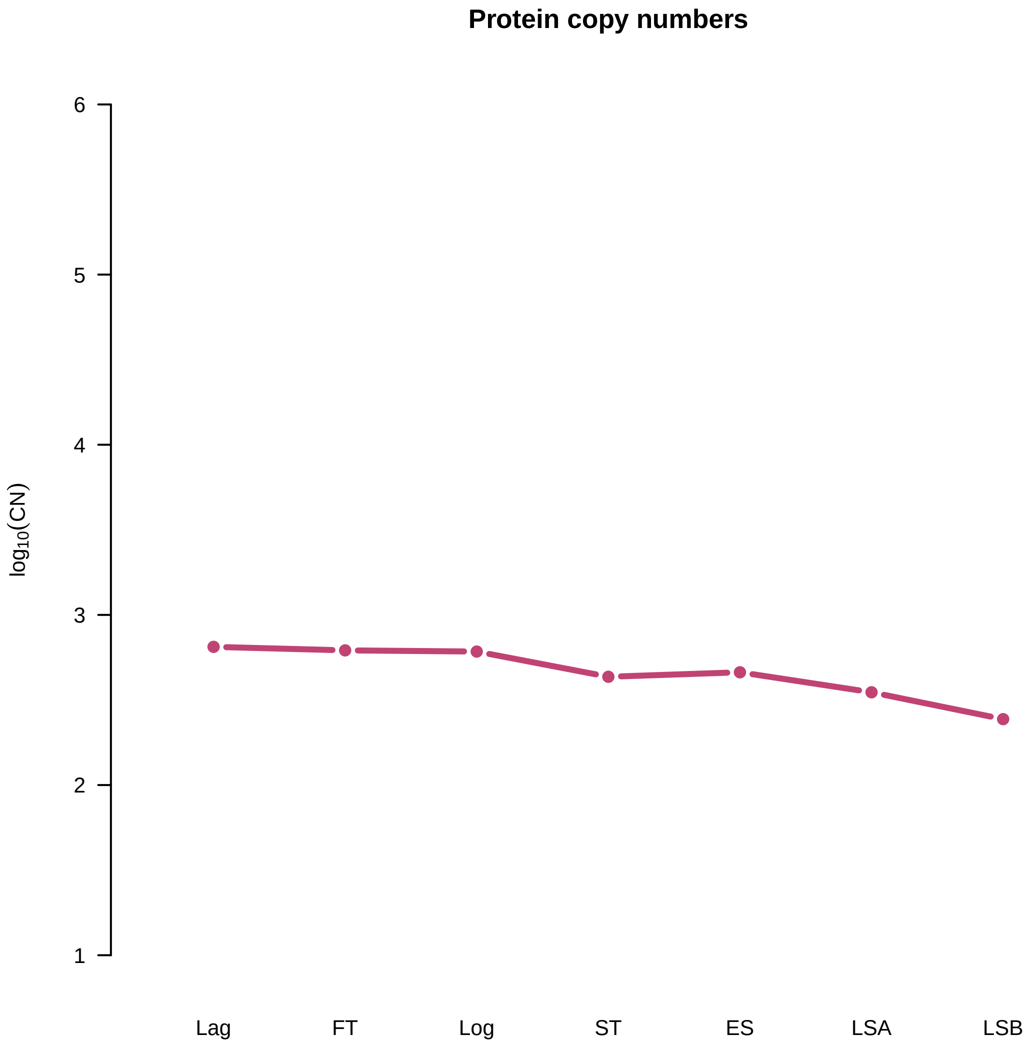

hisM	−	−
hisP	P07109	Histidine transport ATP−bindin ...
hisQ	P52094	Histidine transport system per ...

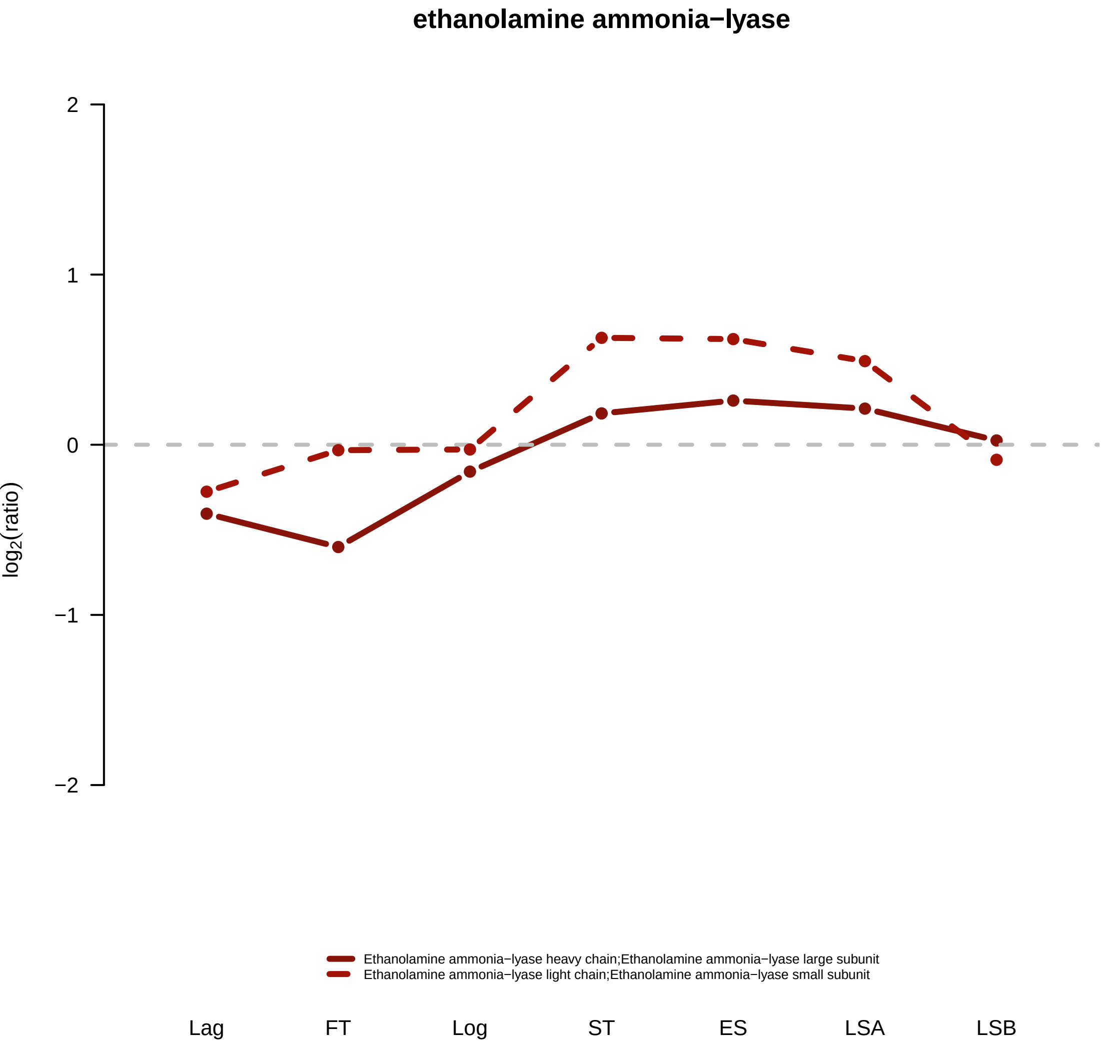

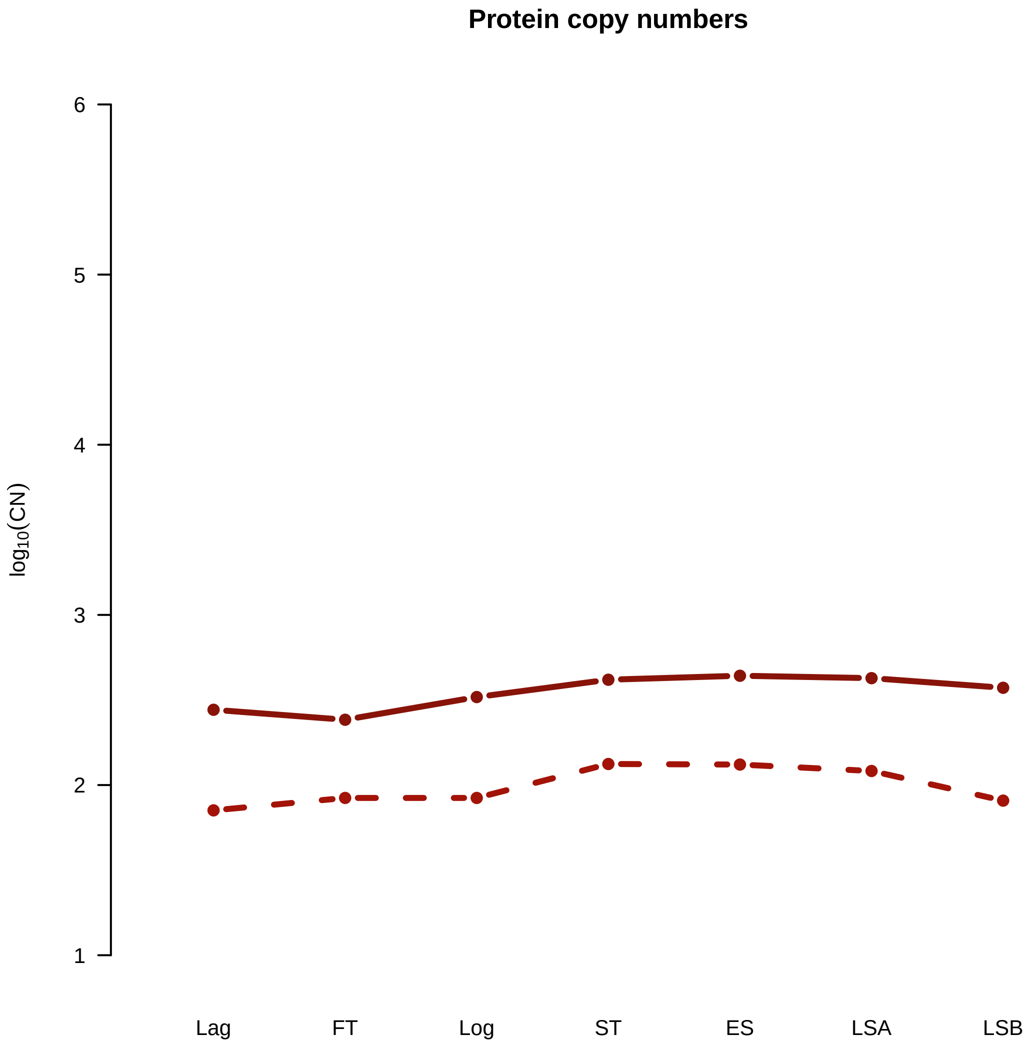

eutB	P0AEJ6	Ethanolamine ammonia−lyase hea ...
eutC	P19636	Ethanolamine ammonia−lyase lig ...

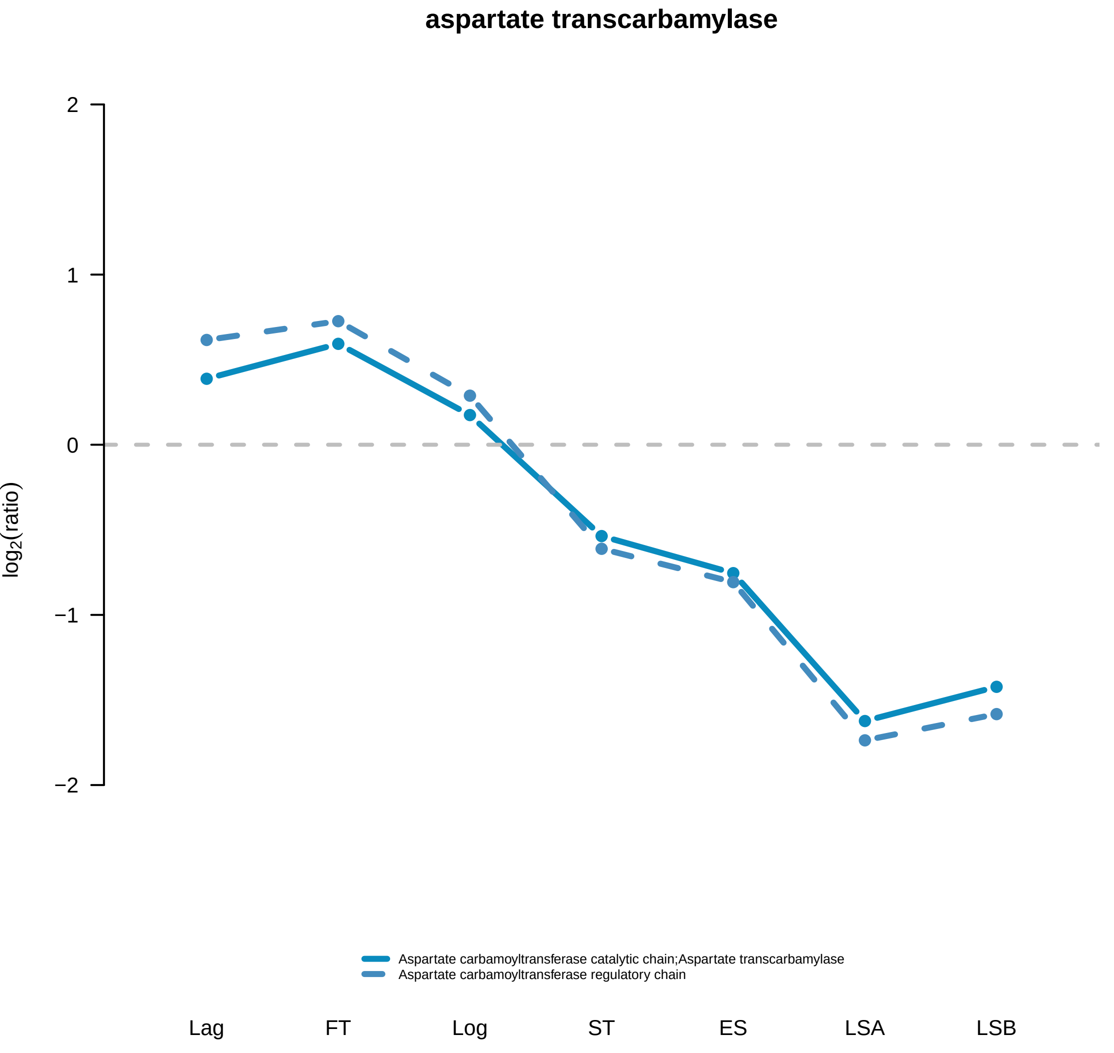

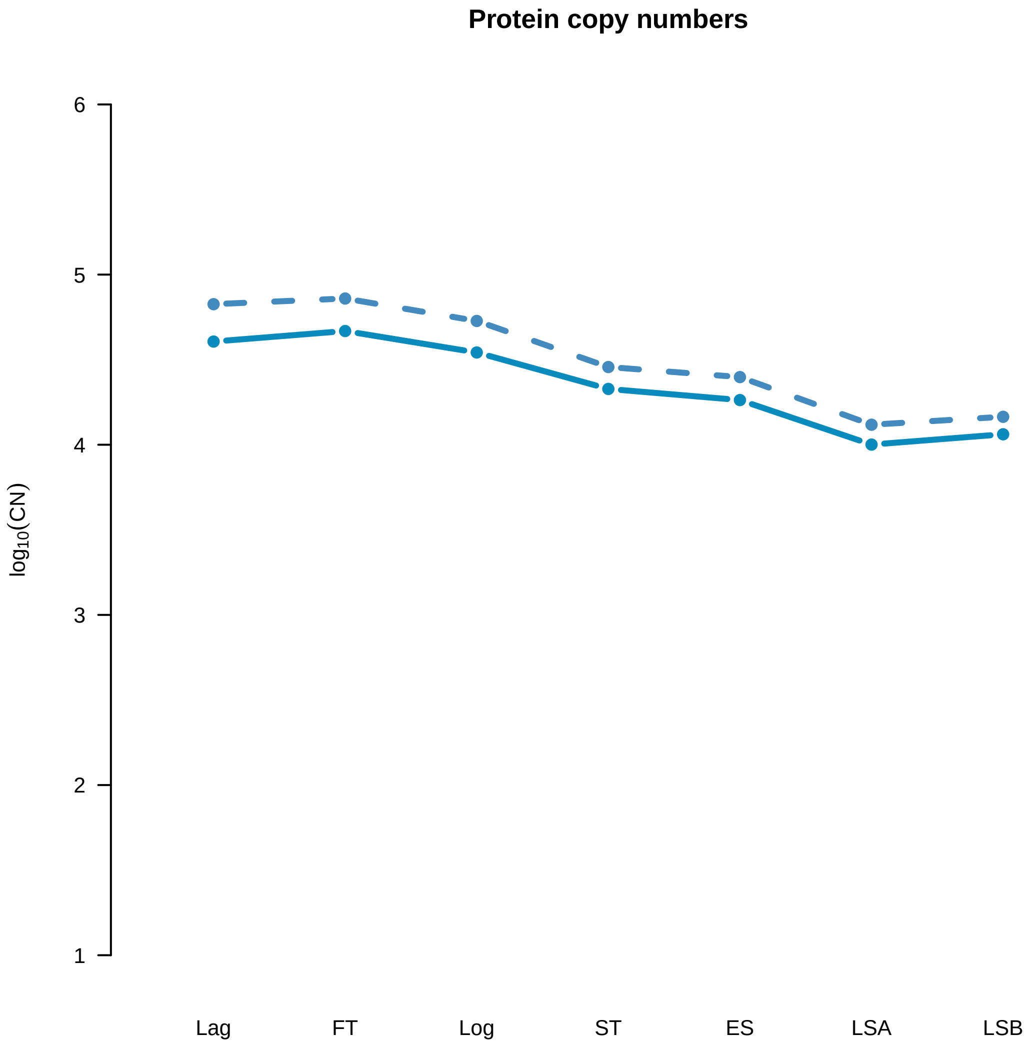

pyrB	P0A786	Aspartate carbamoyltransferase ...
pyrI	P0A7F3	Aspartate carbamoyltransferase ...

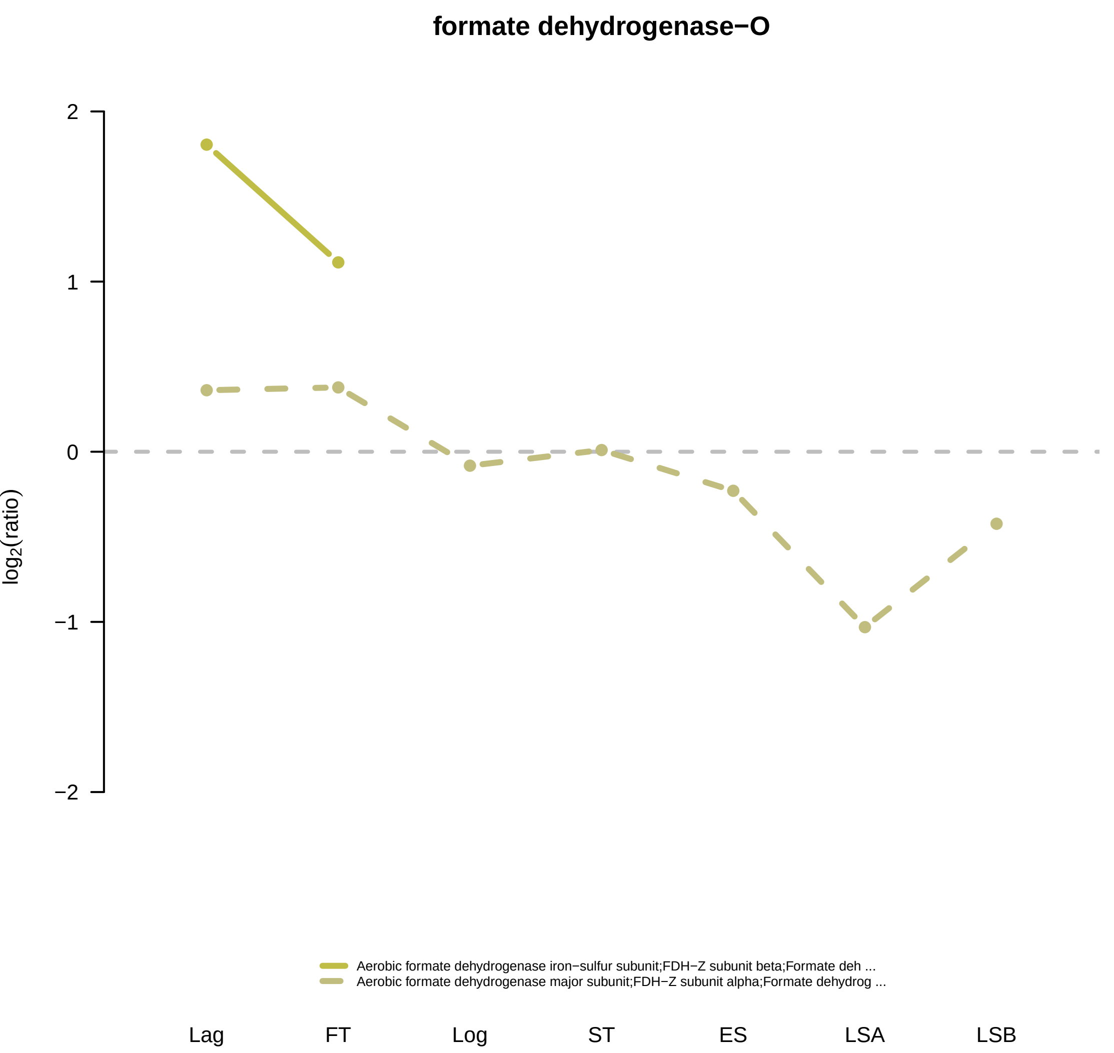

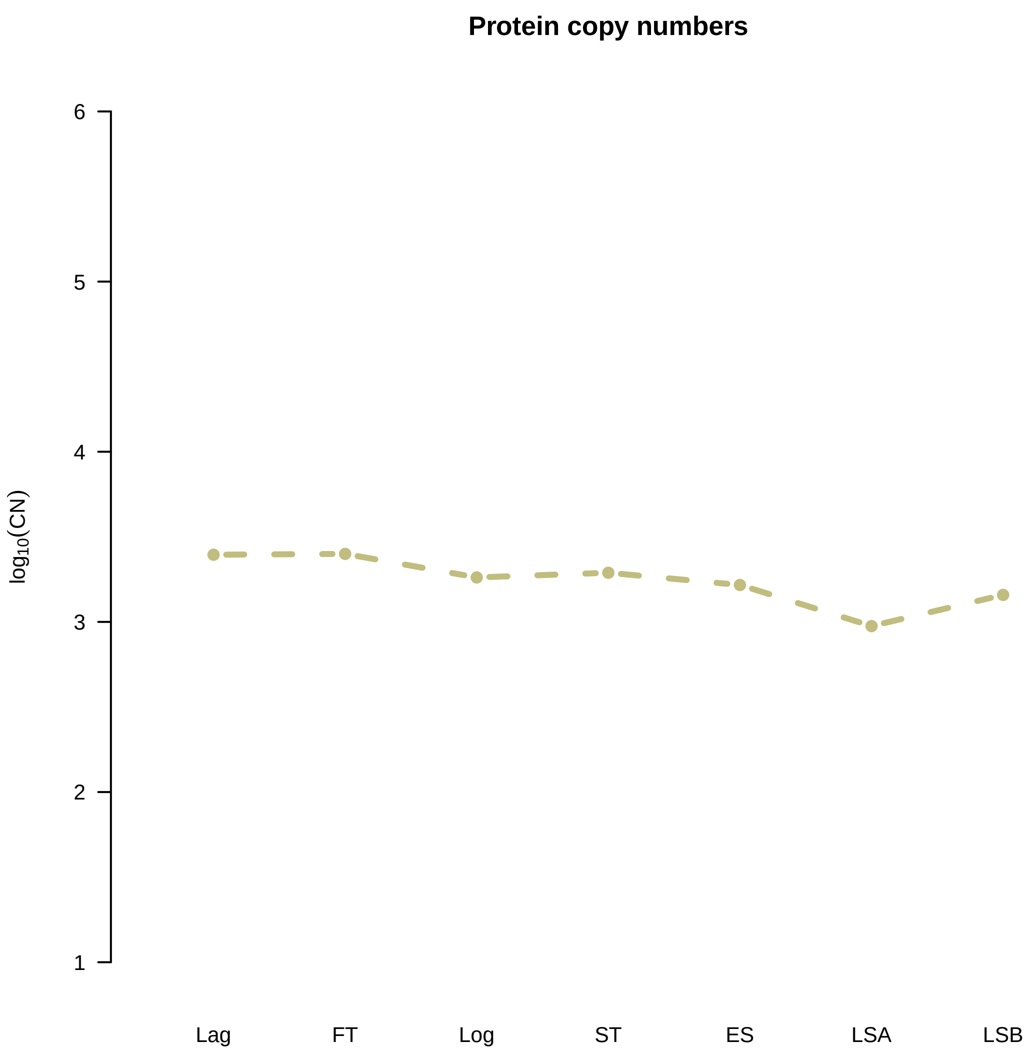

fdoI	−		−
fdoH	P0AAJ5	Aerobic formate dehydrogenase	...
fdoG	P32176	Aerobic formate dehydrogenase	...

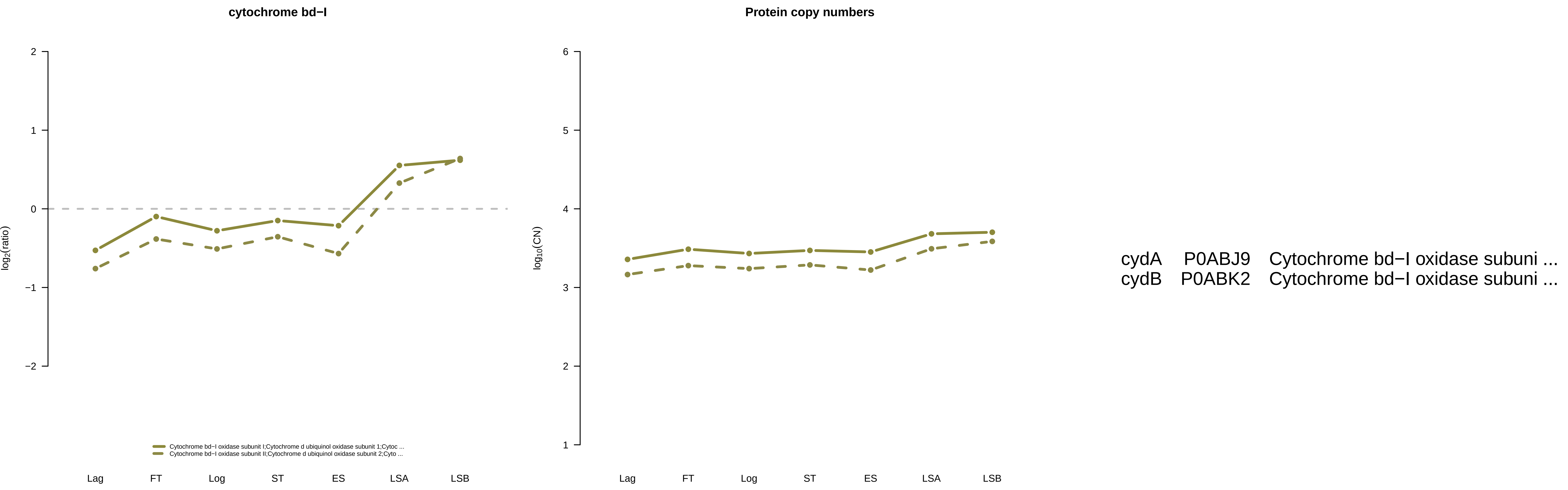
cytochrome bd−I
Protein copy numbers
2
1
0
−1
−2
log2(ratio)
6
5
4
3
2
1
log10(CN)
Cytochrome bd−I oxidase subunit I;Cytochrome d ubiquinol oxidase subunit 1;Cytoc ...
Cytochrome bd−I oxidase subunit II;Cytochrome d ubiquinol oxidase subunit 2;Cyto ...
Lag FT Log ST ES LSA LSB
Lag FT Log ST ES LSA LSB
cydA P0ABJ9 Cytochrome bd−I oxidase subuni ...
cydB P0ABK2 Cytochrome bd−I oxidase subuni ...

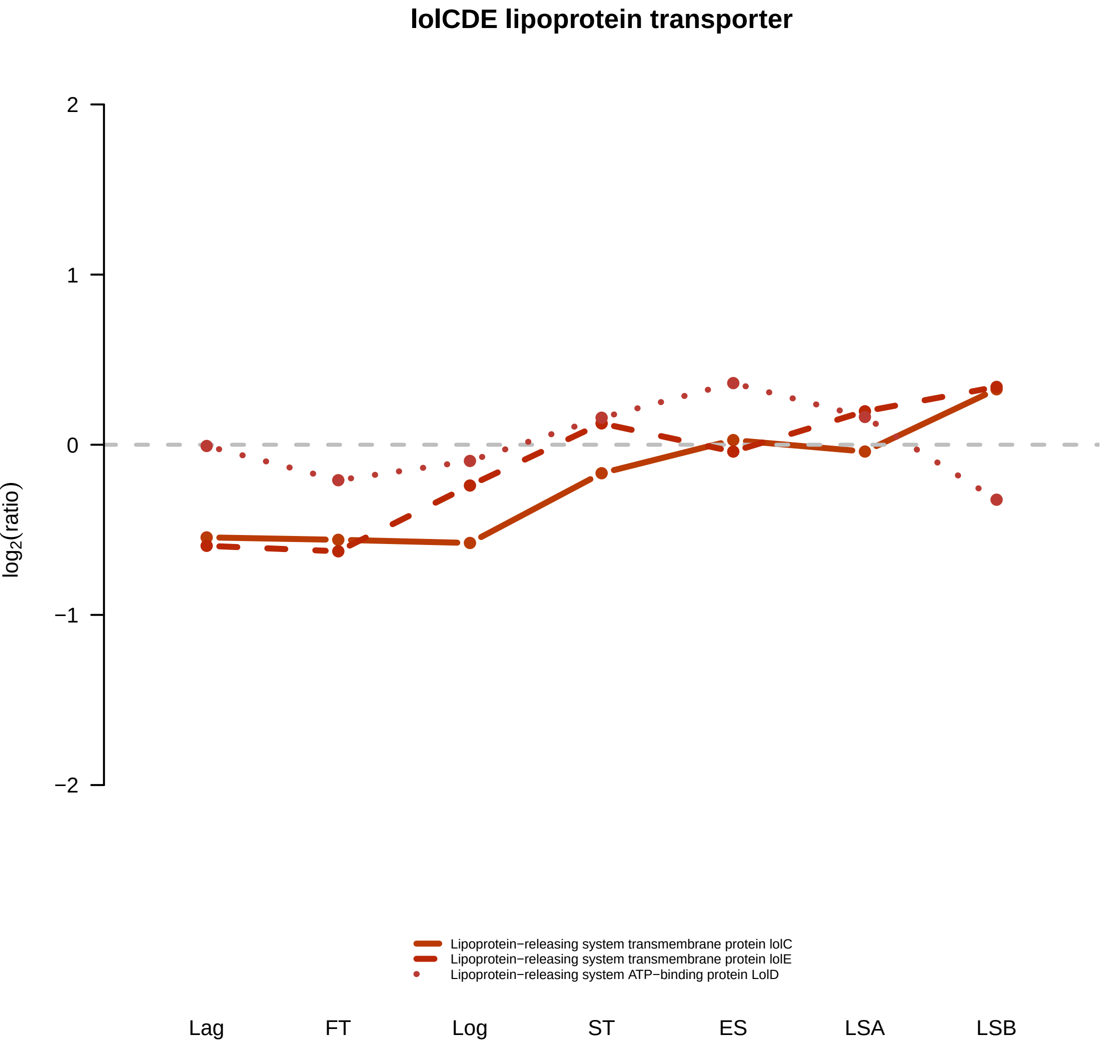

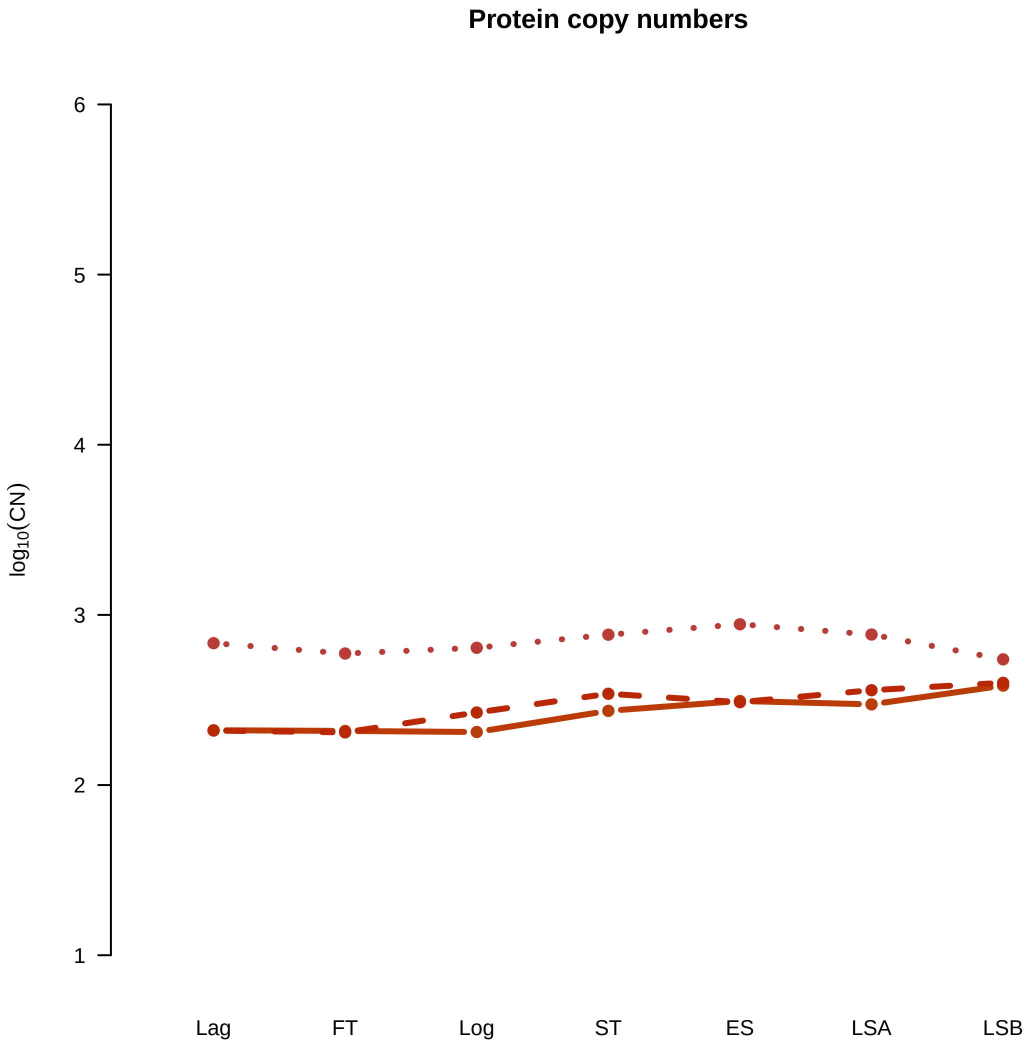

lolC	P0ADC3	Lipoprotein−releasing system t ...
lolE	P75958	Lipoprotein−releasing system t ...
lolD	P75957	Lipoprotein−releasing system A ...

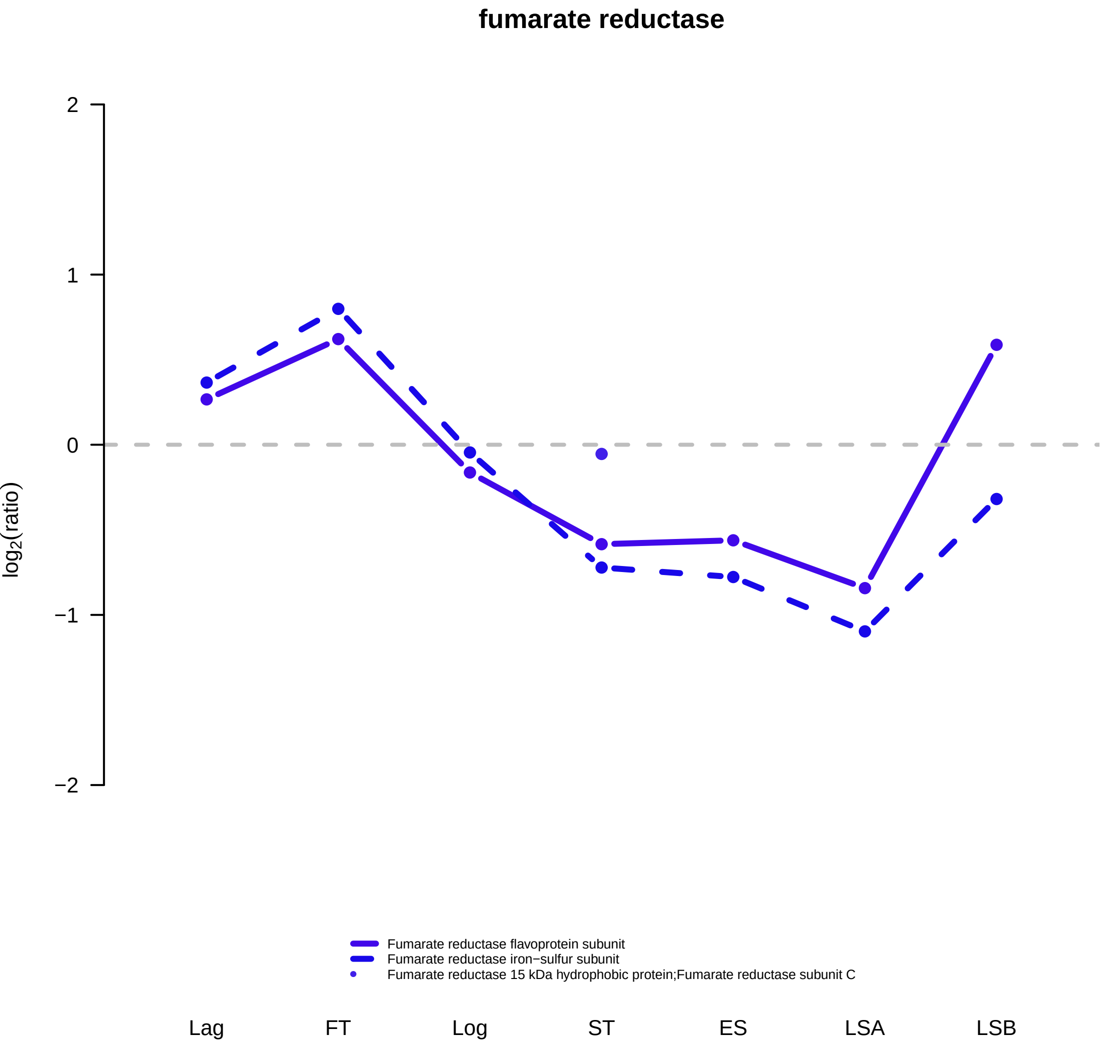

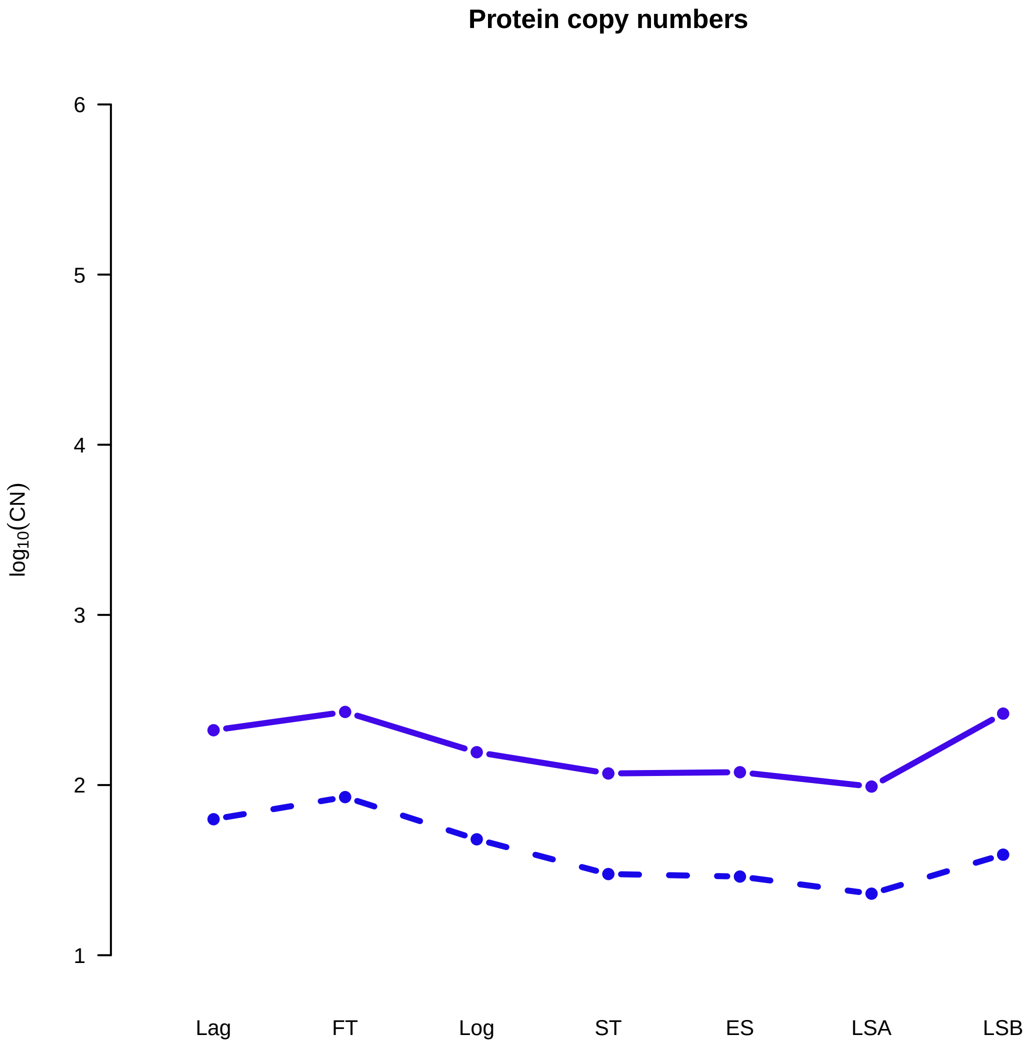

frdA	P00363	Fumarate reductase flavoprotei ...
frdB	P0AC47	Fumarate reductase iron−sulfur ...
frdC	P0A8Q0	Fumarate reductase 15 kDa hydr ...
frdD	−	−

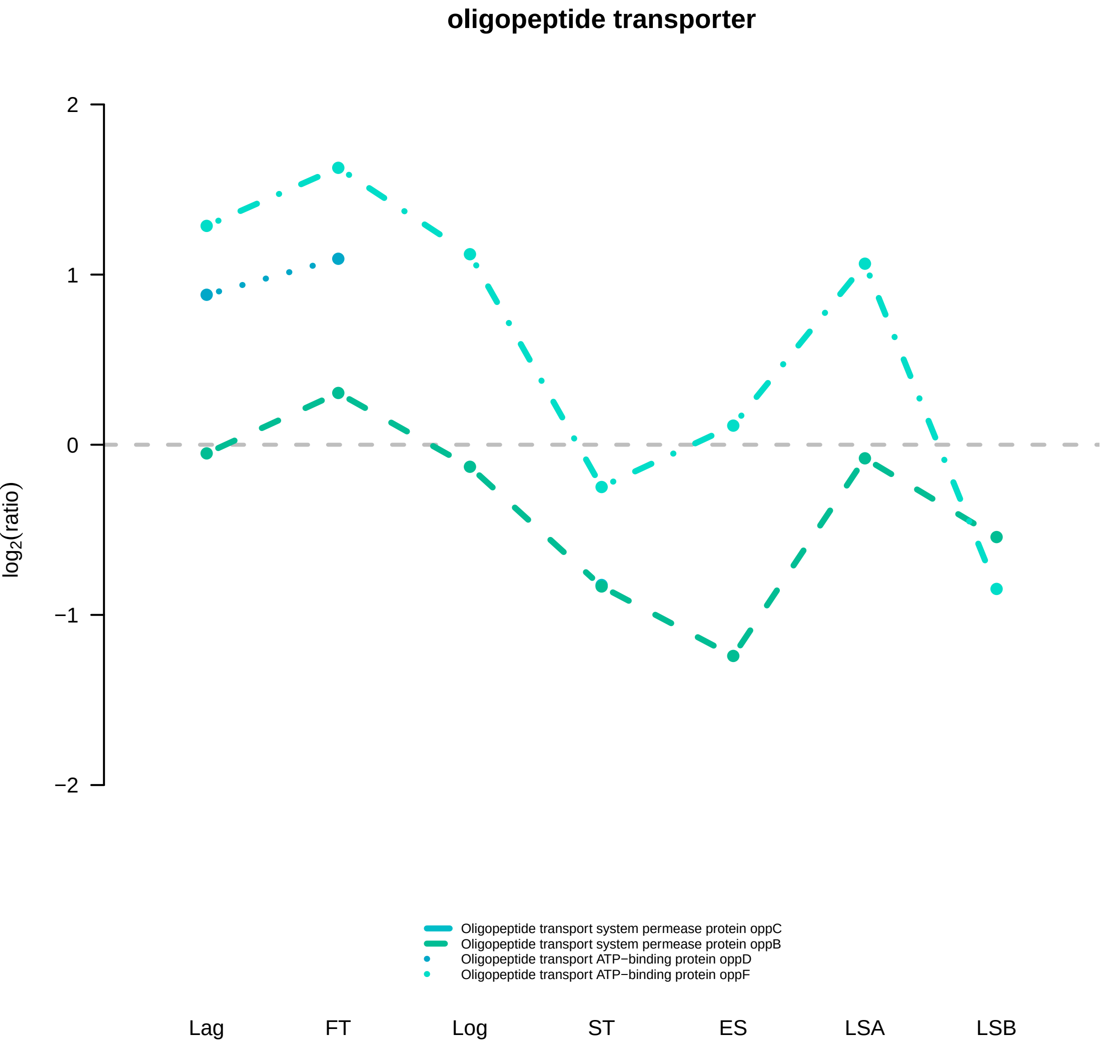

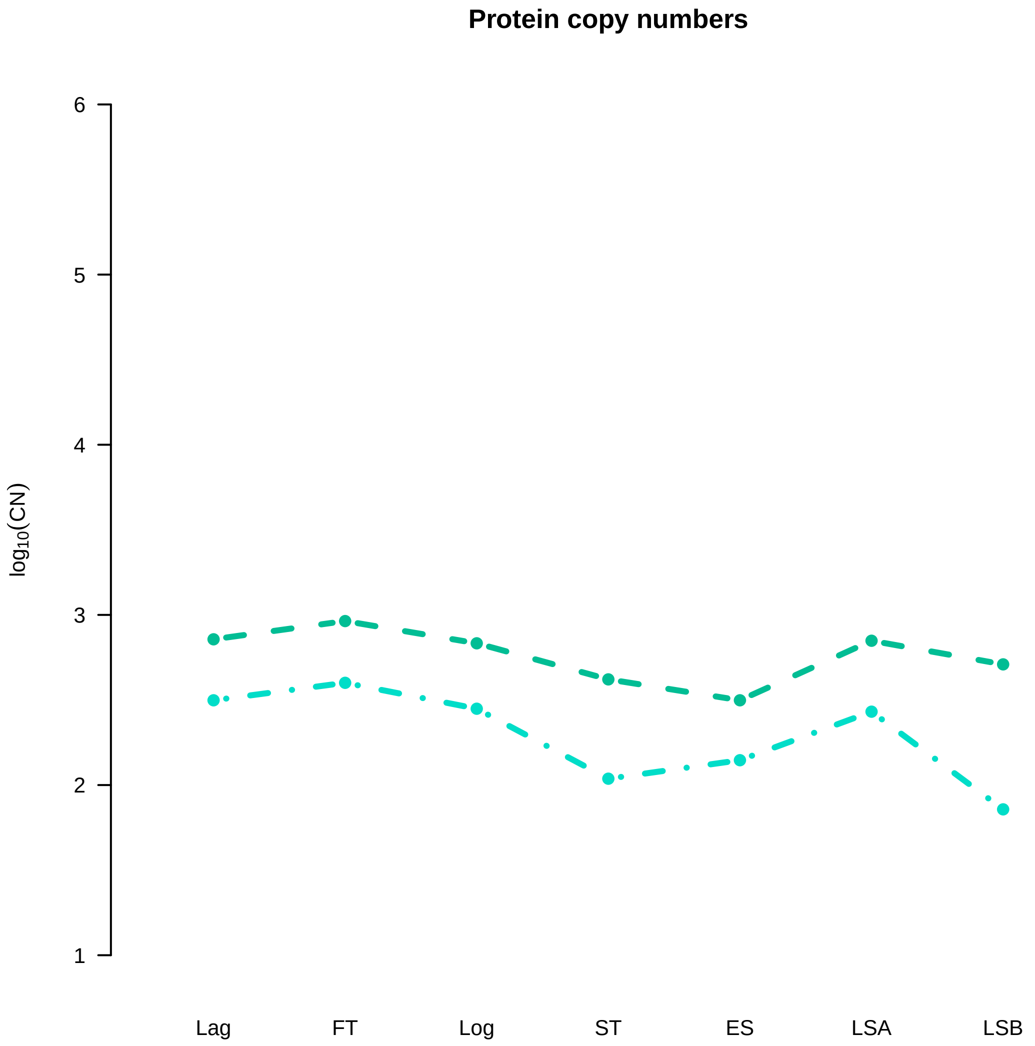

oppC	P0AFH6	Oligopeptide transport system ...
oppB	P0AFH2	Oligopeptide transport system ...
oppD	P76027	Oligopeptide transport ATP−bin ...
oppF	P77737	Oligopeptide transport ATP−bin ...

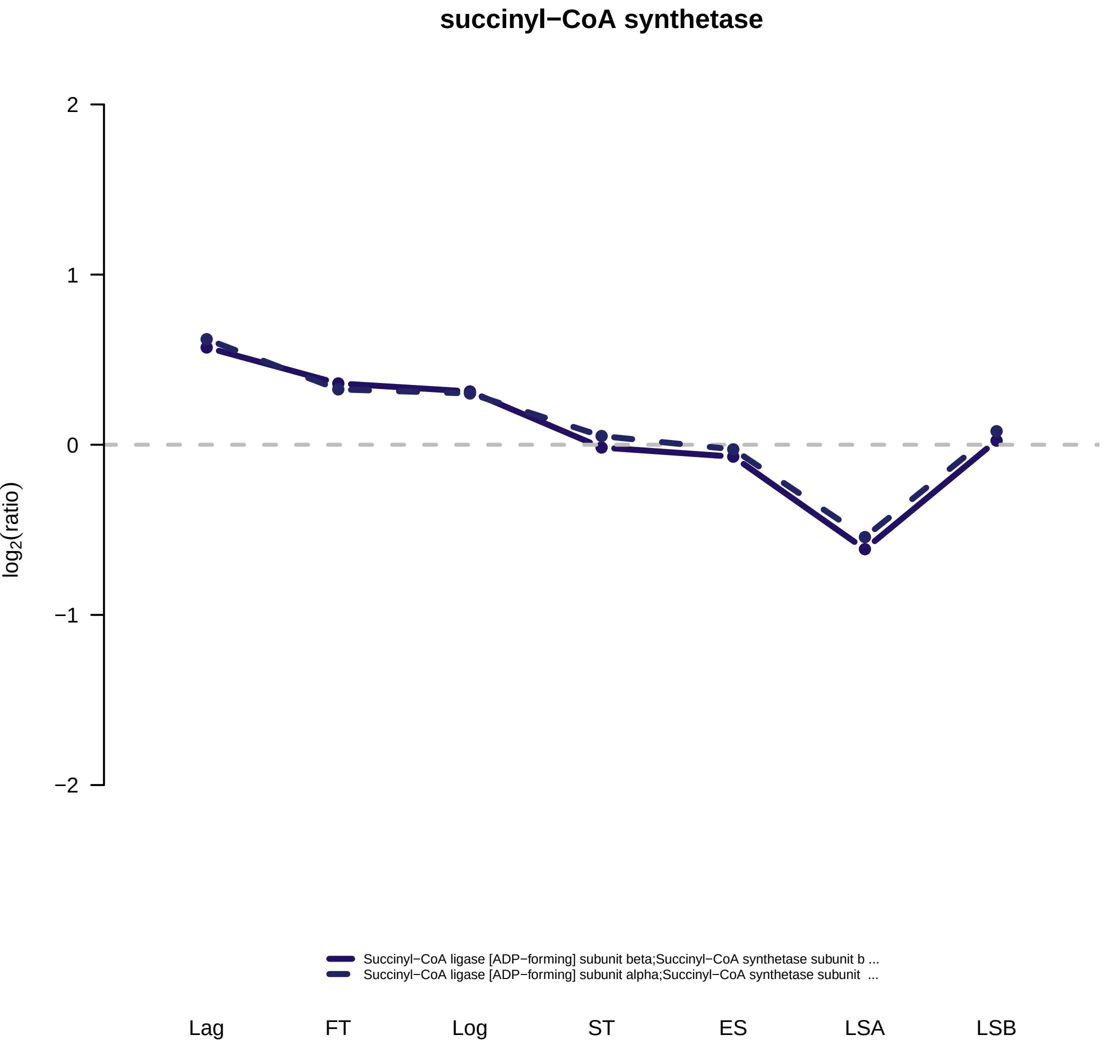

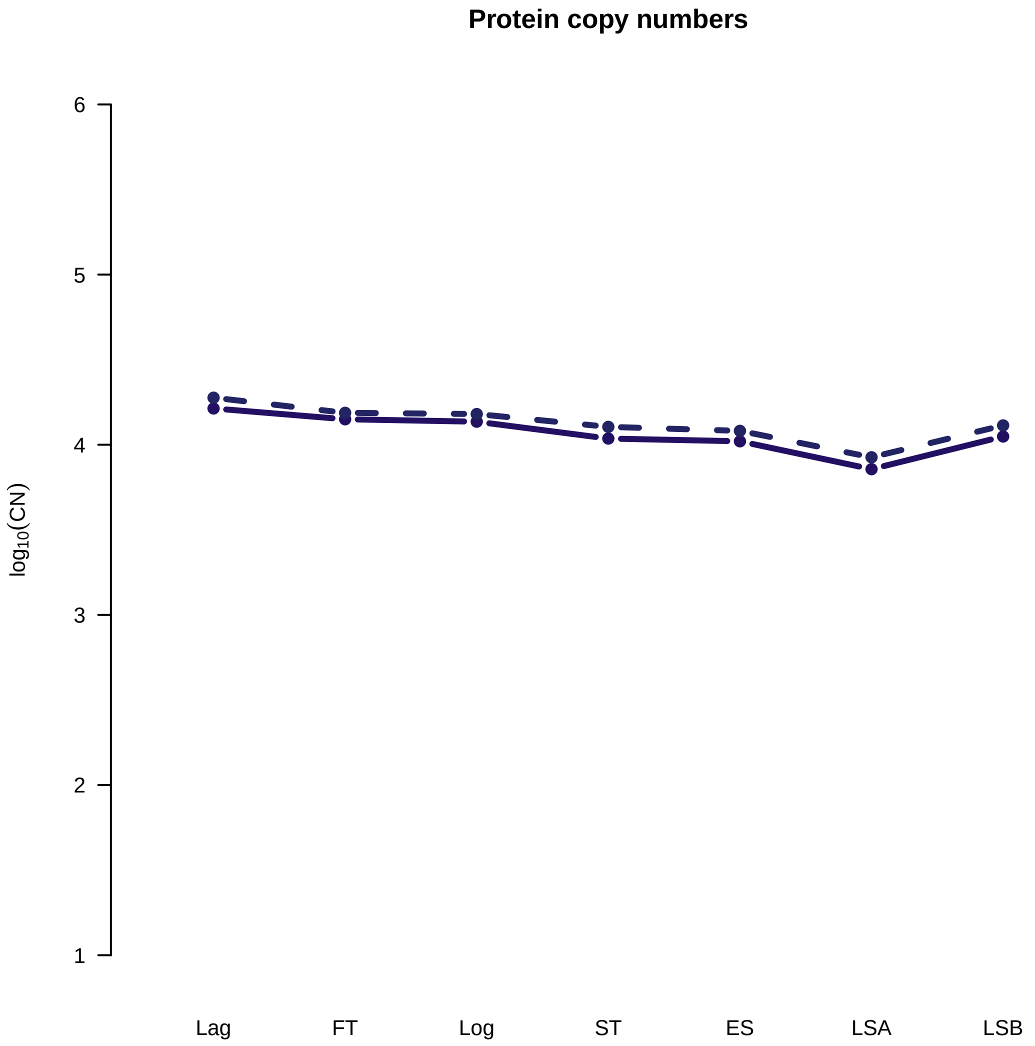

sucC	P0A836	Succinyl−CoA ligase [ADP−formi ...
sucD	P0AGE9	Succinyl−CoA ligase [ADP−formi ...

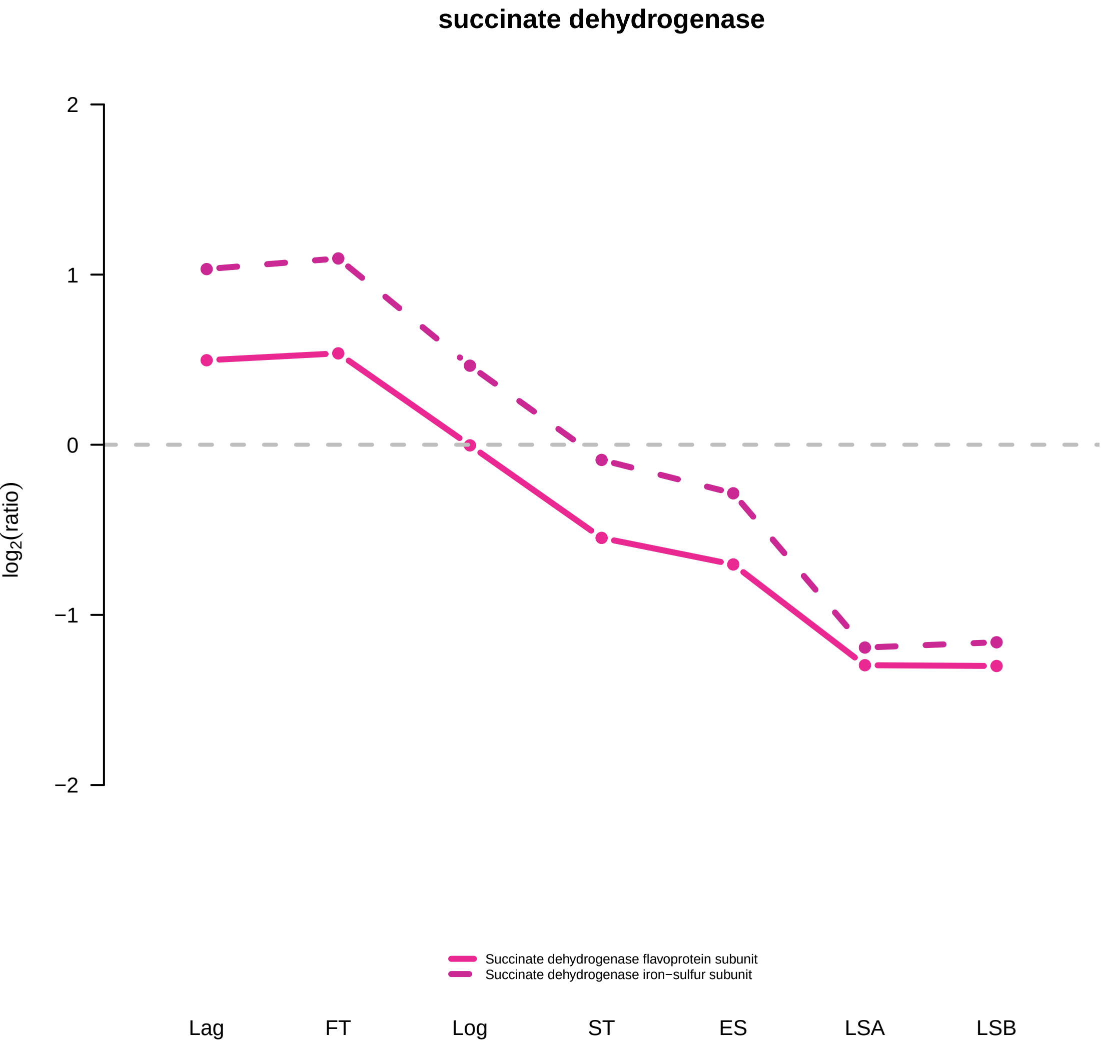

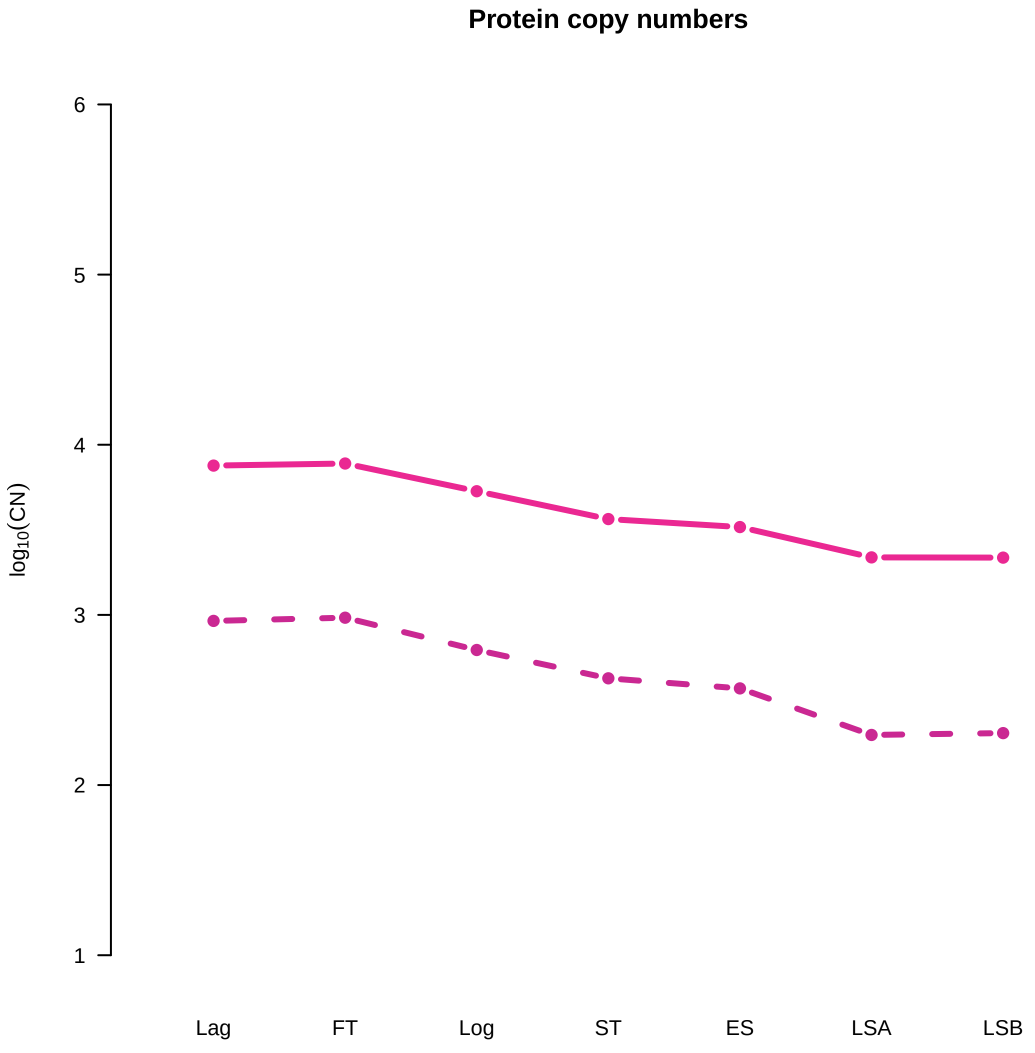

sdhA	P0AC41	Succinate dehydrogenase flavop ...
sdhC	−	−
sdhB	P07014	Succinate dehydrogenase iron−s ...
sdhD	−	−

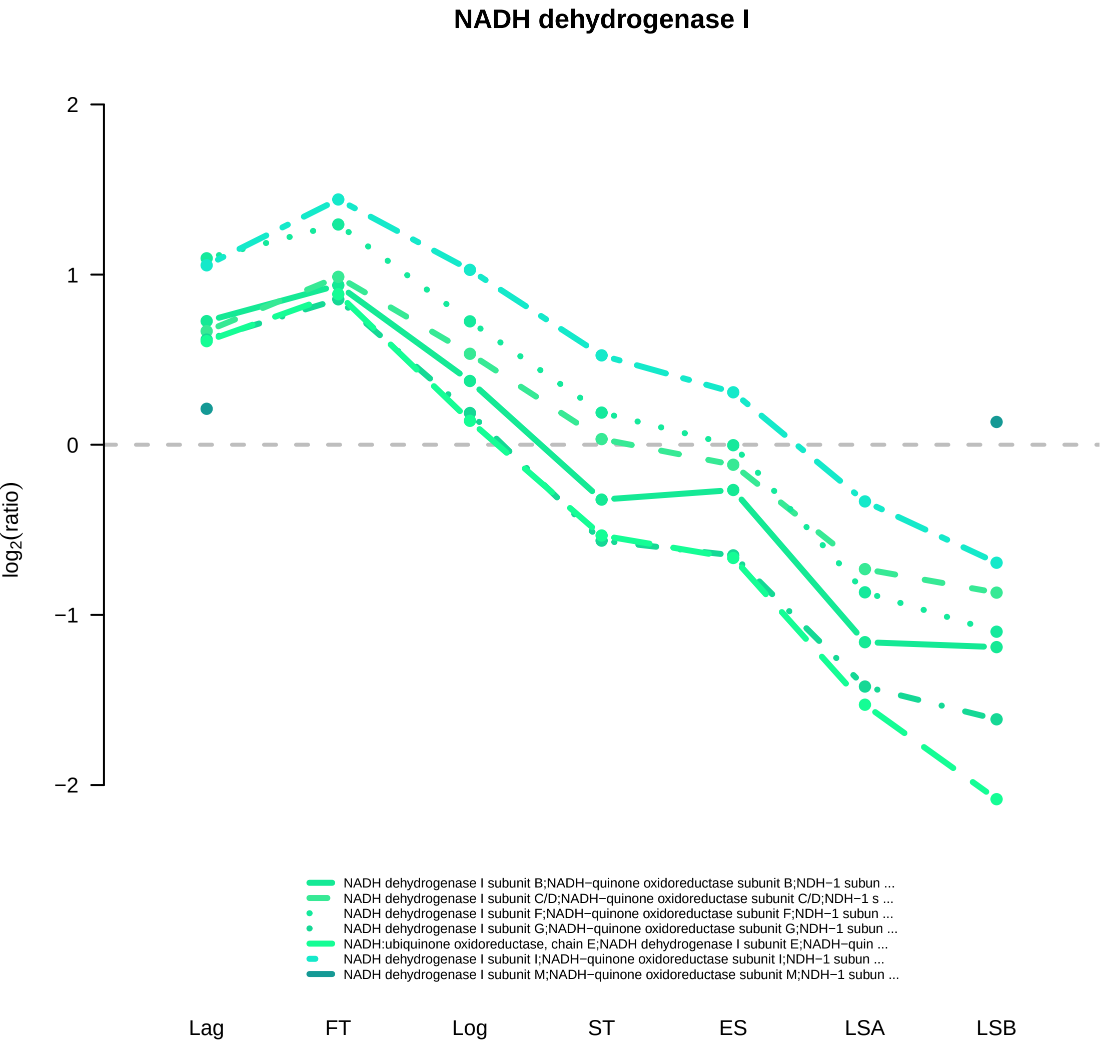

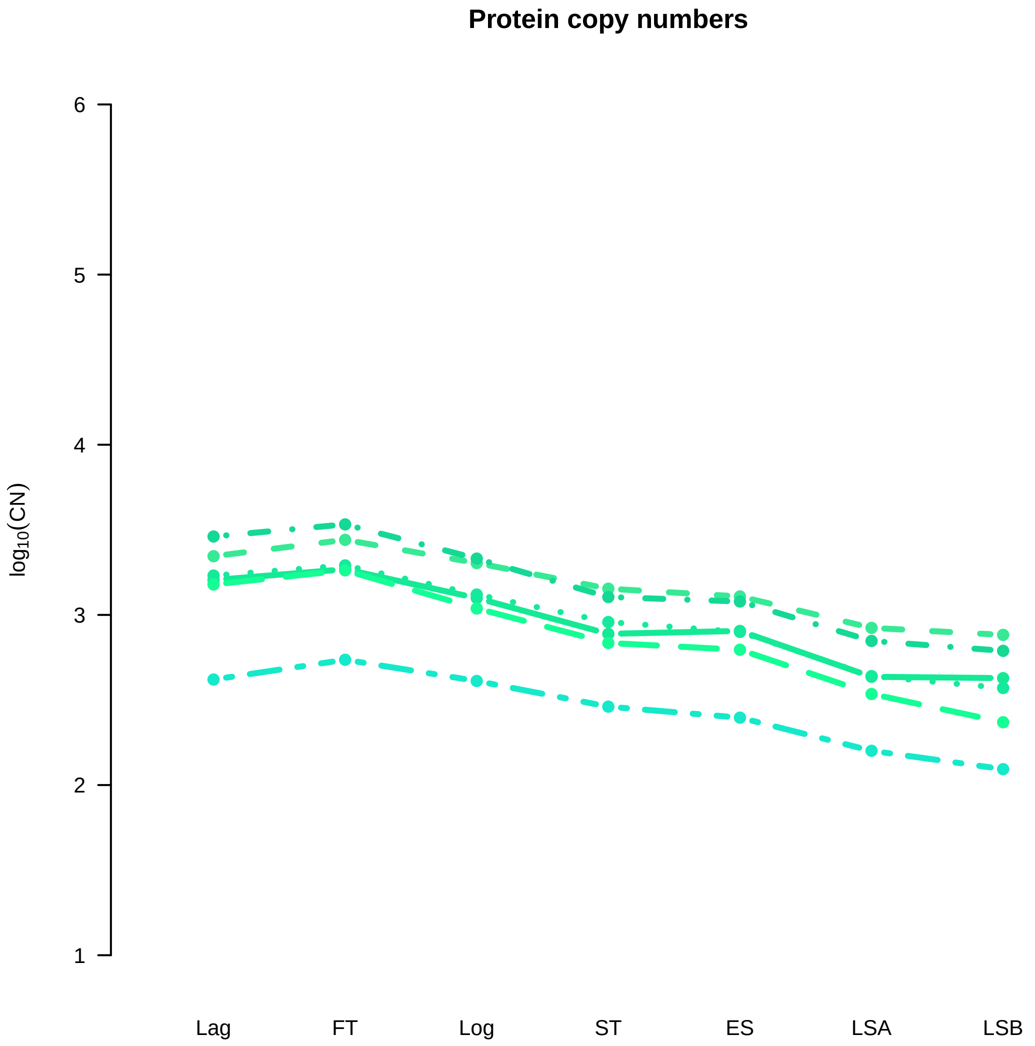

nuoB	B1X8Z9;C4ZUD0;P0AFC7	NADH dehydrogenase I subunit B ...
nuoC	B1X8Z8;C4ZUC9;P33599	NADH dehydrogenase I subunit C ...
nuoA	–	–
nuoF	P31979	NADH dehydrogenase I subunit F ...
nuoG	P33602	NADH dehydrogenase I subunit G ...
nuoE	B1X8Z7;C4ZUC8;P0AFD1	NADH:ubiquinone oxidoreductase ...
nuoJ	–	–
nuoK	–	–
nuoH	–	–
nuoI	P0AFD6	NADH dehydrogenase I subunit I ...
nuoN	–	–
nuoL	P33607	NADH dehydrogenase I subunit L ...
nuoM	P0AFE8	NADH dehydrogenase I subunit M ...

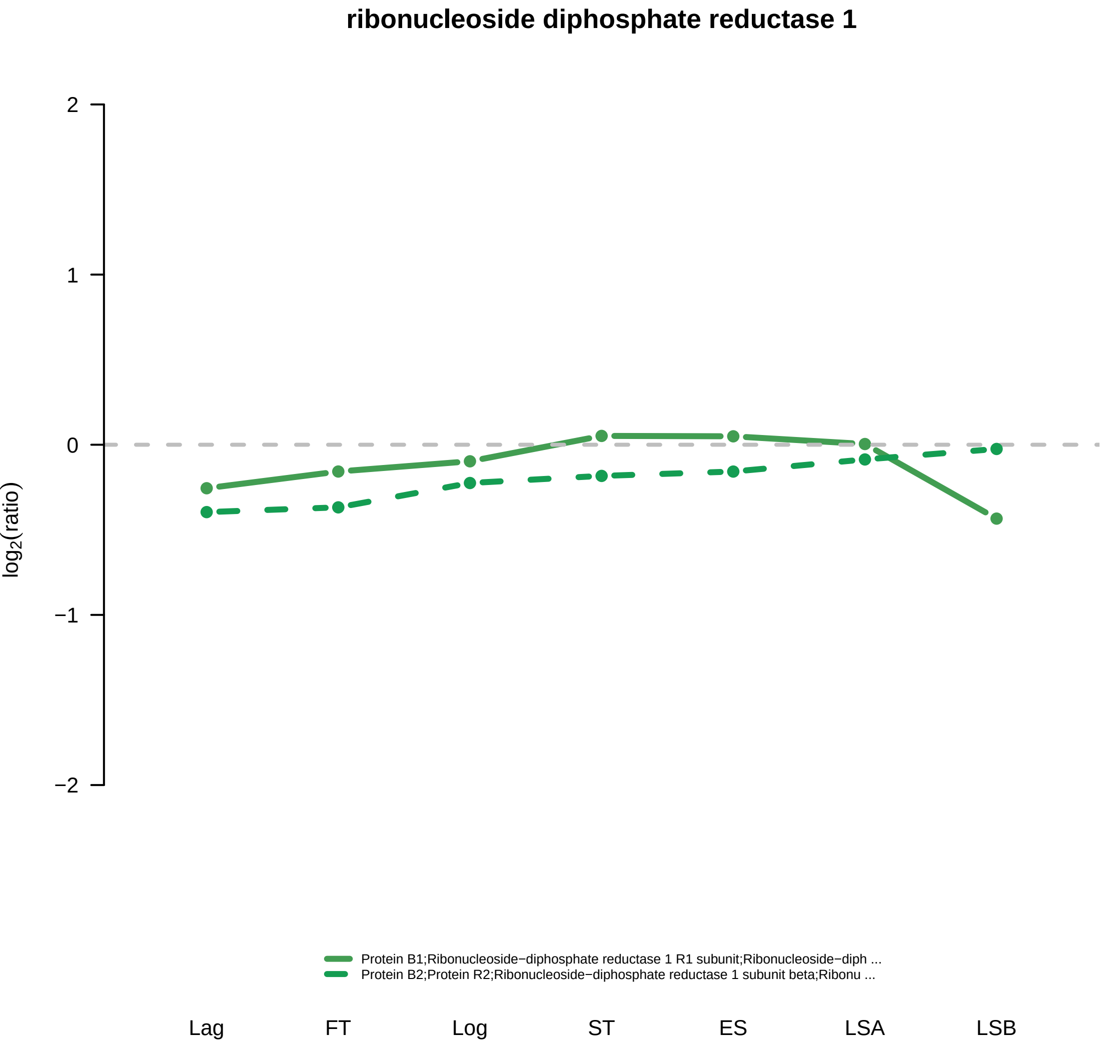

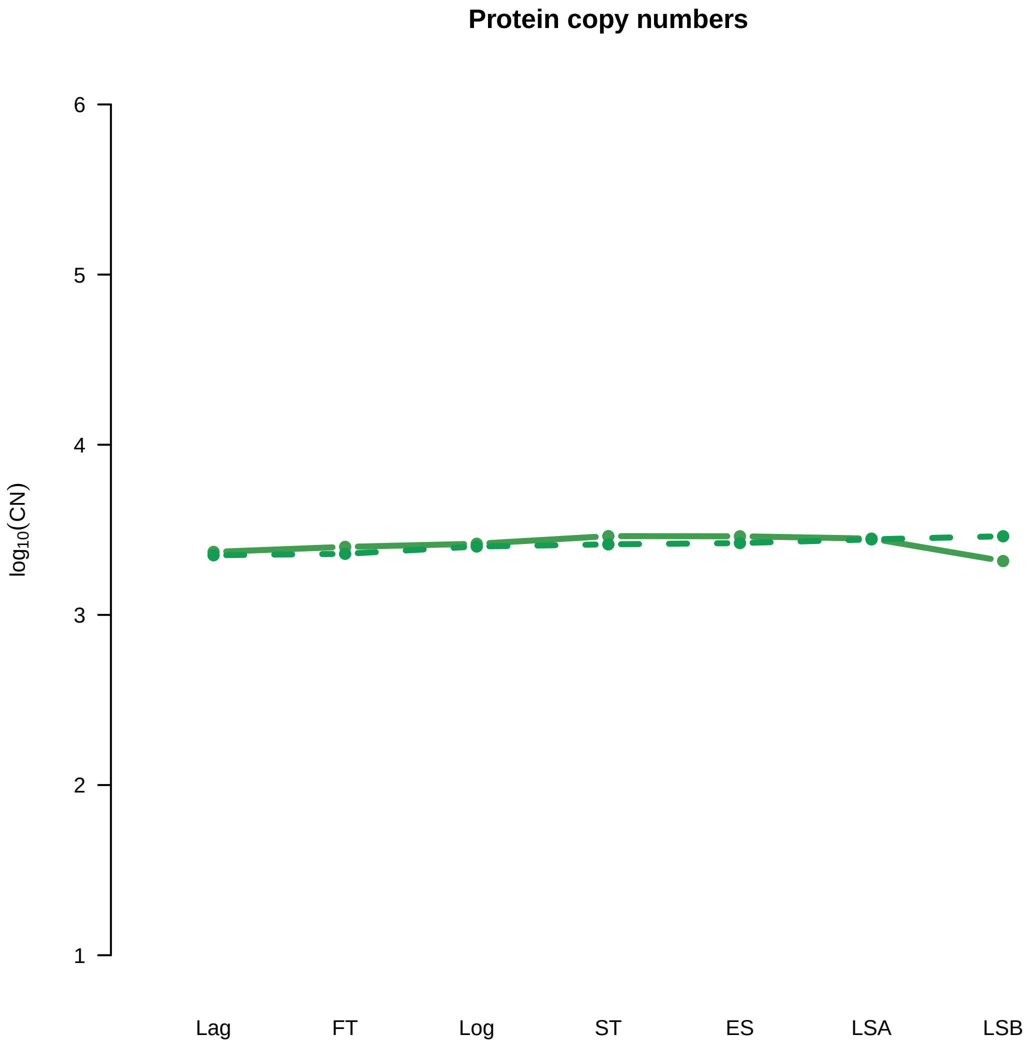

nrdA	P00452;P00452−2	Protein B1;Ribonucleoside−diph ...
nrdB	P69924	Protein B2;Protein R2;Ribonucl ...

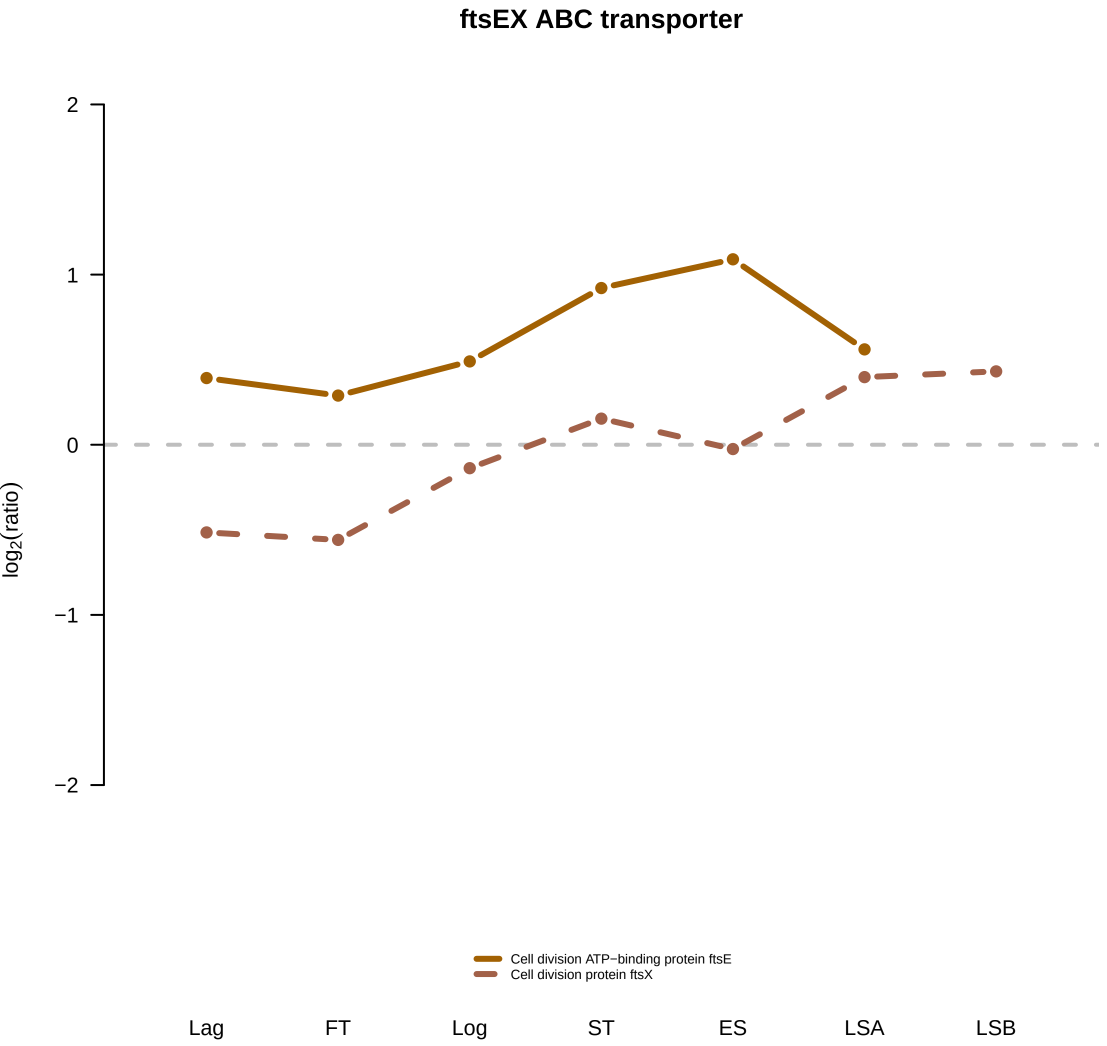

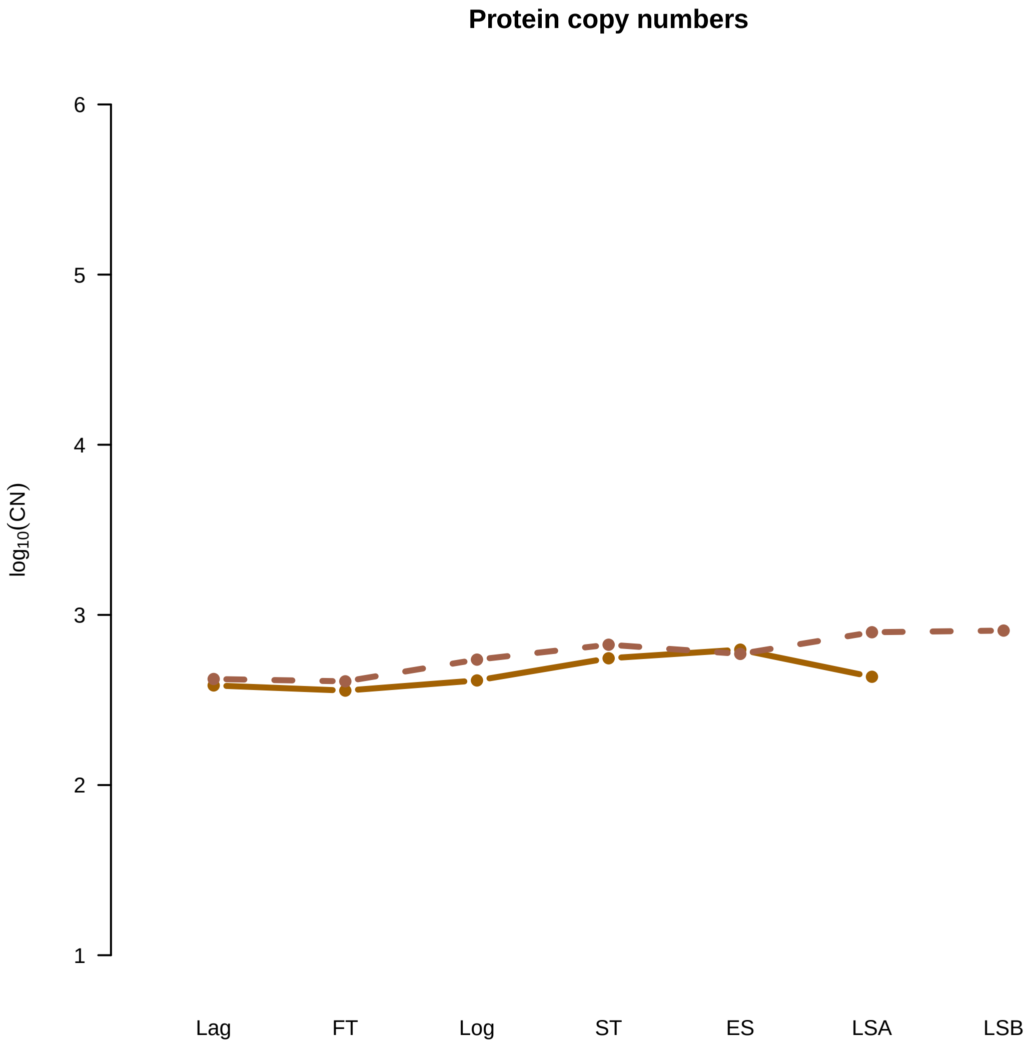

ftsE	P0A9R7	Cell division ATP−binding prot ...
ftsX	P0AC30	Cell division protein ftsX

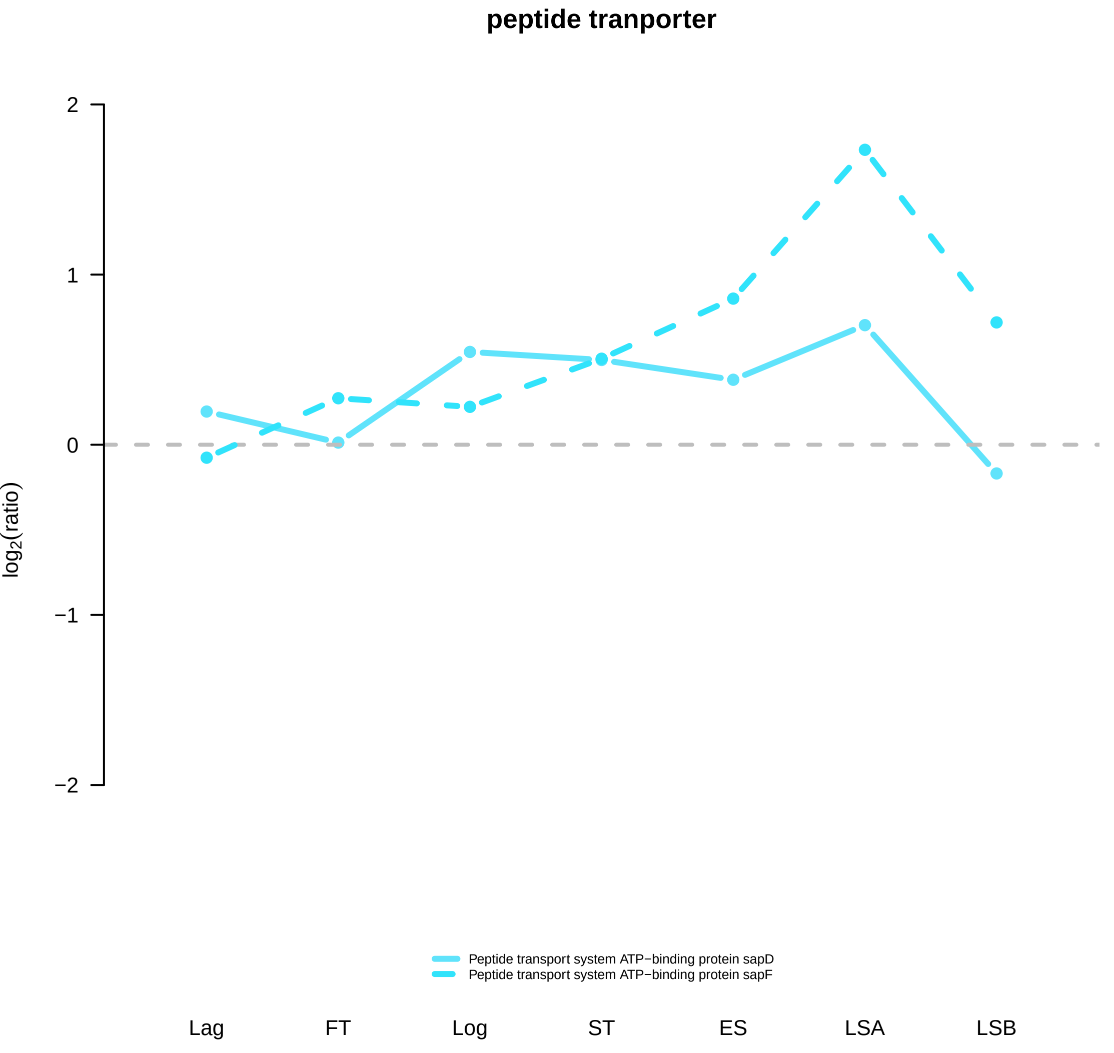

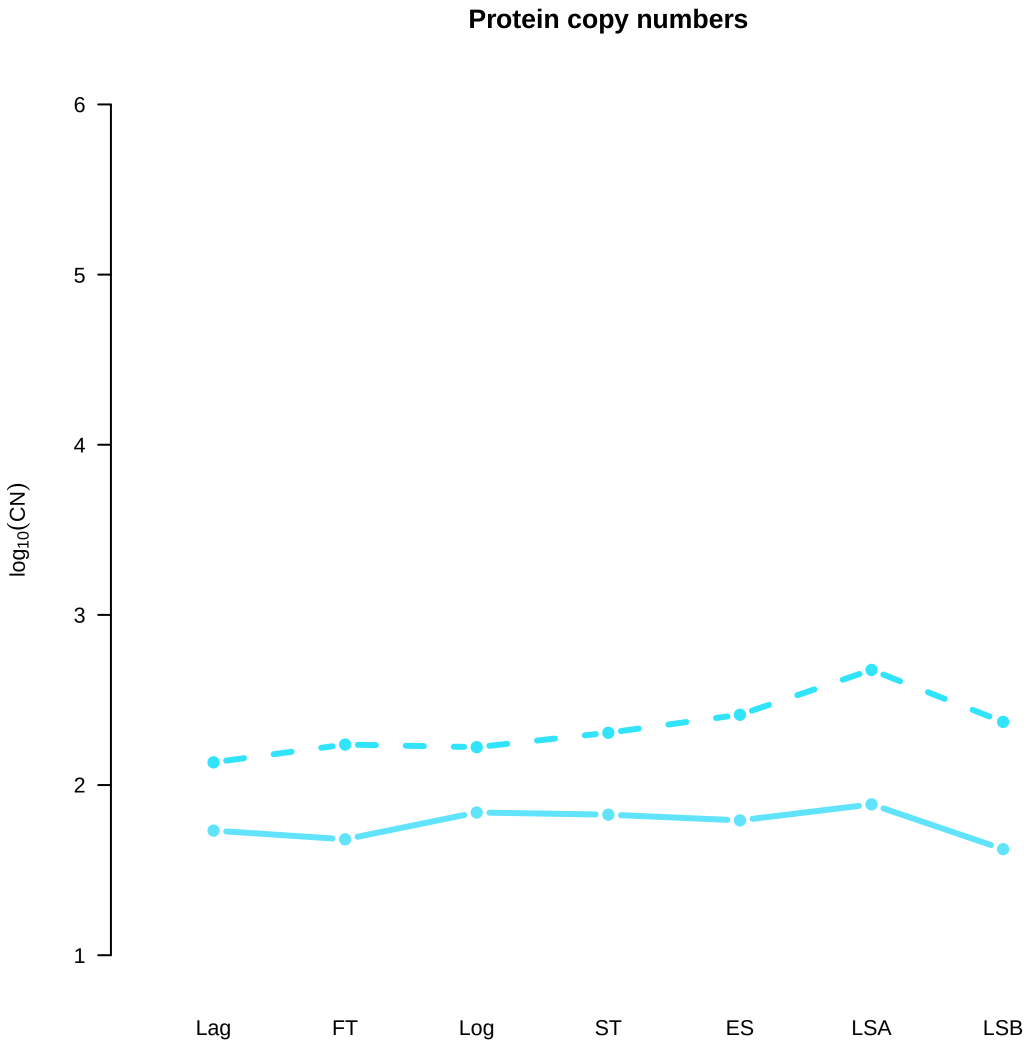

sapD	P0AAH4	Peptide transport system ATP−b ...
sapF	P0AAH8	Peptide transport system ATP−b ...
sapB	−	−
sapC	P0AGH5	Peptide transport system perme ...

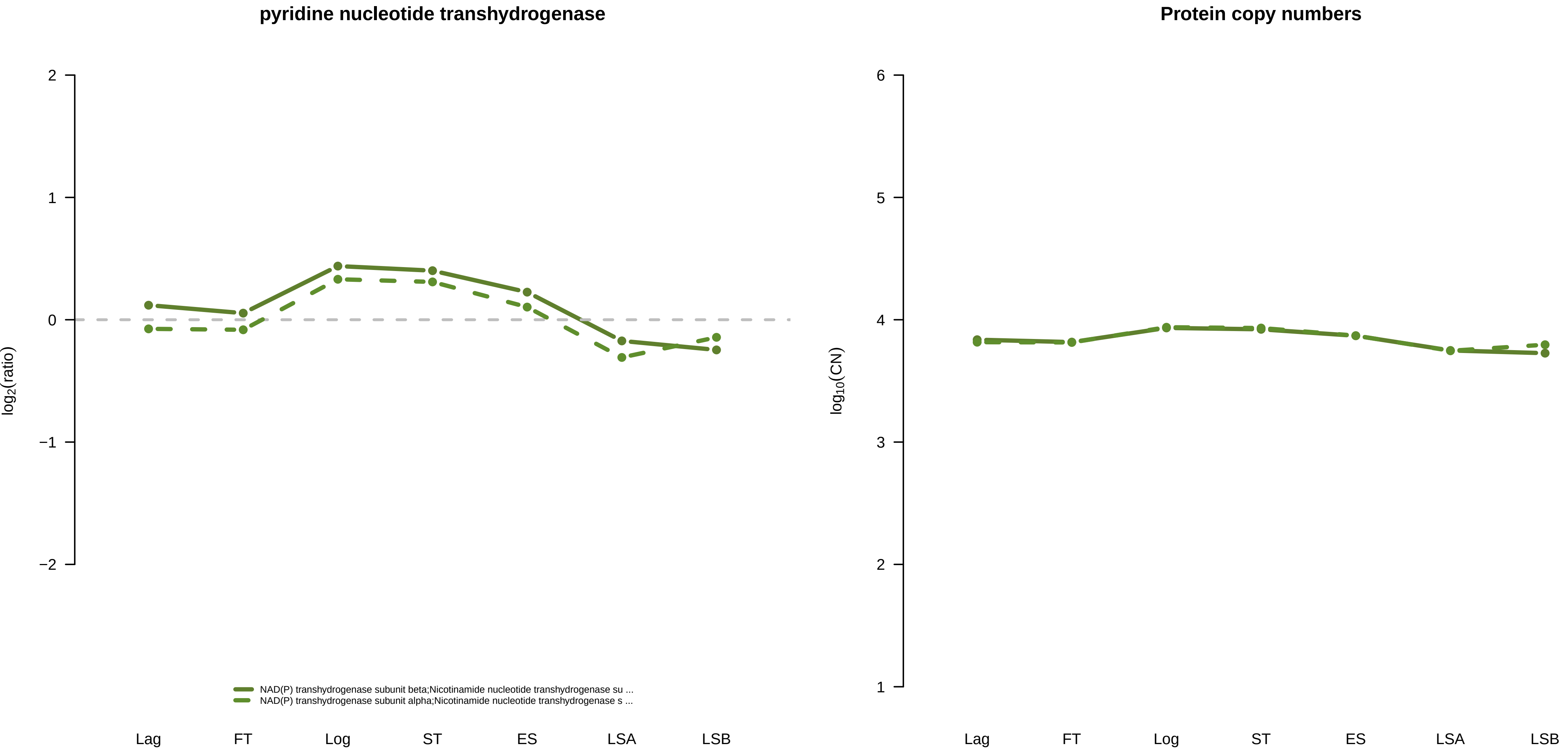

pntB	P0AB67	NAD(P) transhydrogenase subuni ...
pntA	P07001	NAD(P) transhydrogenase subuni ...

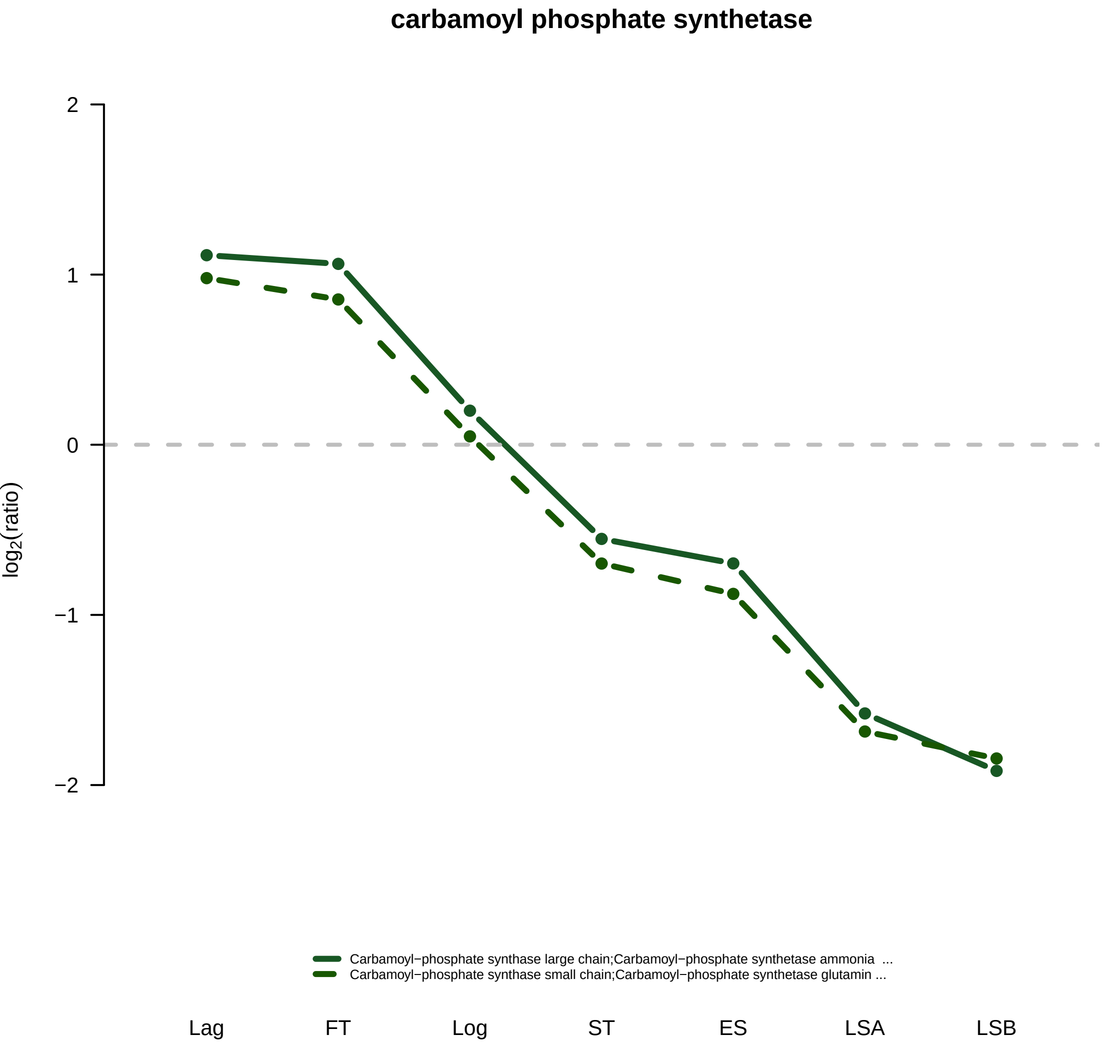

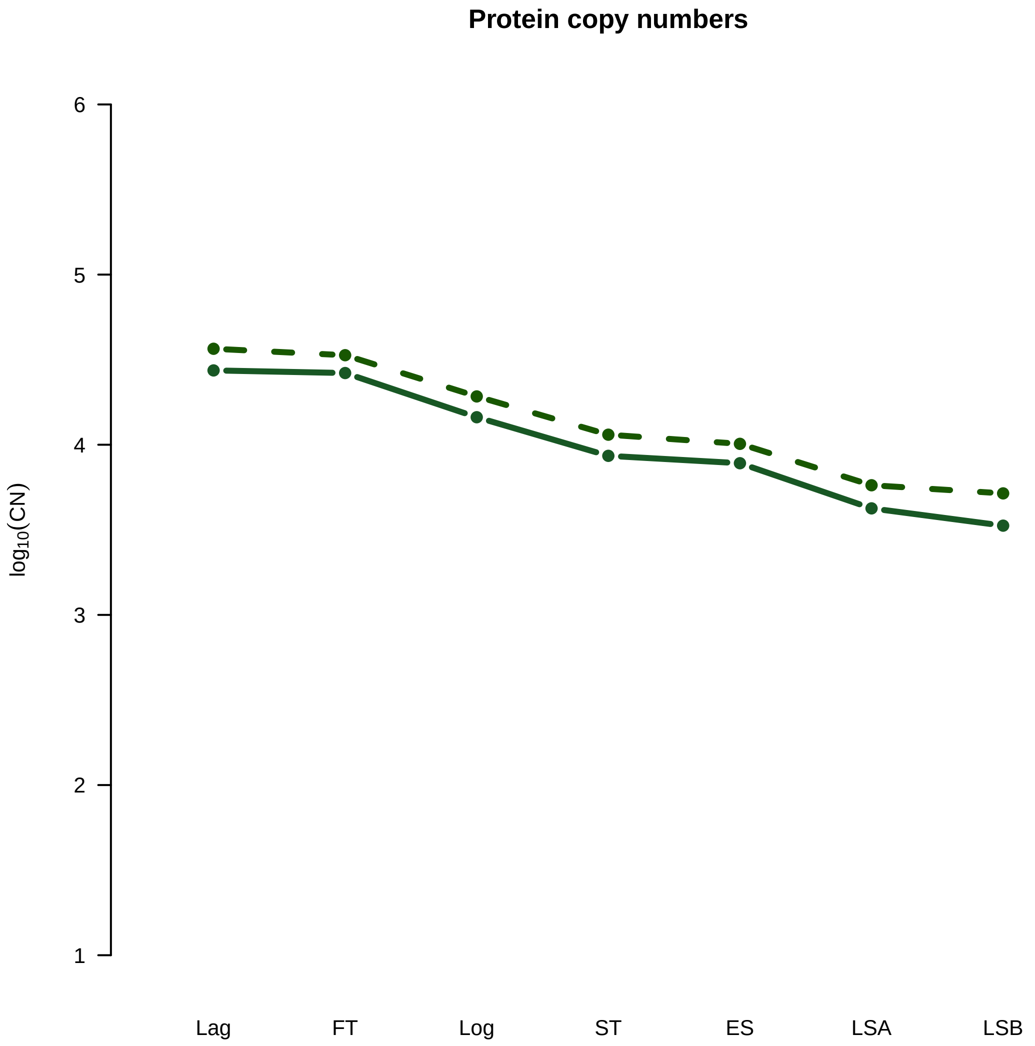

carB	P00968	Carbamoyl−phosphate synthase l ...
carA	P0A6F1	Carbamoyl−phosphate synthase s ...

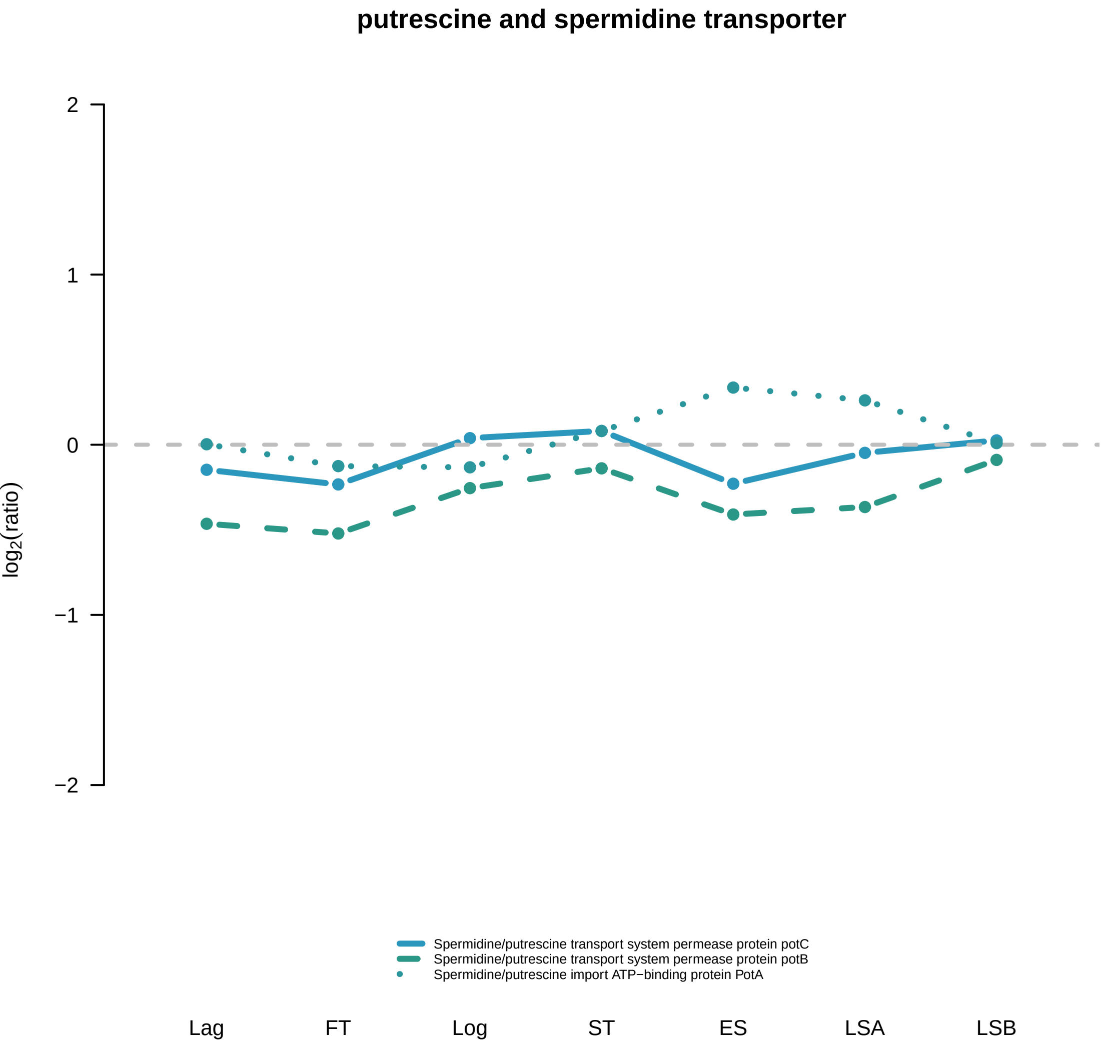

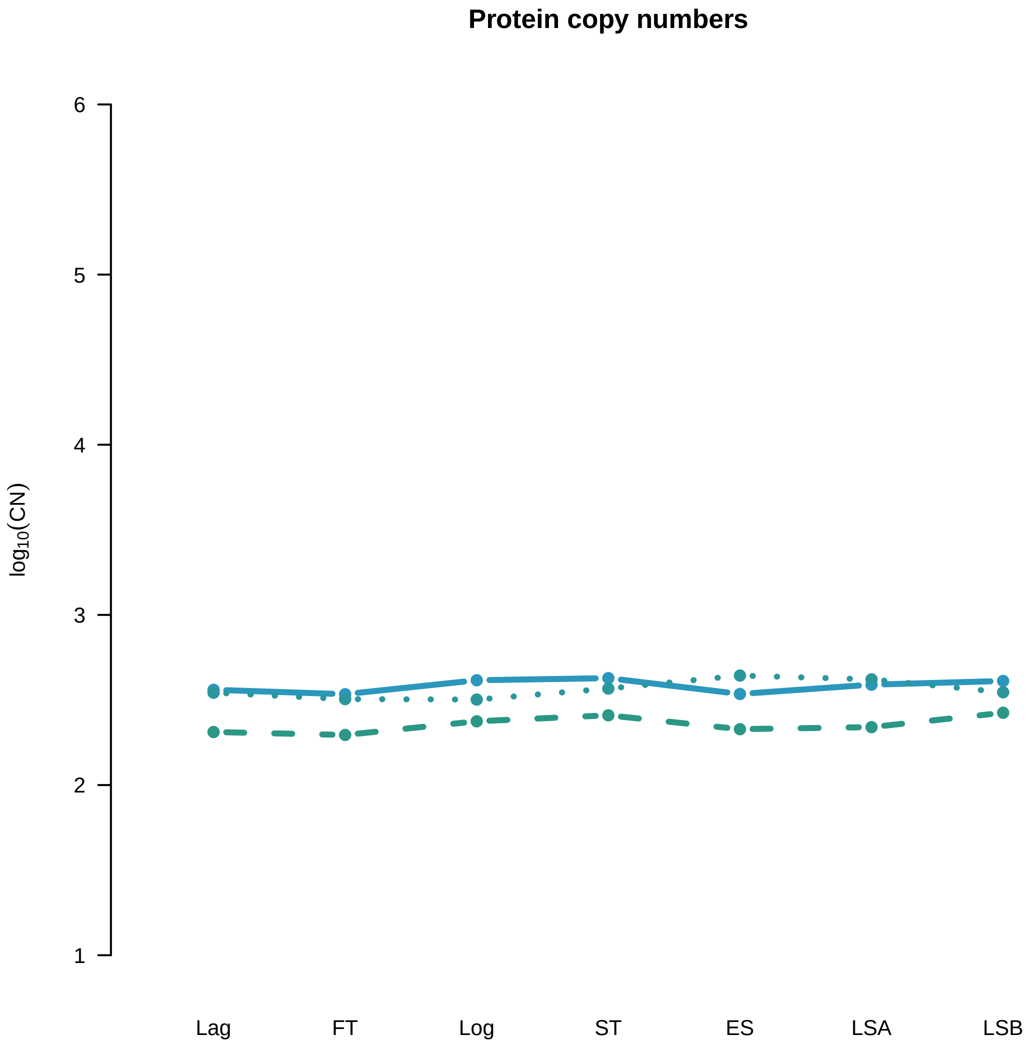

potC	P0AFK6	Spermidine/putrescine transpor ...
potB	P0AFK4	Spermidine/putrescine transpor ...
potA	P69874	Spermidine/putrescine import A ...

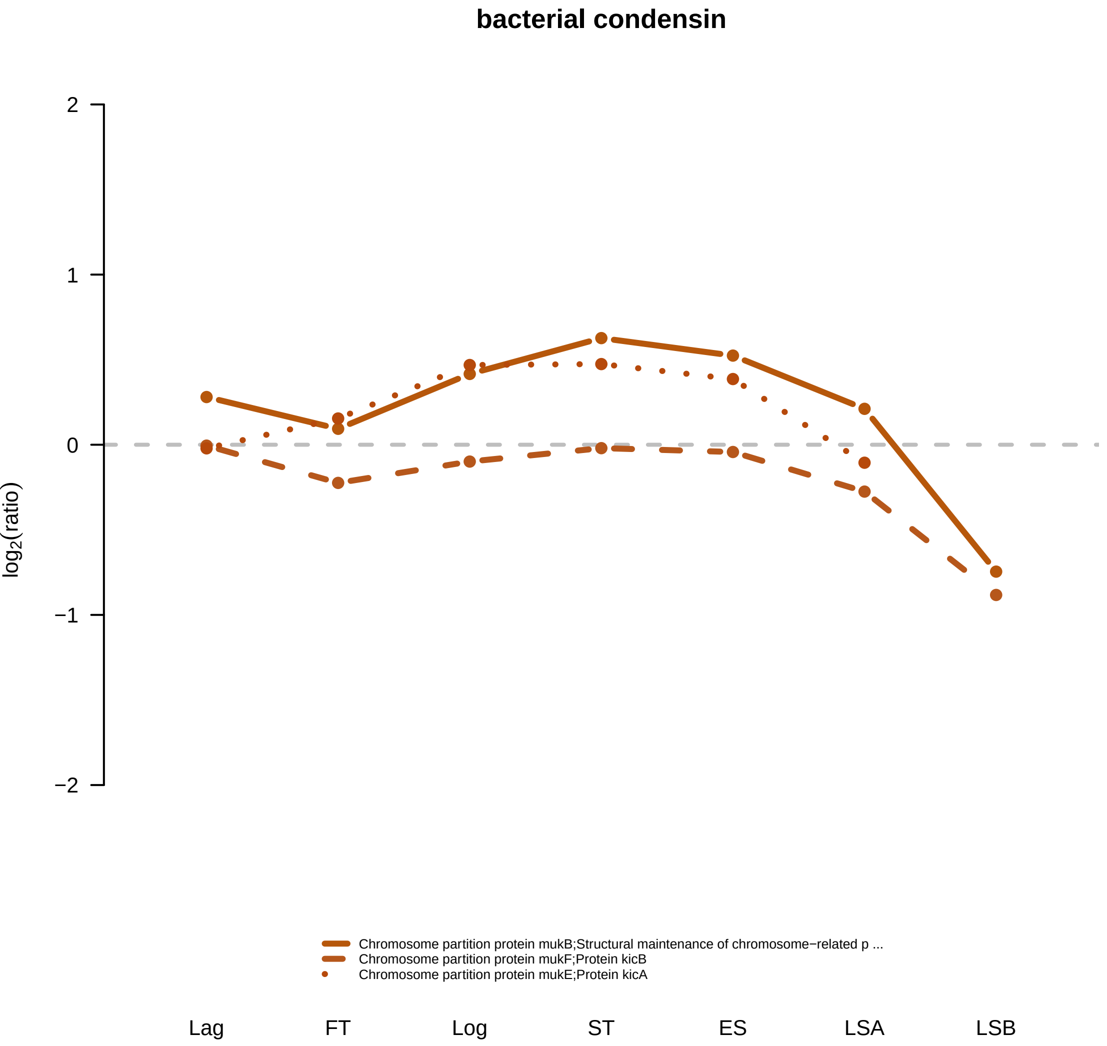

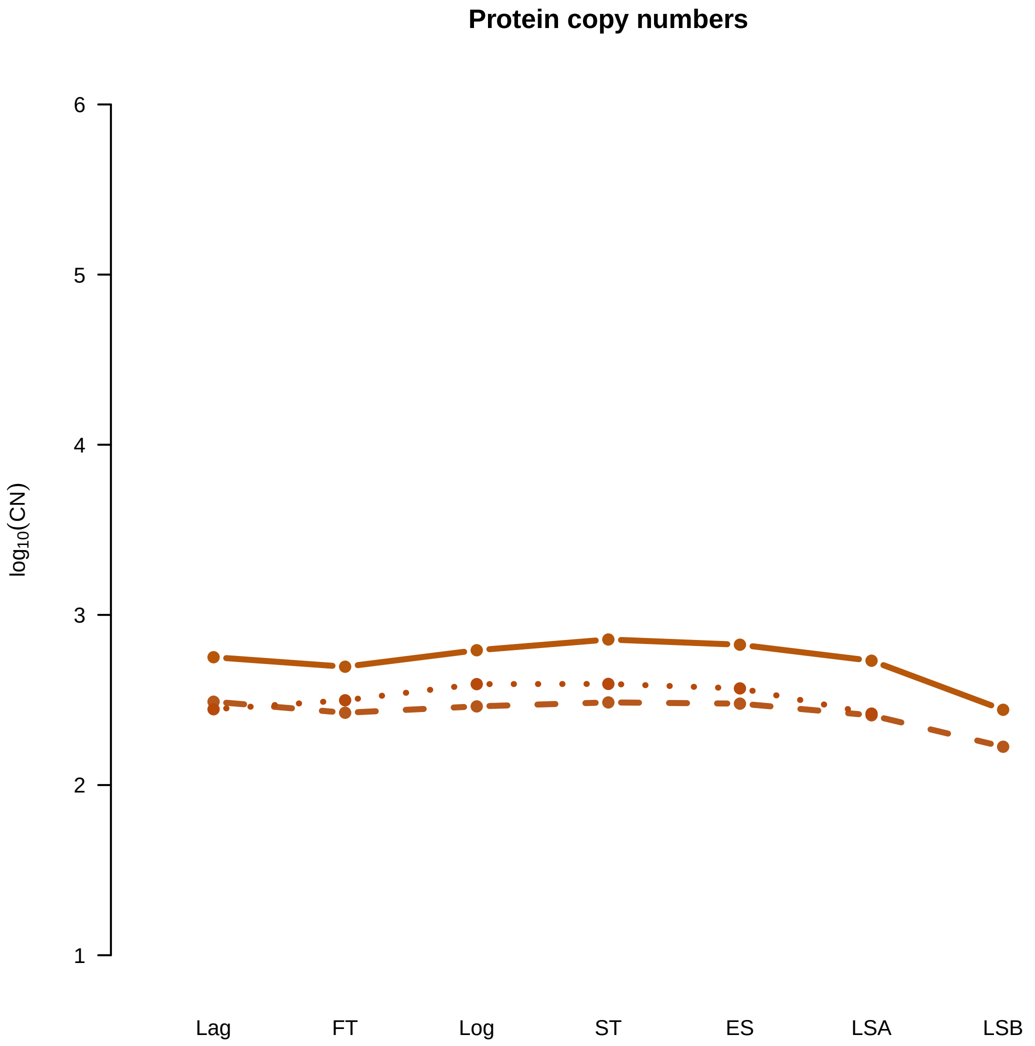

mukB	P22523	Chromosome partition protein m ...
mukF	P60293	Chromosome partition protein m ...
mukE	P22524	Chromosome partition protein m ...

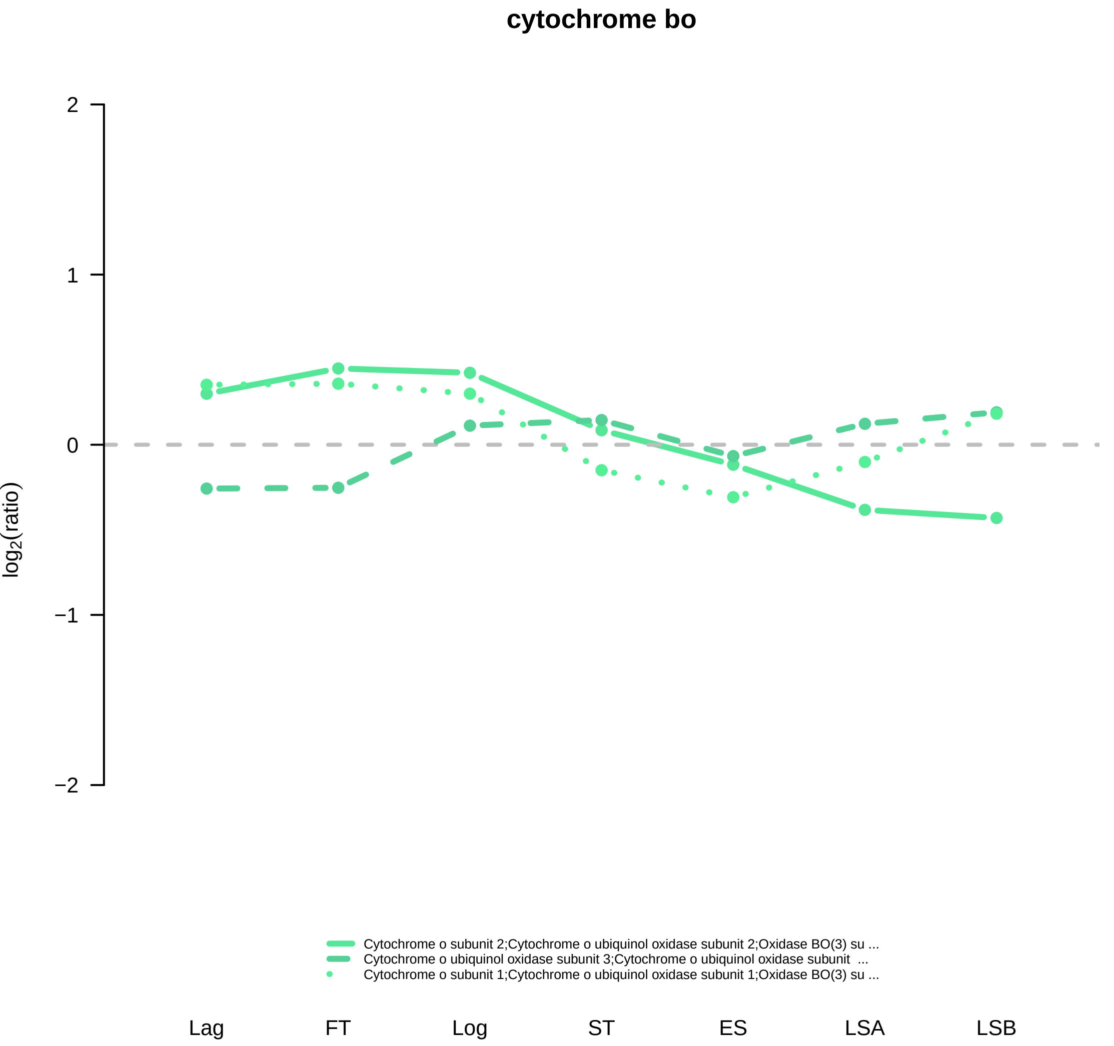

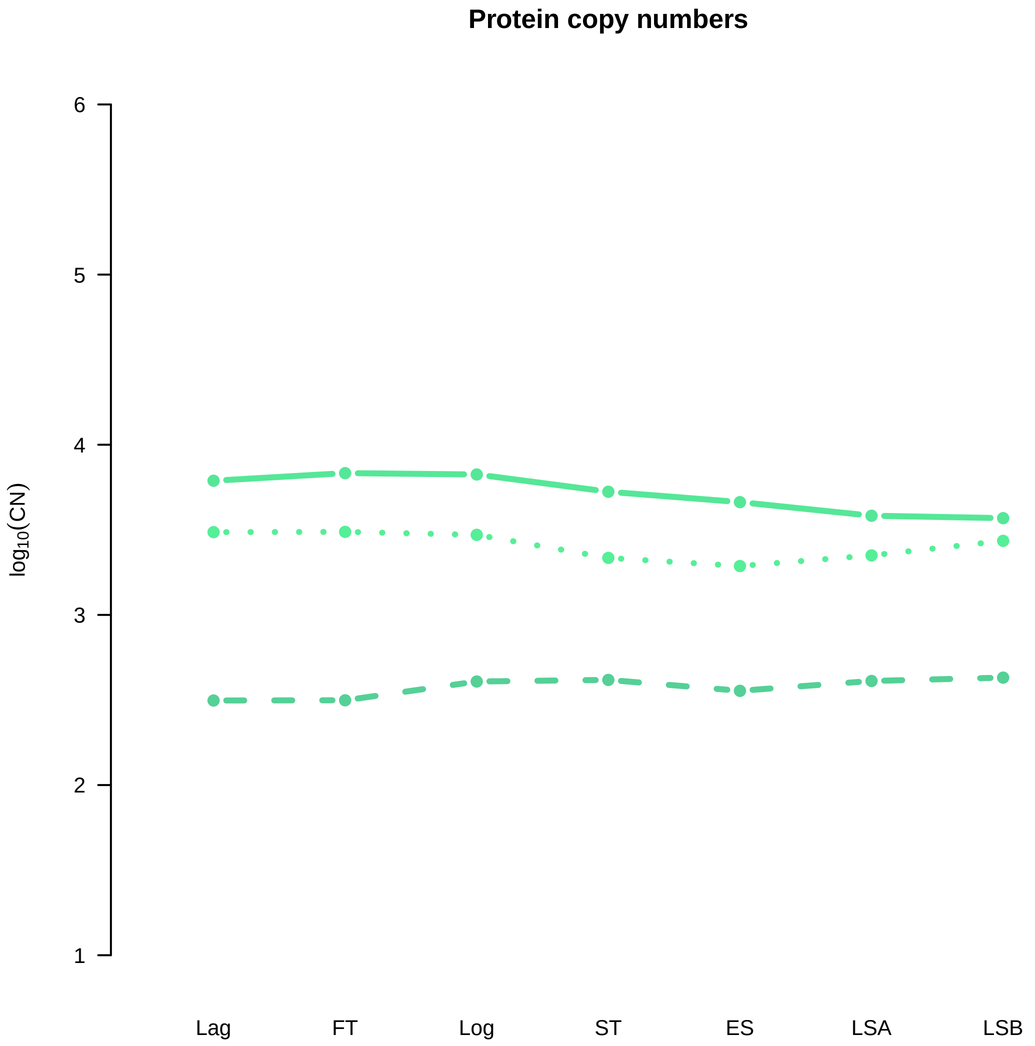

cyoA	P0ABJ1	Cytochrome o subunit 2;Cytochr ...
cyoC	P0ABJ3	Cytochrome o ubiquinol oxidase ...
cyoB	P0ABI8	Cytochrome o subunit 1;Cytochr ...
cyoD	P0ABJ6	Cytochrome o ubiquinol oxidase ...

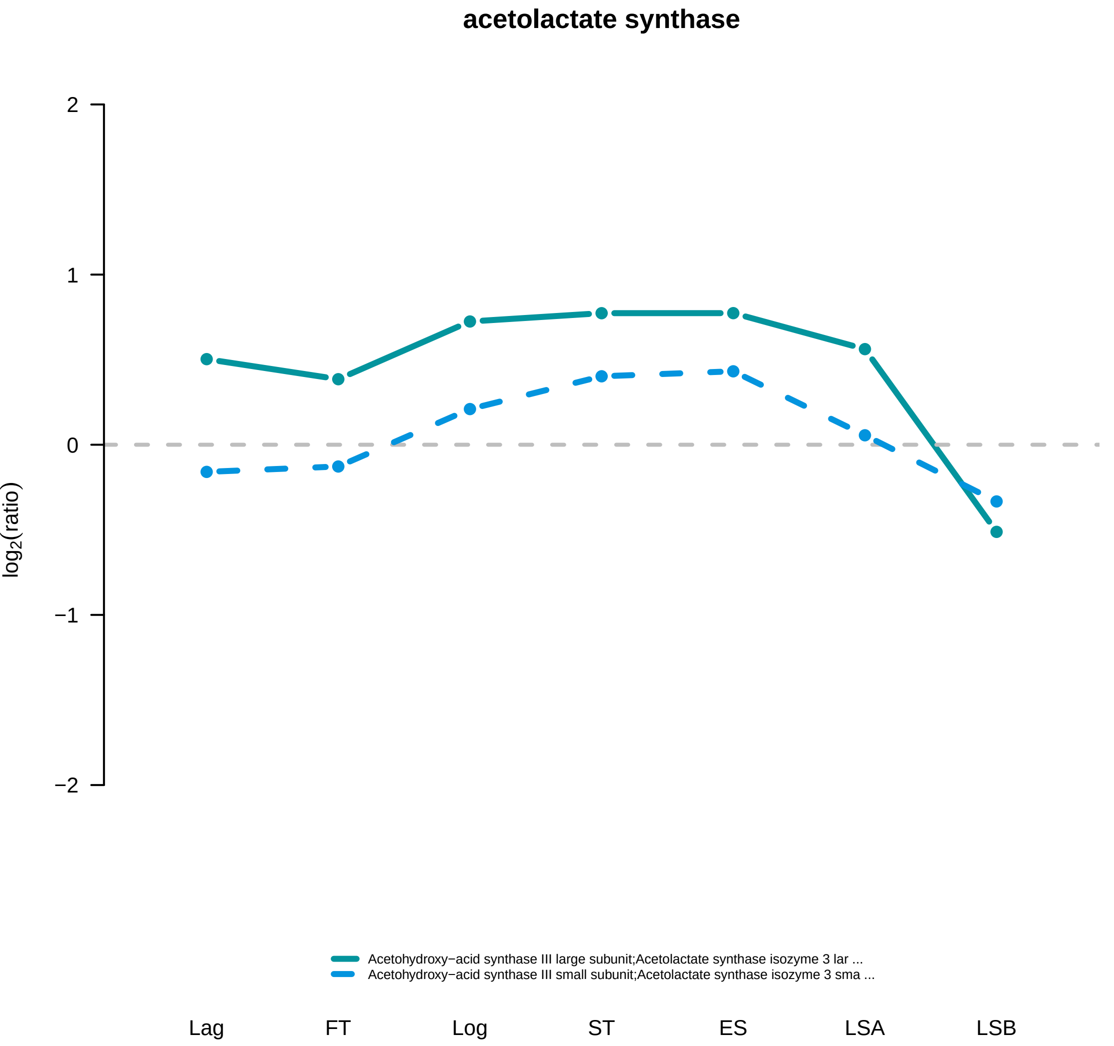

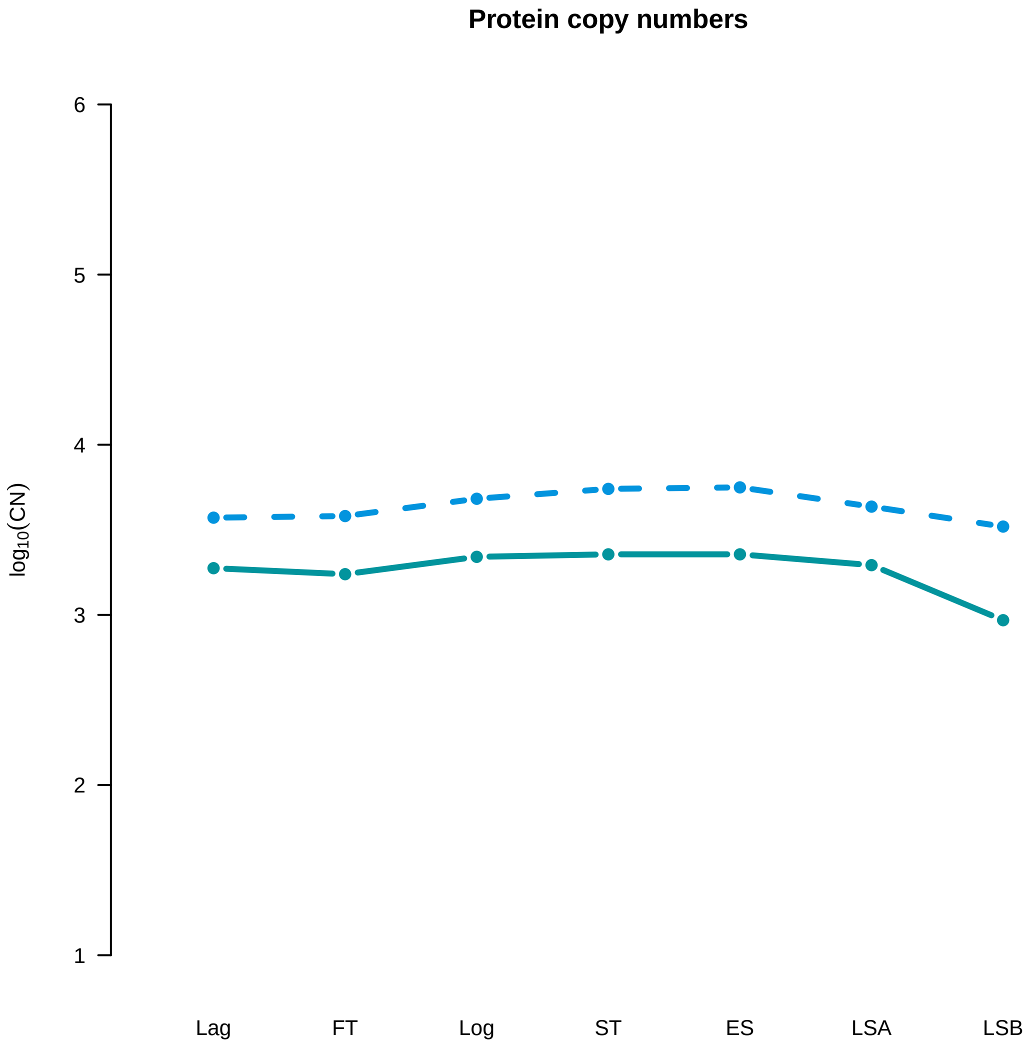

ilvI	P00893	Acetohydroxy−acid synthase III ...
ilvH	P00894	Acetohydroxy−acid synthase III ...

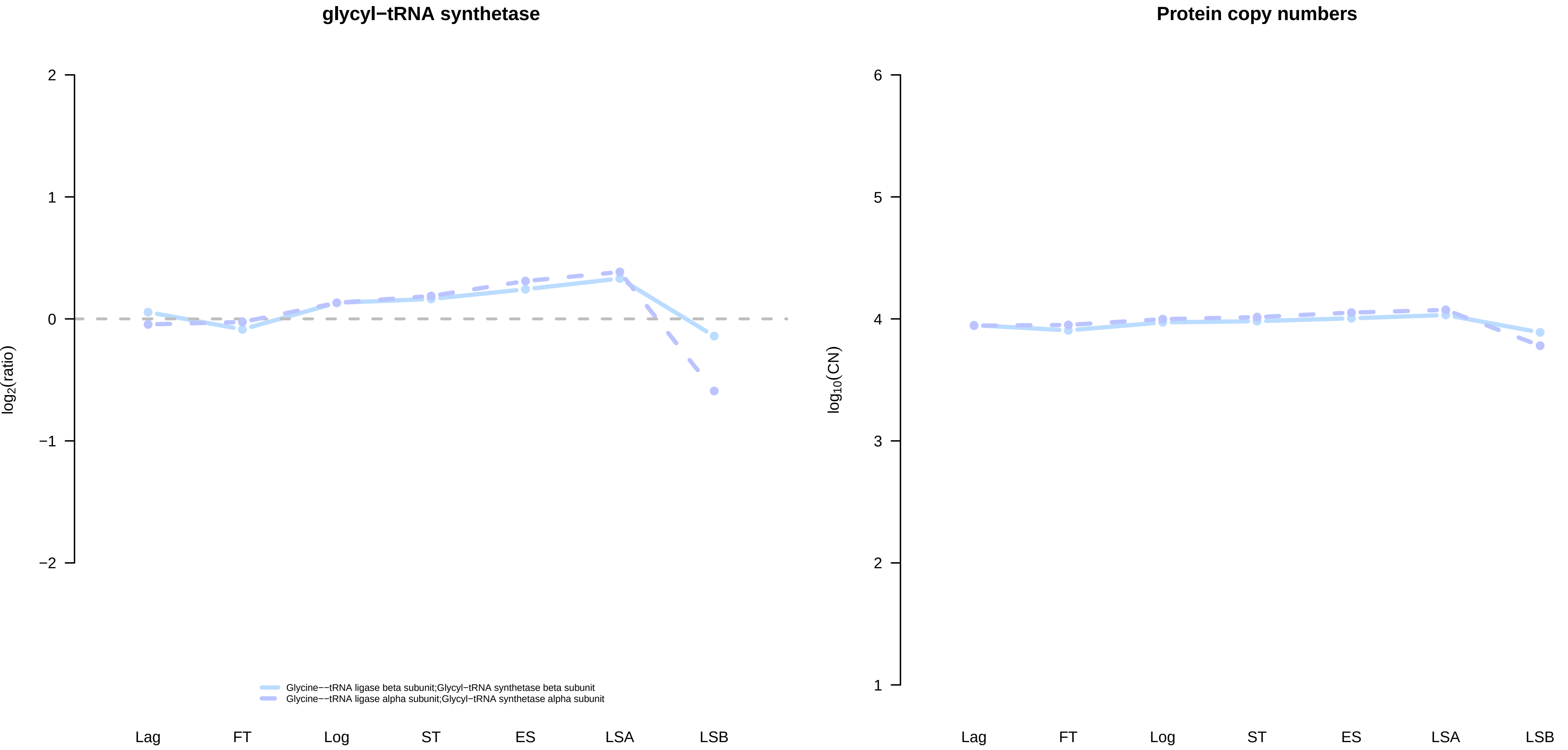

glyS P00961 Glycine−−tRNA ligase beta subu ...
glyQ P00960 Glycine−−tRNA ligase alpha sub ...

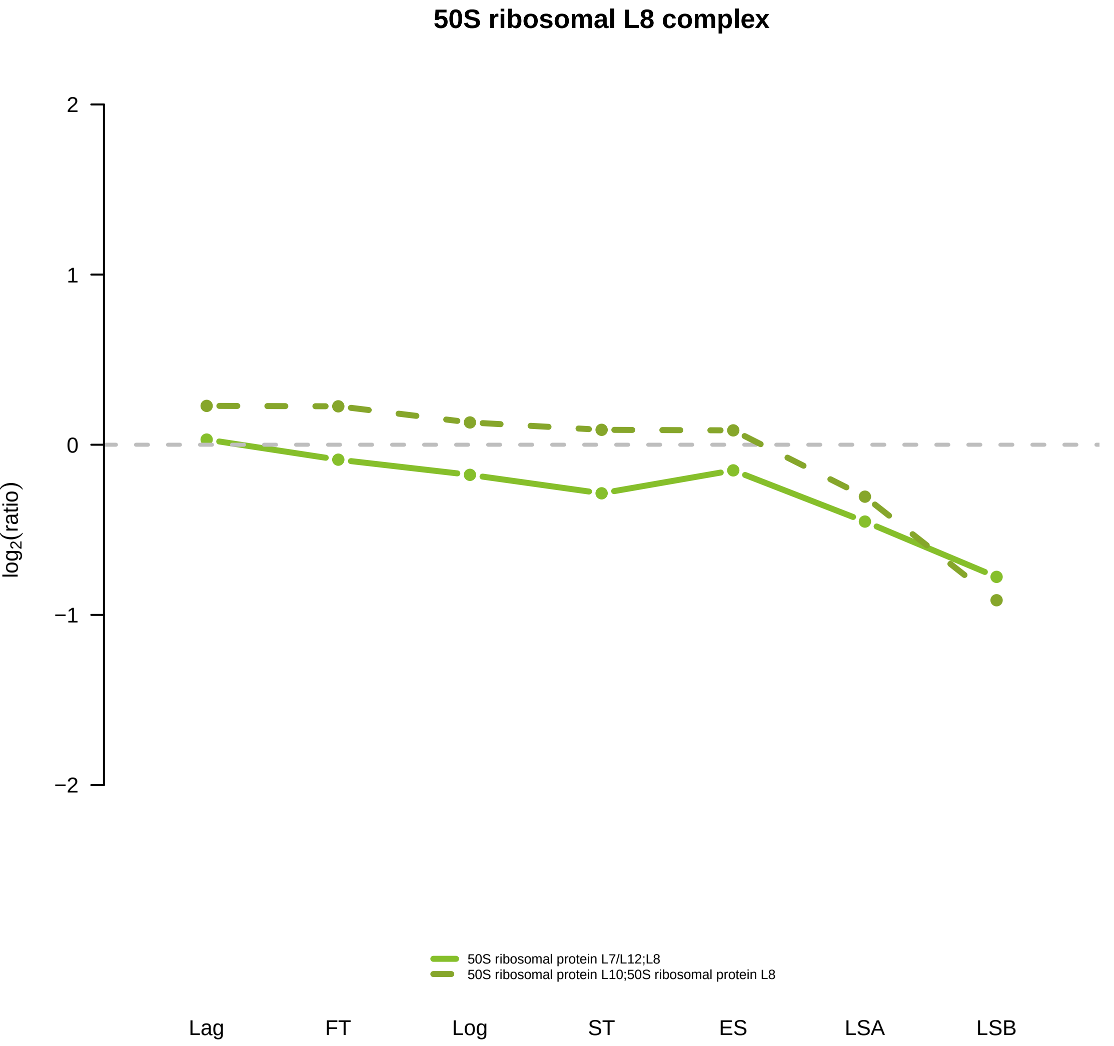

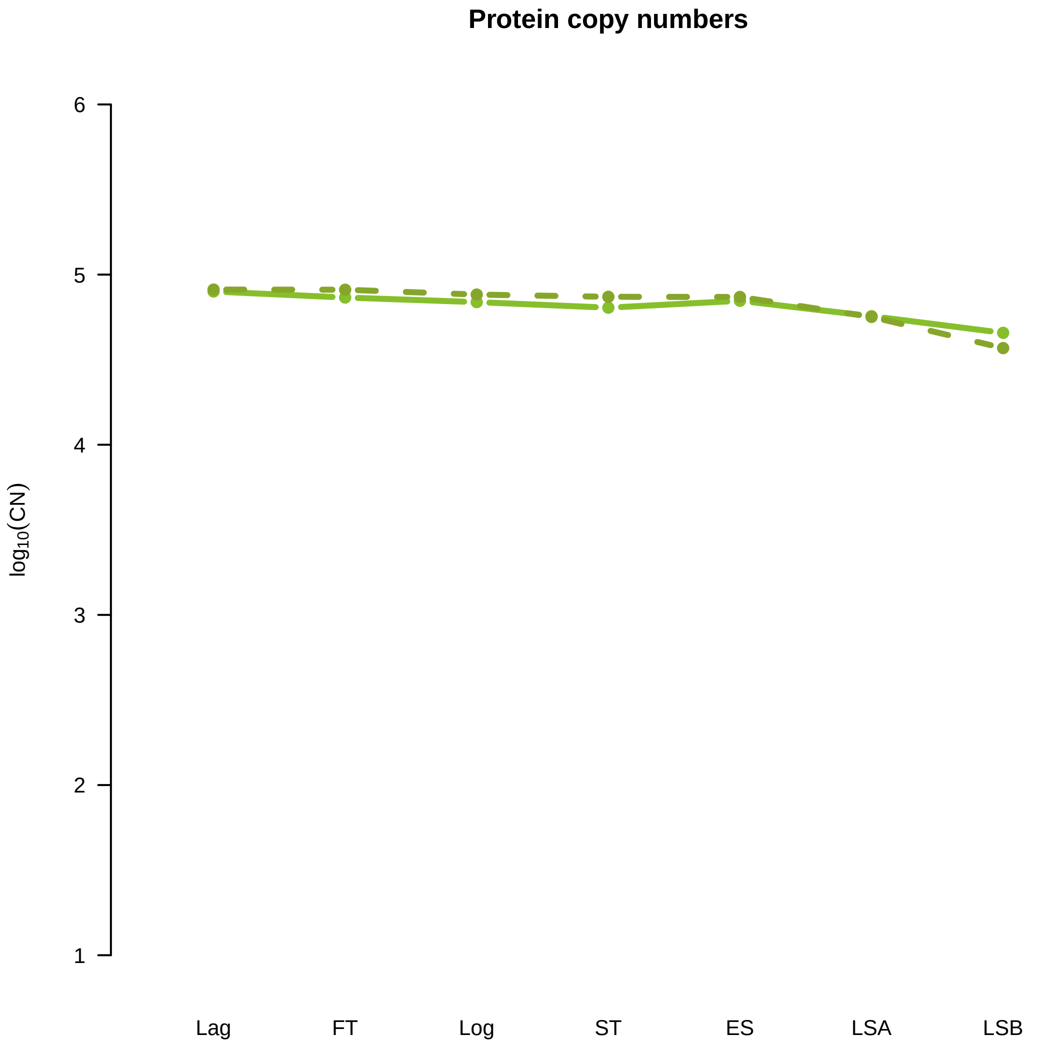

rplL	P0A7K2	50S ribosomal protein L7/L12;L ...
rplJ	P0A7J3	50S ribosomal protein L10;50S ...

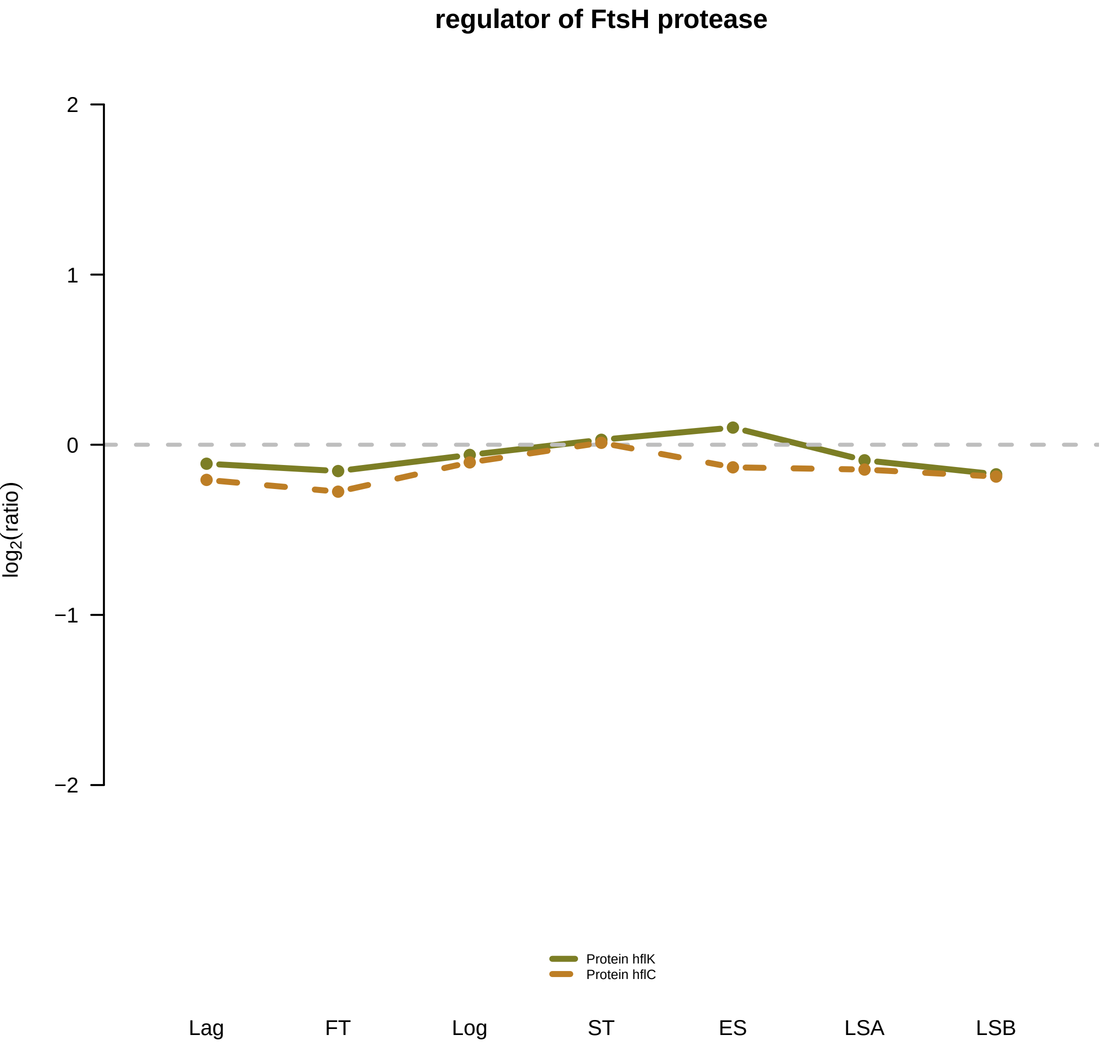

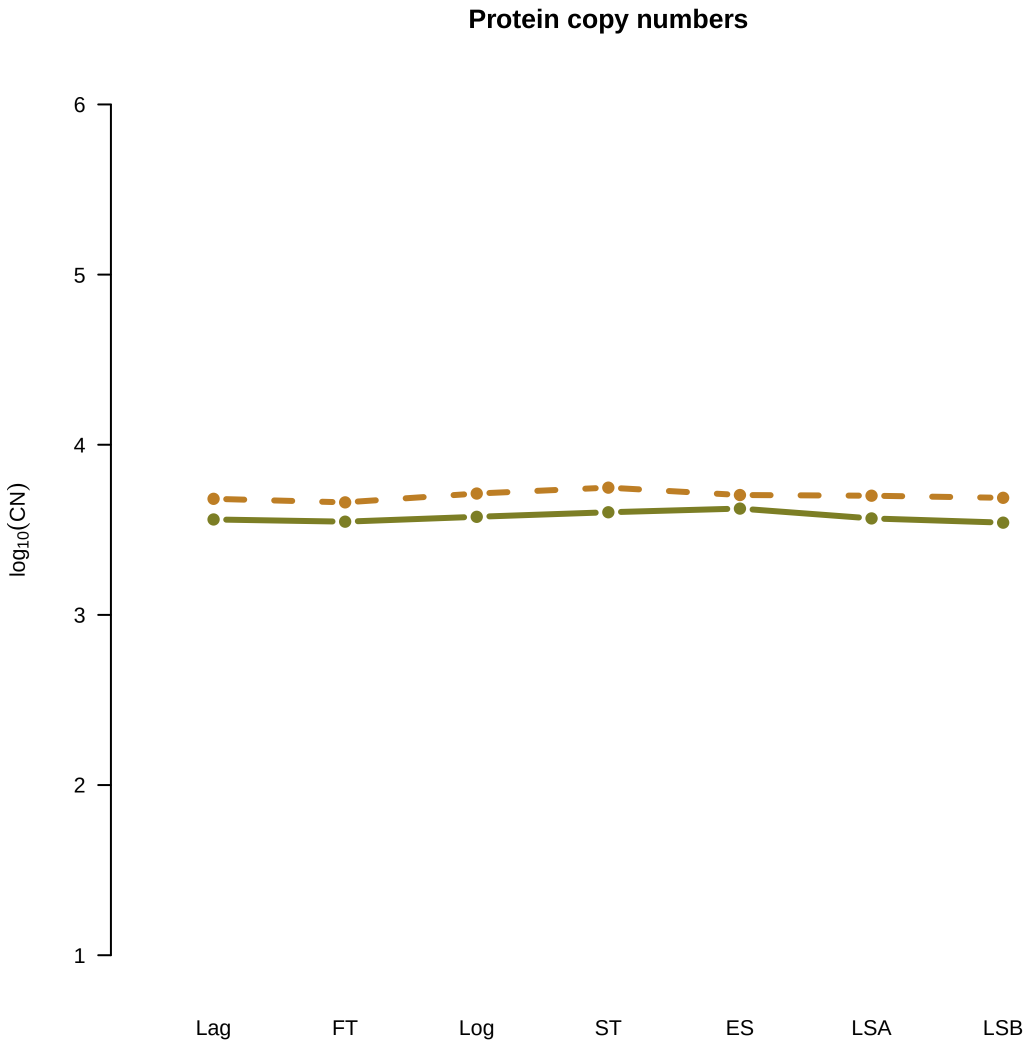

hflK P0ABC7 Protein hflK
hflC P0ABC3 Protein hflC

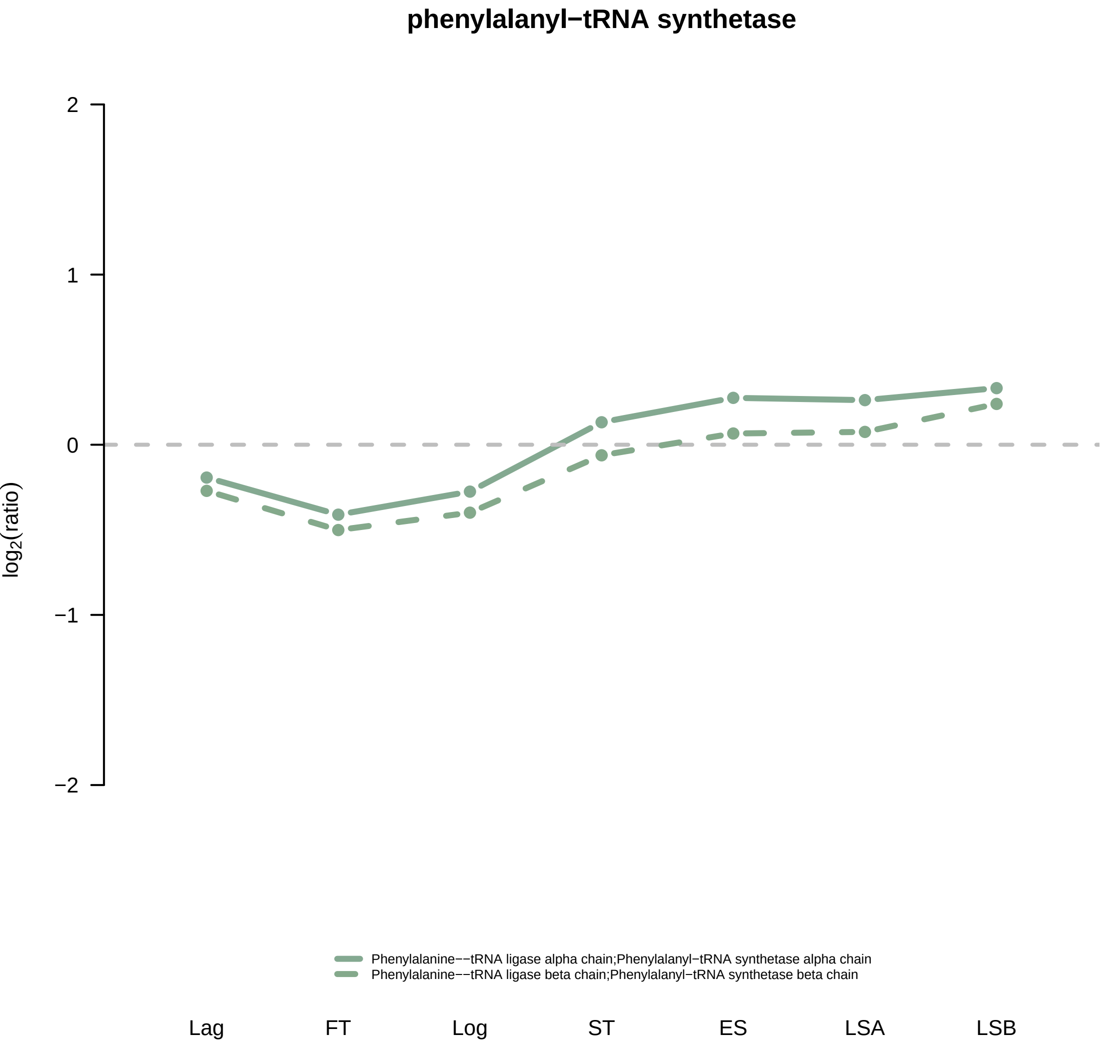

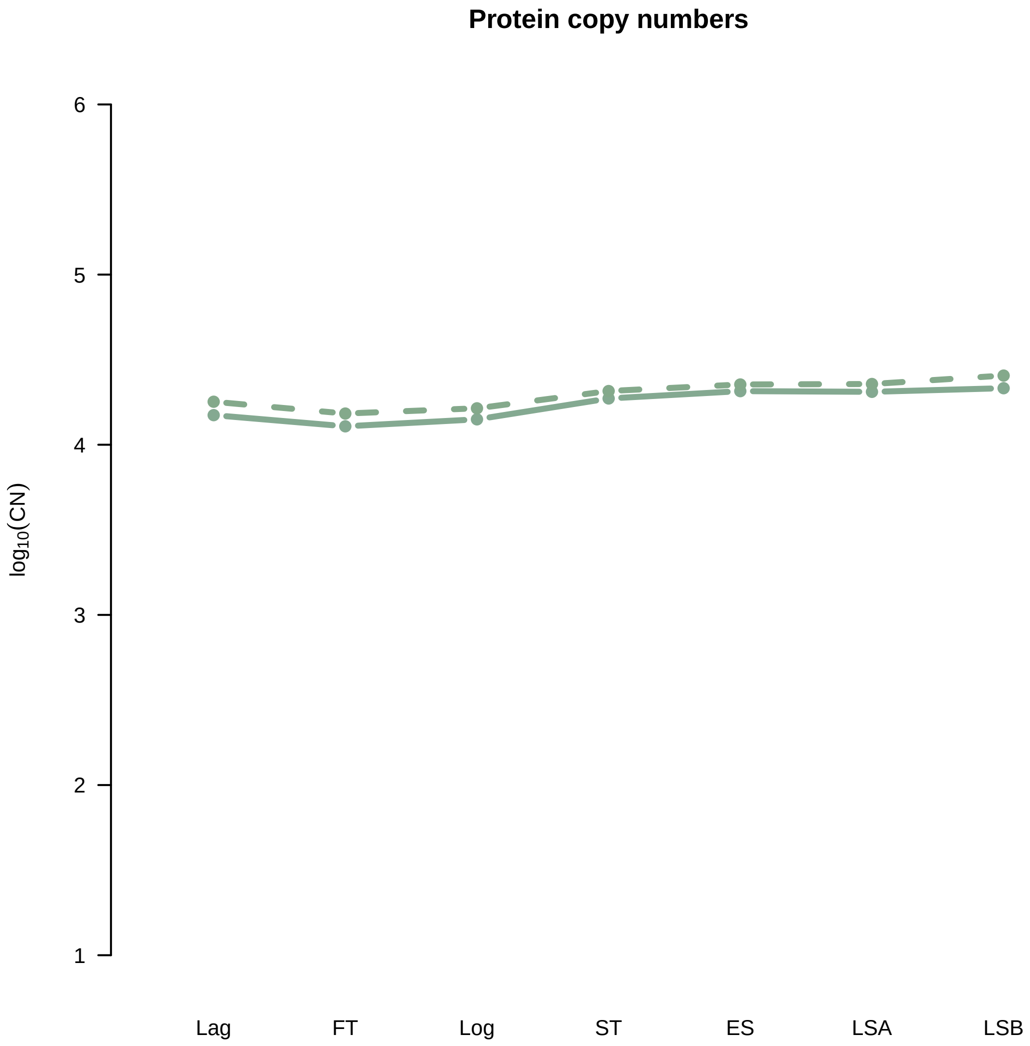

pheS	P08312	Phenylalanine−−tRNA ligase alp ...
pheT	P07395	Phenylalanine−−tRNA ligase bet ...

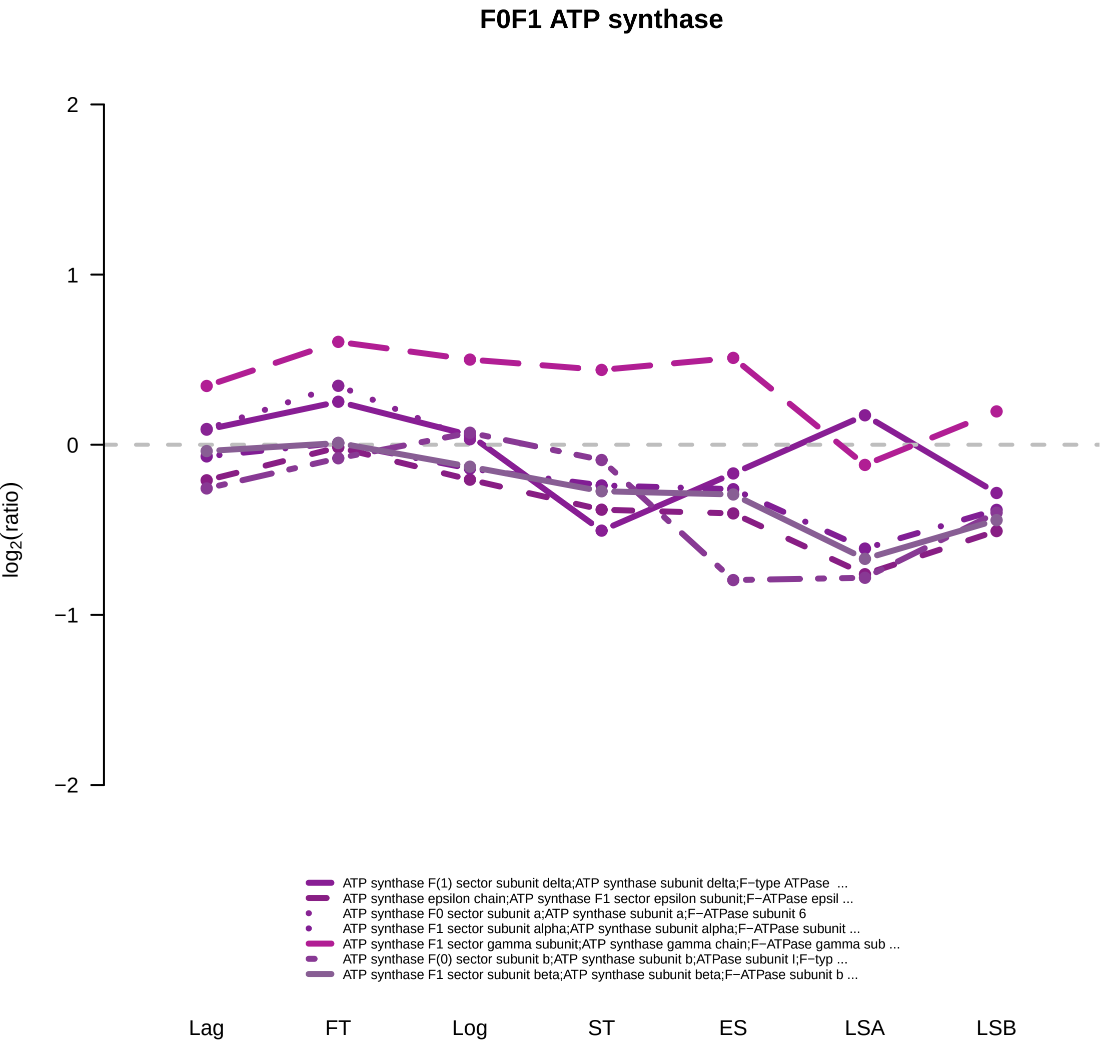

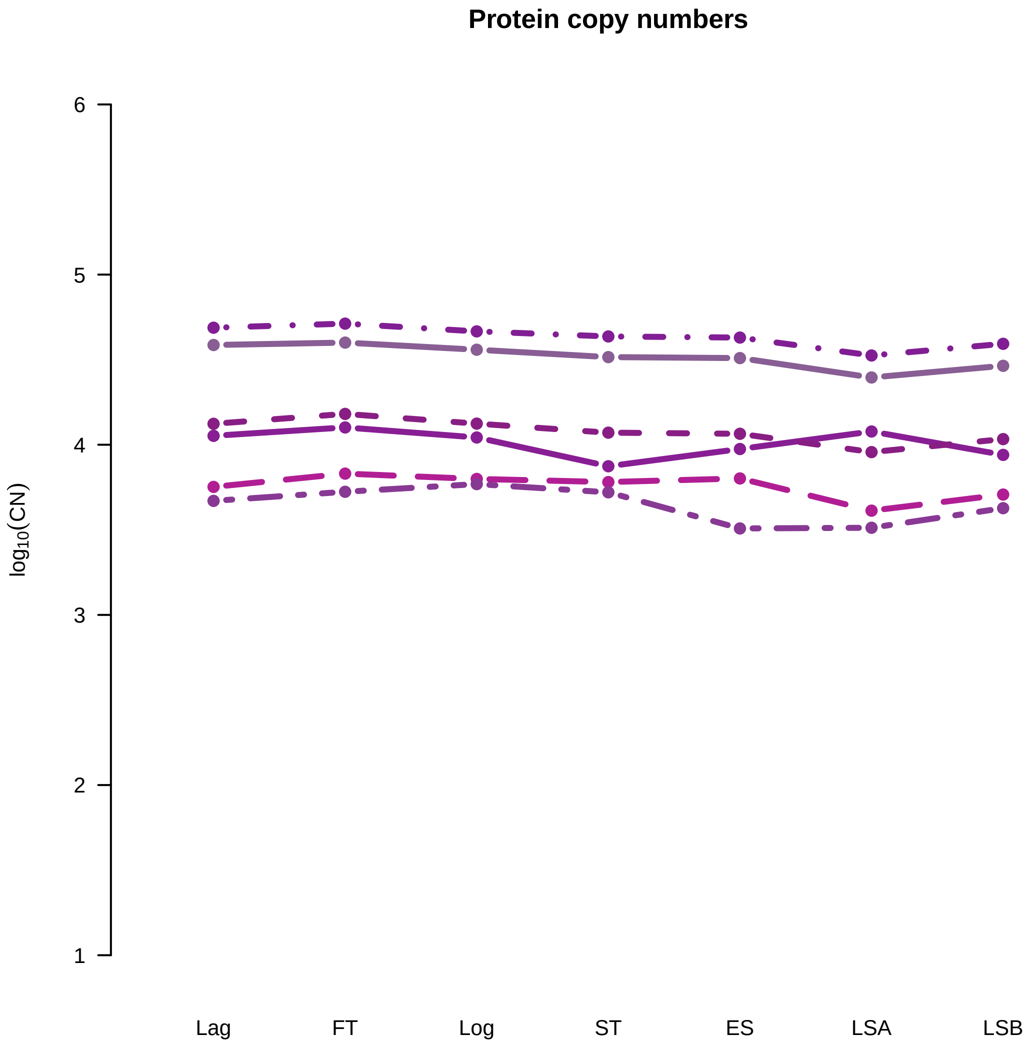

atpH	P0ABA4	ATP synthase F(1) sector subun ...
atpC	B1X9V9;C4ZZ09;P0A6E6	ATP synthase epsilon chain;ATP ...
atpB	P0AB98	ATP synthase F0 sector subunit ...
atpA	P0ABB0	ATP synthase F1 sector subunit ...
atpG	P0ABA6	ATP synthase F1 sector gamma s ...
atpF	P0ABA0	ATP synthase F(0) sector subun ...
atpE	–	–
atpD	P0ABB4	ATP synthase F1 sector subunit ...

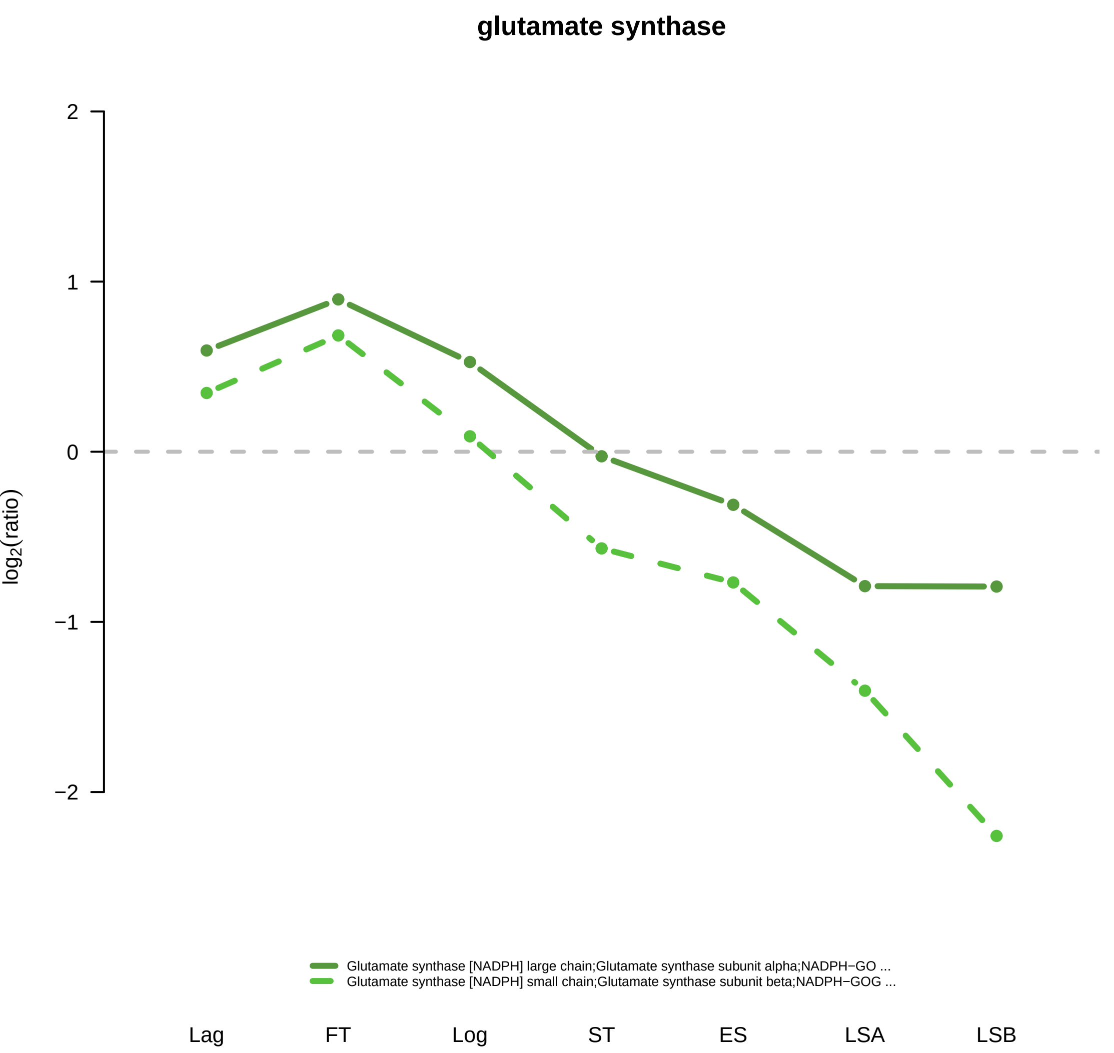

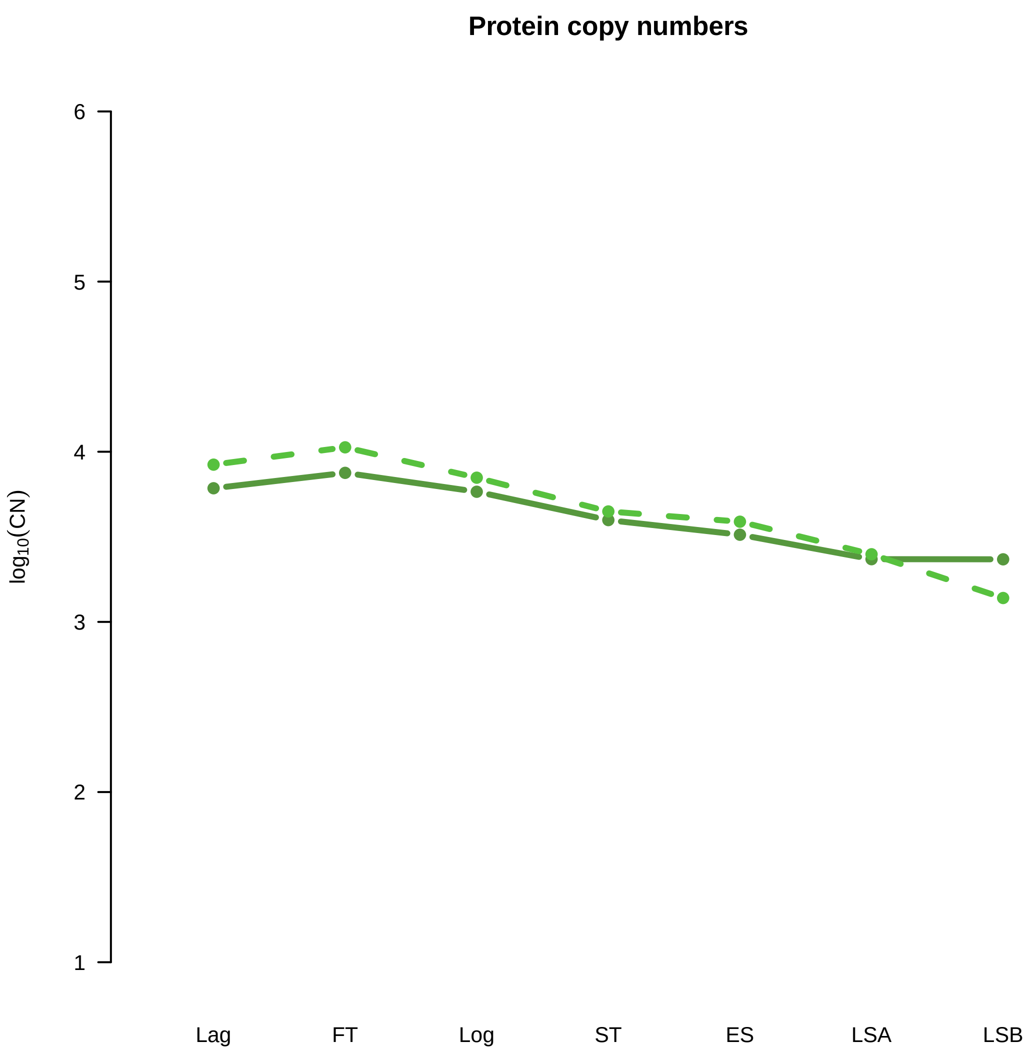

gltB P09831 Glutamate synthase [NADPH] lar ...
gltD P09832 Glutamate synthase [NADPH] sma ...

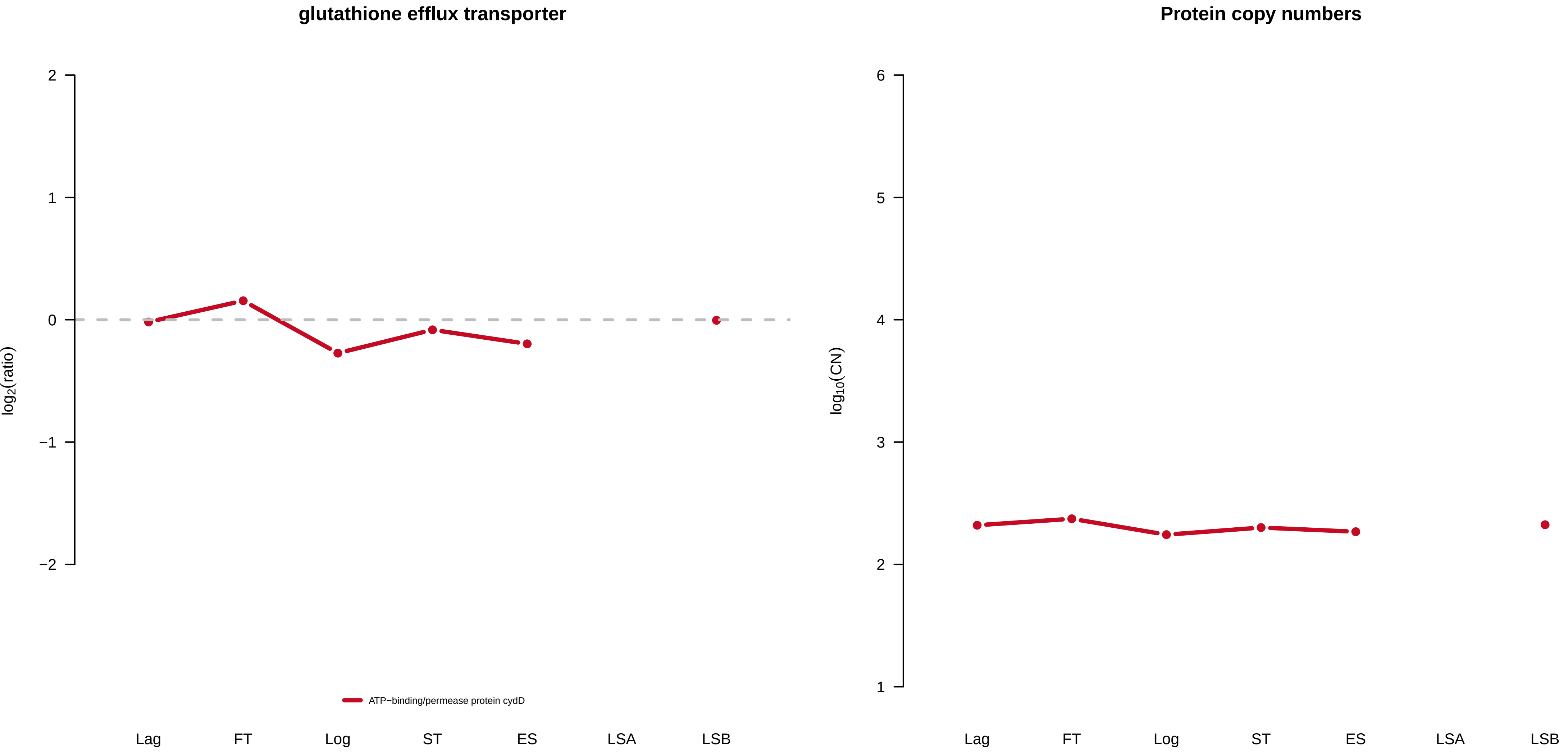

cydC	P23886	ATP−binding/permease protein c ...
cydD	P29018	ATP−binding/permease protein c ...

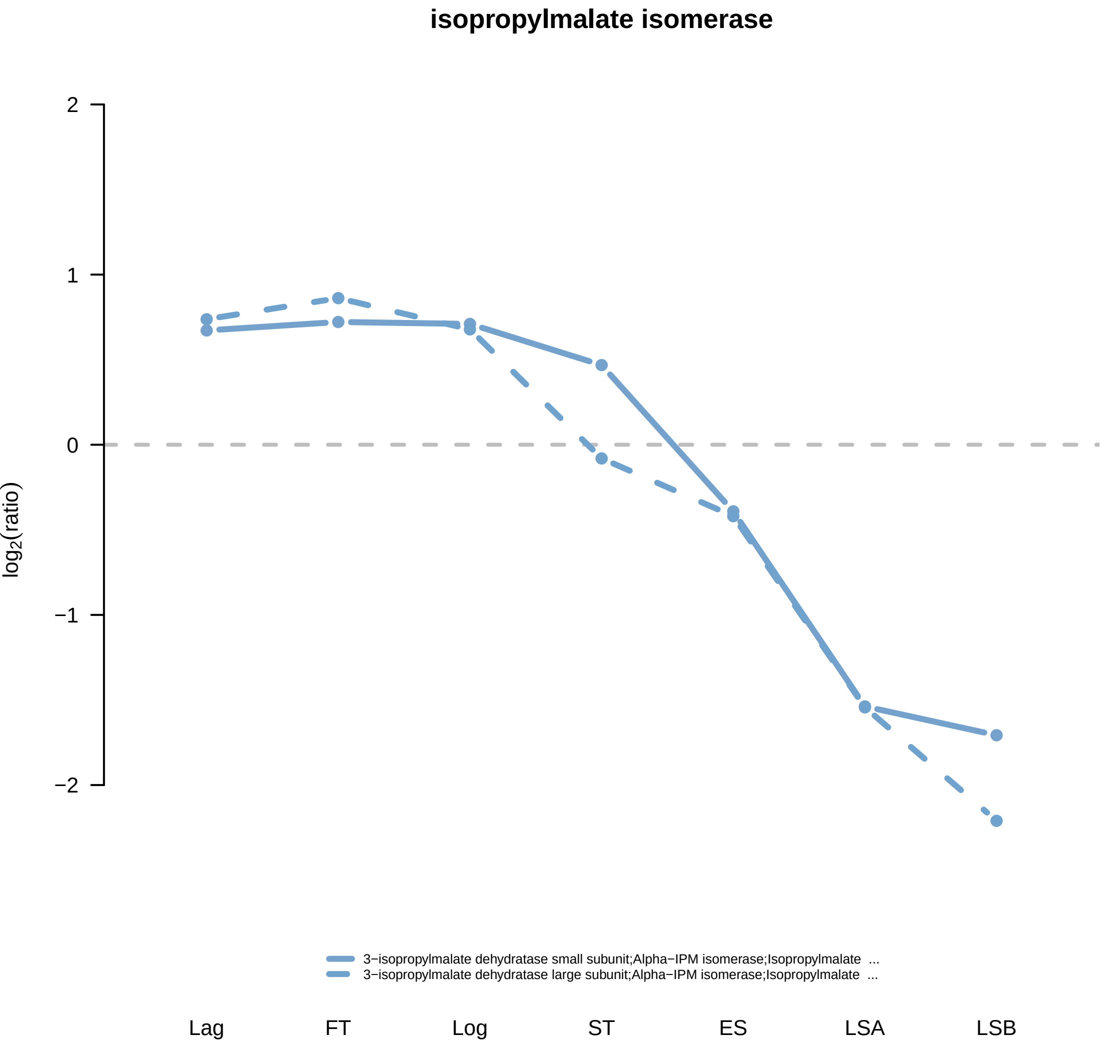

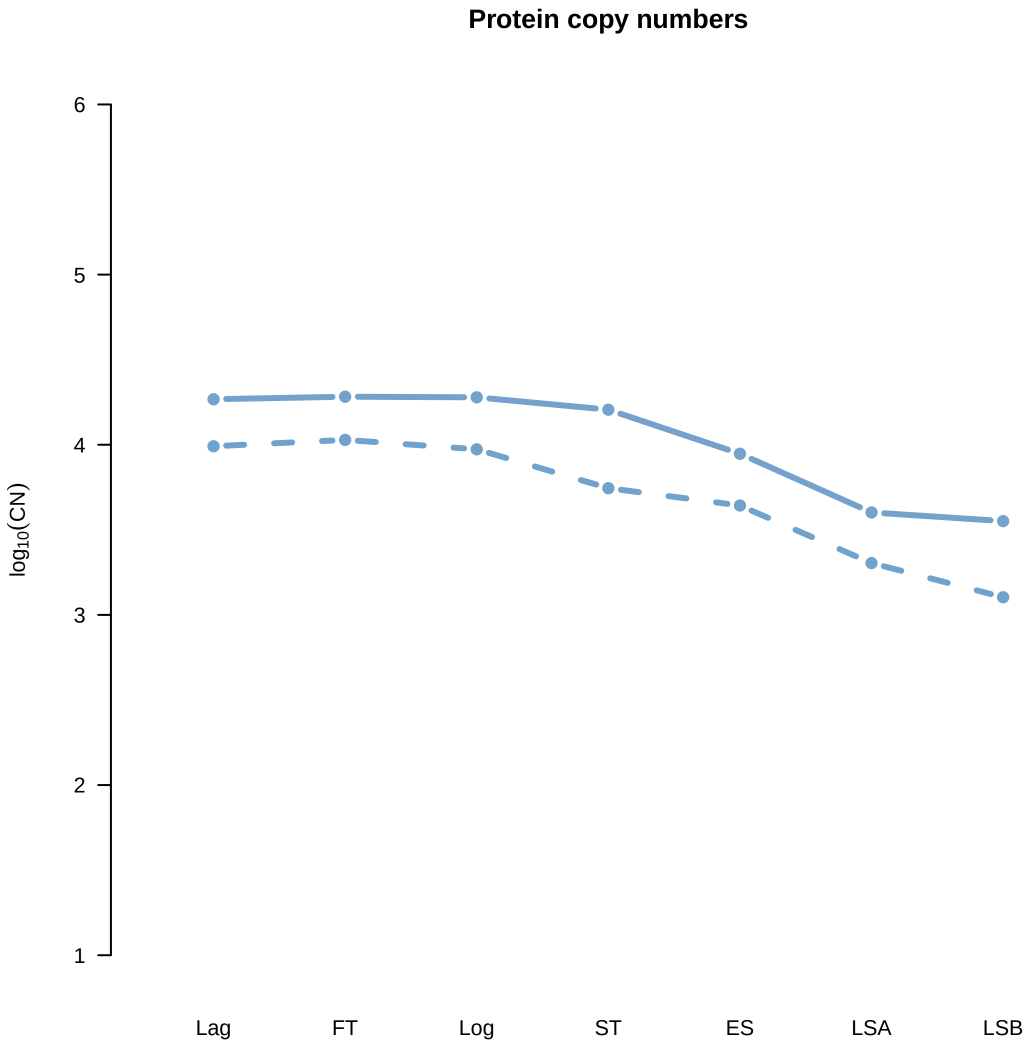

leuD	P30126	3−isopropylmalate dehydratase ...
leuC	P0A6A6	3−isopropylmalate dehydratase ...

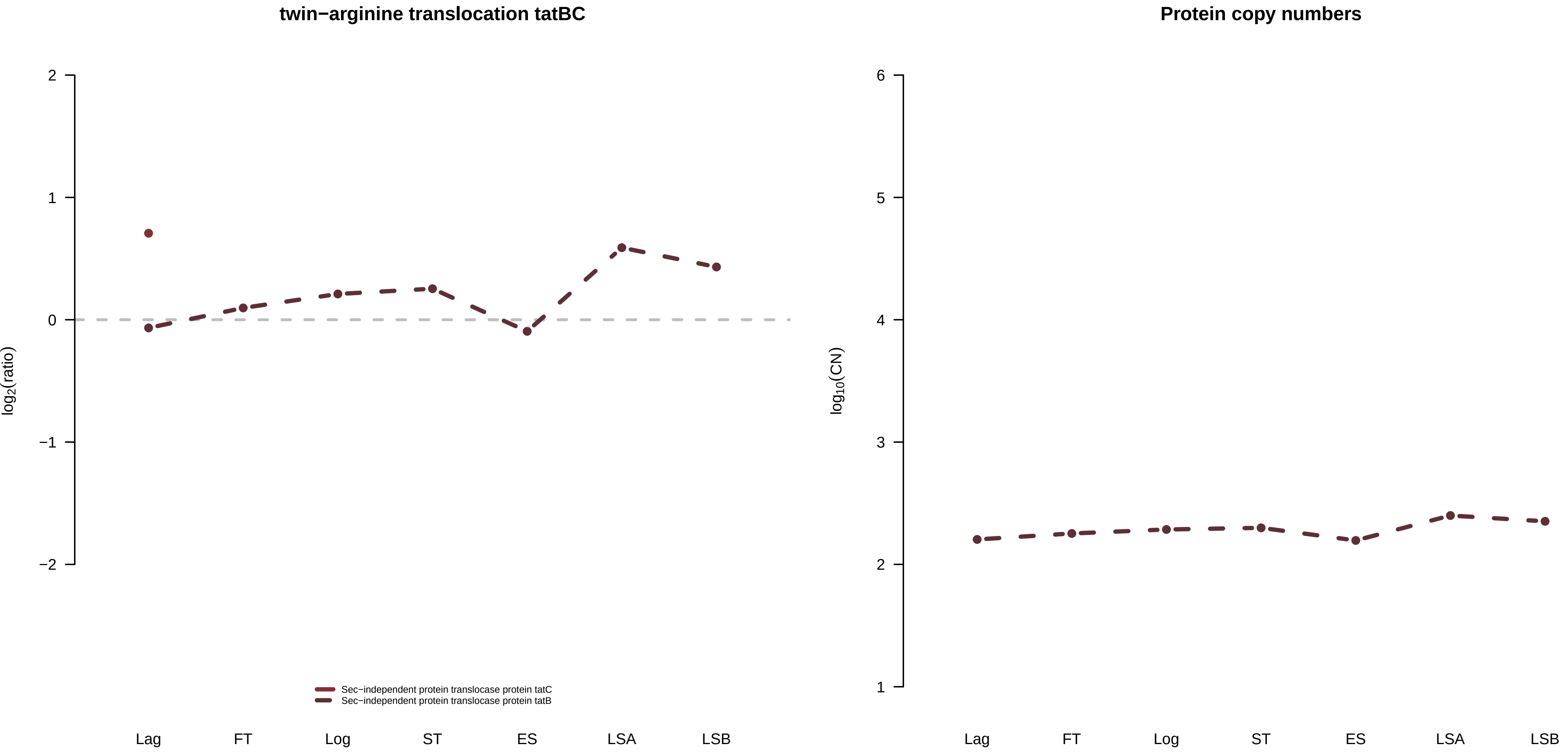

tatC P69423 Sec−independent protein transl ...
tatB P69425 Sec−independent protein transl ...

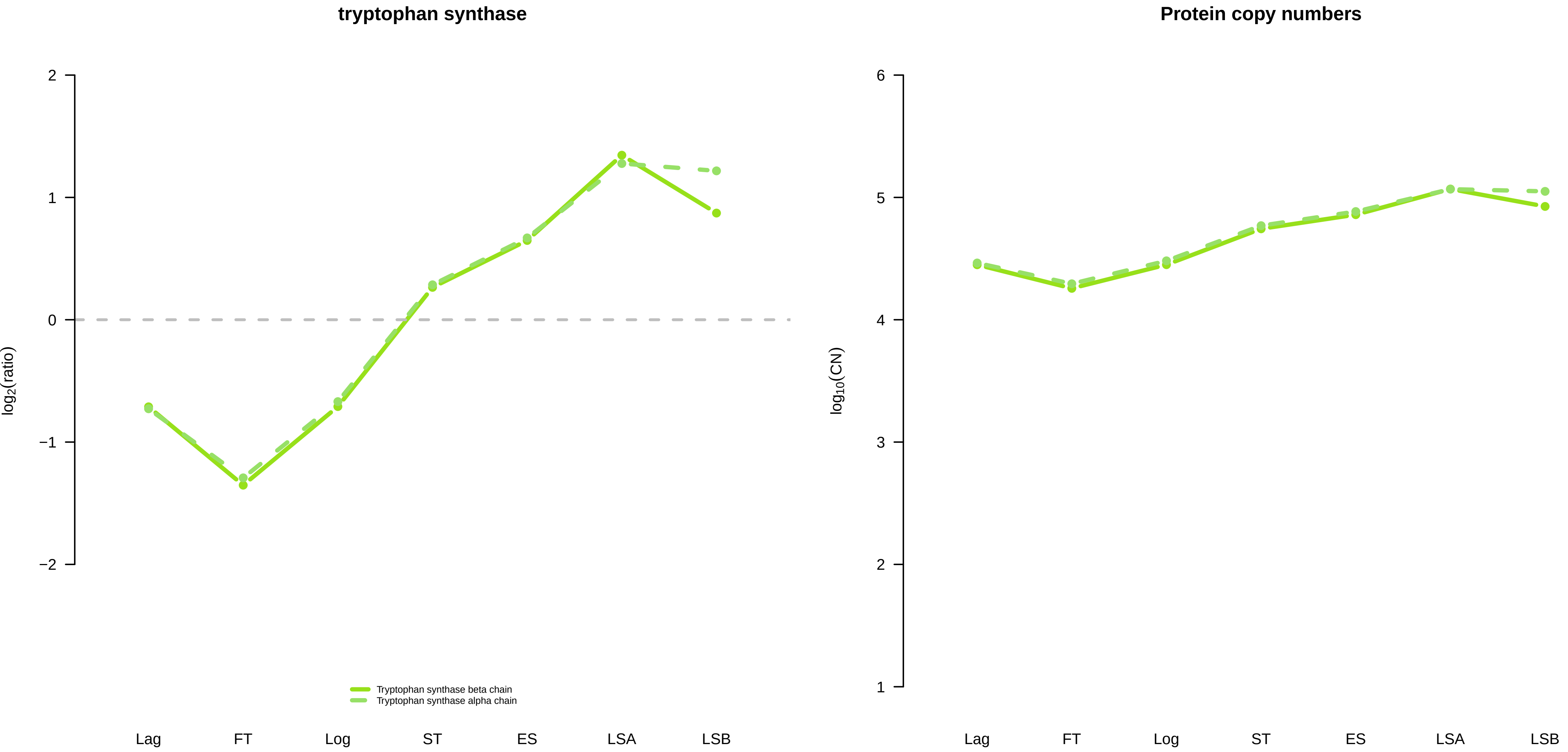

trpB	P0A879	Tryptophan synthase beta chain
trpA	P0A877	Tryptophan synthase alpha chai ...

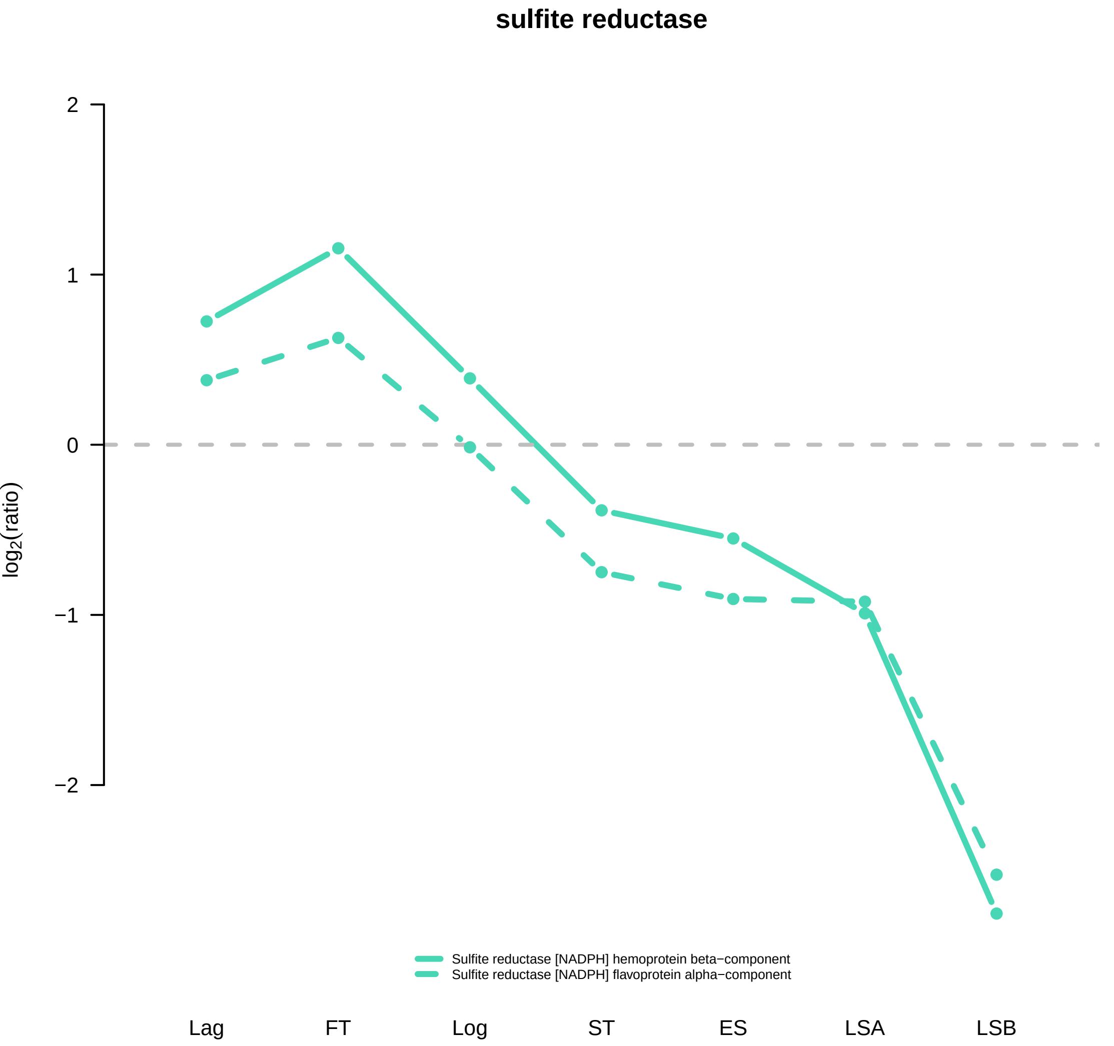

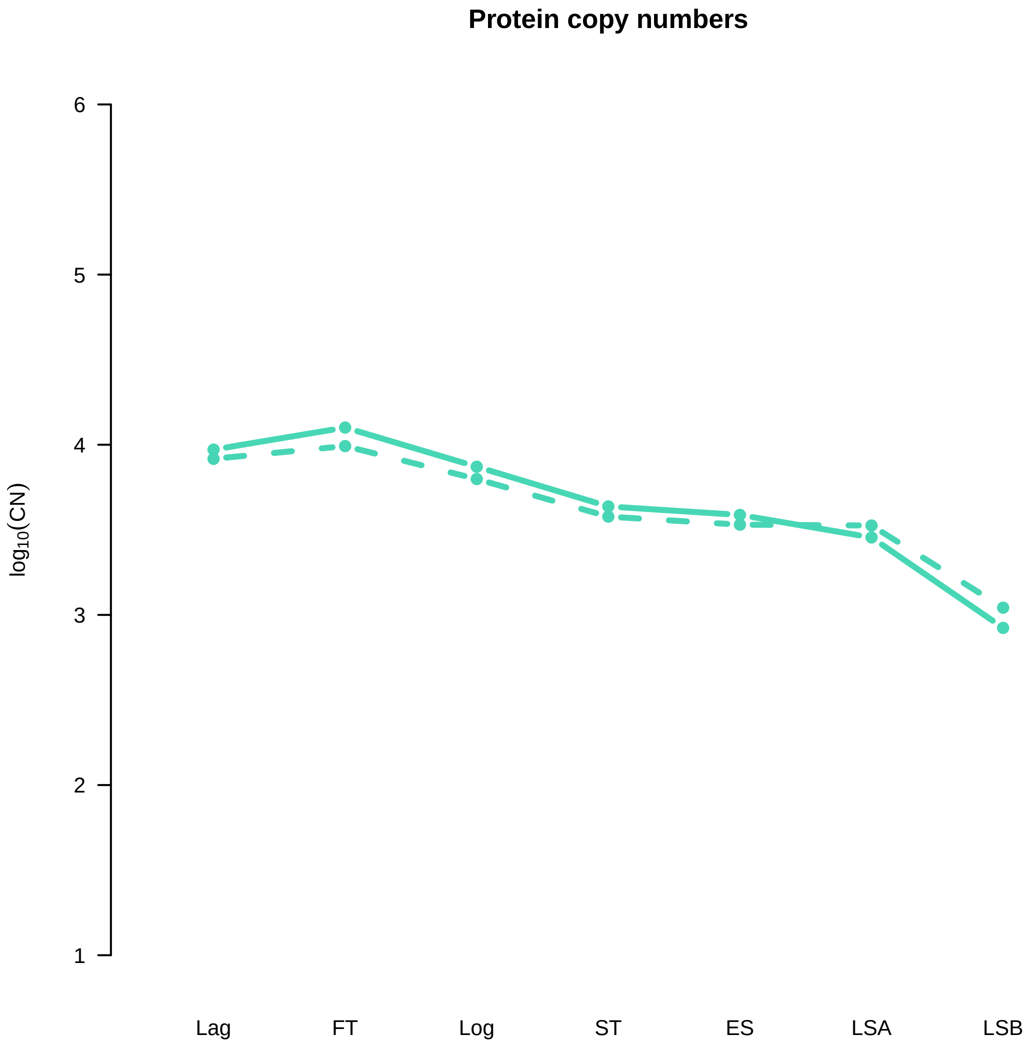

cysI	P17846	Sulfite reductase [NADPH] hemo ...
cysJ	P38038	Sulfite reductase [NADPH] flav ...

Fe−S cluster scaffold

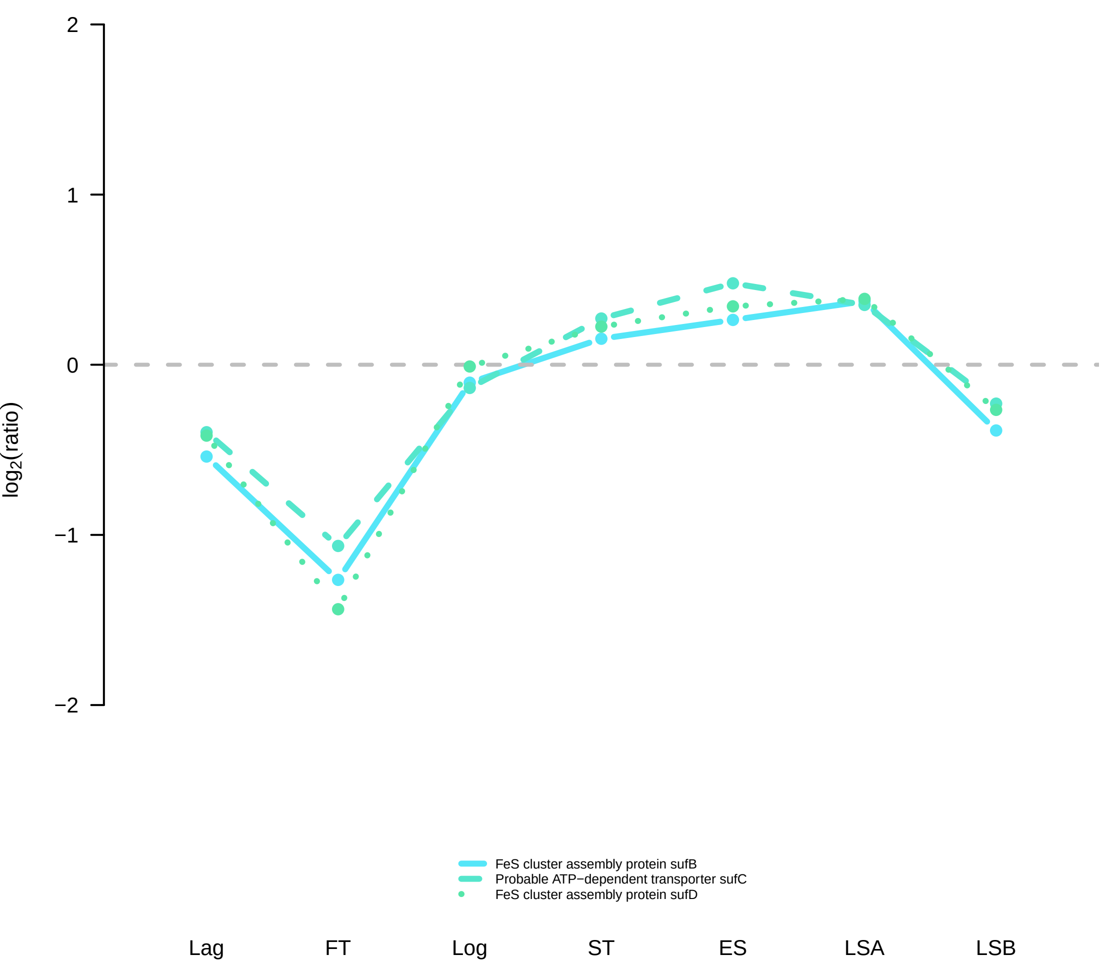

Protein copy numbers

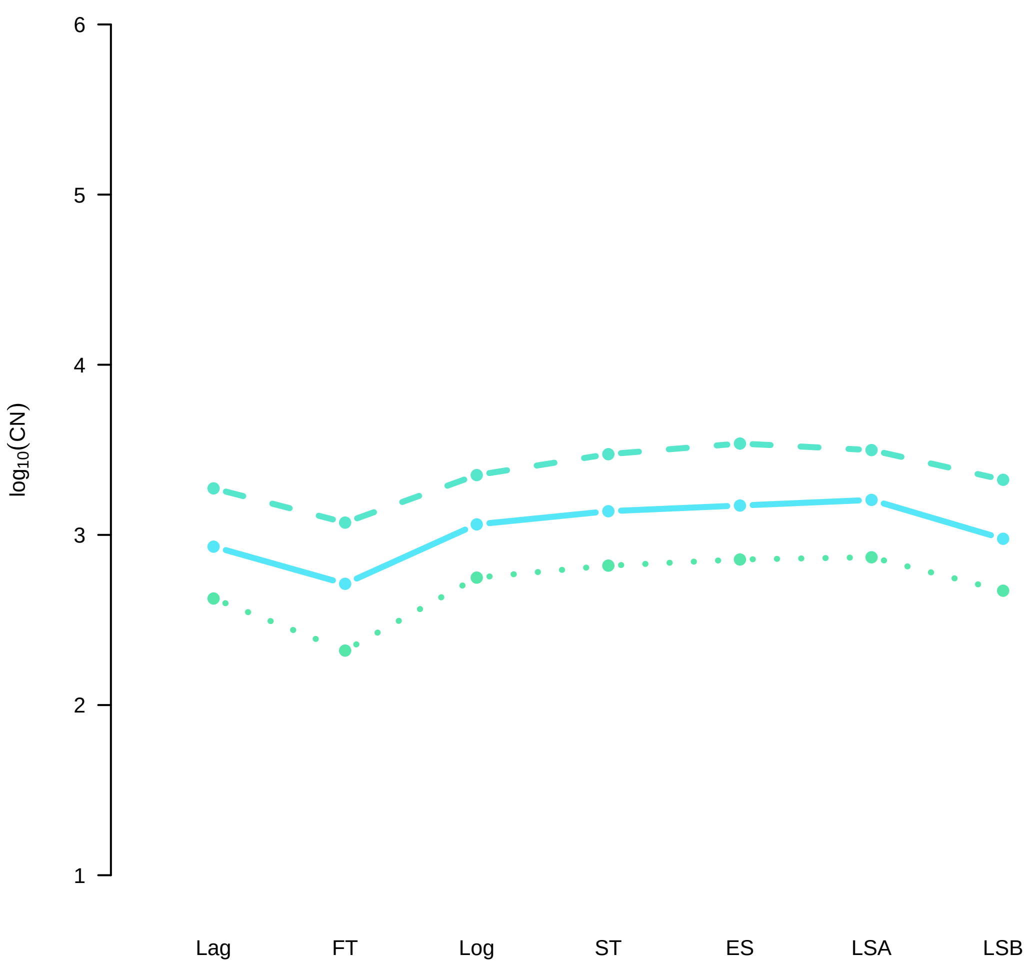

sufB	P77522	FeS cluster assembly protein s ...
sufC	P77499	Probable ATP−dependent transpo ...
sufD	P77689	FeS cluster assembly protein s ...

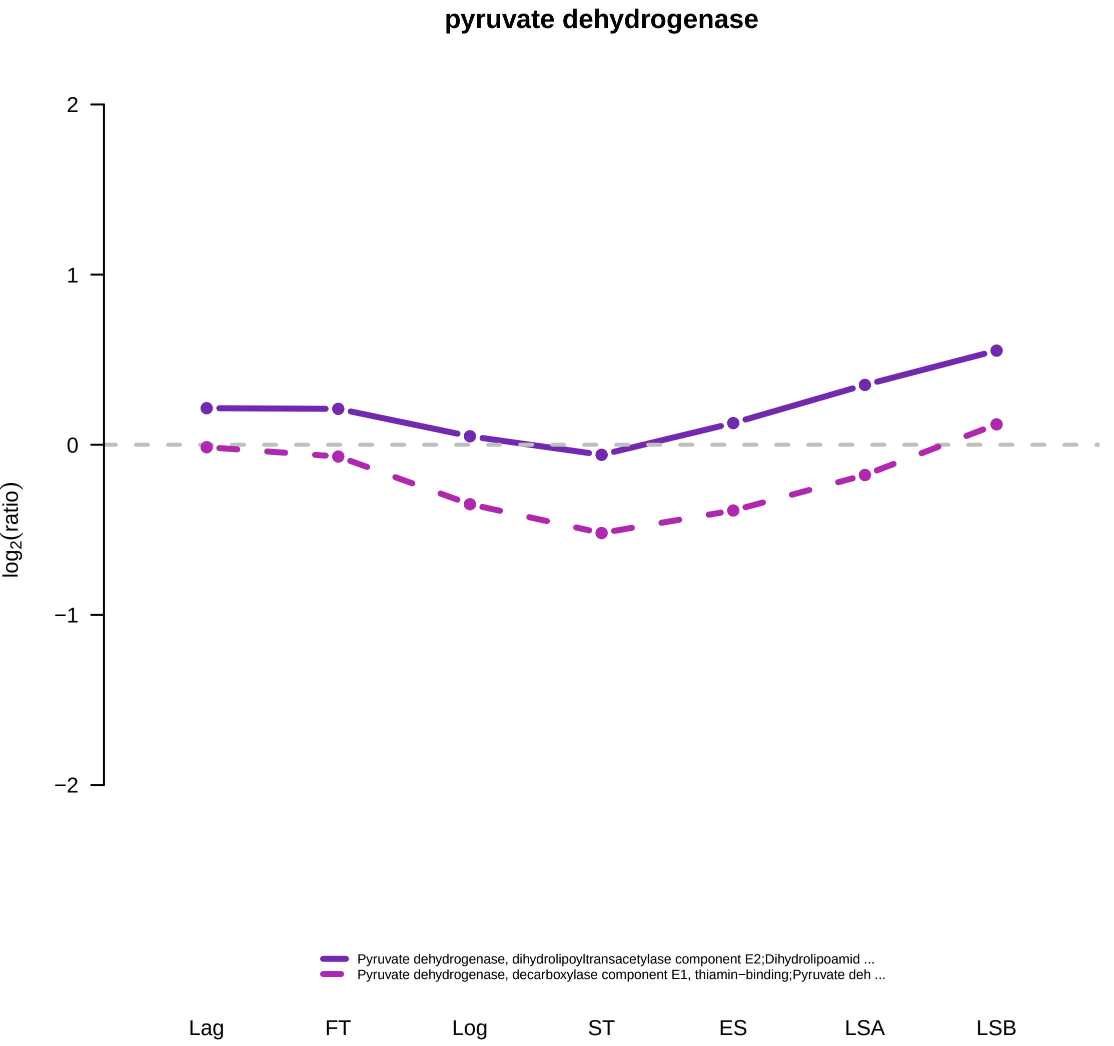

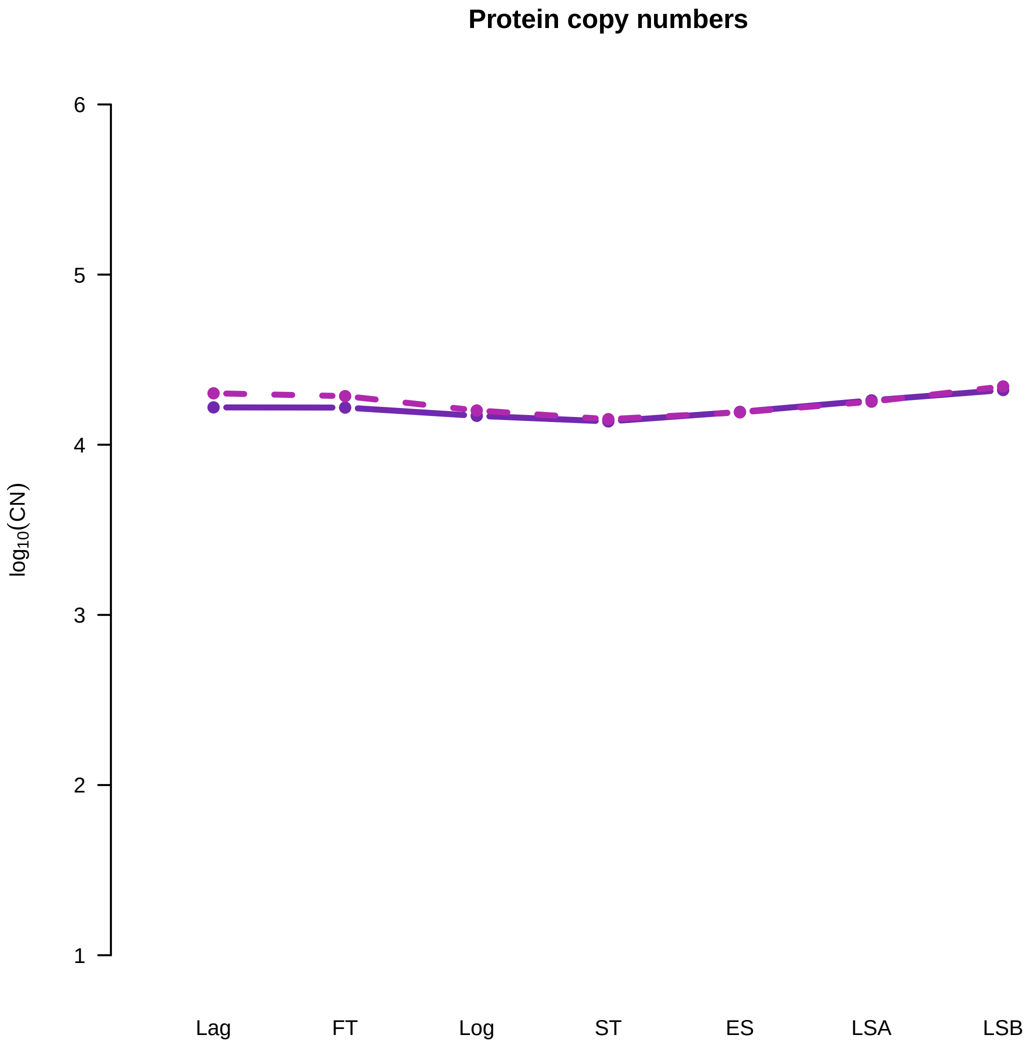

aceF	B1XC90;C4ZRK8;P06959	Pyruvate dehydrogenase, dihydr ...
aceE	B1XC89;C4ZRK7;P0AFG8	Pyruvate dehydrogenase, decarb ...

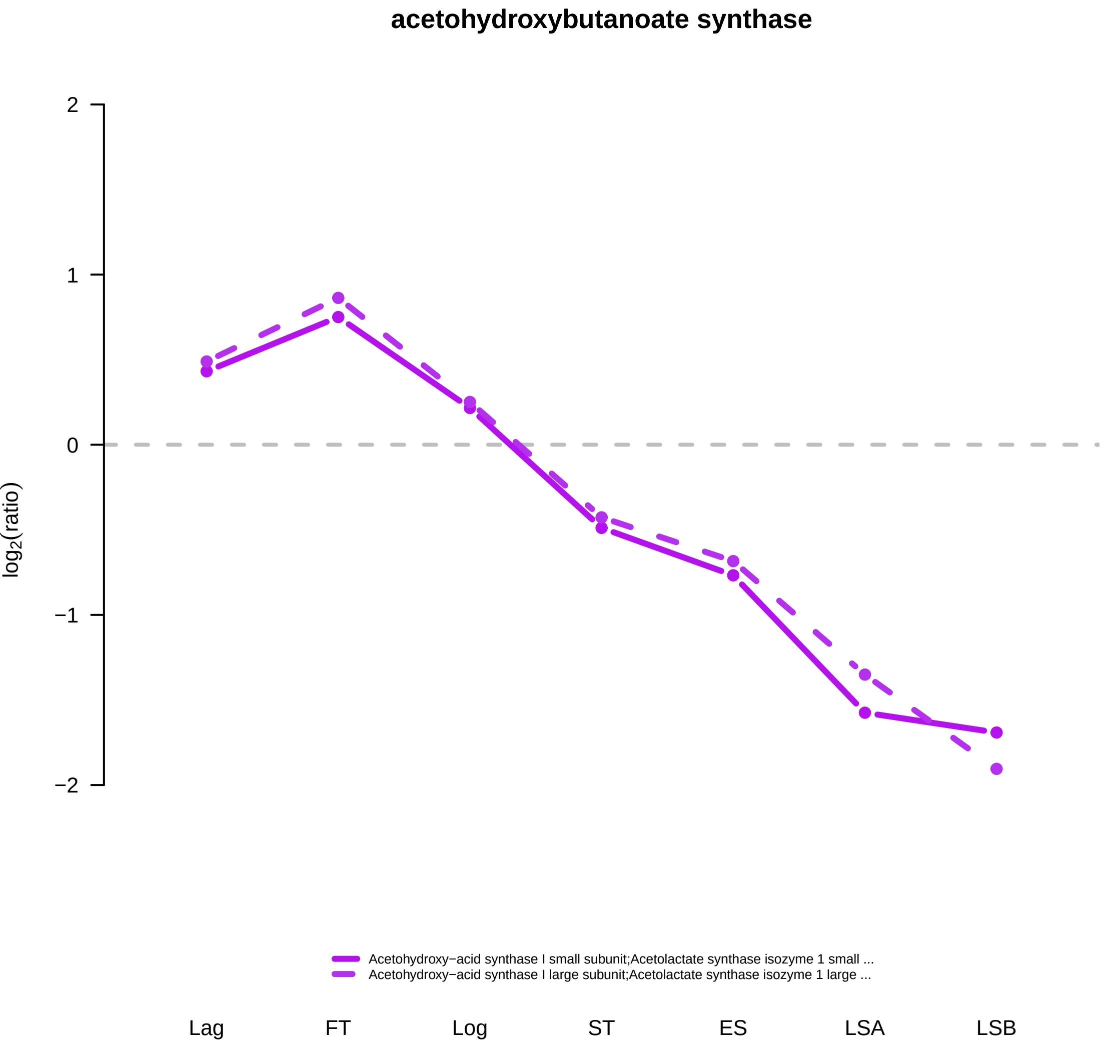

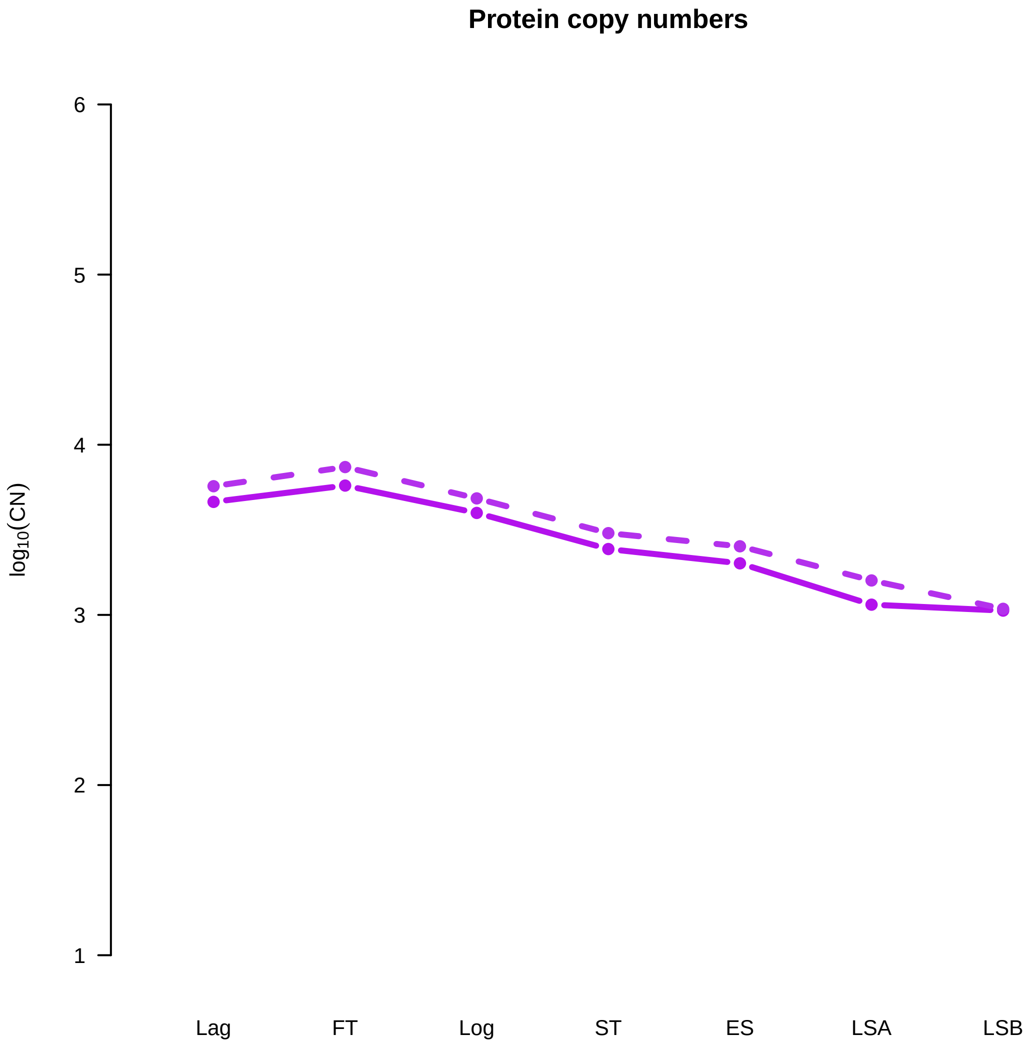

ilvN	P0ADF8	Acetohydroxy−acid synthase I s ...
ilvB	P08142	Acetohydroxy−acid synthase I l ...

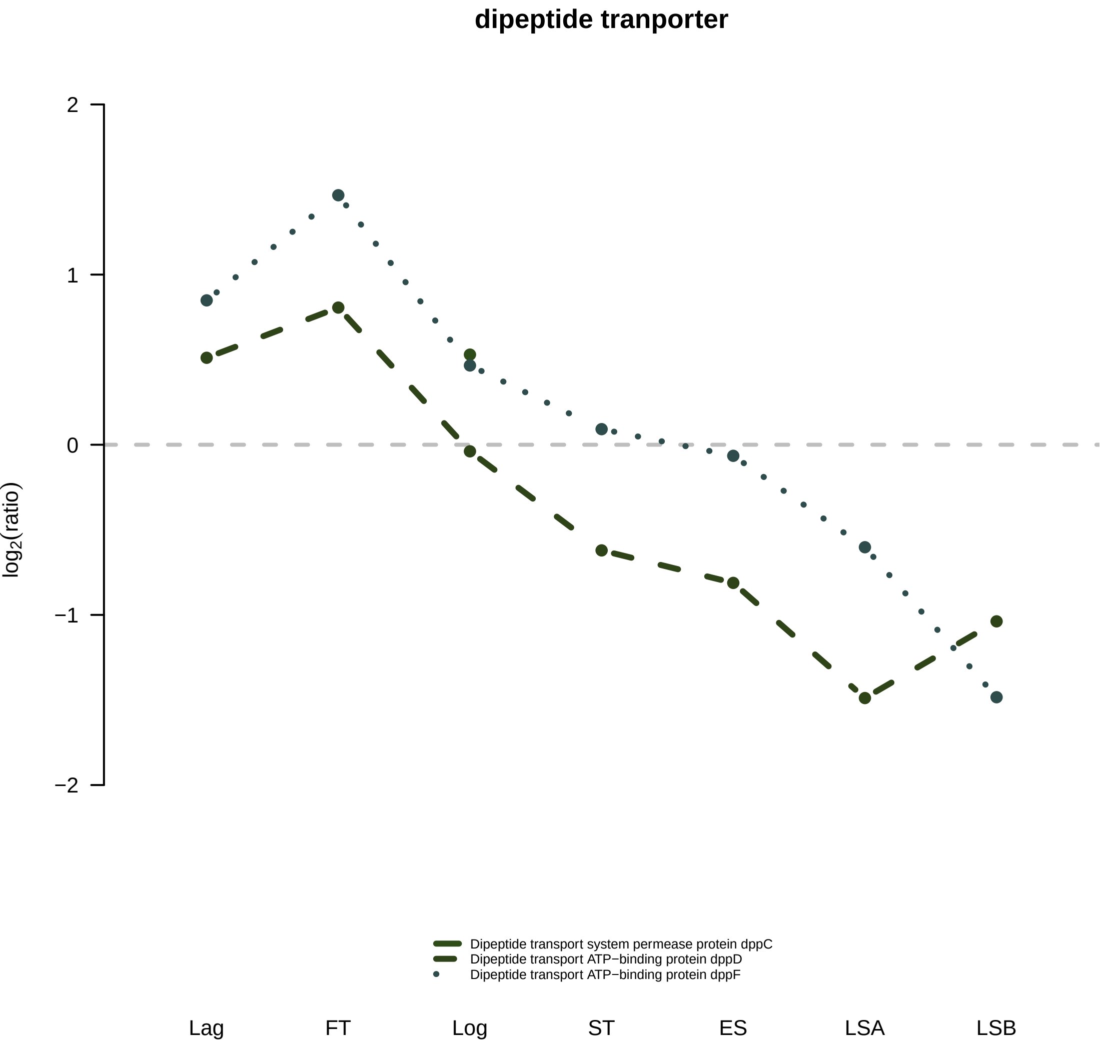

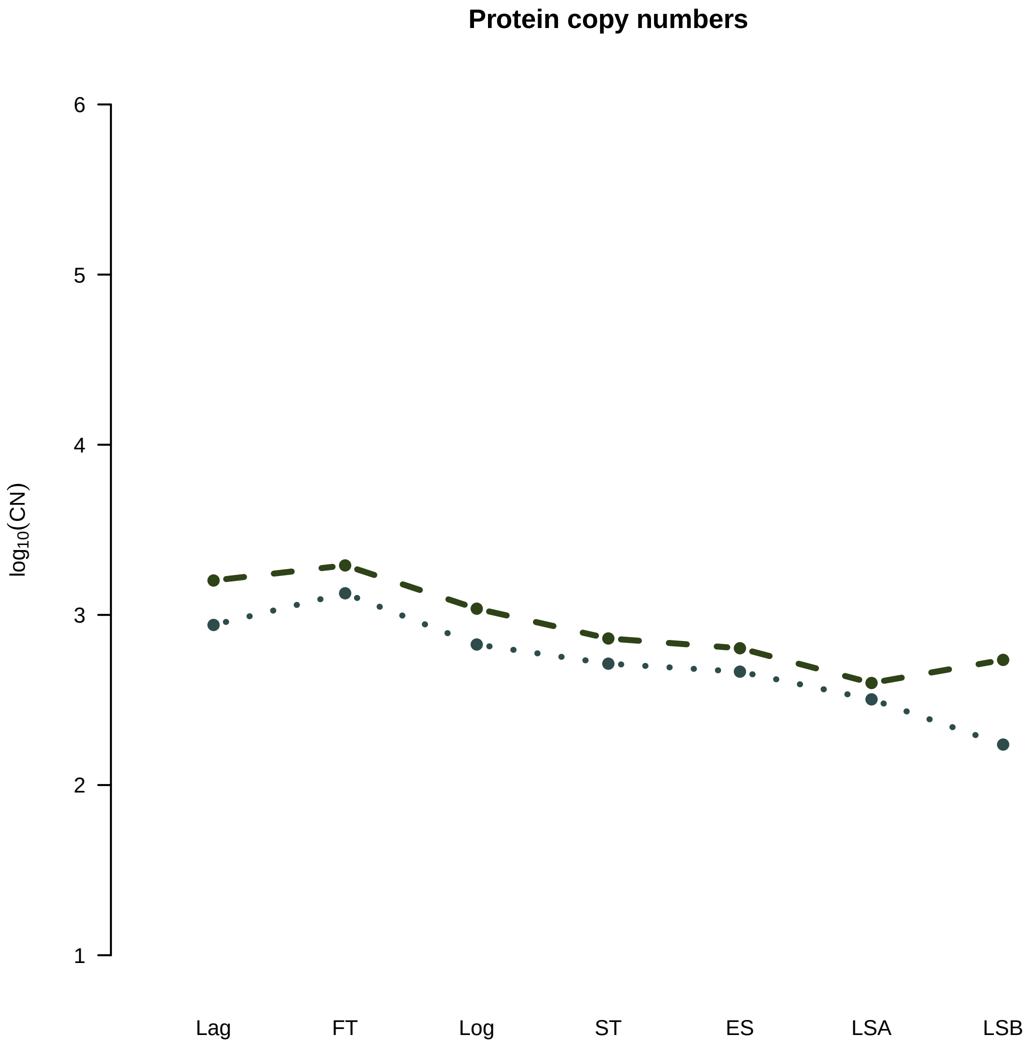

dppB	−	−
dppC	P0AEG1	Dipeptide transport system per ...
dppD	P0AAG0	Dipeptide transport ATP−bindin ...
dppF	P37313	Dipeptide transport ATP−bindin ...

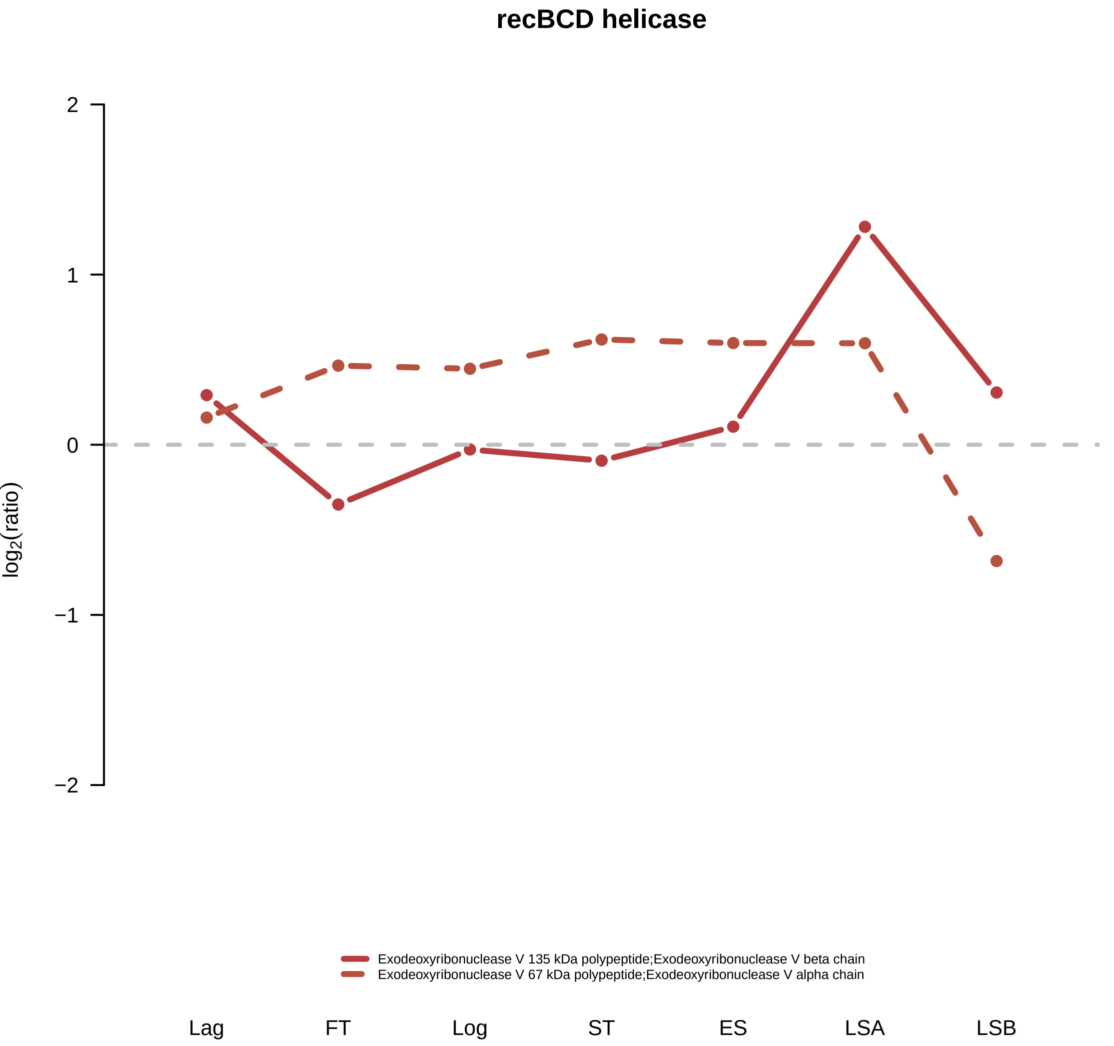

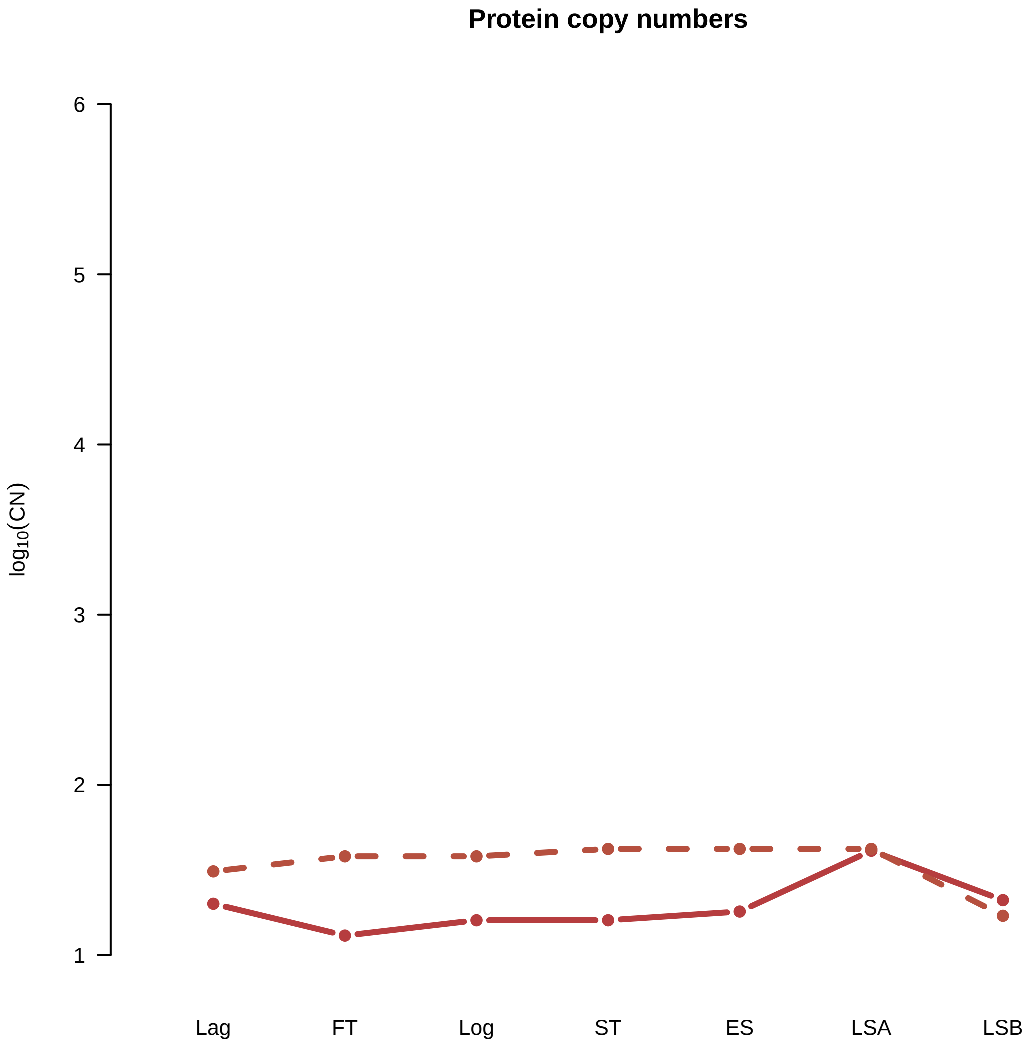

recB	P08394	Exodeoxyribonuclease V 135 kDa ...
recC	–	–
recD	P04993	Exodeoxyribonuclease V 67 kDa ...

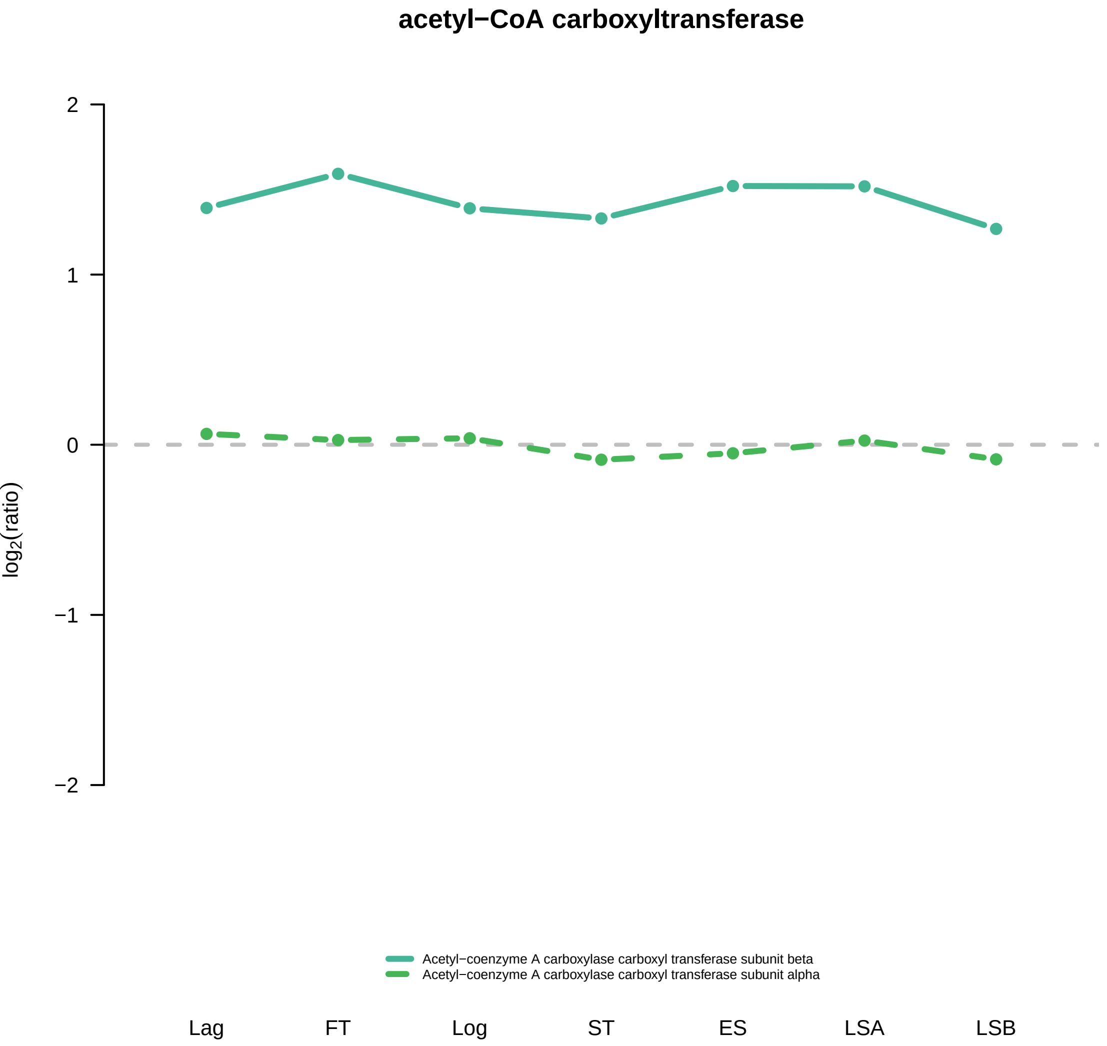

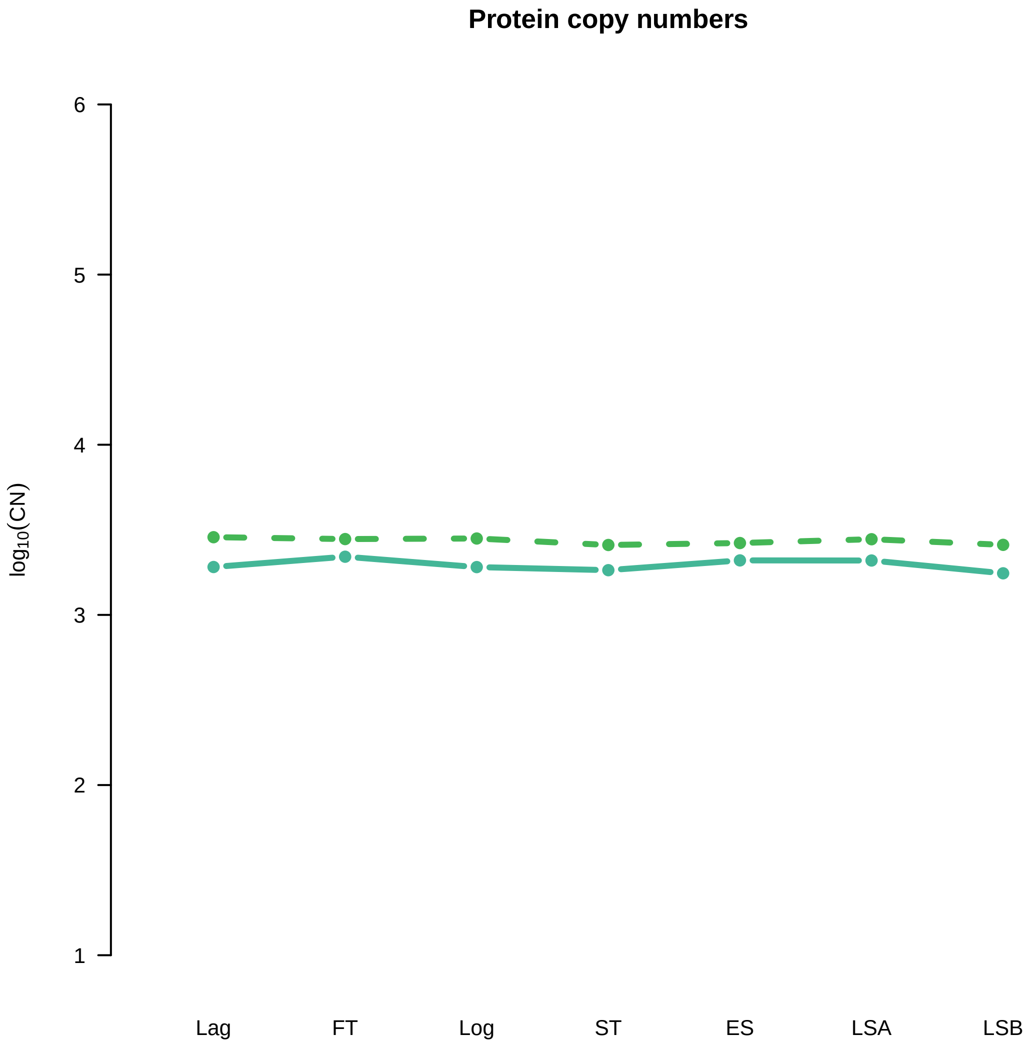

accD	P0A9Q5	Acetyl−coenzyme A carboxylase ...
accA	P0ABD5	Acetyl−coenzyme A carboxylase ...

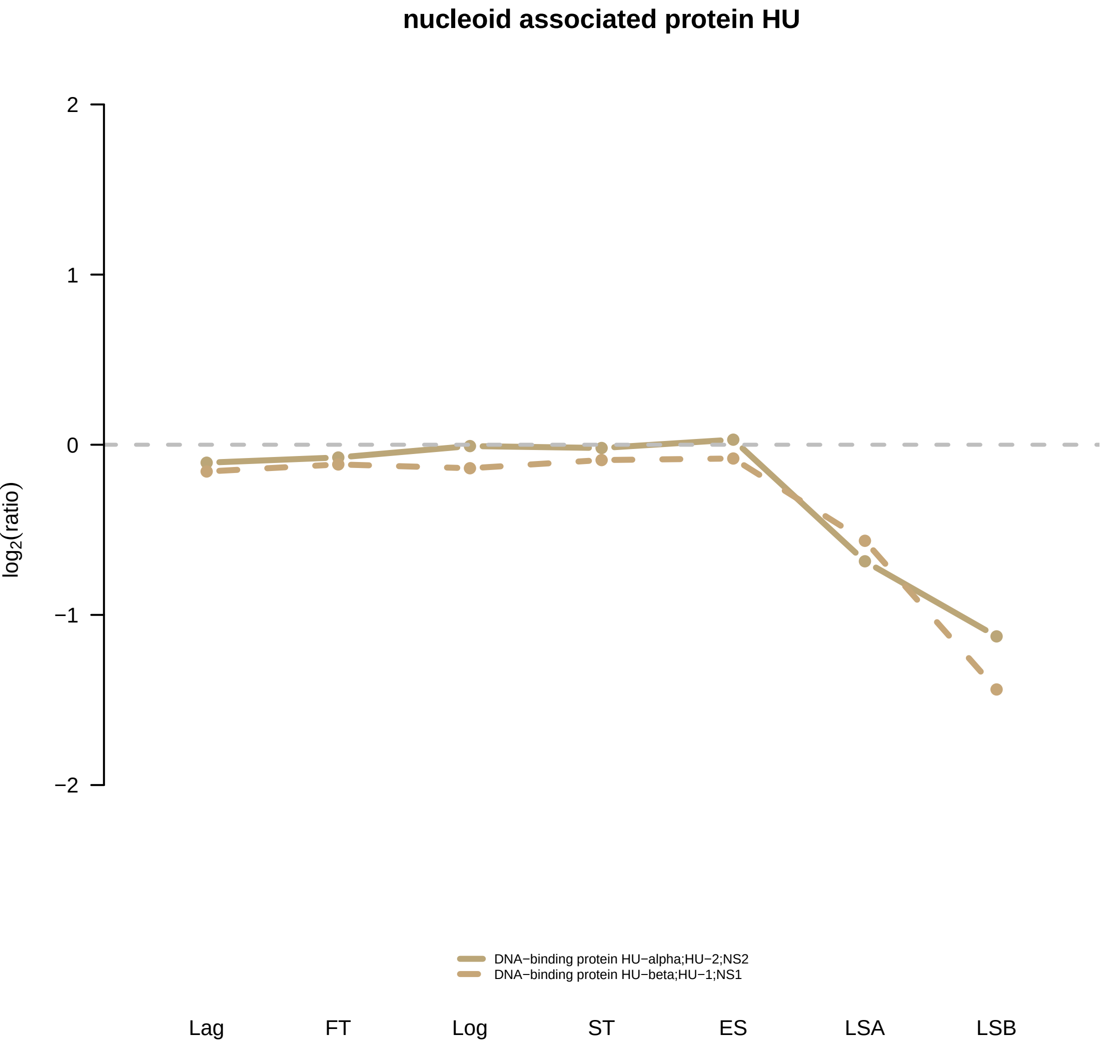

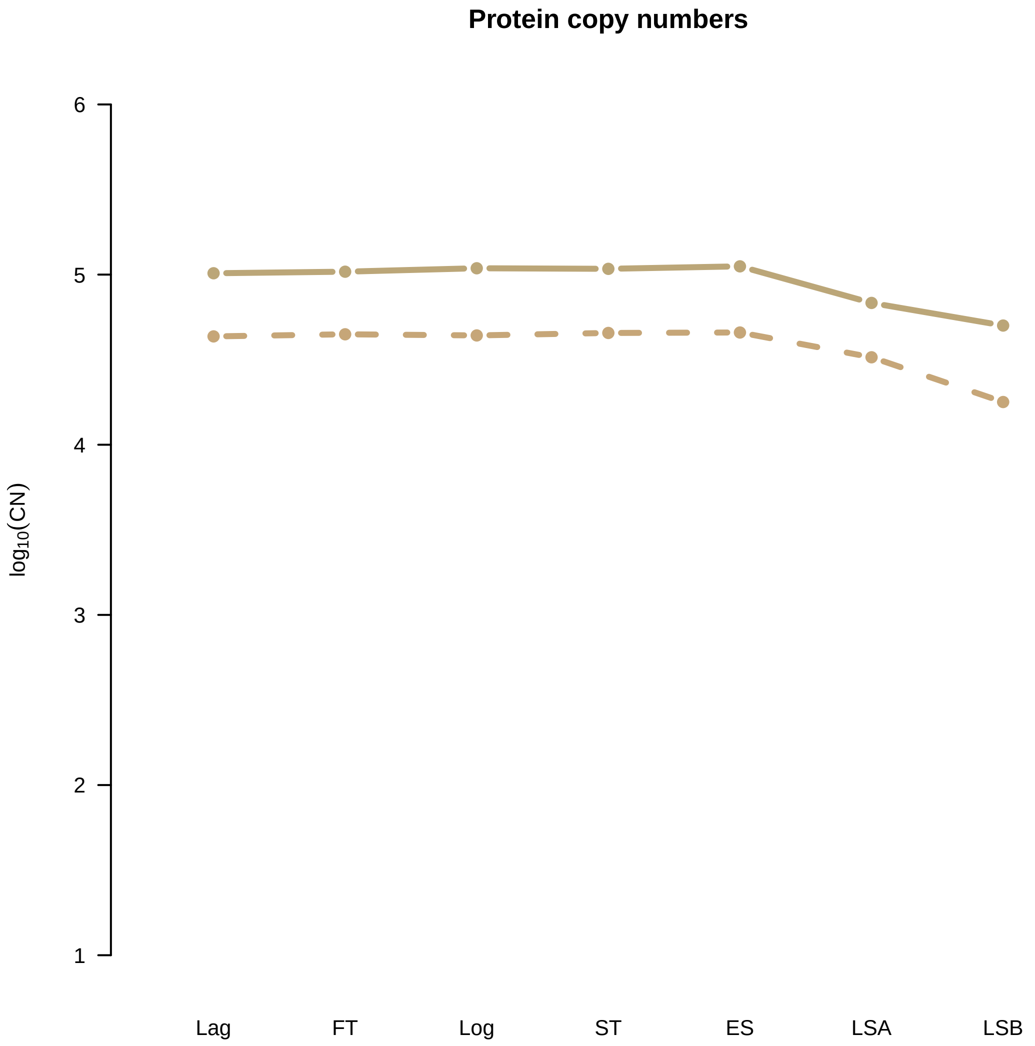

hupA P0ACF0 DNA−binding protein HU−alpha;H ...
hupB P0ACF4 DNA−binding protein HU−beta;HU ...

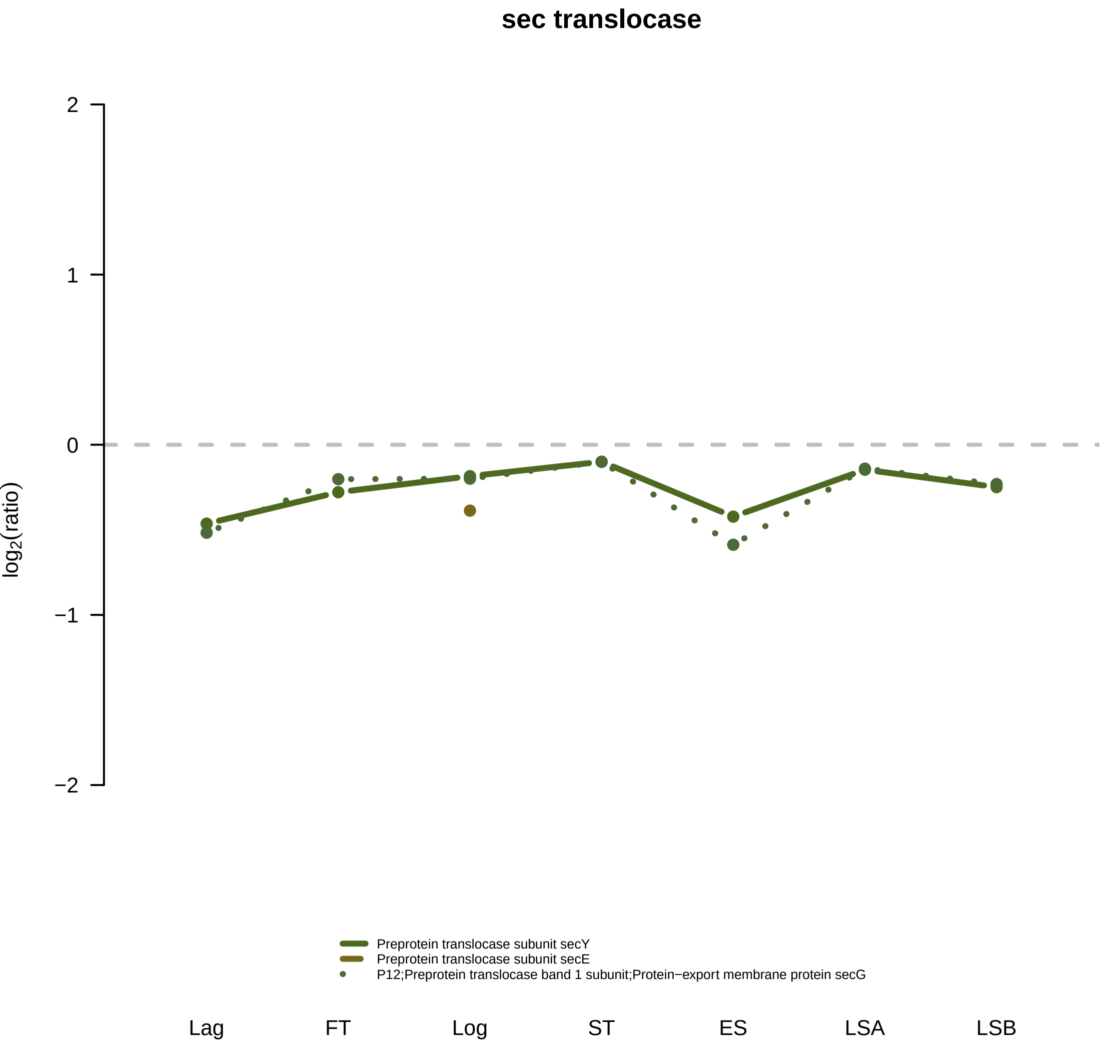

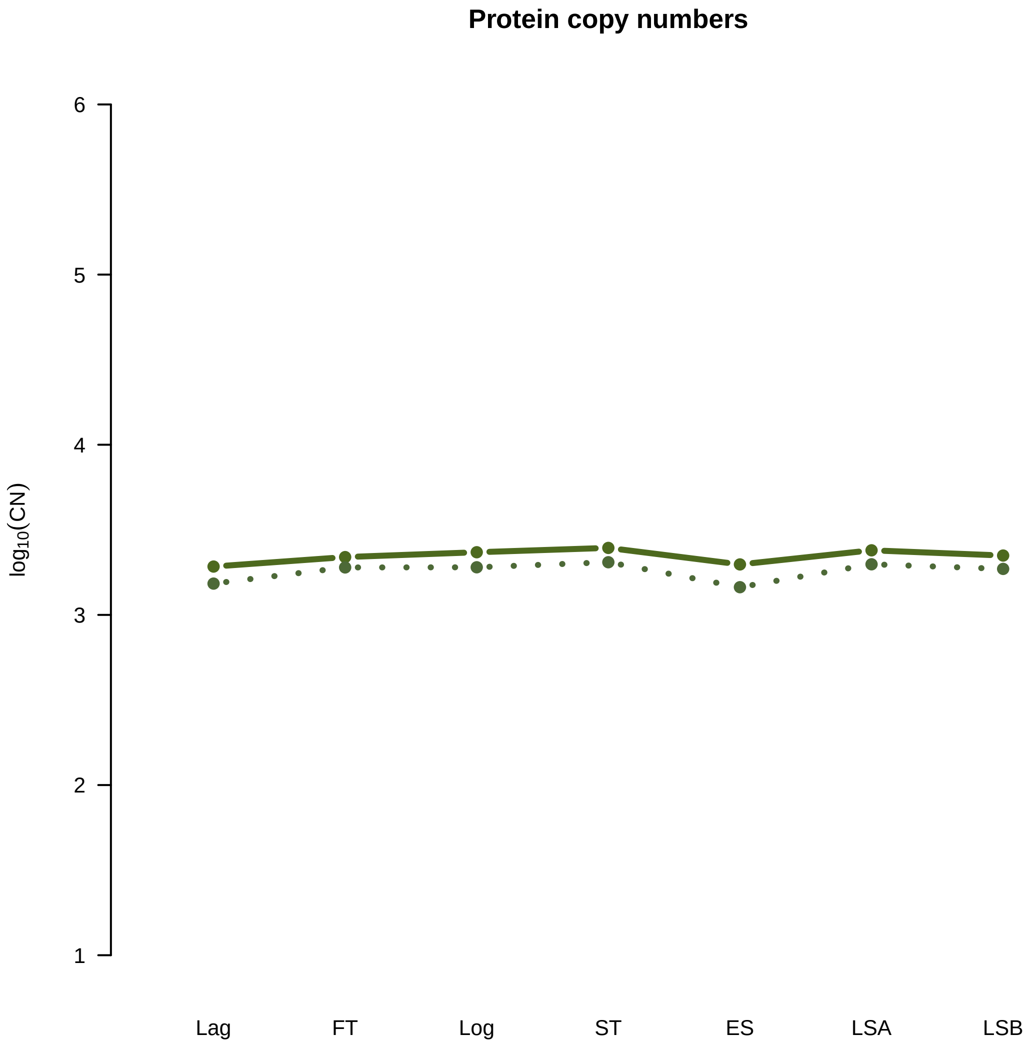

secY	P0AGA2	Preprotein translocase subunit ...
secE	P0AG96	Preprotein translocase subunit ...
secG	P0AG99	P12;Preprotein translocase ban ...

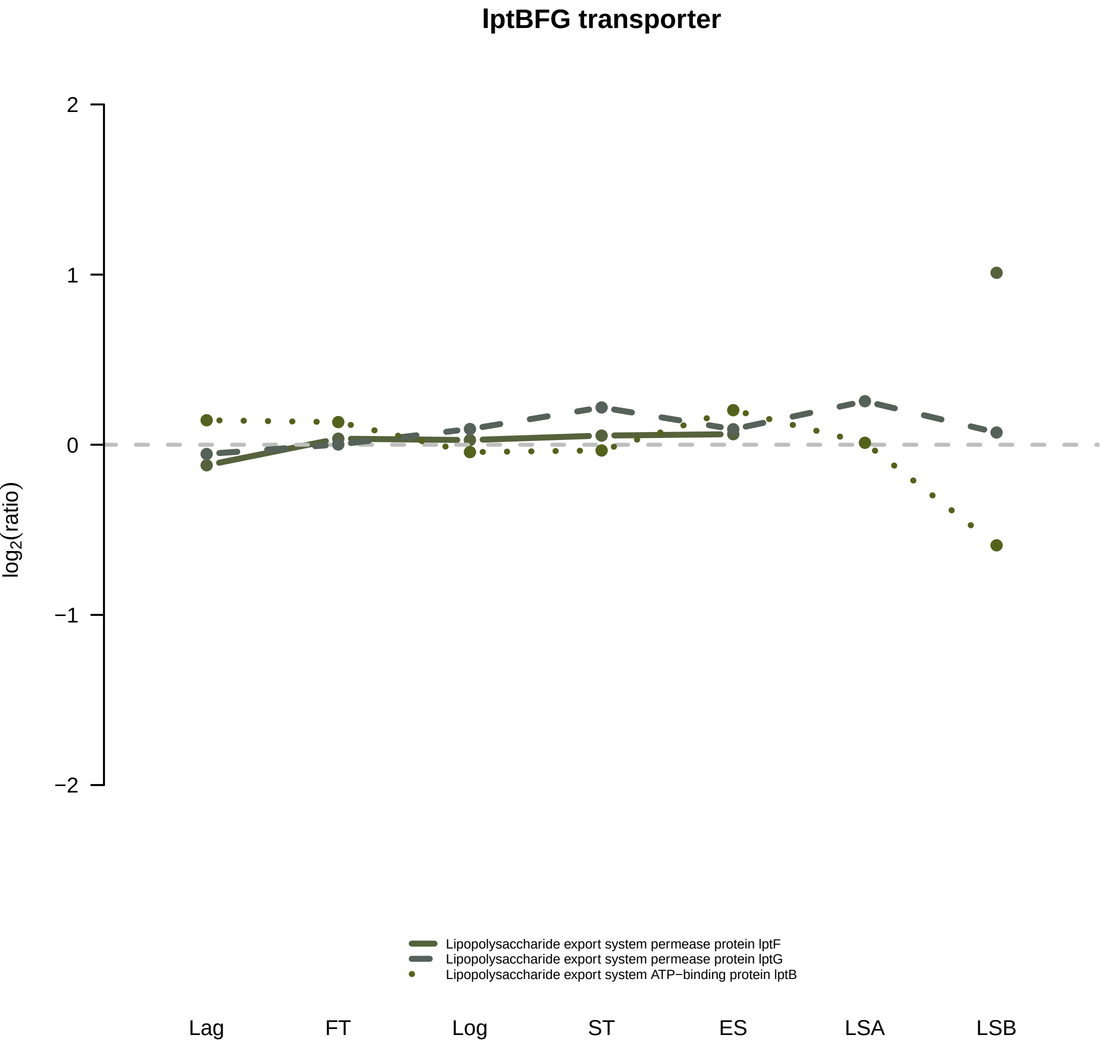

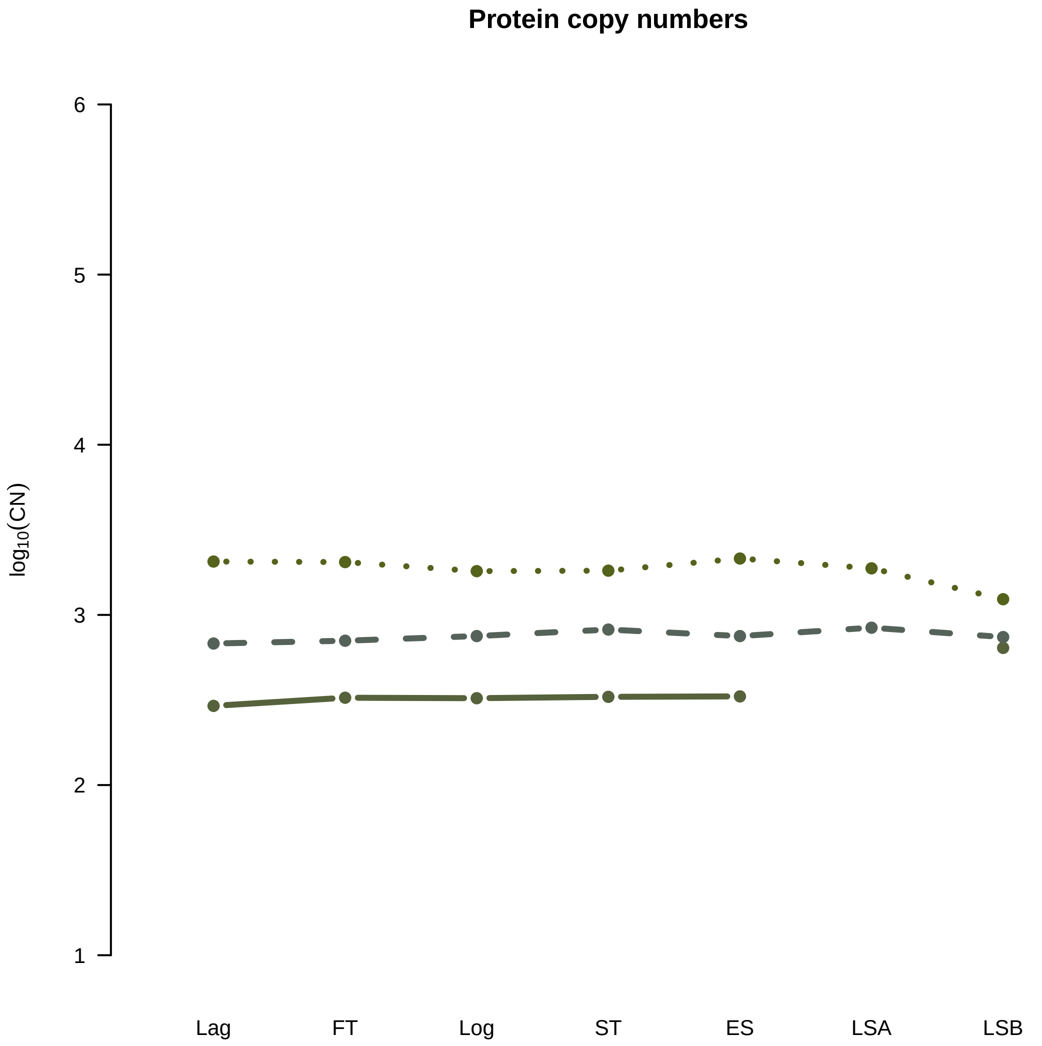

lptF	P0AF98	Lipopolysaccharide export syst ...
lptG	P0ADC6	Lipopolysaccharide export syst ...
lptB	P0A9V1	Lipopolysaccharide export syst ...

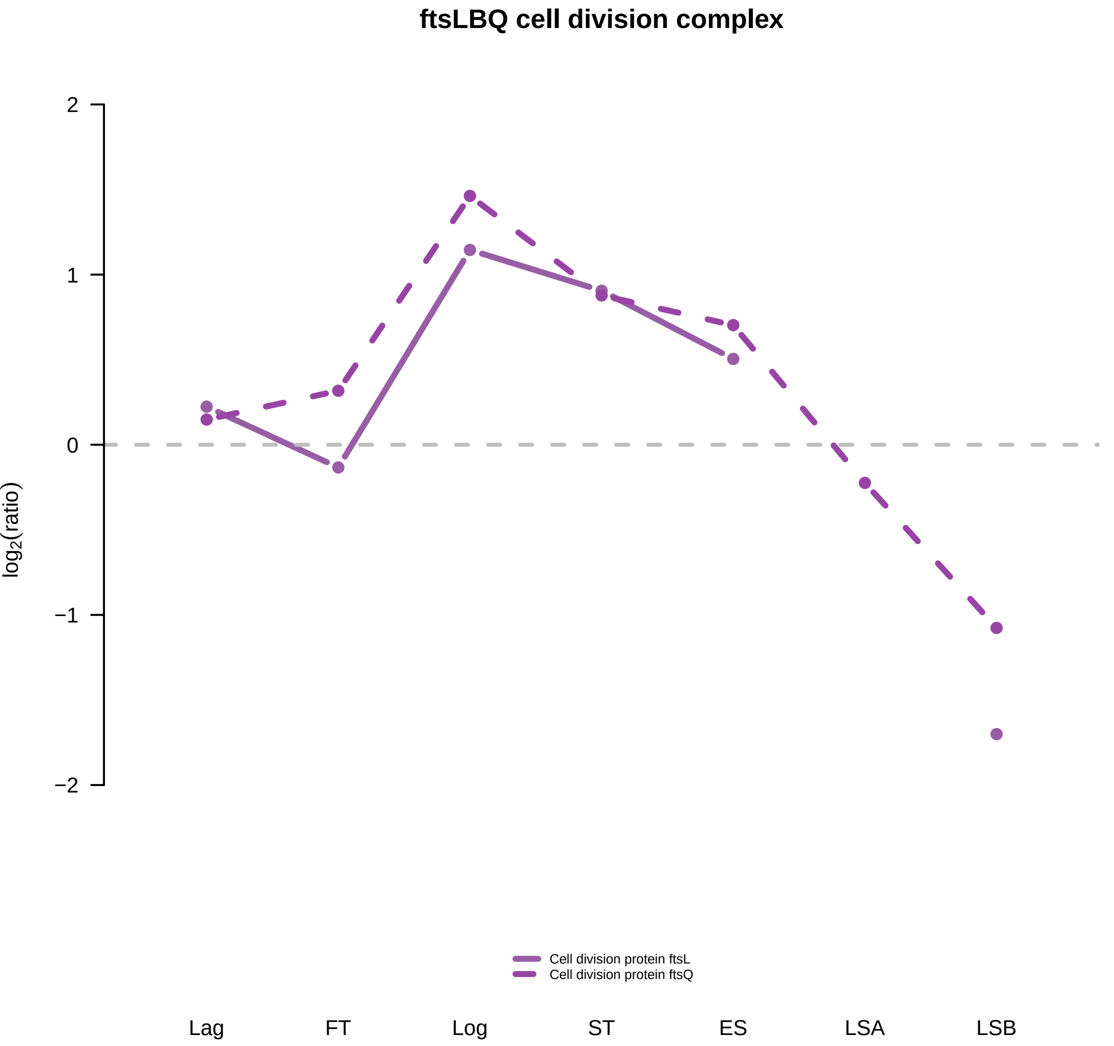

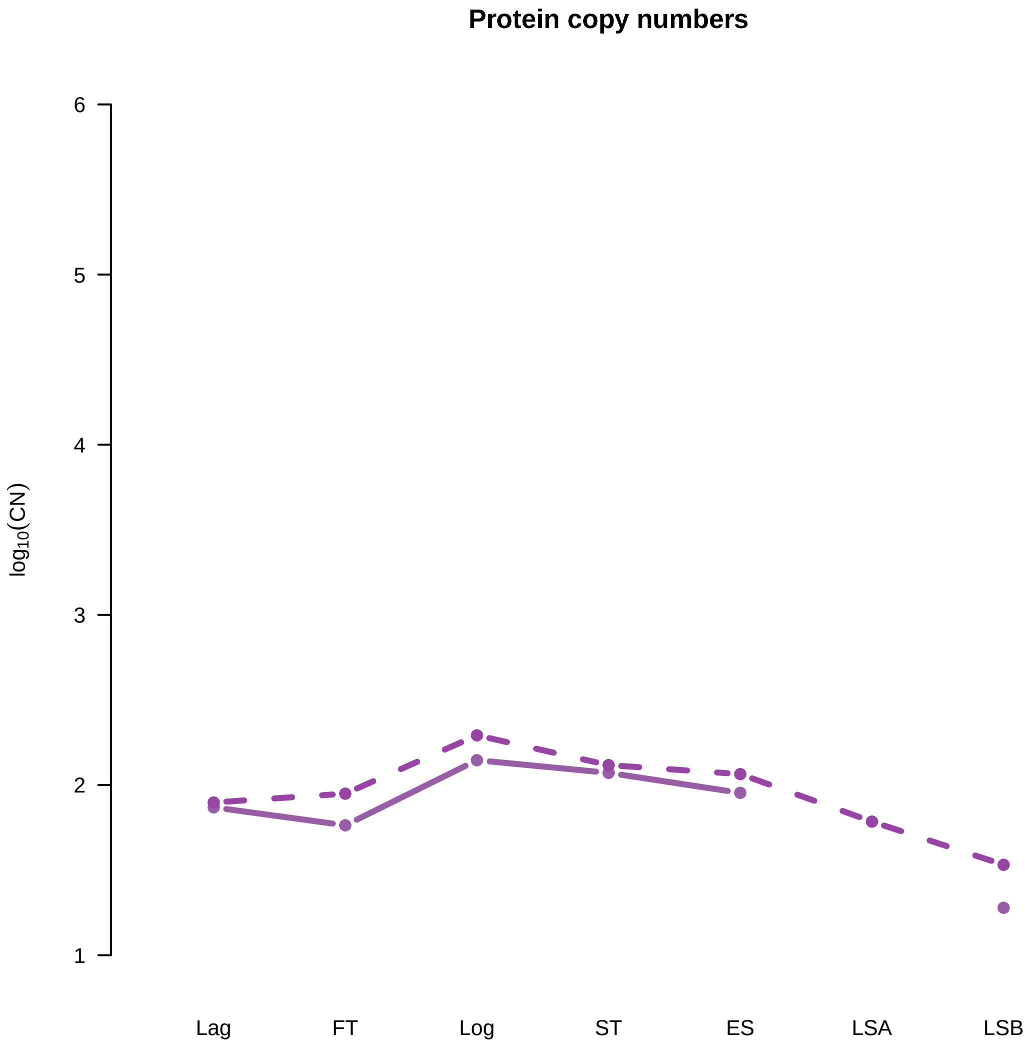

2 hits for ftsB in protein groups table

ftsL	P0AEN4	Cell division protein ftsL
ftsB	P0A6S5 | P69924	Cell division protein ftsB | Protein B2;Protein R2;Ribonucl ...
ftsQ	P06136	Cell division protein ftsQ

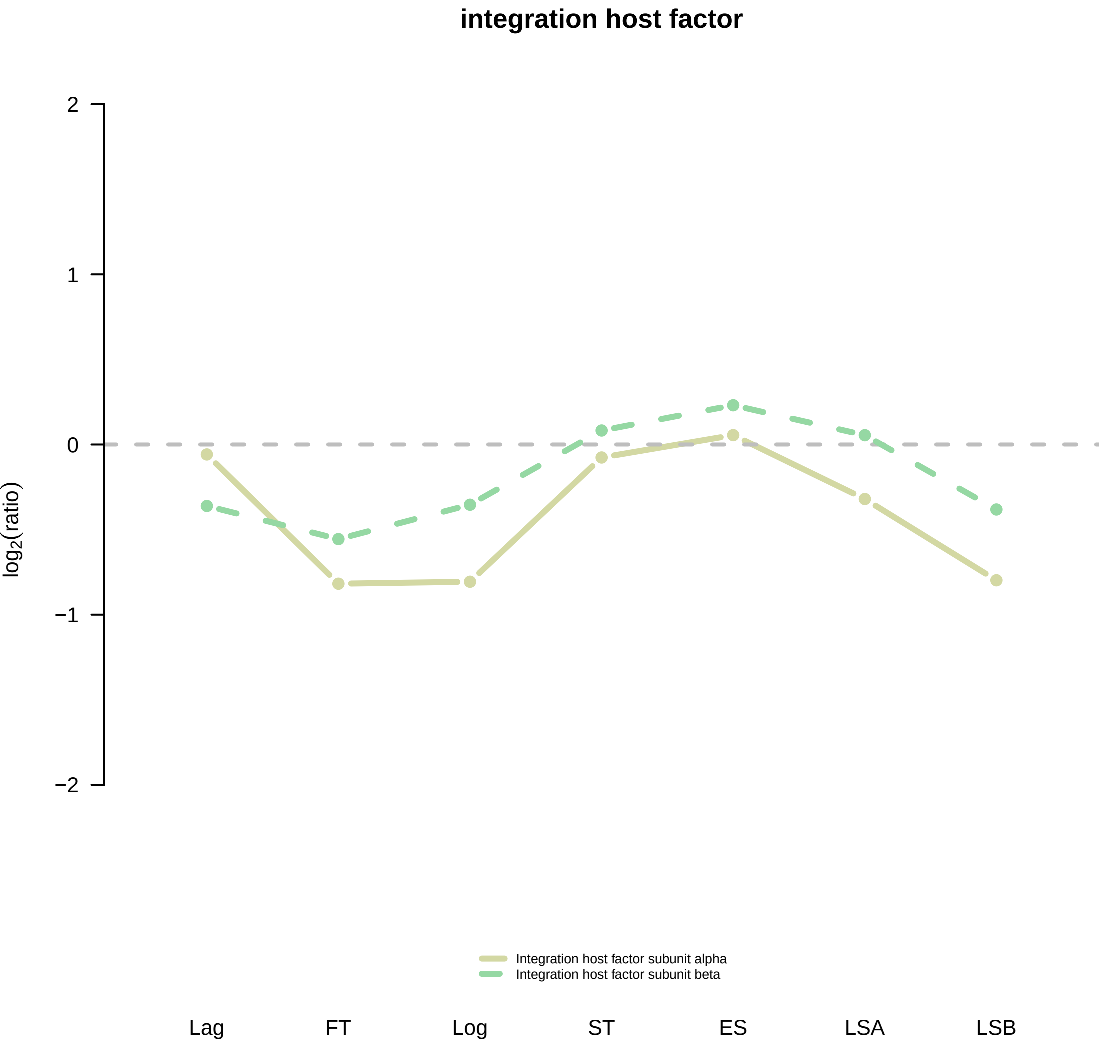

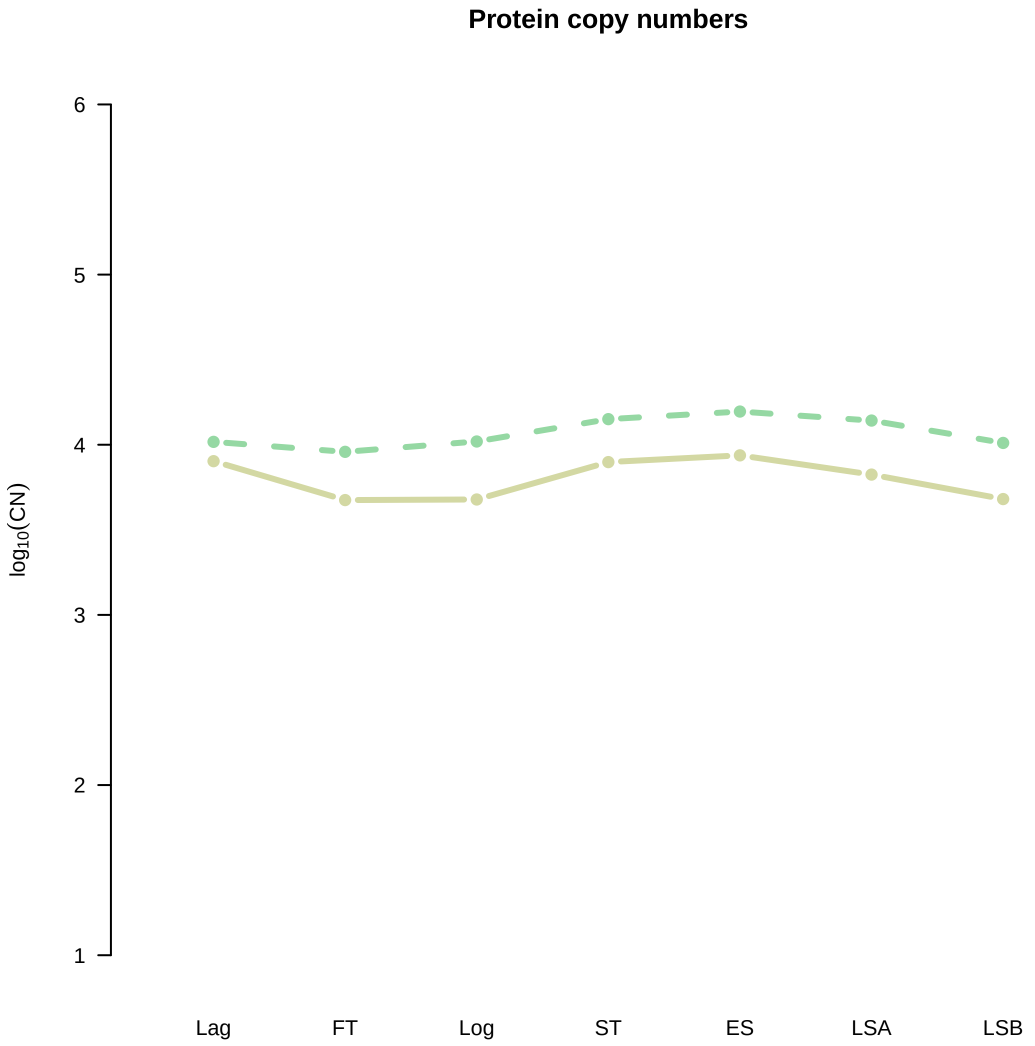

ihfA	P0A6X7	Integration host factor subuni ...
ihfB	P0A6Y1	Integration host factor subuni ...

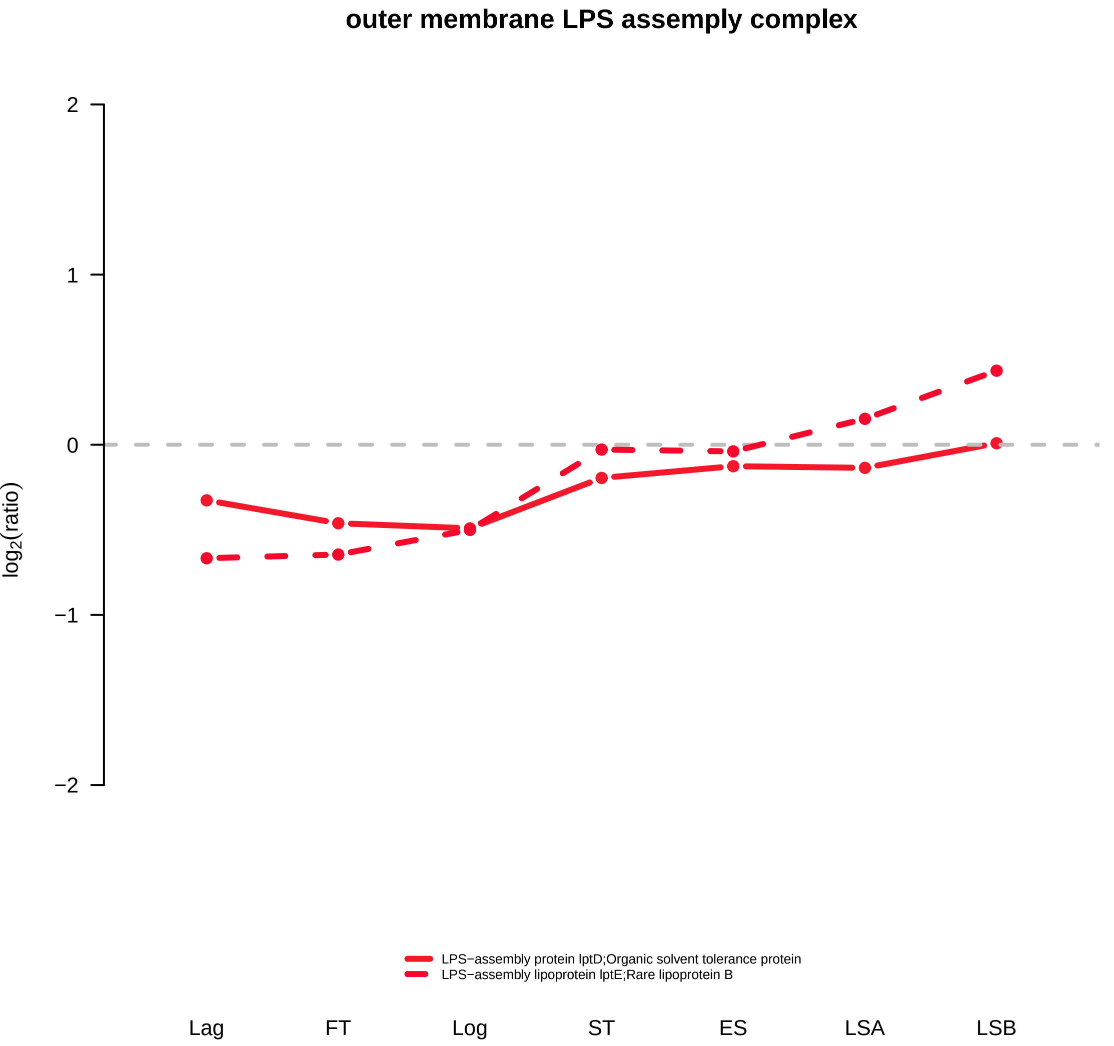

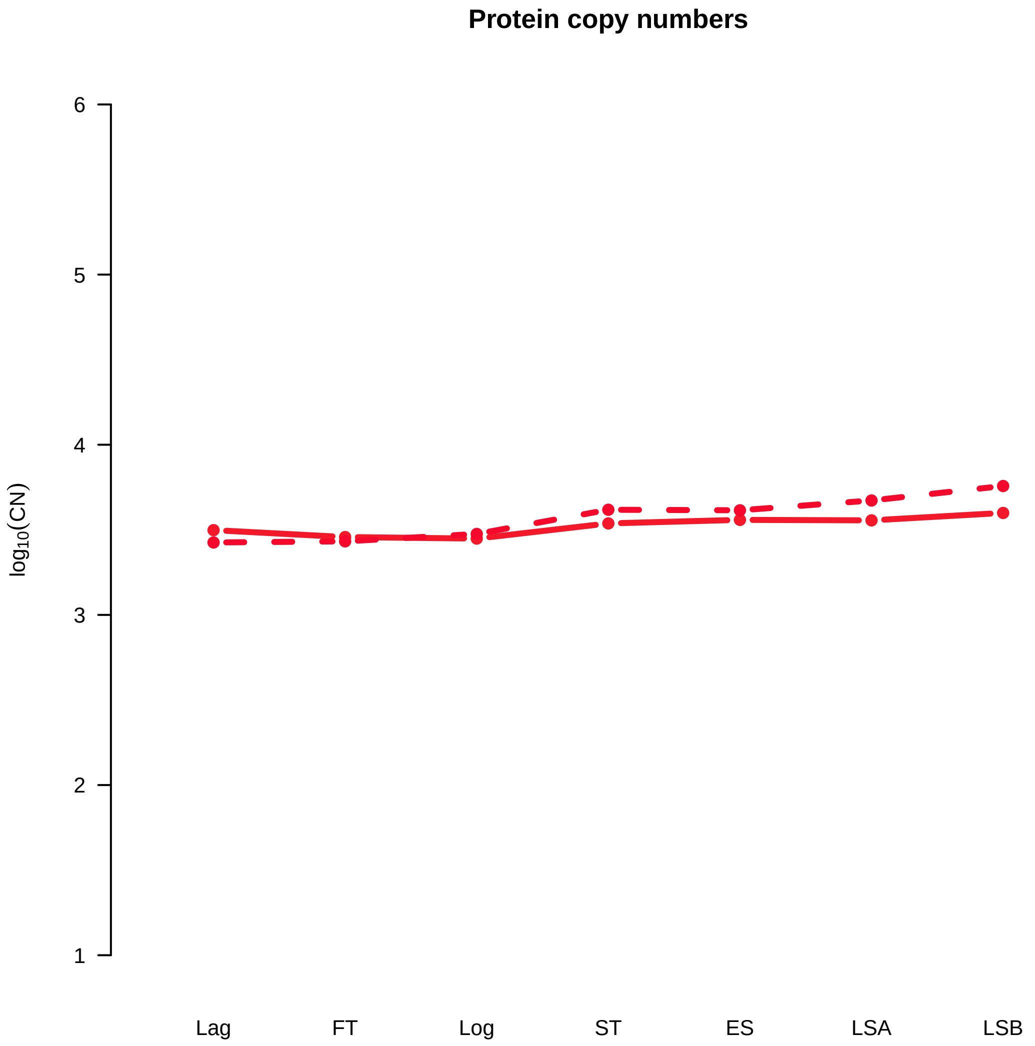

lptD	P31554	LPS−assembly protein lptD;Orga ...
lptE	P0ADC1	LPS−assembly lipoprotein lptE; ...

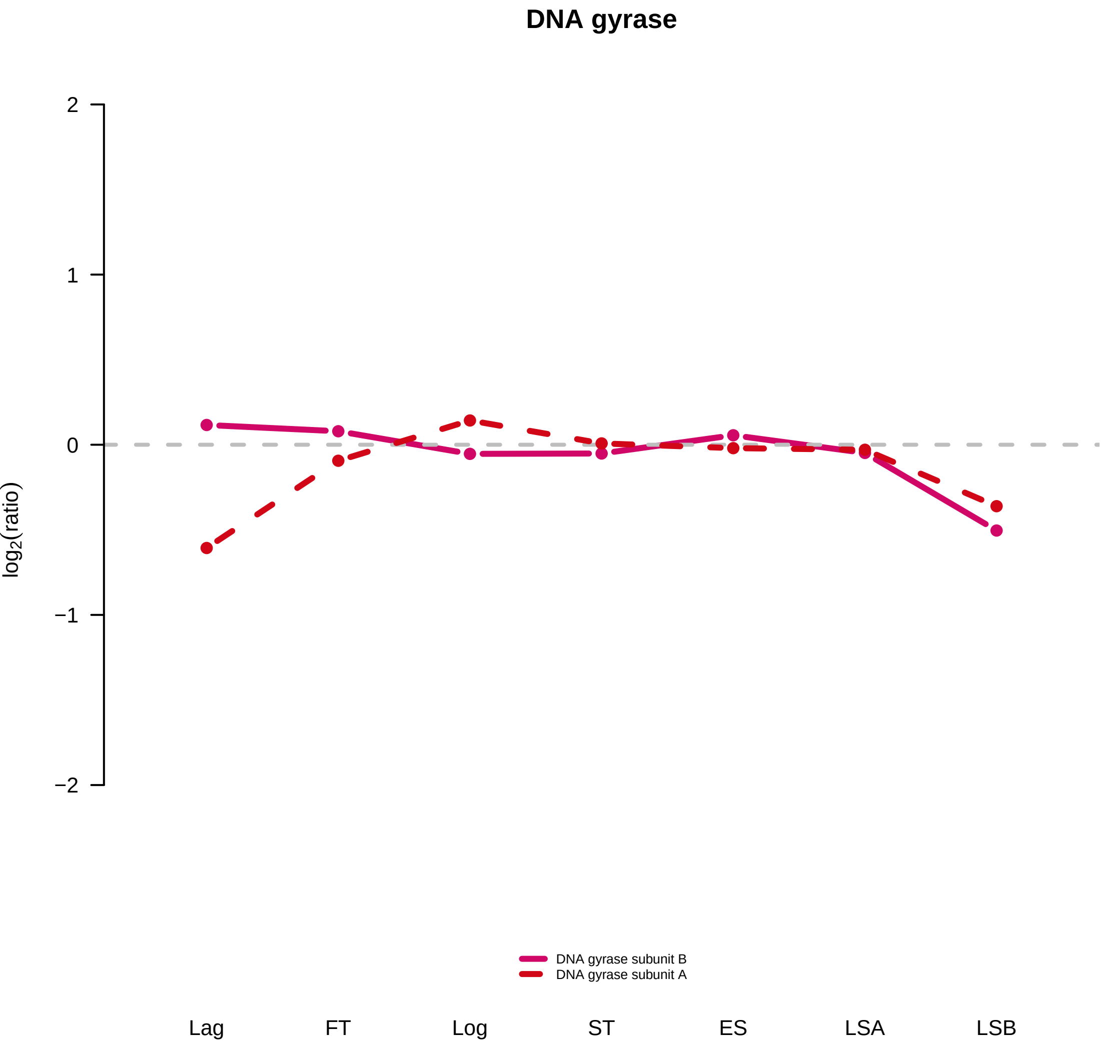

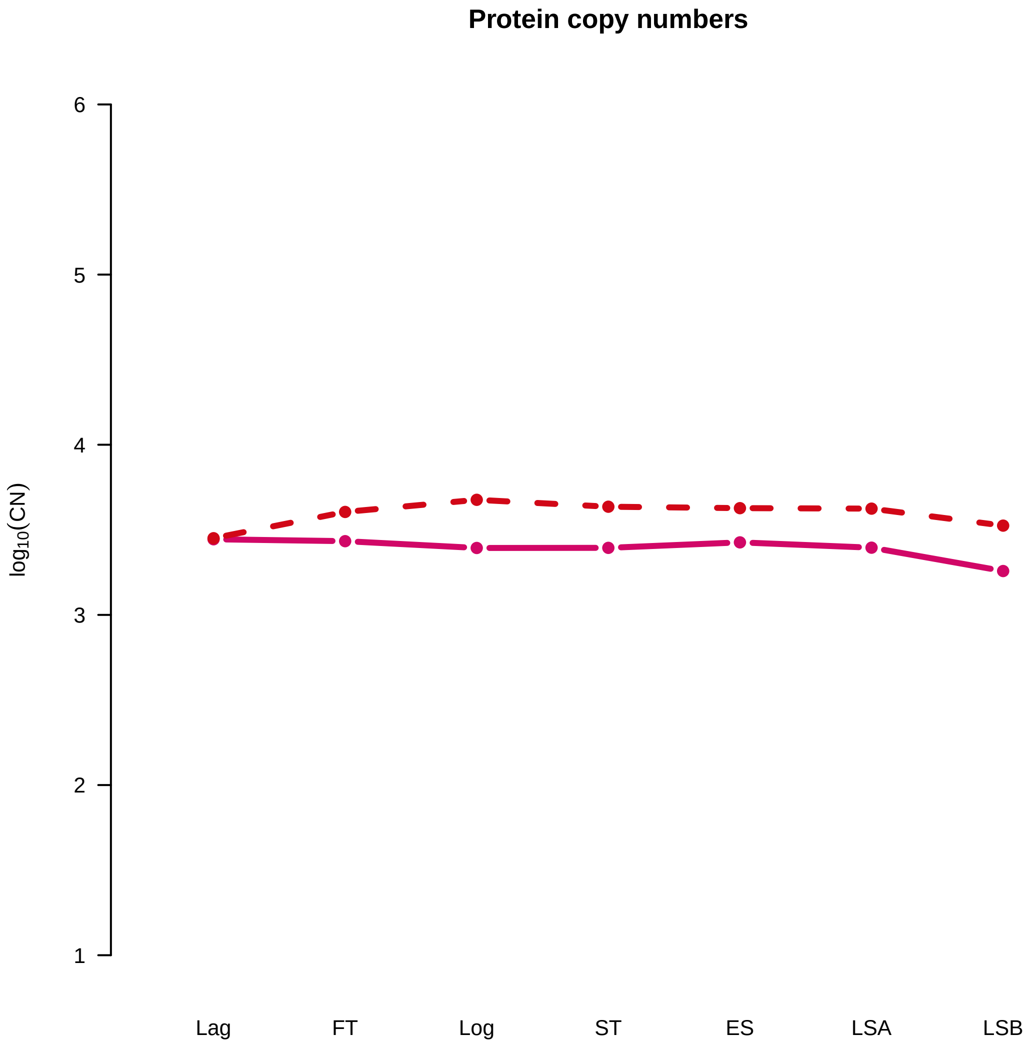

gyrB	P0AES6	DNA gyrase subunit B
gyrA	P0AES4	DNA gyrase subunit A

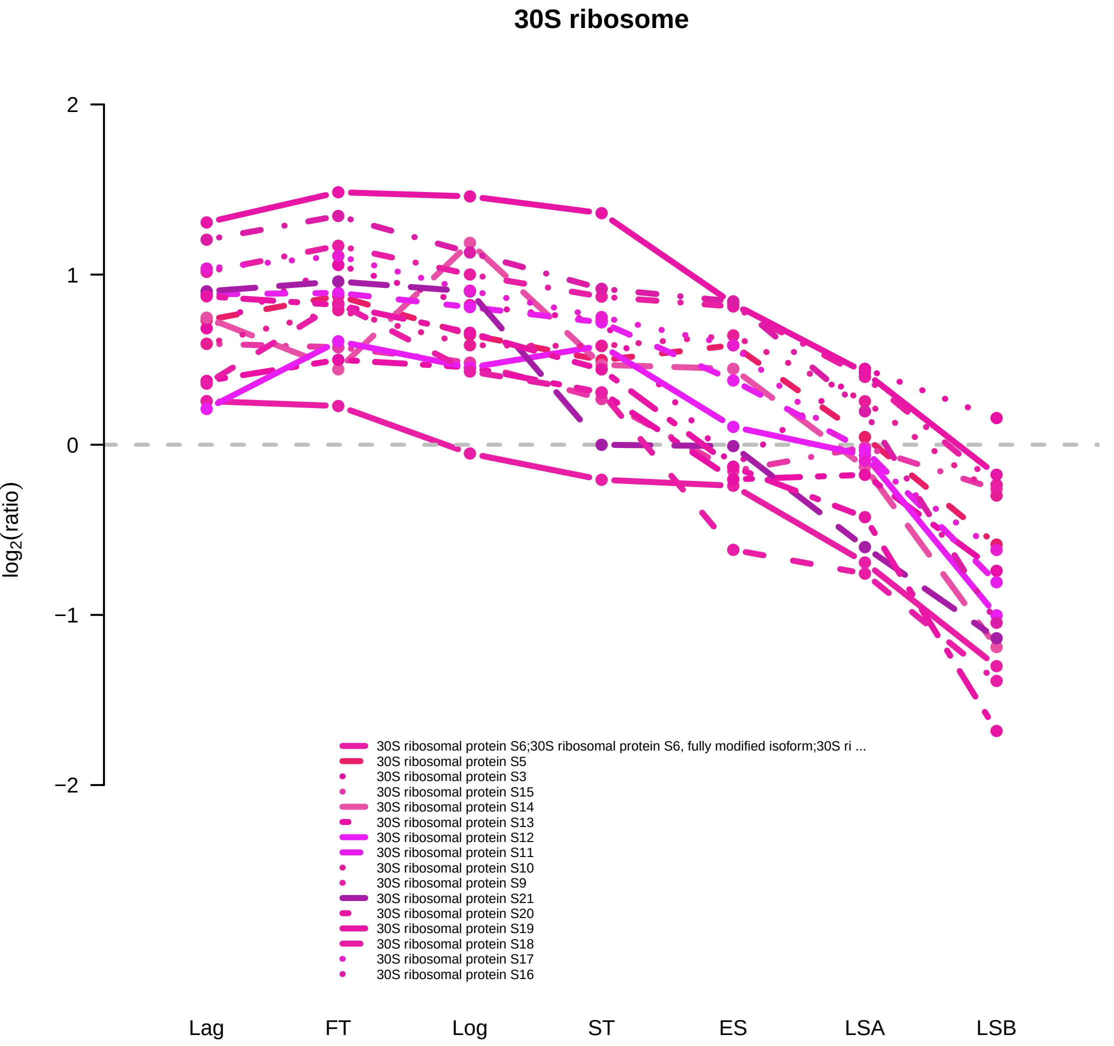

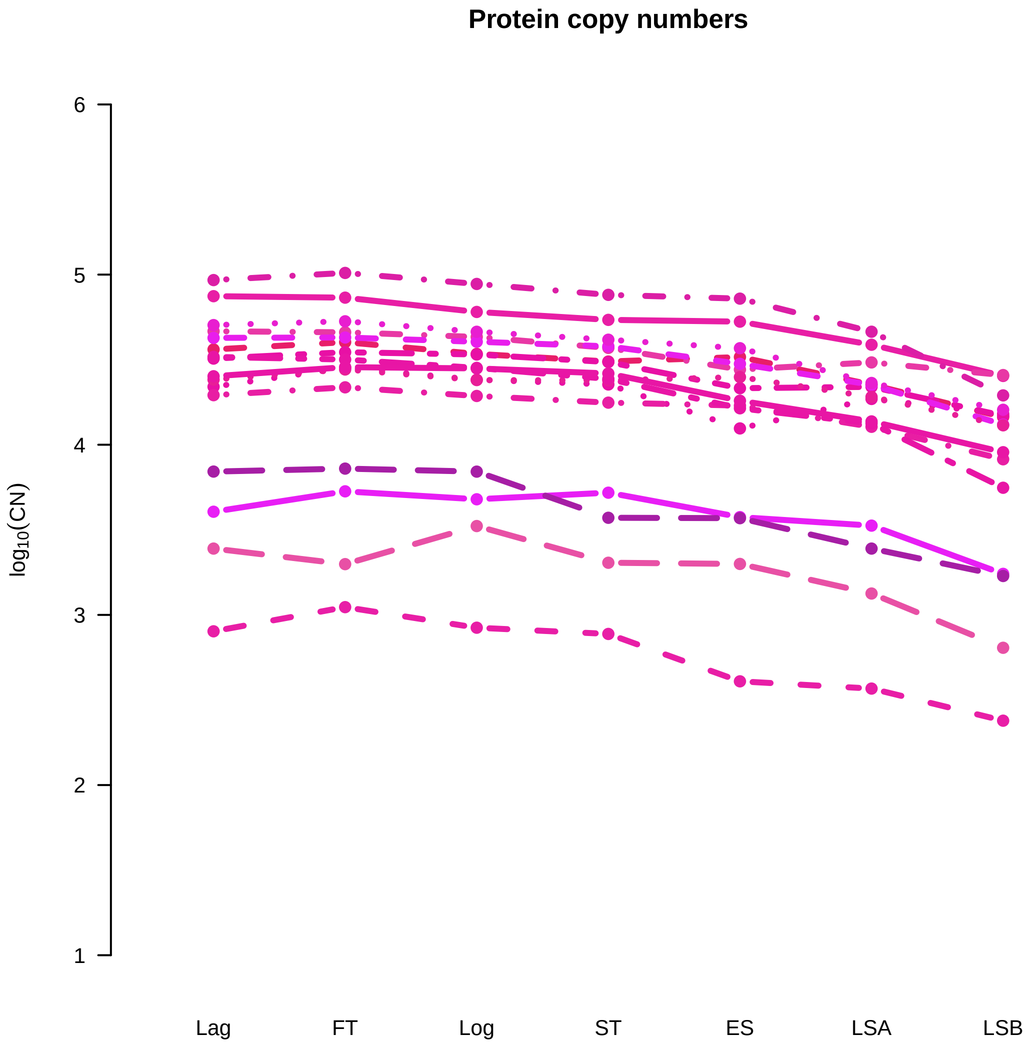

rpsF	P02358	30S ribosomal protein S6;30S r ...
rpsE	P0A7W1	30S ribosomal protein S5
rpsC	P0A7V3	30S ribosomal protein S3
rpsO	P0ADZ4	30S ribosomal protein S15
rpsN	P0AG59	30S ribosomal protein S14
rpsM	P0A7S9	30S ribosomal protein S13
rpsL	P0A7S3	30S ribosomal protein S12
rpsK	P0A7R9	30S ribosomal protein S11
rpsJ	P0A7R5	30S ribosomal protein S10
rpsI	P0A7X3	30S ribosomal protein S9
rpsU	P68679	30S ribosomal protein S21
rpsT	P0A7U7	30S ribosomal protein S20
rpsS	P0A7U3	30S ribosomal protein S19
rpsR	P0A7T7	30S ribosomal protein S18
rpsQ	P0AG63	30S ribosomal protein S17
rpsP	P0A7T3	30S ribosomal protein S16

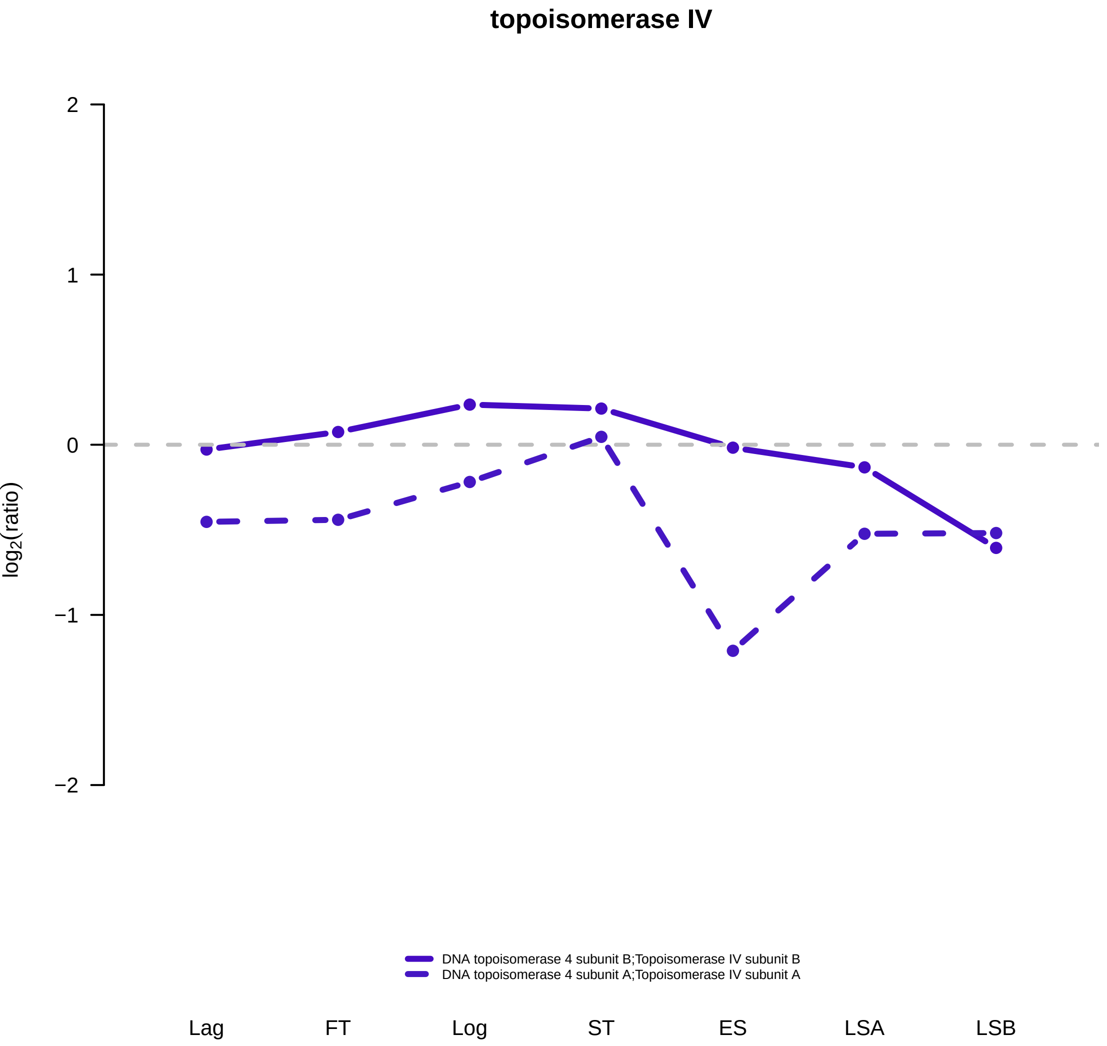

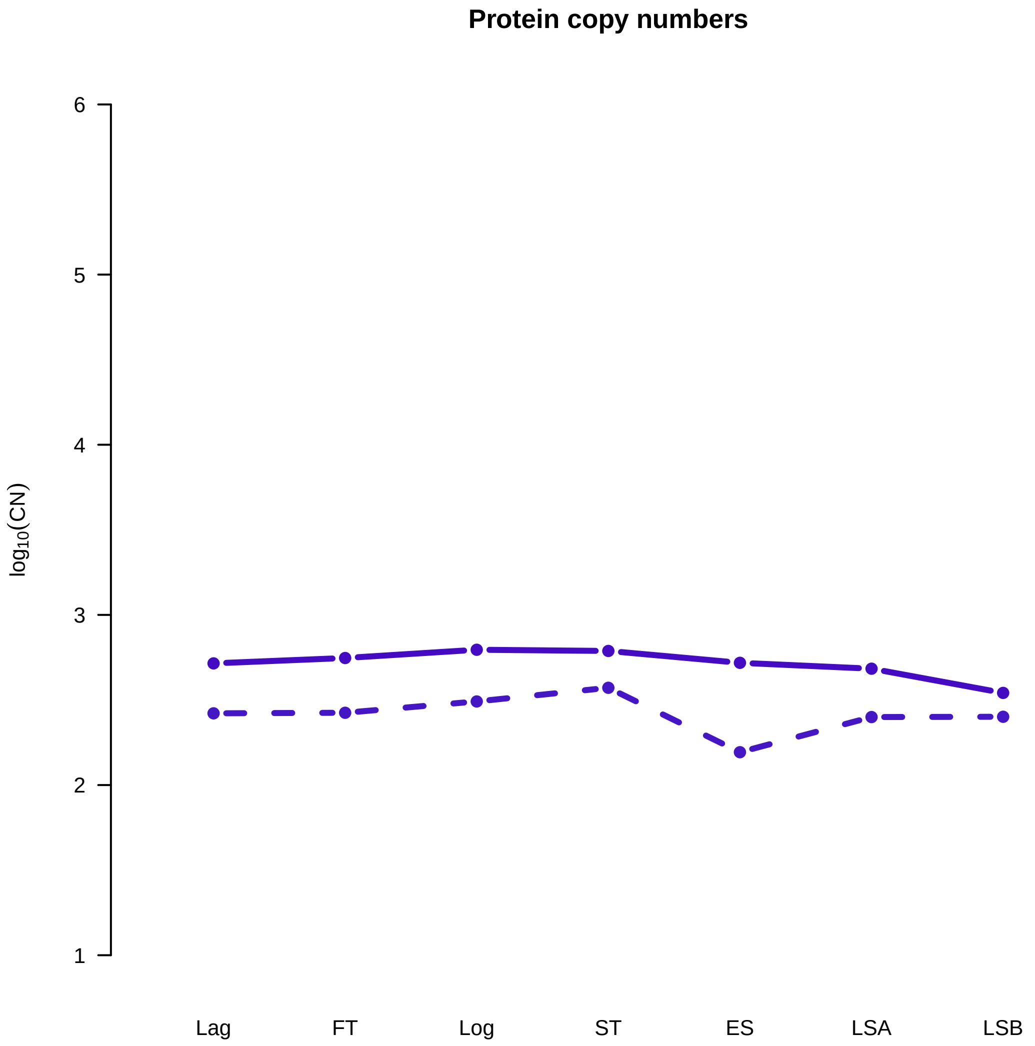

parE	P20083	DNA topoisomerase 4 subunit B; ...
parC	P0AFI2	DNA topoisomerase 4 subunit A; ...

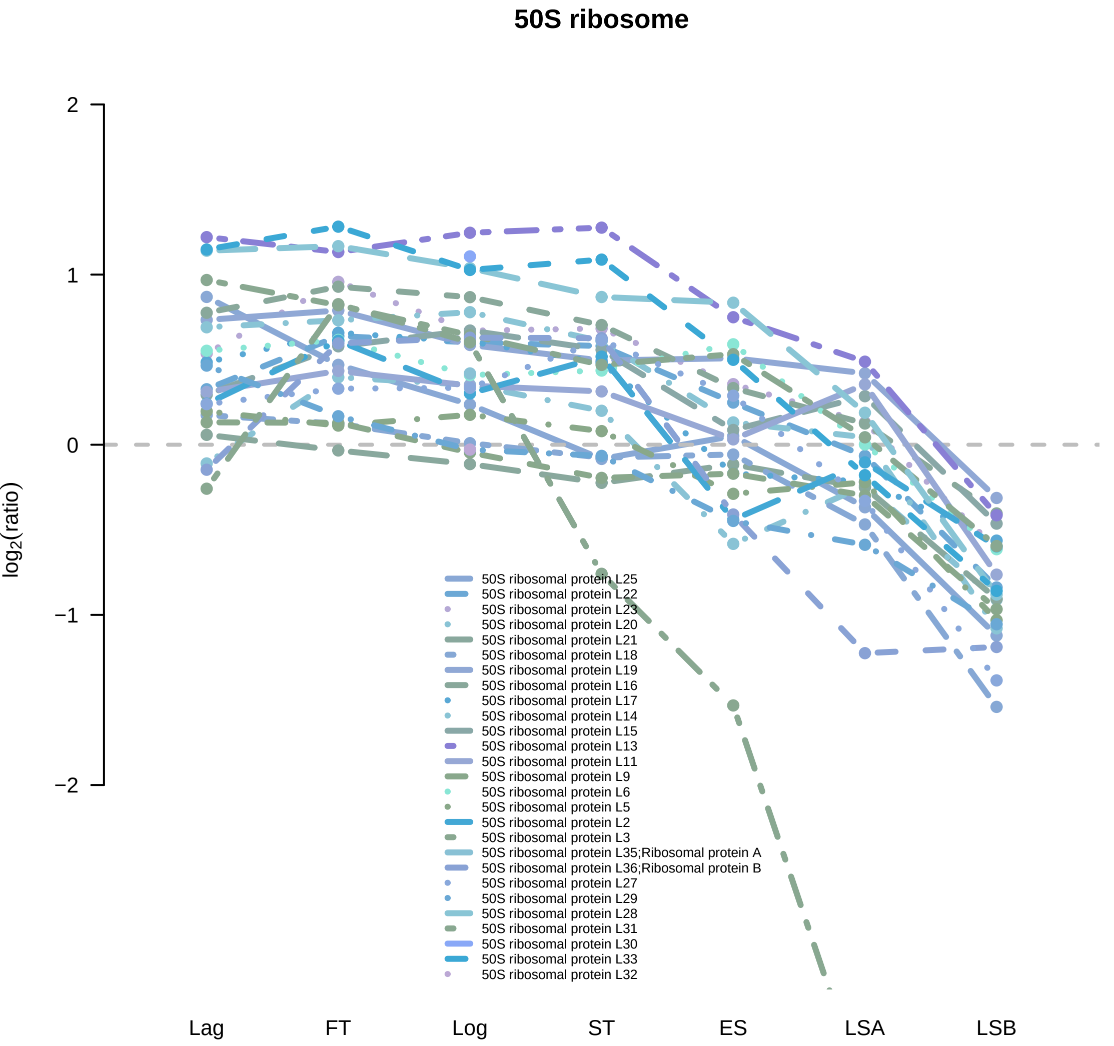

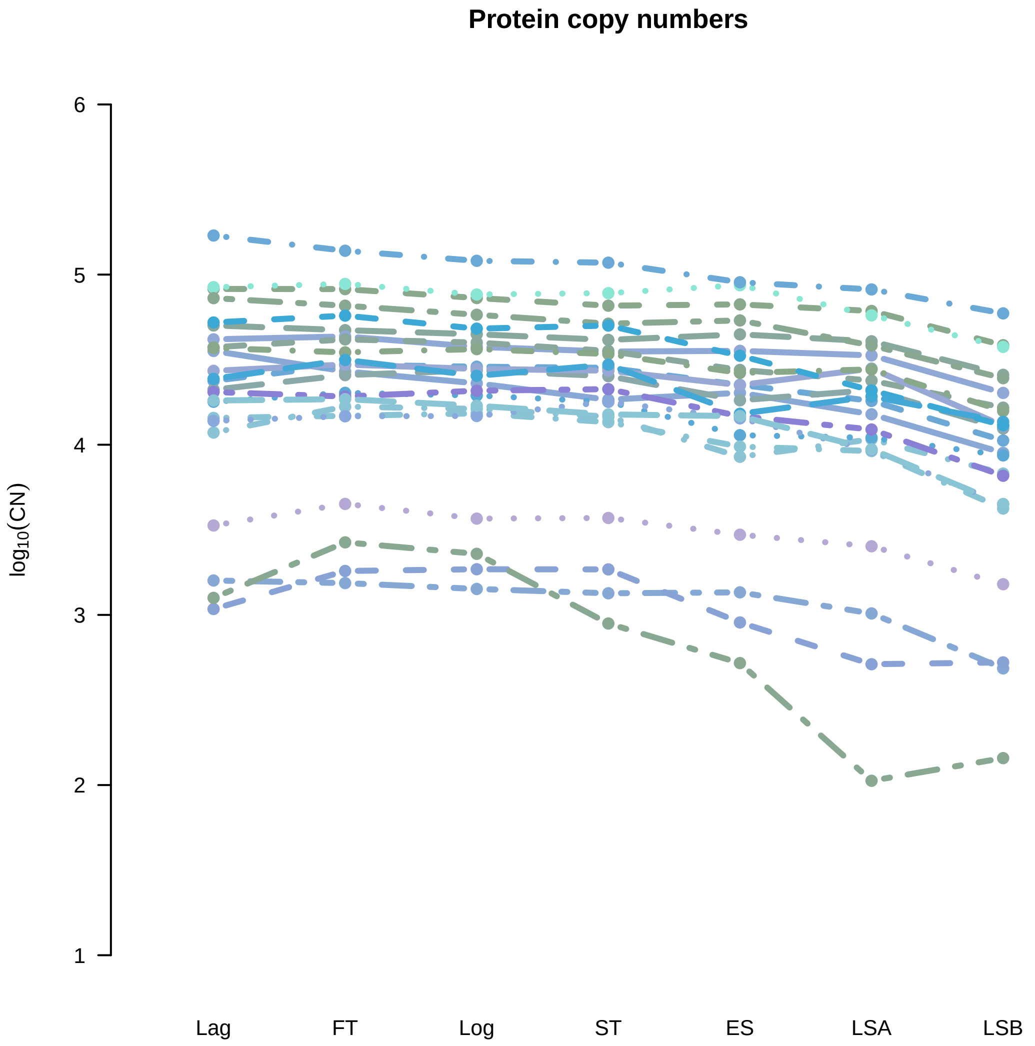

rplY	P68919	50S ribosomal protein L25
rplV	B1X6G6;C4ZUH0;P61175	50S ribosomal protein L22
rplW	P0ADZ0	50S ribosomal protein L23
rplT	P0A7L3	50S ribosomal protein L20
rplU	P0AG48	50S ribosomal protein L21
rplR	P0C018	50S ribosomal protein L18
rplS	P0A7K6	50S ribosomal protein L19
rplP	P0ADY7	50S ribosomal protein L16
rplQ	P0AG44	50S ribosomal protein L17
rplN	P0ADY3	50S ribosomal protein L14
rplO	P02413	50S ribosomal protein L15
rplM	P0AA10	50S ribosomal protein L13
rplK	P0A7J7	50S ribosomal protein L11
rplI	P0A7R1	50S ribosomal protein L9
rplF	P0AG55	50S ribosomal protein L6
rplE	P62399	50S ribosomal protein L5
rplB	P60422	50S ribosomal protein L2
rplC	P60438	50S ribosomal protein L3
rpmI	P0A7Q1	50S ribosomal protein L35;Ribo ...
rpmH	–	–
rpmJ	P0A7Q6	50S ribosomal protein L36;Ribo ...
rpmA	P0A7L8	50S ribosomal protein L27
rpmC	P0A7M6	50S ribosomal protein L29
rpmB	P0A7M2	50S ribosomal protein L28
rpmE	P0A7M9	50S ribosomal protein L31
rpmD	P0AG51	50S ribosomal protein L30
rpmG	P0A7N9	50S ribosomal protein L33
rpmF	P0A7N4	50S ribosomal protein L32

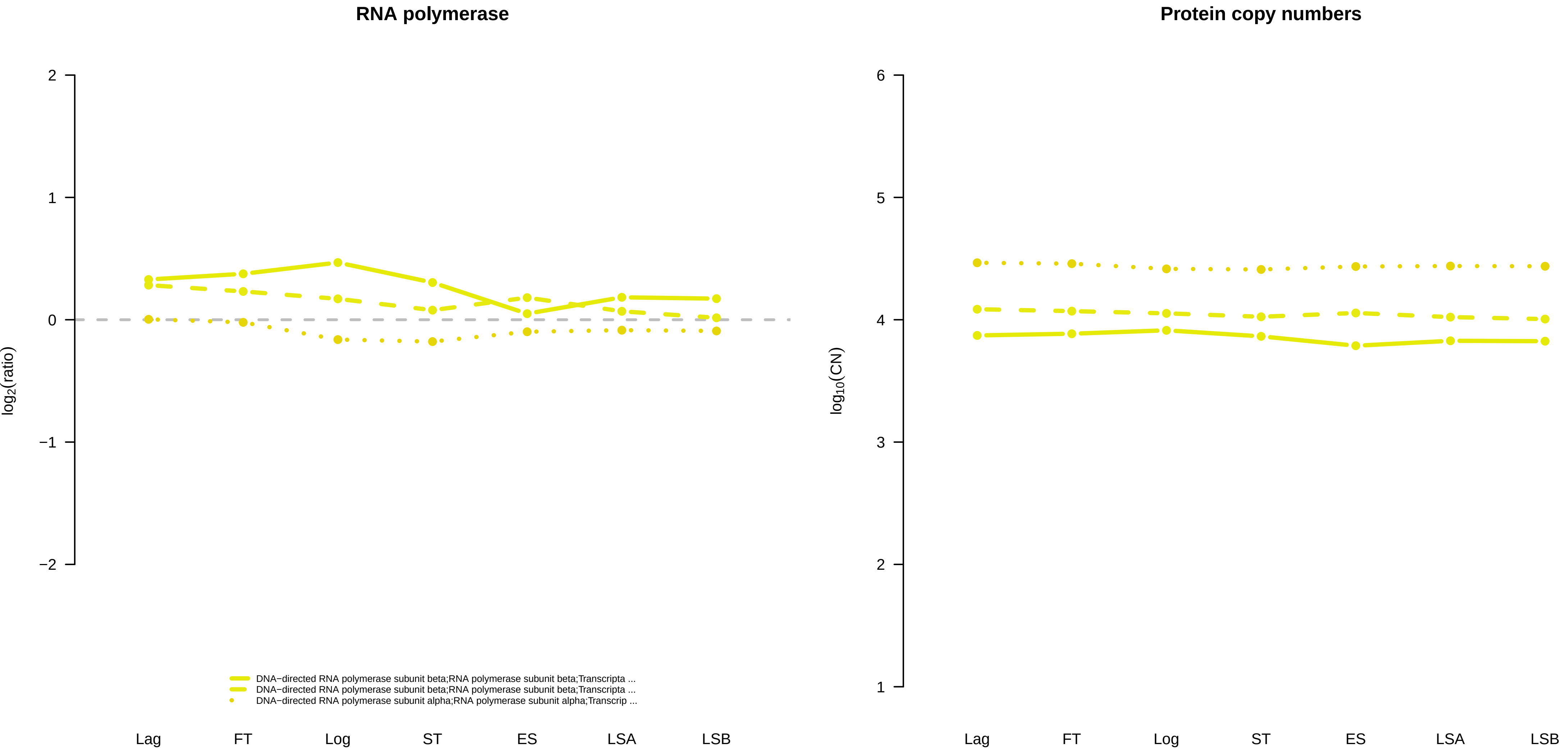

rpoC	P0A8T7	DNA−directed RNA polymerase su ...
rpoB	P0A8V2	DNA−directed RNA polymerase su ...
rpoA	P0A7Z4	DNA−directed RNA polymerase su ...
