# Supplementary material for: Characterization of the E. coli proteome and its modifications during growth and ethanol stress
Source: Front Microbiol. 2015 Feb 18;6:103. doi: 10.3389/fmicb.2015.00103 (PMC4332353; doi:10.3389/fmicb.2015.00103)
Supplement: Supplementary file 1 [file DataSheet1.ZIP › Supplementary File 2- DP pairwise comparison growth.pdf]

Pairwise Comparison of Dependent peptide analysis during growth (TP1-TP7)

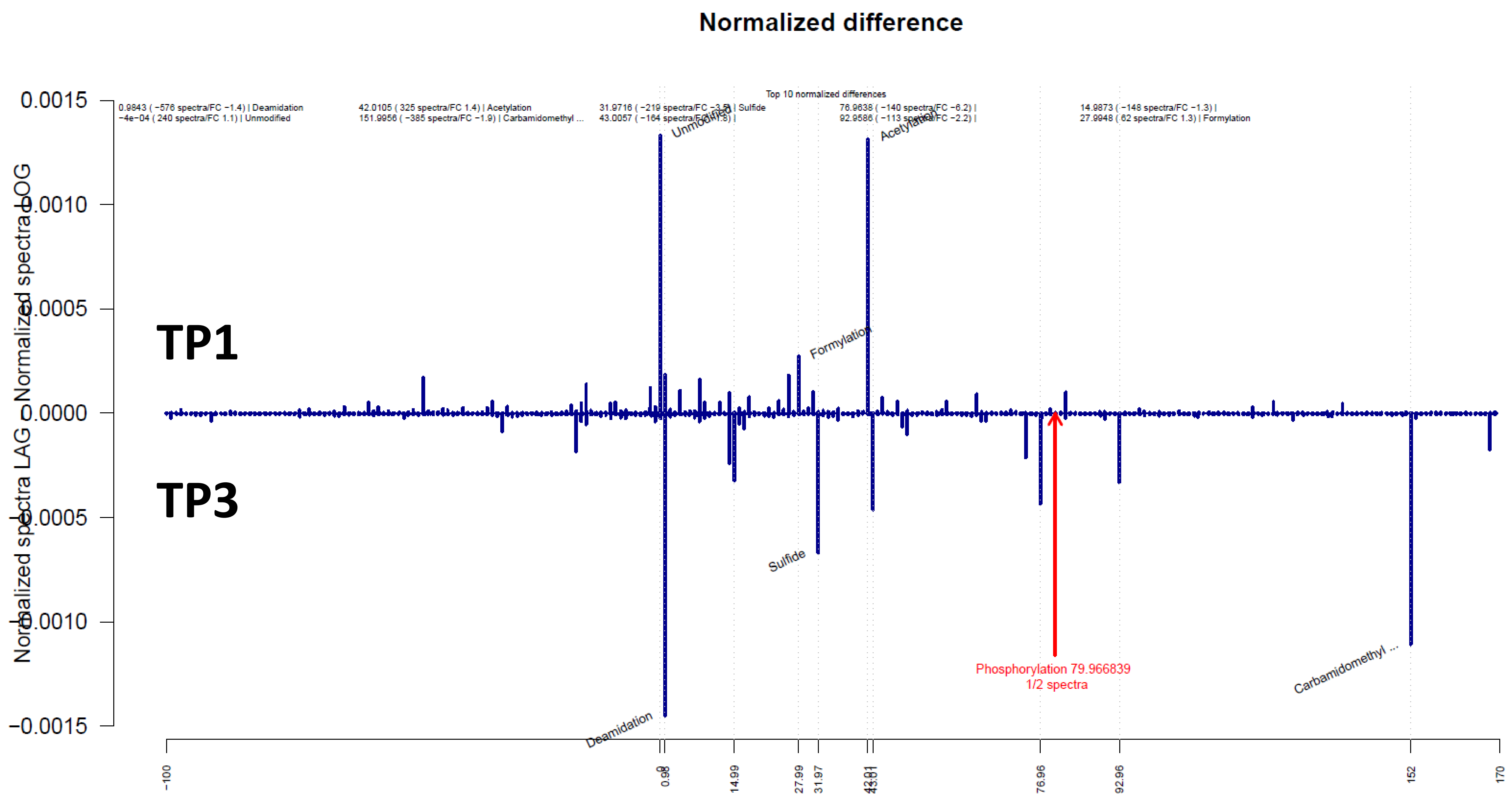
Normalized difference
Top 10 normalized differences
0.9843 (−576 spectra/FC −1.4) | Deamidation
−4e−04 (240 spectra/FC 1.1) | Unmodified
42.0105 (325 spectra/FC 1.4) | Acetylation
151.9956 (−385 spectra/FC −1.9) | Carbamidomethyl ...
31.9716 (−219 spectra/FC −3.5) | Sulfide
43.0057 (−164 spectra/FC −1.8) |
76.9638 (−140 spectra/FC −6.2) |
92.9586 (−113 spectra/FC −2.2) |
14.9873 (−148 spectra/FC −1.3) |
27.9948 (62 spectra/FC 1.3) | Formylation
Normalized spectra LAG − Normalized spectra LOG
0.0015
0.0010
0.0005
0.0000
−0.0005
−0.0010
−0.0015
TP1
TP3
Unmodified
Acetylation
Formylation
Sulfide
Deamidation
Carbamidomethyl ...
Phosphorylation 79.966839
1/2 spectra
−100
0.98
14.99
27.99
31.97
43.01
76.96
92.96
152
170

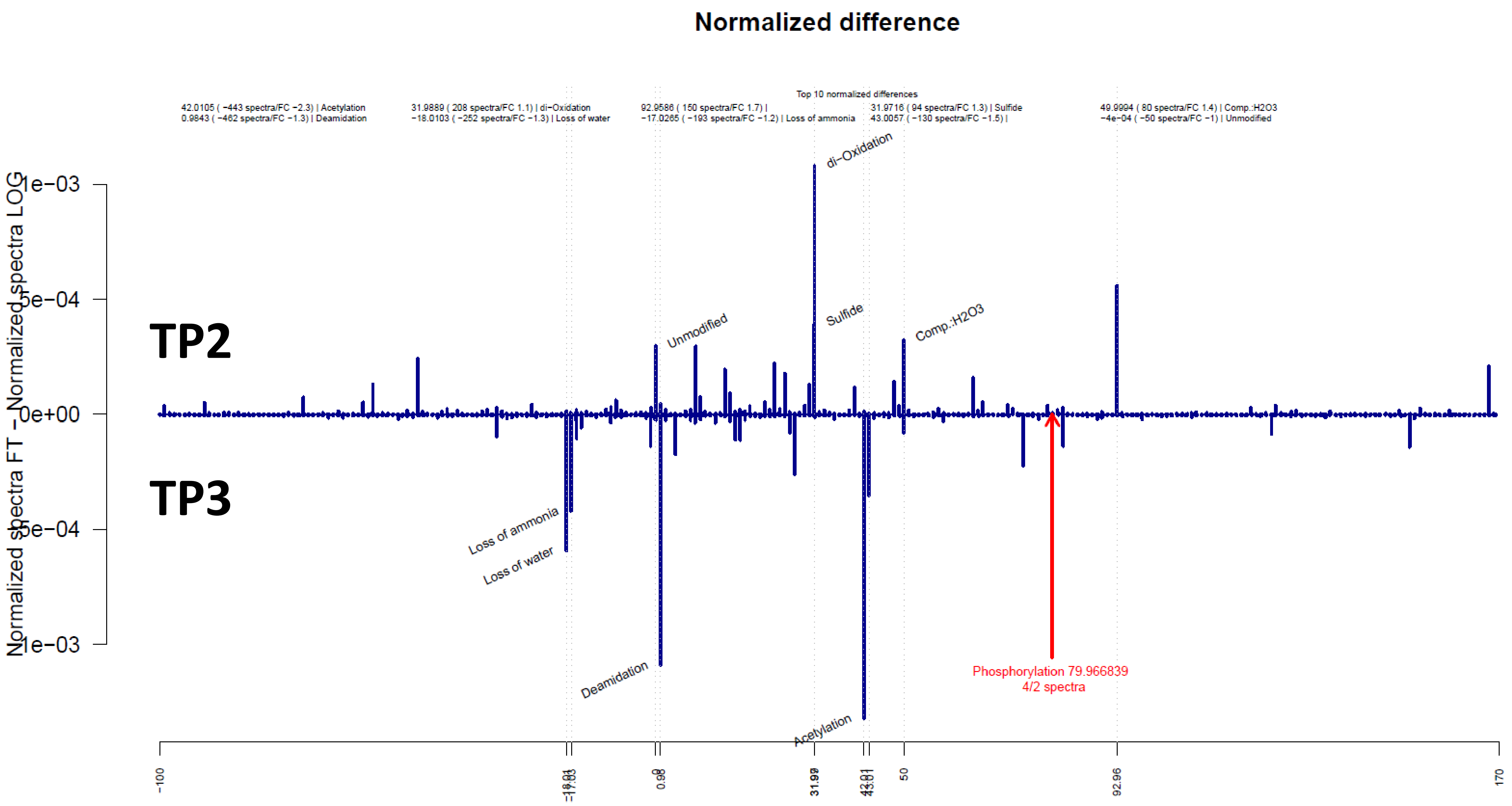

Normalized difference
Top 10 normalized differences
42.0105 (−443 spectra/FC −2.3) | Acetylation
0.9843 (−462 spectra/FC −1.3) | Deamidation
31.9889 (208 spectra/FC 1.1) | di−Oxidation
−18.0103 (−252 spectra/FC −1.3) | Loss of water
92.9586 (150 spectra/FC 1.7) |
−17.0265 (−193 spectra/FC −1.2) | Loss of ammonia
31.9716 (94 spectra/FC 1.3) | Sulfide
43.0057 (−130 spectra/FC −1.5) |
49.9994 (80 spectra/FC 1.4) | Comp.:H2O3
−4e−04 (−50 spectra/FC −1) | Unmodified
Normalized spectra FT − Normalized spectra LOG
1e−03
5e−04
0e+00
5e−04
1e−03
TP2
TP3
di−Oxidation
Sulfide
Unmodified
Comp.:H2O3
Loss of ammonia
Loss of water
Deamidation
Acetylation
Phosphorylation 79.966839
4/2 spectra
−100
50
92.96
170

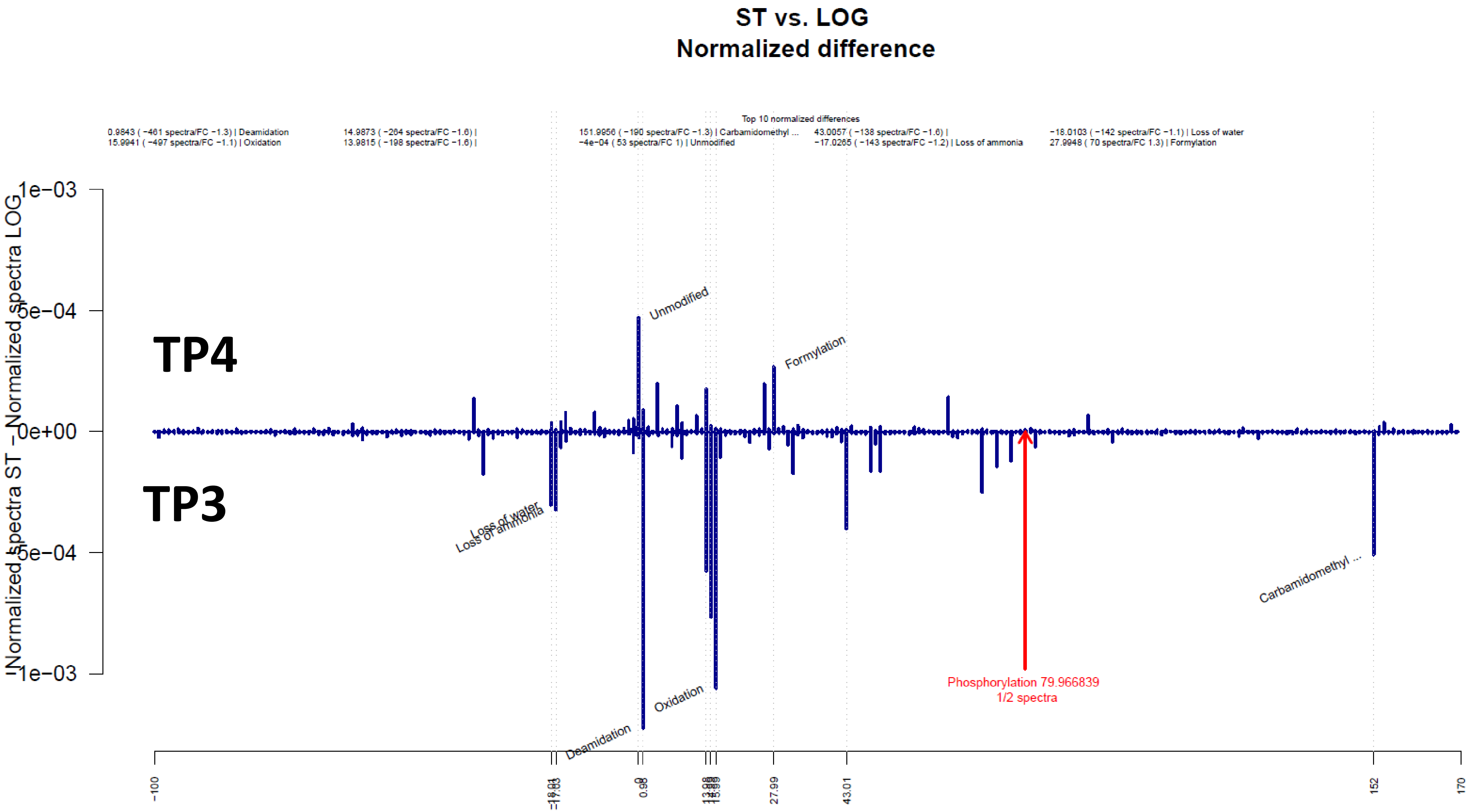
ST vs. LOG
Normalized difference
Top 10 normalized differences
0.9843 (−461 spectra/FC −1.3) | Deamidation
15.9941 (−497 spectra/FC −1.1) | Oxidation
14.9873 (−264 spectra/FC −1.6) |
13.9815 (−198 spectra/FC −1.6) |
151.9956 (−190 spectra/FC −1.3) | Carbamidomethyl ...
−4e−04 (53 spectra/FC 1) | Unmodified
43.0057 (−138 spectra/FC −1.6) |
−17.0265 (−143 spectra/FC −1.2) | Loss of ammonia
−18.0103 (−142 spectra/FC −1.1) | Loss of water
27.9948 (70 spectra/FC 1.3) | Formylation
Normalized spectra ST − Normalized spectra LOG
1e−03
5e−04
0e+00
−5e−04
−1e−03
TP4
TP3
Unmodified
Formylation
Loss of water
Loss of ammonia
Deamidation
Oxidation
Carbamidomethyl ...
Phosphorylation 79.966839
1/2 spectra
−100
0.98
27.99
43.01
152
170

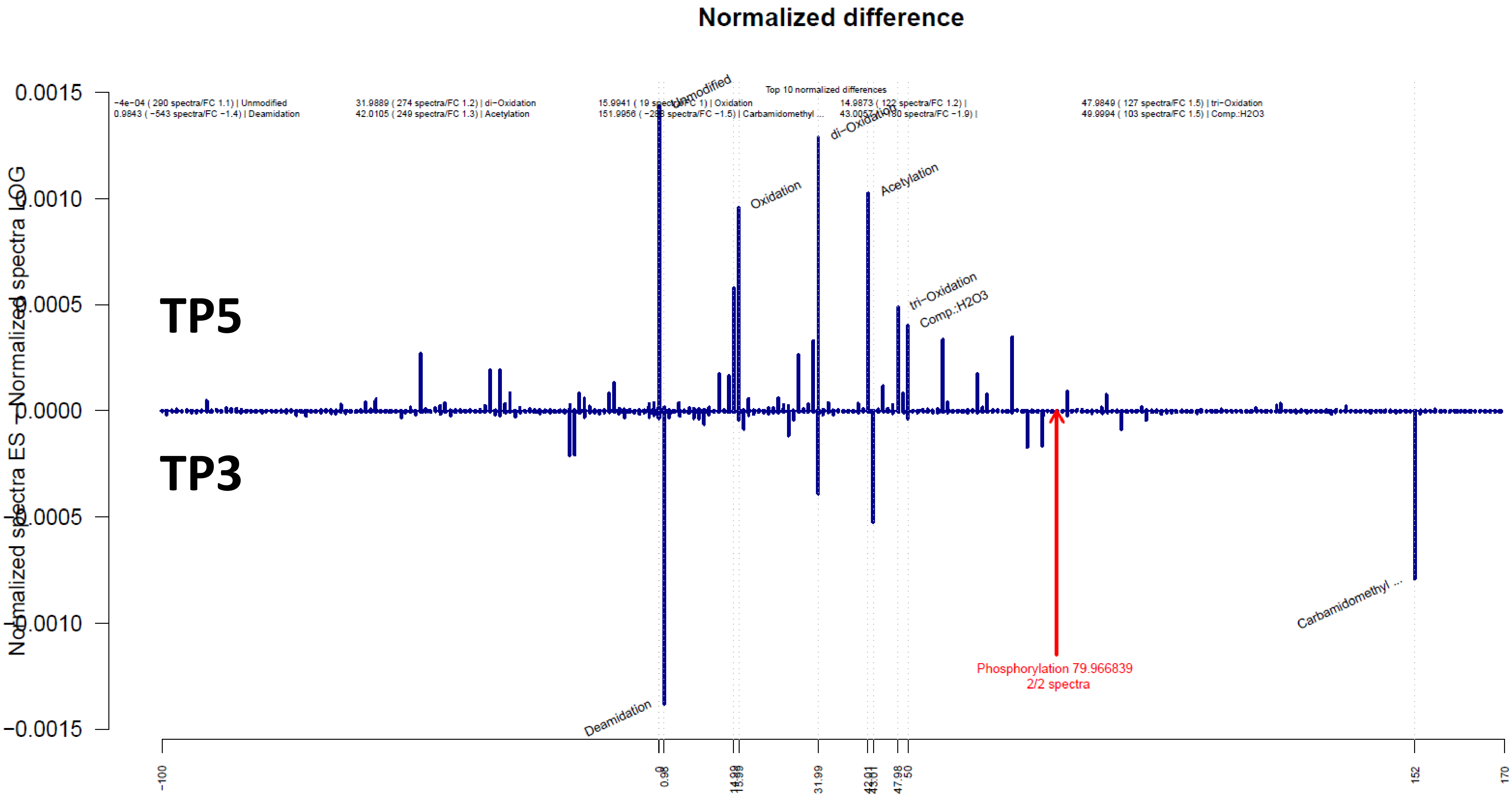

Normalized difference
Top 10 normalized differences
−4e−04 (290 spectra/FC 1.1) | Unmodified
0.9843 (−543 spectra/FC −1.4) | Deamidation
31.9889 (274 spectra/FC 1.2) | di−Oxidation
42.0105 (249 spectra/FC 1.3) | Acetylation
15.9941 (19 spectra/FC 1) | Oxidation
151.9956 (spectra/FC −1.5) | Carbamidomethyl ...
14.9873 (122 spectra/FC 1.2) |
43.0057 (spectra/FC −1.9) |
47.9849 (127 spectra/FC 1.5) | tri−Oxidation
49.9994 (103 spectra/FC 1.5) | Comp.:H2O3
Unmodified
Oxidation
di−Oxidation
Acetylation
tri−Oxidation
Comp.:H2O3
Deamidation
Carbamidomethyl ...
Phosphorylation 79.966839
2/2 spectra
TP5
TP3
Normalized spectra ES − Normalized spectra LOG
0.0015
0.0010
0.0005
0.0000
−0.0005
−0.0010
−0.0015
-100
0
0.98
14.99
15.99
31.99
42.01
43.01
47.98
50
152
170

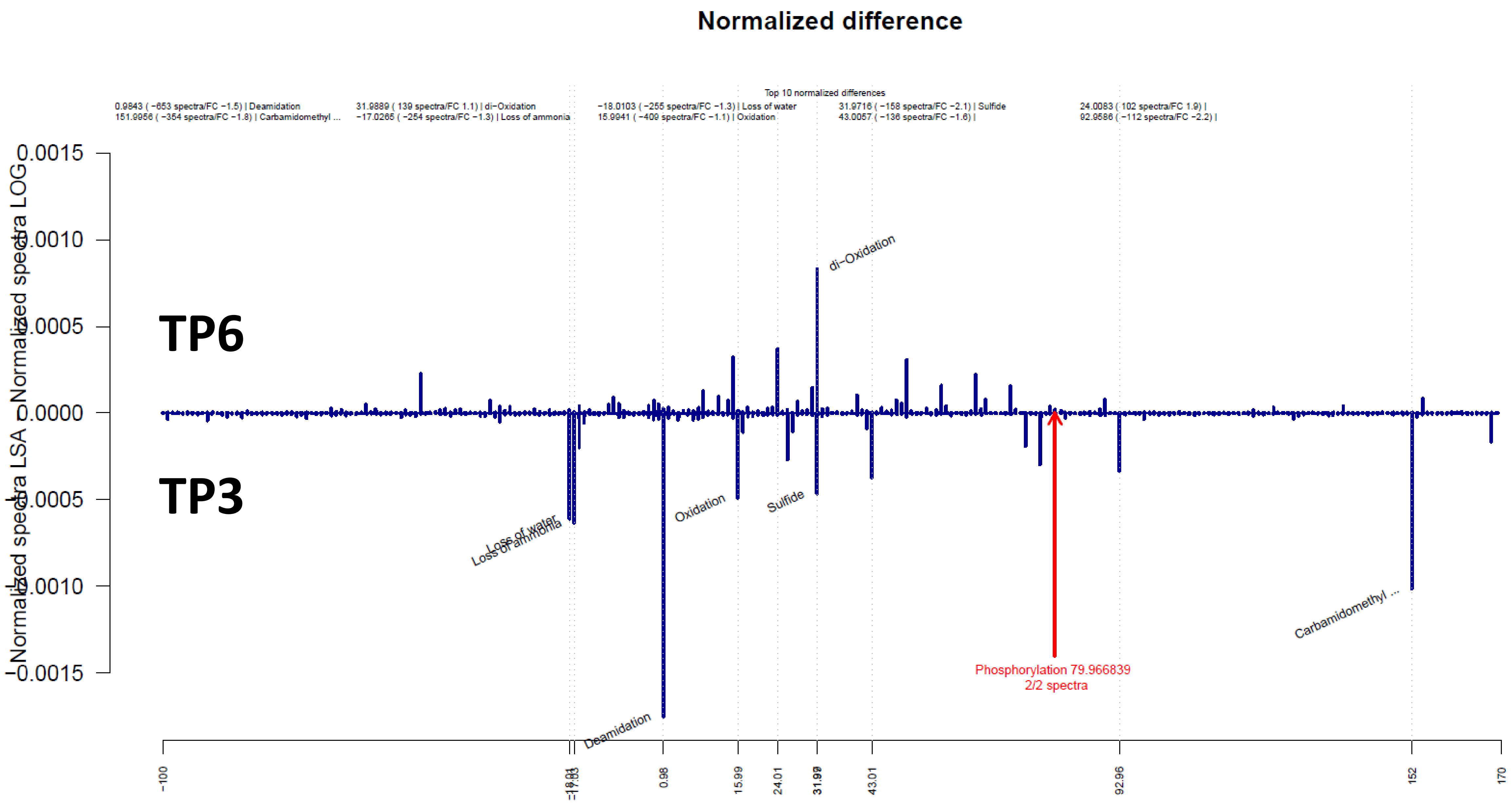

Normalized difference
Top 10 normalized differences
0.9843 (−653 spectra/FC −1.5) | Deamidation
151.9956 (−354 spectra/FC −1.8) | Carbamidomethyl ...
31.9889 (139 spectra/FC 1.1) | di−Oxidation
−17.0265 (−254 spectra/FC −1.3) | Loss of ammonia
−18.0103 (−255 spectra/FC −1.3) | Loss of water
15.9941 (−409 spectra/FC −1.1) | Oxidation
31.9716 (−158 spectra/FC −2.1) | Sulfide
43.0057 (−136 spectra/FC −1.6) |
24.0083 (102 spectra/FC 1.9) |
92.9586 (−112 spectra/FC −2.2) |
Normalized spectra LSA − Normalized spectra LOG
0.0015
0.0010
0.0005
0.0000
−0.0005
−0.0010
−0.0015
TP6
TP3
di−Oxidation
Loss of water
Loss of ammonia
Oxidation
Sulfide
Deamidation
Carbamidomethyl ...
Phosphorylation 79.966839
2/2 spectra
−100
0.98
15.99
24.01
43.01
92.96
152
170

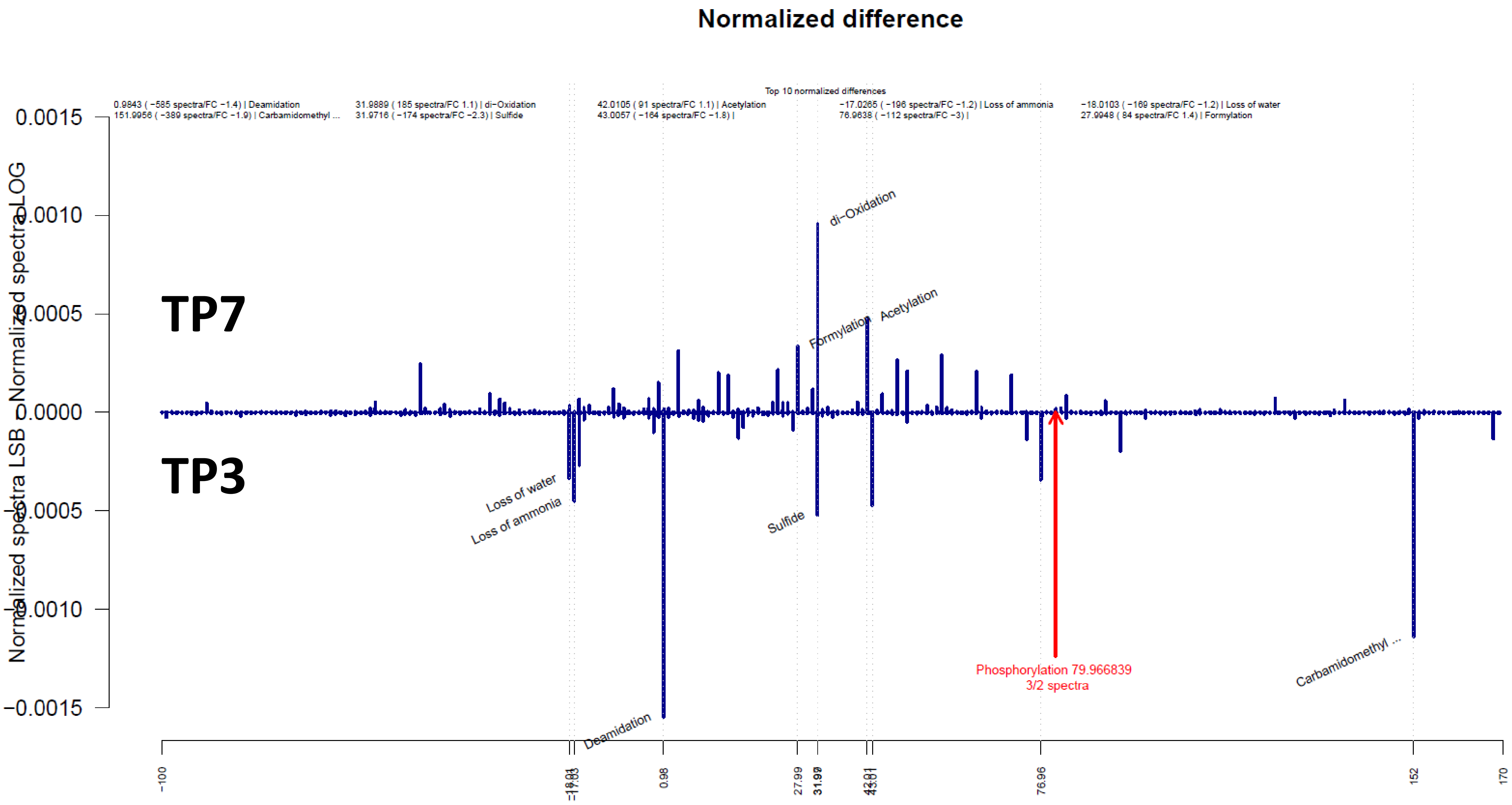

Normalized difference
Top 10 normalized differences
0.9843 (−585 spectra/FC −1.4) | Deamidation
151.9956 (−389 spectra/FC −1.9) | Carbamidomethyl ...
31.9889 (185 spectra/FC 1.1) | di−Oxidation
31.9716 (−174 spectra/FC −2.3) | Sulfide
42.0105 (91 spectra/FC 1.1) | Acetylation
43.0057 (−164 spectra/FC −1.8) |
−17.0265 (−196 spectra/FC −1.2) | Loss of ammonia
76.9638 (−112 spectra/FC −3) |
−18.0103 (−169 spectra/FC −1.2) | Loss of water
27.9948 (84 spectra/FC 1.4) | Formylation
Normalized spectra LSB - Normalized spectra LOG
TP7
TP3
di−Oxidation
Formylation
Acetylation
Loss of water
Loss of ammonia
Sulfide
Deamidation
Carbamidomethyl ...
Phosphorylation 79.966839
3/2 spectra
0.0015
0.0010
0.0005
0.0000
−0.0005
−0.0010
−0.0015
−100
0.98
27.99
76.96
152
170

Dependent peptide (DP) analysis during growth (TP1-TP7)

Quality Control : Dataset (SILAC) processed as unlabeled with DP function on

TP1

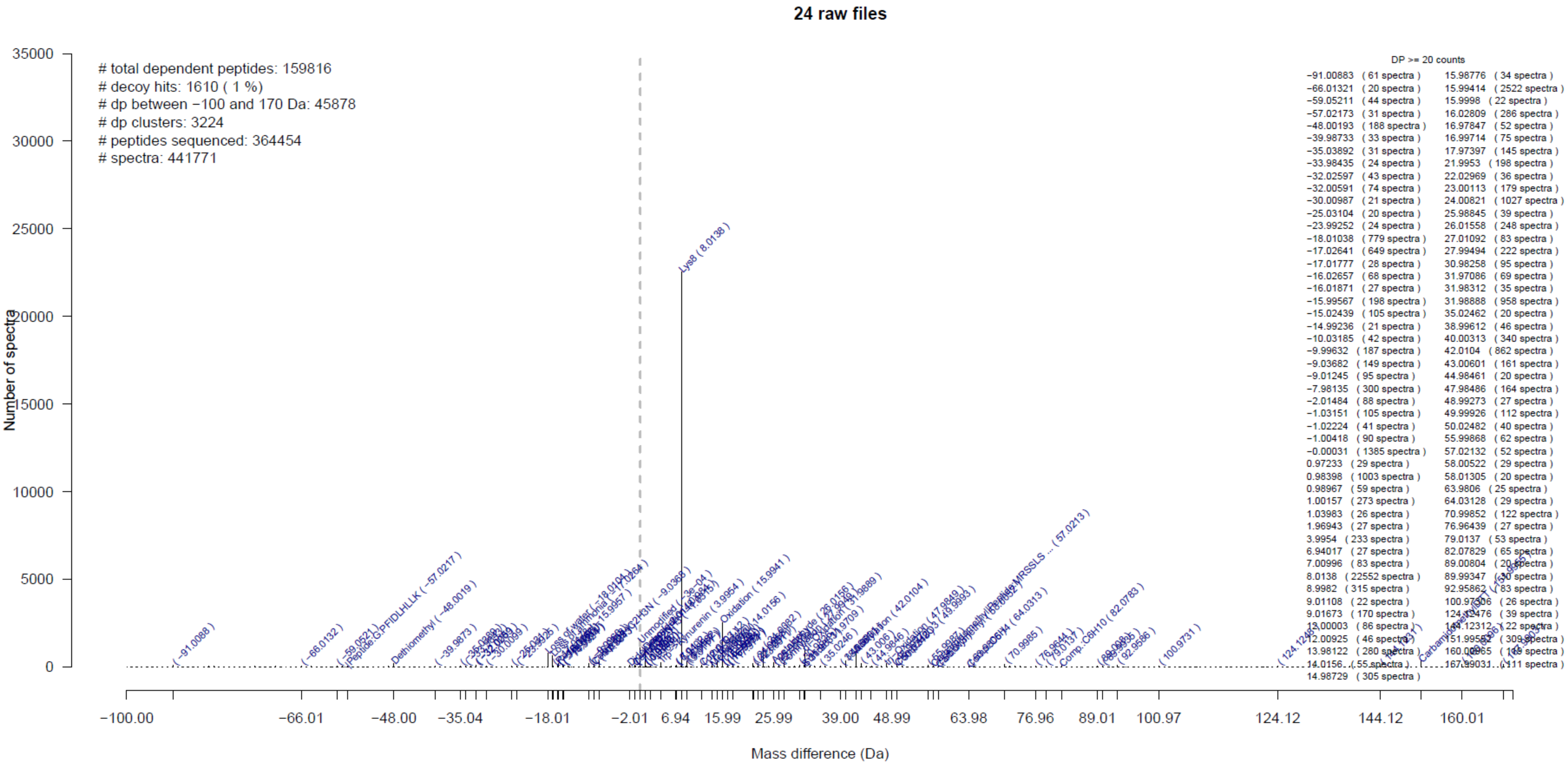

24 raw files
total dependent peptides: 159816
decoy hits: 1610 (1 %)
dp between −100 and 170 Da: 45878
dp clusters: 3224
peptides sequenced: 364454
spectra: 441771
Number of spectra
Mass difference (Da)
Lys8 (8.0138)
DP >= 20 counts
−91.00883 (61 spectra)
−66.01321 (20 spectra)
−59.05211 (44 spectra)
−57.02173 (31 spectra)
−48.00193 (188 spectra)
−39.98733 (33 spectra)
−35.03892 (31 spectra)
−33.98435 (24 spectra)
−32.02597 (43 spectra)
−32.00591 (74 spectra)
−30.00987 (21 spectra)
−25.03104 (20 spectra)
−23.99252 (24 spectra)
−18.01038 (779 spectra)
−17.02641 (649 spectra)
−17.01777 (28 spectra)
−16.02657 (68 spectra)
−16.01871 (27 spectra)
−15.99567 (198 spectra)
−15.02439 (105 spectra)
−14.99236 (21 spectra)
−10.03185 (42 spectra)
−9.99632 (187 spectra)
−9.03682 (149 spectra)
−9.01245 (95 spectra)
−7.98135 (300 spectra)
−2.01484 (88 spectra)
−1.03151 (105 spectra)
−1.02224 (41 spectra)
−1.00418 (90 spectra)
−0.00031 (1385 spectra)
0.97233 (29 spectra)
0.98398 (1003 spectra)
0.98967 (59 spectra)
1.00157 (273 spectra)
1.03983 (26 spectra)
1.96943 (27 spectra)
3.9954 (233 spectra)
6.94017 (27 spectra)
7.00996 (83 spectra)
8.0138 (22552 spectra)
8.9982 (315 spectra)
9.01108 (22 spectra)
9.01673 (170 spectra)
13.00003 (86 spectra)
12.00925 (46 spectra)
13.98122 (280 spectra)
14.0156 (55 spectra)
14.98729 (305 spectra)
15.98776 (34 spectra)
15.99414 (2522 spectra)
15.9998 (22 spectra)
16.02809 (286 spectra)
16.97847 (52 spectra)
16.99714 (75 spectra)
17.97397 (145 spectra)
21.9953 (198 spectra)
22.02969 (36 spectra)
23.00113 (179 spectra)
24.00821 (1027 spectra)
25.98845 (39 spectra)
26.01558 (248 spectra)
27.01092 (83 spectra)
27.99494 (222 spectra)
30.98258 (95 spectra)
31.97086 (69 spectra)
31.98312 (35 spectra)
31.98888 (958 spectra)
35.02462 (20 spectra)
38.99612 (46 spectra)
40.00313 (340 spectra)
42.0104 (862 spectra)
43.00601 (161 spectra)
44.98461 (20 spectra)
47.98486 (164 spectra)
48.99273 (27 spectra)
49.99926 (112 spectra)
50.02482 (40 spectra)
55.99868 (62 spectra)
57.02132 (52 spectra)
58.00522 (29 spectra)
58.01305 (20 spectra)
63.9806 (25 spectra)
64.03128 (29 spectra)
70.99852 (122 spectra)
76.96439 (27 spectra)
79.0137 (53 spectra)
82.07829 (65 spectra)
89.00804 (20 spectra)
89.99347 (30 spectra)
92.95862 (83 spectra)
100.97306 (26 spectra)
124.12476 (39 spectra)
144.12312 (22 spectra)
151.99582 (309 spectra)
160.00965
167.99031 (111 spectra)

TP2

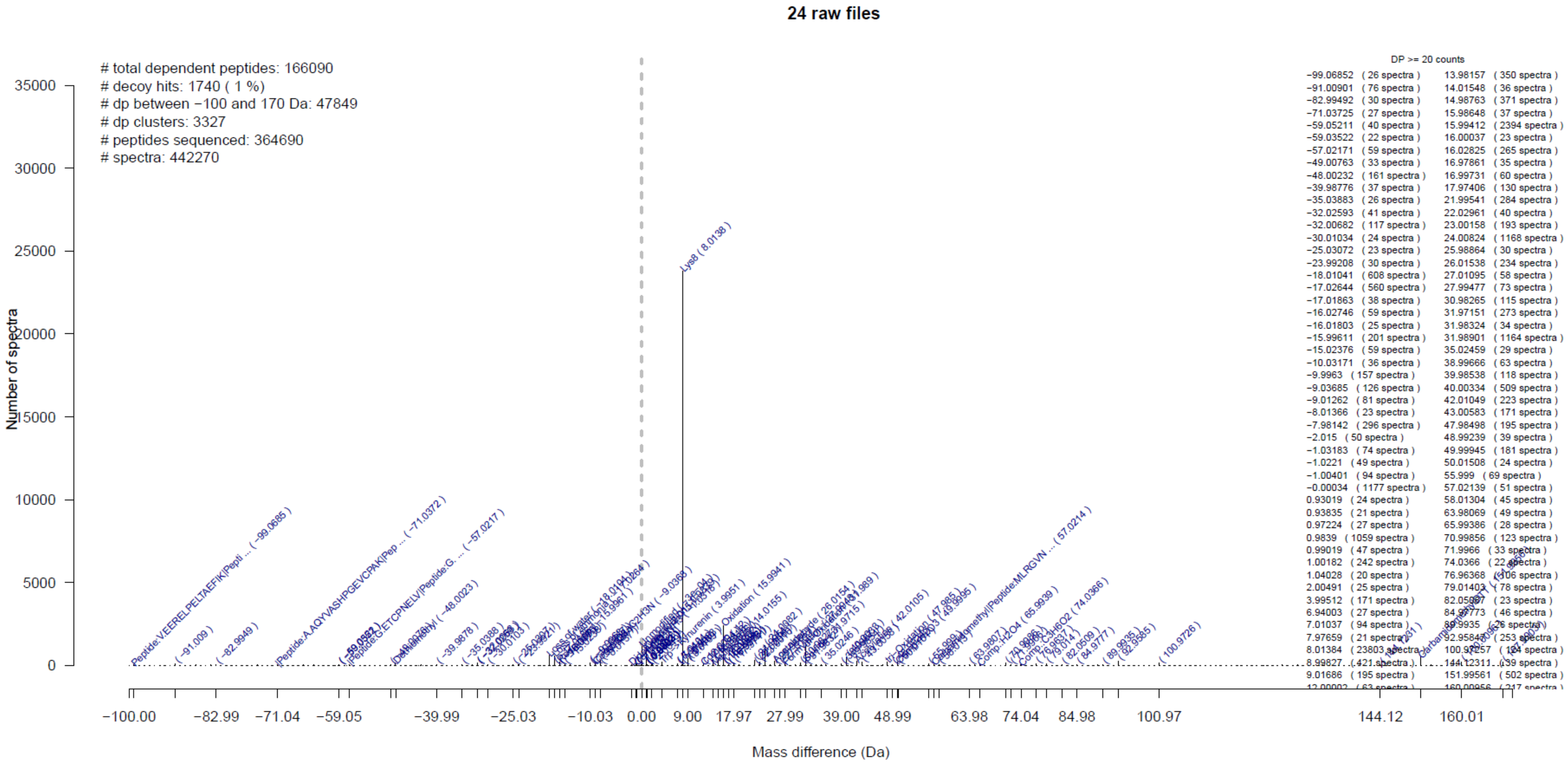
24 raw files
total dependent peptides: 166090
decoy hits: 1740 (1 %)
dp between −100 and 170 Da: 47849
dp clusters: 3327
peptides sequenced: 364690
spectra: 442270
Number of spectra
Mass difference (Da)
Lys8 (8.0138)
DP >= 20 counts
−99.06852 (26 spectra) 13.98157 (350 spectra)
−91.00901 (76 spectra) 14.01548 (36 spectra)
−82.99492 (30 spectra) 14.98763 (371 spectra)
−71.03725 (27 spectra) 15.98648 (37 spectra)
−59.05211 (40 spectra) 15.99412 (2394 spectra)
−59.03522 (22 spectra) 16.00037 (23 spectra)
−57.02171 (59 spectra) 16.02825 (265 spectra)
−49.00763 (33 spectra) 16.97861 (35 spectra)
−48.00232 (161 spectra) 16.99731 (60 spectra)
−39.98776 (37 spectra) 17.97406 (130 spectra)
−35.03883 (26 spectra) 21.99541 (284 spectra)
−32.02593 (41 spectra) 22.02961 (40 spectra)
−32.00682 (117 spectra) 23.00158 (193 spectra)
−30.01034 (24 spectra) 24.00824 (1168 spectra)
−25.03072 (23 spectra) 25.98864 (30 spectra)
−23.99208 (30 spectra) 26.01538 (234 spectra)
−18.01041 (608 spectra) 27.01095 (58 spectra)
−17.02644 (560 spectra) 27.99477 (73 spectra)
−17.01863 (38 spectra) 30.98265 (115 spectra)
−16.02746 (59 spectra) 31.97151 (273 spectra)
−16.01803 (25 spectra) 31.98324 (34 spectra)
−15.99611 (201 spectra) 31.98901 (1164 spectra)
−15.02376 (59 spectra) 35.02459 (29 spectra)
−10.03171 (36 spectra) 38.99666 (63 spectra)
−9.9963 (157 spectra) 39.98538 (118 spectra)
−9.03685 (126 spectra) 40.00334 (509 spectra)
−9.01262 (81 spectra) 42.01049 (223 spectra)
−8.01366 (23 spectra) 43.00583 (171 spectra)
−7.98142 (296 spectra) 47.98498 (195 spectra)
−2.015 (50 spectra) 48.99239 (39 spectra)
−1.03183 (74 spectra) 49.99945 (181 spectra)
−1.0221 (49 spectra) 50.01508 (24 spectra)
−1.00401 (94 spectra) 55.999 (69 spectra)
−0.00034 (1177 spectra) 57.02139 (51 spectra)
0.93019 (24 spectra) 58.01304 (45 spectra)
0.93835 (21 spectra) 63.98069 (49 spectra)
0.97224 (27 spectra) 65.99386 (28 spectra)
0.9839 (1059 spectra) 70.99856 (123 spectra)
0.99019 (47 spectra) 71.9966 (33 spectra)
1.00182 (242 spectra) 74.0366 (22 spectra)
1.04028 (20 spectra) 76.96368 (106 spectra)
2.00491 (25 spectra) 79.01403 (78 spectra)
3.99512 (171 spectra) 82.05087 (23 spectra)
6.94003 (27 spectra) 84.97773 (46 spectra)
7.01037 (94 spectra) 89.9935 (26 spectra)
7.97659 (21 spectra) 92.95847 (253 spectra)
8.01384 (23803 spectra) 100.97257 (124 spectra)
8.99827 (421 spectra) 144.12311 (39 spectra)
9.01686 (195 spectra) 151.99561 (502 spectra)
12.00002 (63 spectra) 160.00956 (217 spectra)

TP3

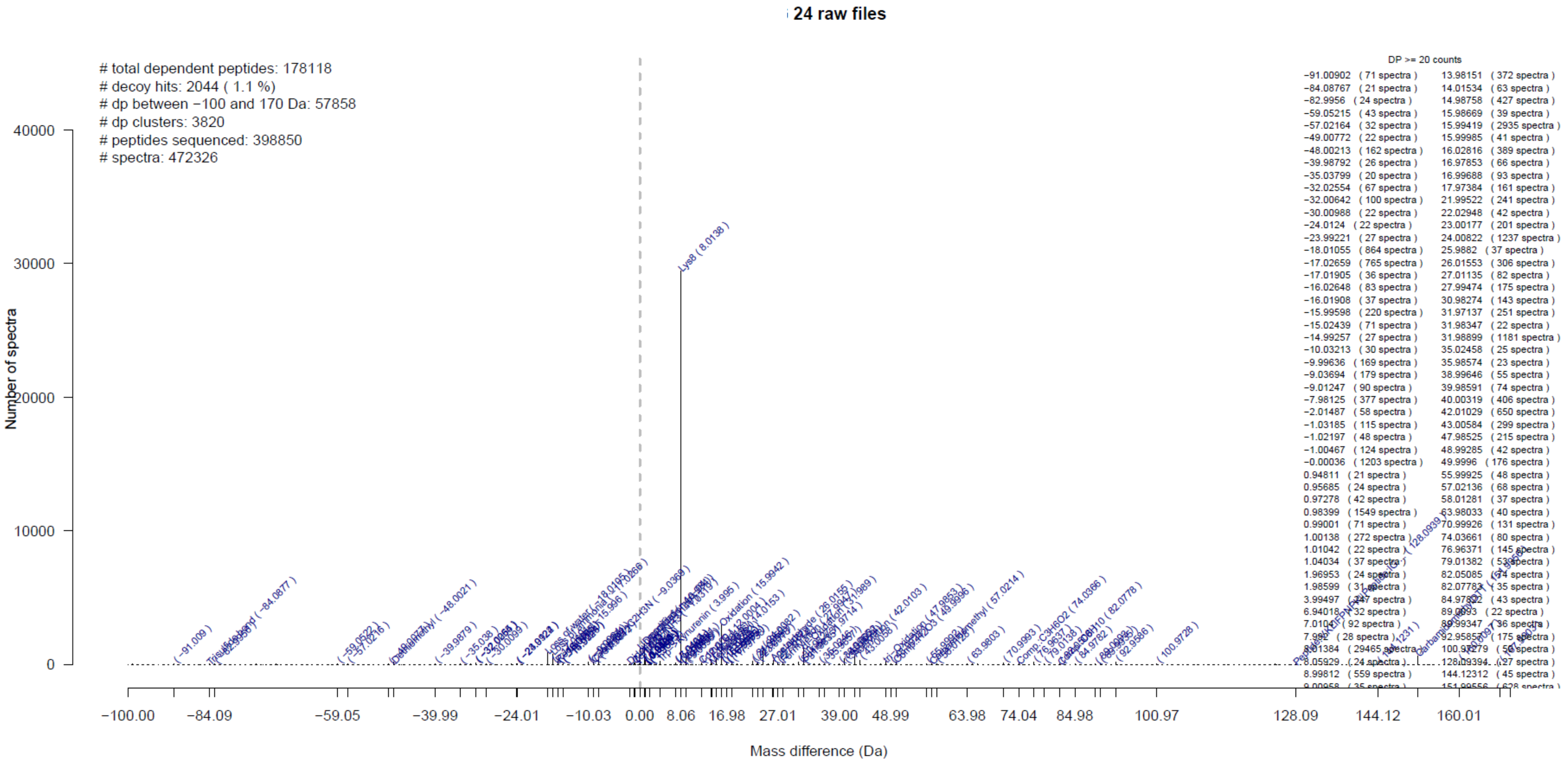
24 raw files
total dependent peptides: 178118
decoy hits: 2044 (1.1 %)
dp between −100 and 170 Da: 57858
dp clusters: 3820
peptides sequenced: 398850
spectra: 472326
Number of spectra
40000
30000
20000
10000
0
Lys8 (8.0138)
Mass difference (Da)
−100.00
−84.09
−59.05
−39.99
−24.01
−10.03
0.00
8.06
16.98
27.01
39.00
48.99
63.98
74.04
84.98
100.97
128.09
144.12
160.01
DP >= 20 counts
−91.00902 (71 spectra)
−84.08767 (21 spectra)
−82.9956 (24 spectra)
−59.05215 (43 spectra)
−57.02164 (32 spectra)
−49.00772 (22 spectra)
−48.00213 (162 spectra)
−39.98792 (26 spectra)
−35.03799 (20 spectra)
−32.02554 (67 spectra)
−32.00642 (100 spectra)
−30.00988 (22 spectra)
−24.0124 (22 spectra)
−23.99221 (27 spectra)
−18.01055 (864 spectra)
−17.02659 (765 spectra)
−17.01905 (36 spectra)
−16.02648 (83 spectra)
−16.01908 (37 spectra)
−15.99598 (220 spectra)
−15.02439 (71 spectra)
−14.99257 (27 spectra)
−10.03213 (30 spectra)
−9.99636 (169 spectra)
−9.03694 (179 spectra)
−9.01247 (90 spectra)
−7.98125 (377 spectra)
−2.01487 (58 spectra)
−1.03185 (115 spectra)
−1.02197 (48 spectra)
−1.00467 (124 spectra)
−0.00036 (1203 spectra)
0.94811 (21 spectra)
0.95685 (24 spectra)
0.97278 (42 spectra)
0.98399 (1549 spectra)
0.99001 (71 spectra)
1.00138 (272 spectra)
1.01042 (22 spectra)
1.04034 (37 spectra)
1.96953 (24 spectra)
1.98599 (31 spectra)
3.99497 (247 spectra)
6.94018 (32 spectra)
7.0104 (92 spectra)
7.992 (28 spectra)
8.01384 (29465 spectra)
8.05929 (24 spectra)
8.99812 (559 spectra)
13.98151 (372 spectra)
14.01534 (63 spectra)
14.98758 (427 spectra)
15.98669 (39 spectra)
15.99419 (2935 spectra)
15.99985 (41 spectra)
16.02816 (389 spectra)
16.97853 (66 spectra)
16.99688 (93 spectra)
17.97384 (161 spectra)
21.99522 (241 spectra)
22.02948 (42 spectra)
23.00177 (201 spectra)
24.00822 (1237 spectra)
25.9882 (37 spectra)
26.01553 (306 spectra)
27.01135 (82 spectra)
27.99474 (175 spectra)
30.98274 (143 spectra)
31.97137 (251 spectra)
31.98347 (22 spectra)
31.98899 (1181 spectra)
35.02458 (25 spectra)
35.98574 (23 spectra)
38.99646 (55 spectra)
39.98591 (74 spectra)
40.00319 (406 spectra)
42.01029 (650 spectra)
43.00584 (299 spectra)
47.98525 (215 spectra)
48.99285 (42 spectra)
49.9996 (176 spectra)
55.99925 (48 spectra)
57.02136 (68 spectra)
58.01281 (37 spectra)
63.98033 (40 spectra)
70.99926 (131 spectra)
74.03661 (80 spectra)
76.96371 (145 spectra)
82.07783 (35 spectra)
89.0093 (22 spectra)
92.95857 (175 spectra)
128.09394 (27 spectra)
144.12312 (45 spectra)

TP4

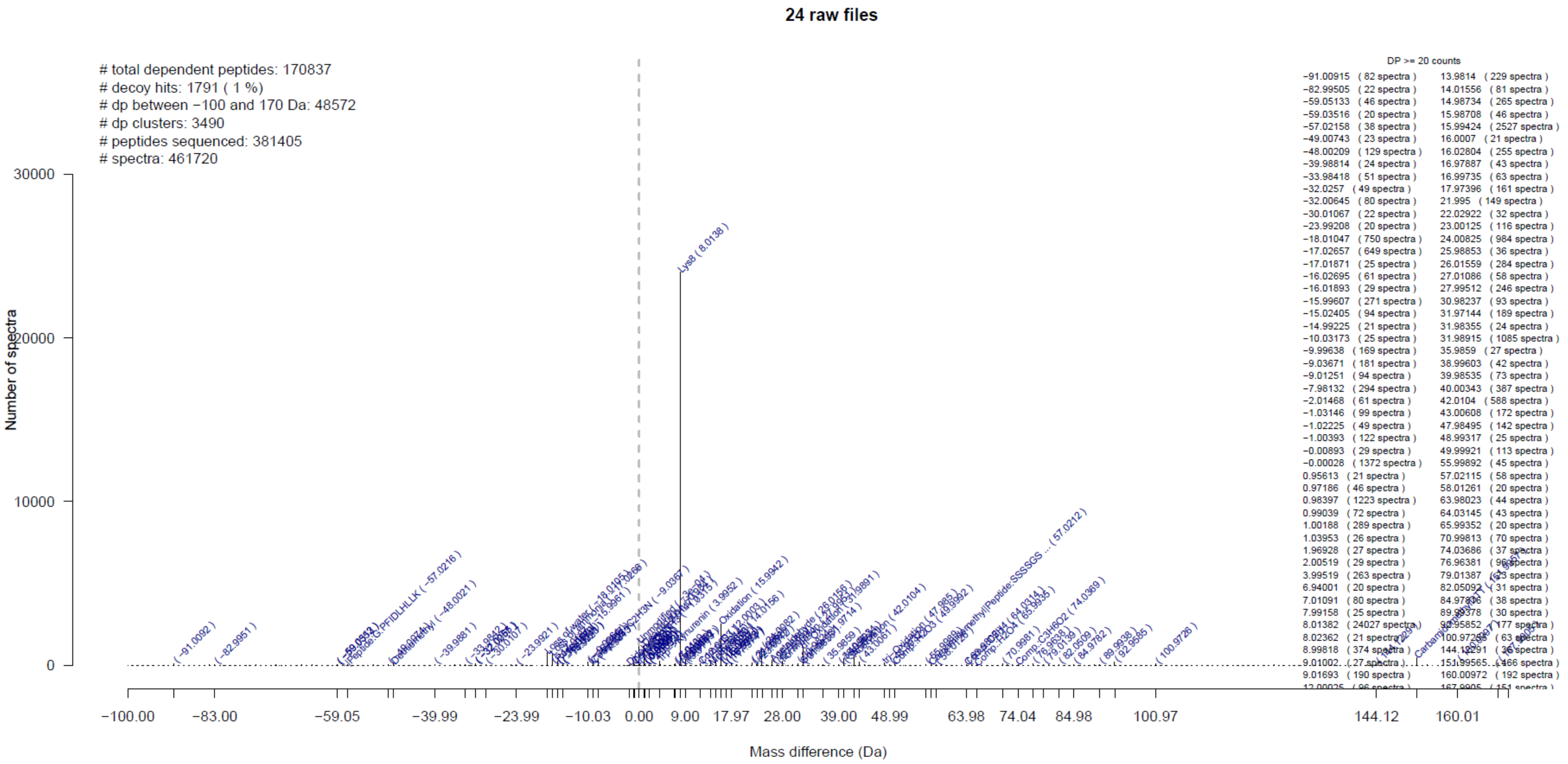
24 raw files
total dependent peptides: 170837
decoy hits: 1791 (1 %)
dp between −100 and 170 Da: 48572
dp clusters: 3490
peptides sequenced: 381405
spectra: 461720
Number of spectra
30000
20000
10000
0
Mass difference (Da)
−100.00
−83.00
−59.05
−39.99
−23.99
−10.03
0.00
9.00
17.97
28.00
39.00
48.99
63.98
74.04
84.98
100.97
144.12
160.01
Lys8 (8.0138)
(−91.0092)
(−82.9951)
Peptide:G.PFIDLHLLK (−57.0216)
Dethiomethyl (−48.0021)
(−39.9881)
(−23.9921)
Unmodified
Oxidation (15.9942)
Acetyl (42.0104)
Comp.:H2O4 (64.0314)
Comp.:C3H6O2 (74.0369)
(70.9981)
(82.0509)
(84.9782)
(89.9938)
(92.9585)
(100.9726)
DP >= 20 counts
−91.00915 (82 spectra)
−82.99505 (22 spectra)
−59.05133 (46 spectra)
−59.03516 (20 spectra)
−57.02158 (38 spectra)
−49.00743 (23 spectra)
−48.00209 (129 spectra)
−39.98814 (24 spectra)
−33.98418 (51 spectra)
−32.0257 (49 spectra)
−32.00645 (80 spectra)
−30.01067 (22 spectra)
−23.99208 (20 spectra)
−18.01047 (750 spectra)
−17.02657 (649 spectra)
−17.01871 (25 spectra)
−16.02695 (61 spectra)
−16.01893 (29 spectra)
−15.99607 (271 spectra)
−15.02405 (94 spectra)
−14.99225 (21 spectra)
−10.03173 (25 spectra)
−9.99638 (169 spectra)
−9.03671 (181 spectra)
−9.01251 (94 spectra)
−7.98132 (294 spectra)
−2.01468 (61 spectra)
−1.03146 (99 spectra)
−1.02225 (49 spectra)
−1.00393 (122 spectra)
−0.00893 (29 spectra)
−0.00028 (1372 spectra)
0.95613 (21 spectra)
0.97186 (46 spectra)
0.98397 (1223 spectra)
0.99039 (72 spectra)
1.00188 (289 spectra)
1.03953 (26 spectra)
1.96928 (27 spectra)
2.00519 (29 spectra)
3.99519 (263 spectra)
6.94001 (20 spectra)
7.01091 (80 spectra)
7.99158 (25 spectra)
8.01382 (24027 spectra)
8.02362 (21 spectra)
8.99818 (374 spectra)
9.01002 (27 spectra)
9.01693 (190 spectra)
12.00025 (96 spectra)
13.9814 (229 spectra)
14.01556 (81 spectra)
14.98734 (265 spectra)
15.98708 (46 spectra)
15.99424 (2527 spectra)
16.0007 (21 spectra)
16.02804 (255 spectra)
16.97887 (43 spectra)
16.99735 (63 spectra)
17.97396 (161 spectra)
21.995 (149 spectra)
22.02922 (32 spectra)
23.00125 (116 spectra)
24.00825 (984 spectra)
25.98853 (36 spectra)
26.01559 (284 spectra)
27.01086 (58 spectra)
27.99512 (246 spectra)
30.98237 (93 spectra)
31.97144 (189 spectra)
31.98355 (24 spectra)
31.98915 (1085 spectra)
35.9859 (27 spectra)
38.99603 (42 spectra)
39.98535 (73 spectra)
40.00343 (387 spectra)
42.0104 (588 spectra)
43.00608 (172 spectra)
47.98495 (142 spectra)
48.99317 (25 spectra)
49.99921 (113 spectra)
55.99892 (45 spectra)
57.02115 (58 spectra)
58.01261 (20 spectra)
63.98023 (44 spectra)
64.03145 (43 spectra)
65.99352 (20 spectra)
70.99813 (70 spectra)
74.03686 (37 spectra)
76.96381 (96 spectra)
79.01387 (23 spectra)
82.05092 (31 spectra)
89.99378 (30 spectra)
151.99565 (466 spectra)
160.00972 (192 spectra)
167.9905 (151 spectra)

TP5

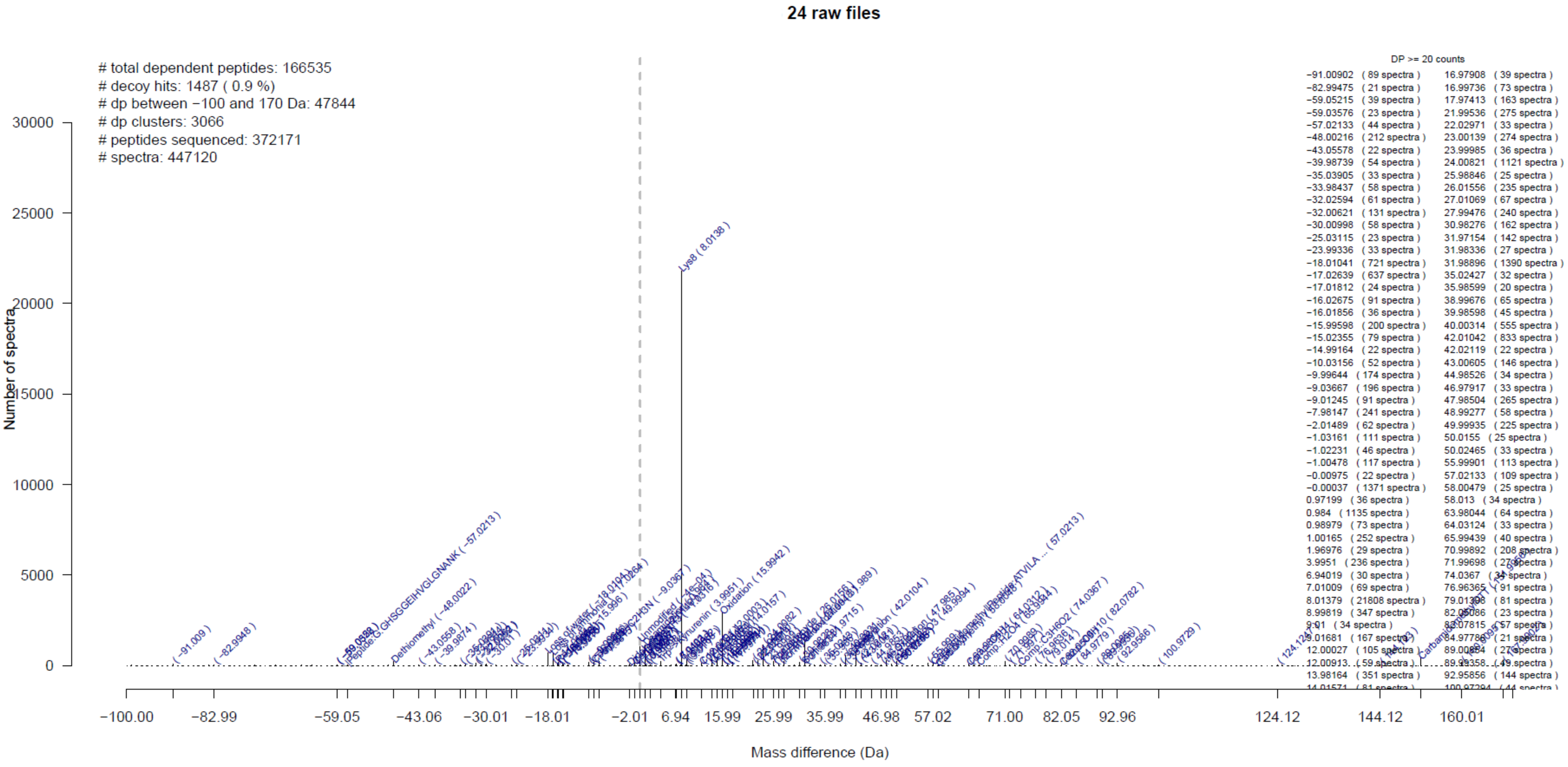
24 raw files
total dependent peptides: 166535
decoy hits: 1487 (0.9 %)
dp between −100 and 170 Da: 47844
dp clusters: 3066
peptides sequenced: 372171
spectra: 447120
Number of spectra
0
5000
10000
15000
20000
25000
30000
Lys8 (8.0138)
Oxidation (15.9942)
Dethiomethyl (−48.0022)
−100.00
−82.99
−59.05
−43.06
−30.01
−18.01
−2.01
6.94
15.99
25.99
35.99
46.98
57.02
71.00
82.05
92.96
124.12
144.12
160.01
Mass difference (Da)
DP >= 20 counts
−91.00902 (89 spectra) 16.97908 (39 spectra)
−82.99475 (21 spectra) 16.99736 (73 spectra)
−59.05215 (39 spectra) 17.97413 (163 spectra)
−59.03576 (23 spectra) 21.99536 (275 spectra)
−57.02133 (44 spectra) 22.02971 (33 spectra)
−48.00216 (212 spectra) 23.00139 (274 spectra)
−43.05578 (22 spectra) 23.99985 (36 spectra)
−39.98739 (54 spectra) 24.00821 (1121 spectra)
−35.03905 (33 spectra) 25.98846 (25 spectra)
−33.98437 (58 spectra) 26.01556 (235 spectra)
−32.02594 (61 spectra) 27.01069 (67 spectra)
−32.00621 (131 spectra) 27.99476 (240 spectra)
−30.00998 (58 spectra) 30.98276 (162 spectra)
−25.03115 (23 spectra) 31.97154 (142 spectra)
−23.99336 (33 spectra) 31.98336 (27 spectra)
−18.01041 (721 spectra) 31.98896 (1390 spectra)
−17.02639 (637 spectra) 35.02427 (32 spectra)
−17.01812 (24 spectra) 35.98599 (20 spectra)
−16.02675 (91 spectra) 38.99676 (65 spectra)
−16.01856 (36 spectra) 39.98598 (45 spectra)
−15.99598 (200 spectra) 40.00314 (555 spectra)
−15.02355 (79 spectra) 42.01042 (833 spectra)
−14.99164 (22 spectra) 42.02119 (22 spectra)
−10.03156 (52 spectra) 43.00605 (146 spectra)
−9.99644 (174 spectra) 44.98526 (34 spectra)
−9.03667 (196 spectra) 46.97917 (33 spectra)
−9.01245 (91 spectra) 47.98504 (265 spectra)
−7.98147 (241 spectra) 48.99277 (58 spectra)
−2.01489 (62 spectra) 49.99935 (225 spectra)
−1.03161 (111 spectra) 50.0155 (25 spectra)
−1.02231 (46 spectra) 50.02465 (33 spectra)
−1.00478 (117 spectra) 55.99901 (113 spectra)
−0.00975 (22 spectra) 57.02133 (109 spectra)
−0.00037 (1371 spectra) 58.00479 (25 spectra)
0.97199 (36 spectra) 58.013 (34 spectra)
0.984 (1135 spectra) 63.98044 (64 spectra)
0.98979 (73 spectra) 64.03124 (33 spectra)
1.00165 (252 spectra) 65.99439 (40 spectra)
1.96976 (29 spectra) 70.99892 (208 spectra)
3.9951 (236 spectra) 71.99698 (27 spectra)
6.94019 (30 spectra) 74.0367 (34 spectra)
7.01009 (69 spectra) 76.96365 (91 spectra)
8.01379 (21808 spectra) 79.01398 (81 spectra)
8.99819 (347 spectra) 82.05086 (23 spectra)
9.01 (34 spectra) 83.07815 (57 spectra)
9.01681 (167 spectra) 84.97786 (21 spectra)
12.00027 (105 spectra) 89.00854 (27 spectra)
12.00913 (59 spectra) 89.93358 (49 spectra)
13.98164 (351 spectra) 92.95856 (144 spectra)
14.01571 (81 spectra) 100.97294 (44 spectra)

TP6

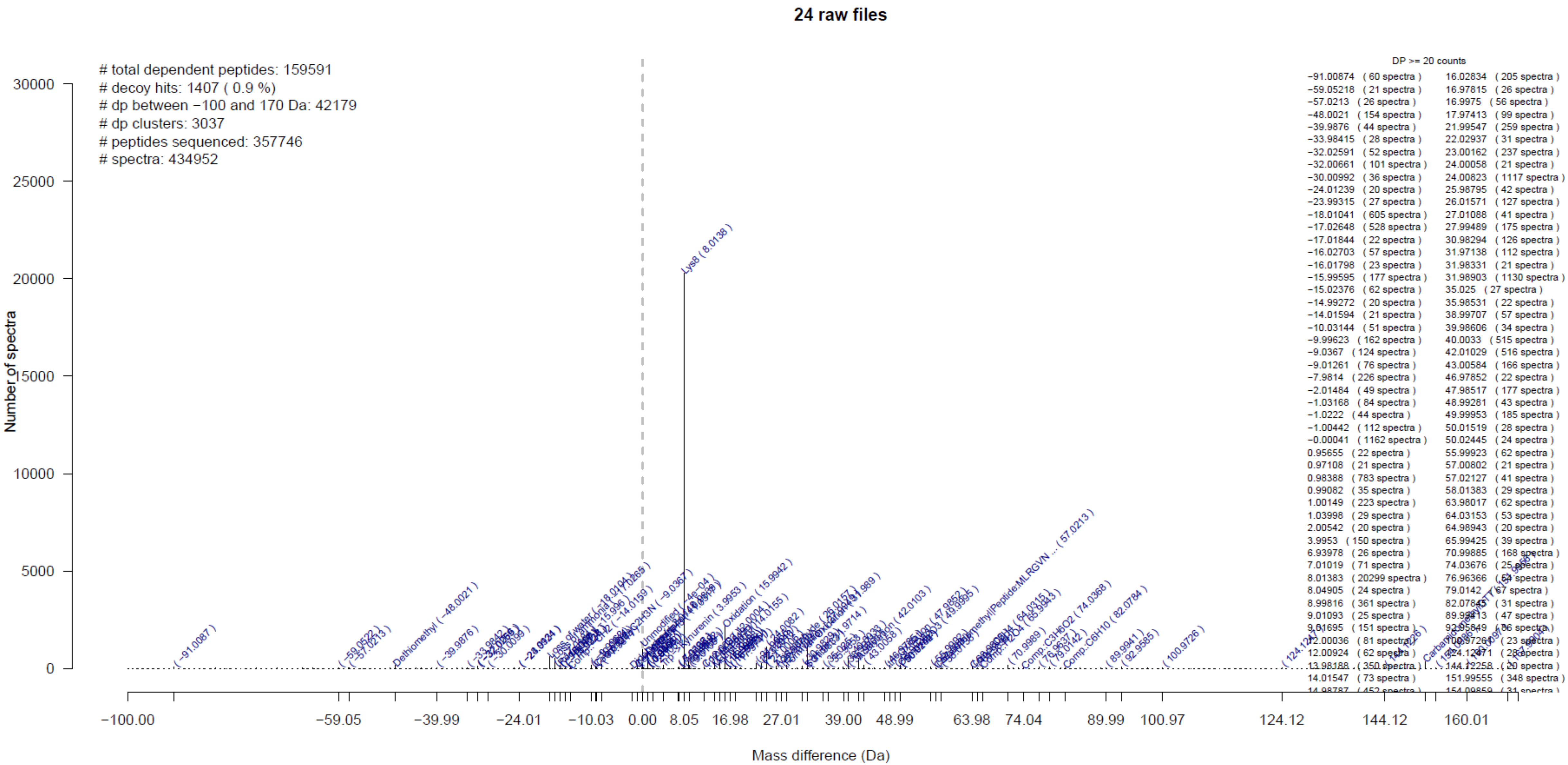
24 raw files
total dependent peptides: 159591
decoy hits: 1407 (0.9 %)
dp between −100 and 170 Da: 42179
dp clusters: 3037
peptides sequenced: 357746
spectra: 434952
Number of spectra
Mass difference (Da)
Lys8 (8.0138)
Dethiomethyl (−48.0021)
DP >= 20 counts
−91.00874 (60 spectra) 16.02834 (205 spectra)
−59.05218 (21 spectra) 16.97815 (26 spectra)
−57.0213 (26 spectra) 16.9975 (56 spectra)
−48.0021 (154 spectra) 17.97413 (99 spectra)
−39.9876 (44 spectra) 21.99547 (259 spectra)
−33.98415 (28 spectra) 22.02937 (31 spectra)
−32.02591 (52 spectra) 23.00162 (237 spectra)
−32.00661 (101 spectra) 24.00058 (21 spectra)
−30.00992 (36 spectra) 24.00823 (1117 spectra)
−24.01239 (20 spectra) 25.98795 (42 spectra)
−23.99315 (27 spectra) 26.01571 (127 spectra)
−18.01041 (605 spectra) 27.01088 (41 spectra)
−17.02648 (528 spectra) 27.99489 (175 spectra)
−17.01844 (22 spectra) 30.98294 (126 spectra)
−16.02703 (57 spectra) 31.97138 (112 spectra)
−16.01798 (23 spectra) 31.98331 (21 spectra)
−15.99595 (177 spectra) 31.98903 (1130 spectra)
−15.02376 (62 spectra) 35.025 (27 spectra)
−14.99272 (20 spectra) 35.98531 (22 spectra)
−14.01594 (21 spectra) 38.99707 (57 spectra)
−10.03144 (51 spectra) 39.98606 (34 spectra)
−9.99623 (162 spectra) 40.0033 (515 spectra)
−9.0367 (124 spectra) 42.01029 (516 spectra)
−9.01261 (76 spectra) 43.00584 (166 spectra)
−7.9814 (226 spectra) 46.97852 (22 spectra)
−2.01484 (49 spectra) 47.98517 (177 spectra)
−1.03168 (84 spectra) 48.99281 (43 spectra)
−1.0222 (44 spectra) 49.99953 (185 spectra)
−1.00442 (112 spectra) 50.01519 (28 spectra)
−0.00041 (1162 spectra) 50.02445 (24 spectra)
0.95655 (22 spectra) 55.99923 (62 spectra)
0.97108 (21 spectra) 57.00802 (21 spectra)
0.98388 (783 spectra) 57.02127 (41 spectra)
0.99082 (35 spectra) 58.01383 (29 spectra)
1.00149 (223 spectra) 63.98017 (62 spectra)
1.03998 (29 spectra) 64.03153 (53 spectra)
2.00542 (20 spectra) 64.98943 (20 spectra)
3.9953 (150 spectra) 65.99425 (39 spectra)
6.93978 (26 spectra) 70.99885 (168 spectra)
7.01019 (71 spectra) 74.03676 (25 spectra)
8.01383 (20299 spectra) 76.96366 (54 spectra)
8.04905 (24 spectra) 79.0142 (67 spectra)
8.99816 (361 spectra) 82.07845 (31 spectra)
9.01093 (25 spectra) 89.99413 (47 spectra)
9.01695 (151 spectra) 92.95849 (86 spectra)
12.00036 (81 spectra) 100.97261 (23 spectra)
12.00924 (62 spectra) 124.12471 (28 spectra)
13.98188 (350 spectra) 144.12258 (20 spectra)
14.01547 (73 spectra) 151.99555 (348 spectra)

TP7

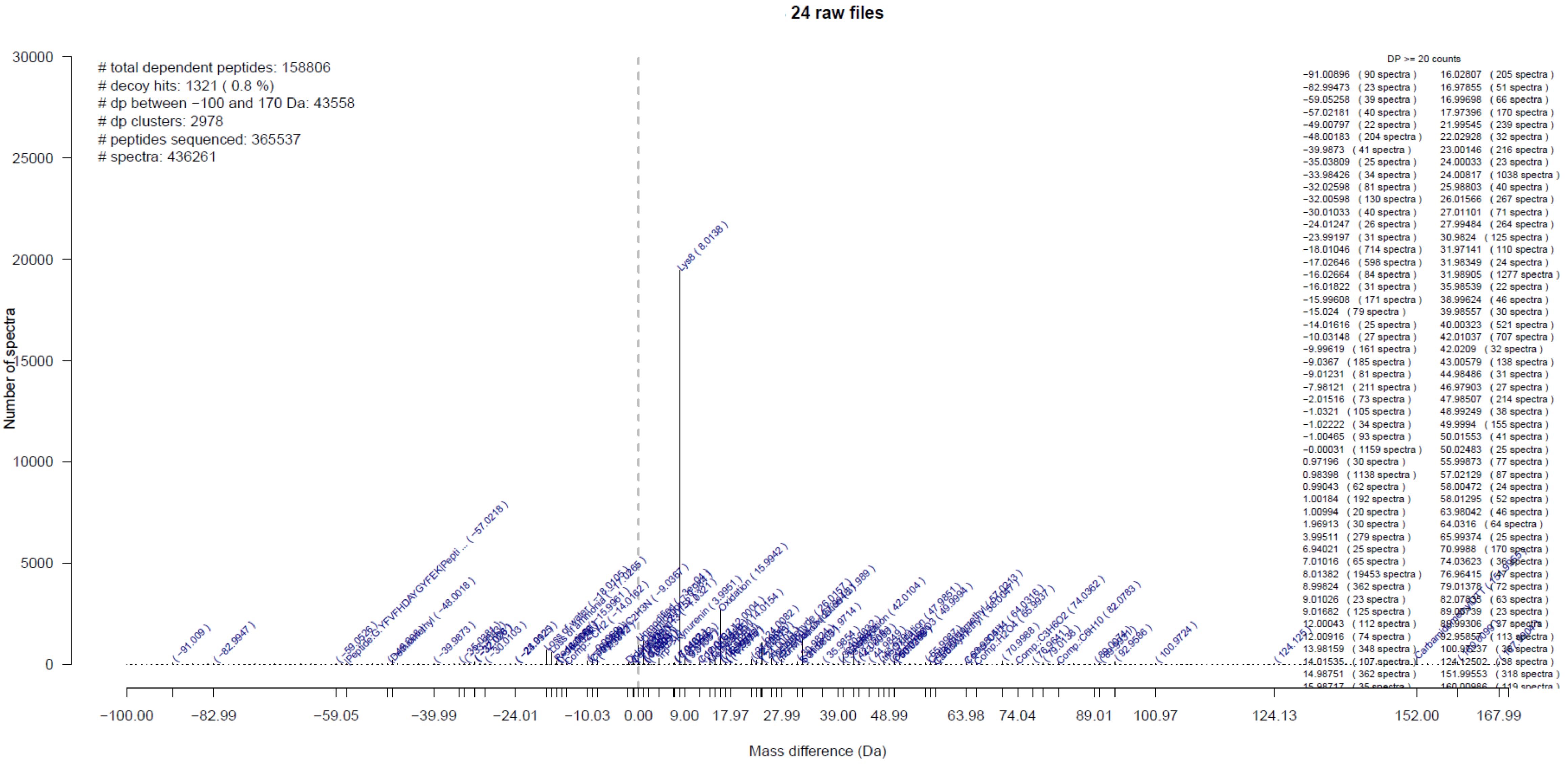

24 raw files
total dependent peptides: 158806
decoy hits: 1321 (0.8 %)
dp between −100 and 170 Da: 43558
dp clusters: 2978
peptides sequenced: 365537
spectra: 436261
Number of spectra
Mass difference (Da)
Lys8 (8.0138)
Oxidation (15.9942)
DP >= 20 counts
−91.00896 (90 spectra) 16.02807 (205 spectra)
−82.99473 (23 spectra) 16.97855 (51 spectra)
−59.05258 (39 spectra) 16.99698 (66 spectra)
−57.02181 (40 spectra) 17.97396 (170 spectra)
−49.00797 (22 spectra) 21.99545 (239 spectra)
−48.00183 (204 spectra) 22.02928 (32 spectra)
−39.9873 (41 spectra) 23.00146 (216 spectra)
−35.03809 (25 spectra) 24.00033 (23 spectra)
−33.98426 (34 spectra) 24.00817 (1038 spectra)
−32.02598 (81 spectra) 25.98803 (40 spectra)
−32.00598 (130 spectra) 26.01566 (267 spectra)
−30.01033 (40 spectra) 27.01101 (71 spectra)
−24.01247 (26 spectra) 27.99484 (264 spectra)
−23.99197 (31 spectra) 30.9824 (125 spectra)
−18.01046 (714 spectra) 31.97141 (110 spectra)
−17.02646 (598 spectra) 31.98349 (24 spectra)
−16.02664 (84 spectra) 31.98905 (1277 spectra)
−16.01822 (31 spectra) 35.98539 (22 spectra)
−15.99608 (171 spectra) 38.99624 (46 spectra)
−15.024 (79 spectra) 39.98557 (30 spectra)
−14.01616 (25 spectra) 40.00323 (521 spectra)
−10.03148 (27 spectra) 42.01037 (707 spectra)
−9.99619 (161 spectra) 42.0209 (32 spectra)
−9.0367 (185 spectra) 43.00579 (138 spectra)
−9.01231 (81 spectra) 44.98486 (31 spectra)
−7.98121 (211 spectra) 46.97903 (27 spectra)
−2.01516 (73 spectra) 47.98507 (214 spectra)
−1.0321 (105 spectra) 48.99249 (38 spectra)
−1.02222 (34 spectra) 49.9994 (155 spectra)
−1.00465 (93 spectra) 50.01553 (41 spectra)
−0.00031 (1159 spectra) 50.02483 (25 spectra)
0.97196 (30 spectra) 55.99873 (77 spectra)
0.98398 (1138 spectra) 57.02129 (87 spectra)
0.99043 (62 spectra) 58.00472 (24 spectra)
1.00184 (192 spectra) 58.01295 (52 spectra)
1.00994 (20 spectra) 63.98042 (46 spectra)
1.96913 (30 spectra) 64.0316 (64 spectra)
3.99511 (279 spectra) 65.99374 (25 spectra)
6.94021 (25 spectra) 70.9988 (170 spectra)
7.01016 (65 spectra) 74.03623 (36 spectra)
8.01382 (19453 spectra) 76.96415 (47 spectra)
8.99824 (362 spectra) 79.01378 (72 spectra)
9.01026 (23 spectra) 82.07835 (63 spectra)
9.01682 (125 spectra) 89.00739 (23 spectra)
12.00043 (112 spectra) 89.99306 (37 spectra)
12.00916 (74 spectra) 92.95857 (113 spectra)
13.98159 (348 spectra) 100.97237 (36 spectra)
14.01535 (107 spectra) 124.12502 (38 spectra)
14.98751 (362 spectra) 151.99553 (318 spectra)
