# Supplementary material for: Characterization of the E. coli proteome and its modifications during growth and ethanol stress
Source: Front Microbiol. 2015 Feb 18;6:103. doi: 10.3389/fmicb.2015.00103 (PMC4332353; doi:10.3389/fmicb.2015.00103)
Supplement: Supplementary file 1 [file DataSheet1.ZIP › Supplementary File 3- DP pairwise comparison ethanol stress.pdf]

Pairwise Comparison of Dependent peptide analysis during ethanol stress

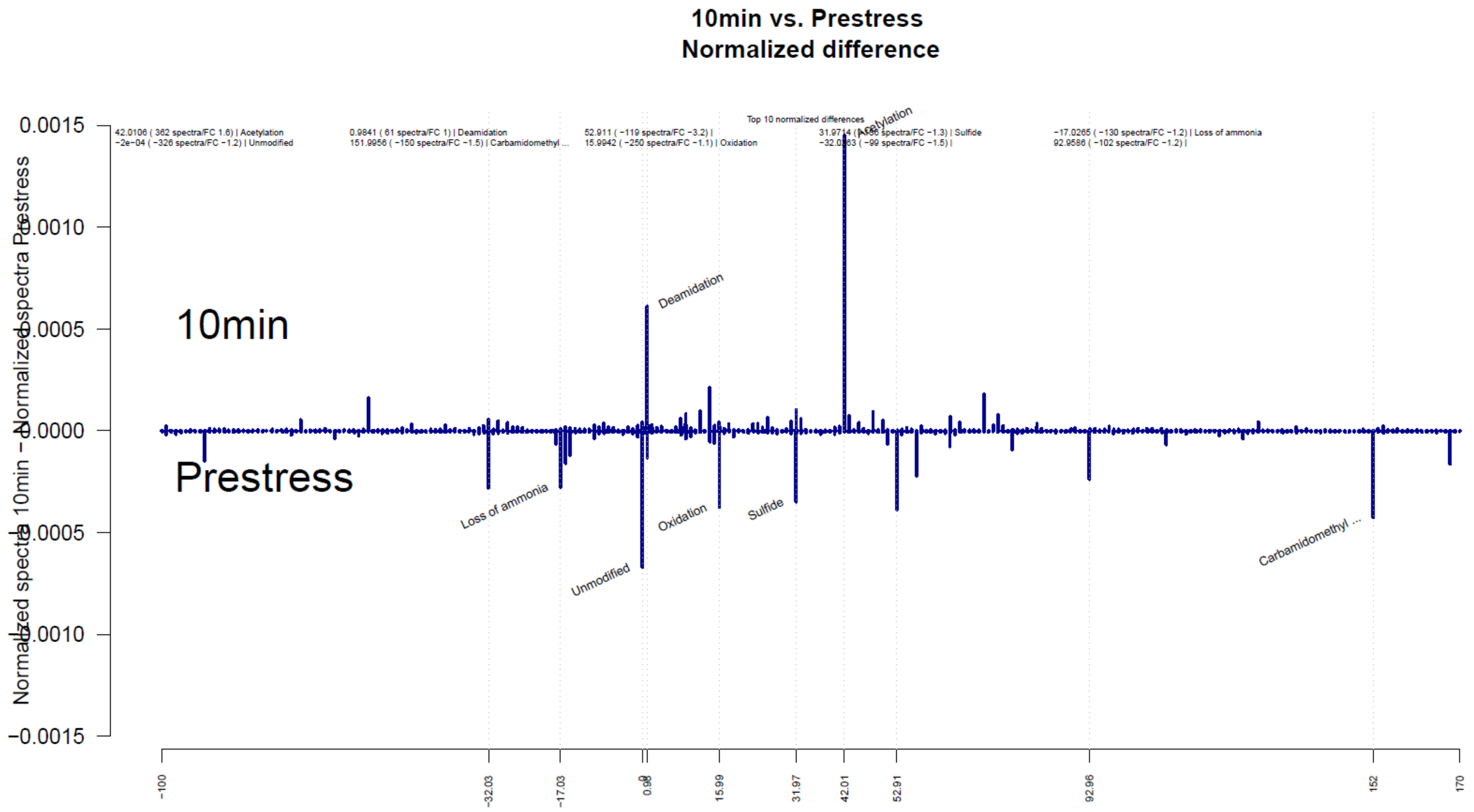

10min vs. Prestress
Normalized difference
Top 10 normalized differences
42.0106 (362 spectra/FC 1.6) | Acetylation
−2e−04 (−326 spectra/FC −1.2) | Unmodified
0.9841 (61 spectra/FC 1) | Deamidation
151.9956 (−150 spectra/FC −1.5) | Carbamidomethyl ...
52.911 (−119 spectra/FC −3.2) |
15.9942 (−250 spectra/FC −1.1) | Oxidation
31.9714 spectra/FC −1.3) | Sulfide
−32.0263 (−99 spectra/FC −1.5) |
−17.0265 (−130 spectra/FC −1.2) | Loss of ammonia
92.9586 (−102 spectra/FC −1.2) |
Normalized spectra 10min − Normalized spectra Prestress
10min
Prestress
Acetylation
Deamidation
Loss of ammonia
Unmodified
Oxidation
Sulfide
Carbamidomethyl ...
0.0015
0.0010
0.0005
0.0000
−0.0005
−0.0010
−0.0015
−100
−32.03
−17.03
0
0.98
15.99
31.97
42.01
52.91
92.96
152
170

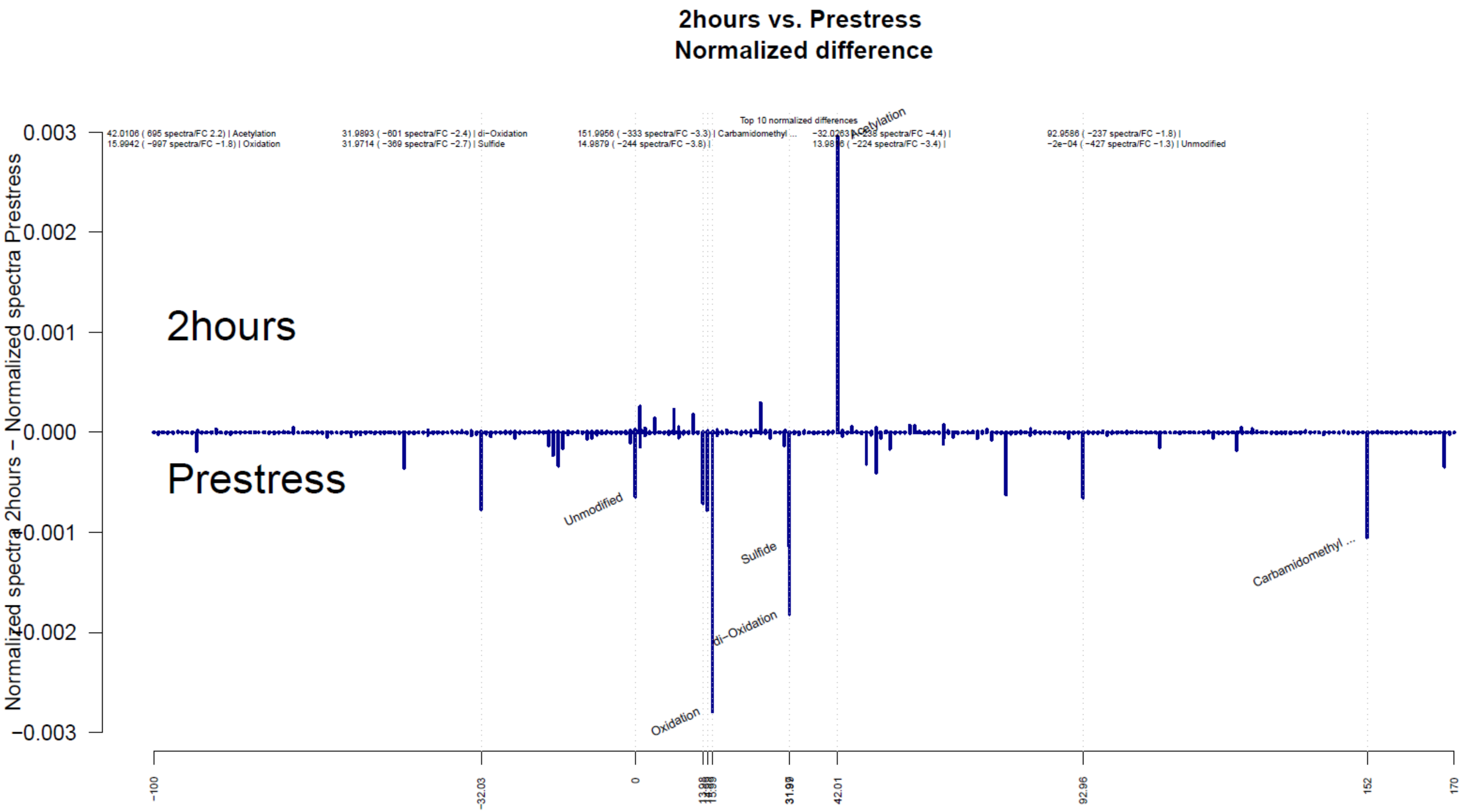
2hours vs. Prestress
Normalized difference
Top 10 normalized differences
42.0106 (695 spectra/FC 2.2) | Acetylation
15.9942 (−997 spectra/FC −1.8) | Oxidation
31.9893 (−601 spectra/FC −2.4) | di−Oxidation
31.9714 (−369 spectra/FC −2.7) | Sulfide
151.9956 (−333 spectra/FC −3.3) | Carbamidomethyl ...
14.9879 (−244 spectra/FC −3.8) |
−32.0263
spectra/FC −4.4) |
(−224 spectra/FC −3.4) |
92.9586 (−237 spectra/FC −1.8) |
−2e−04 (−427 spectra/FC −1.3) | Unmodified
Normalized spectra 2hours − Normalized spectra Prestress
0.003
0.002
0.001
0.000
−0.001
−0.002
−0.003
2hours
Prestress
Acetylation
Unmodified
Sulfide
di−Oxidation
Oxidation
Carbamidomethyl ...
−100
−32.03
0
42.01
92.96
152
170

Dependent peptide (DP) analysis during ethanol stress

Quality Control : Dataset (SILAC) processed as unlabeled with DP function on

Prestress 24 raw files

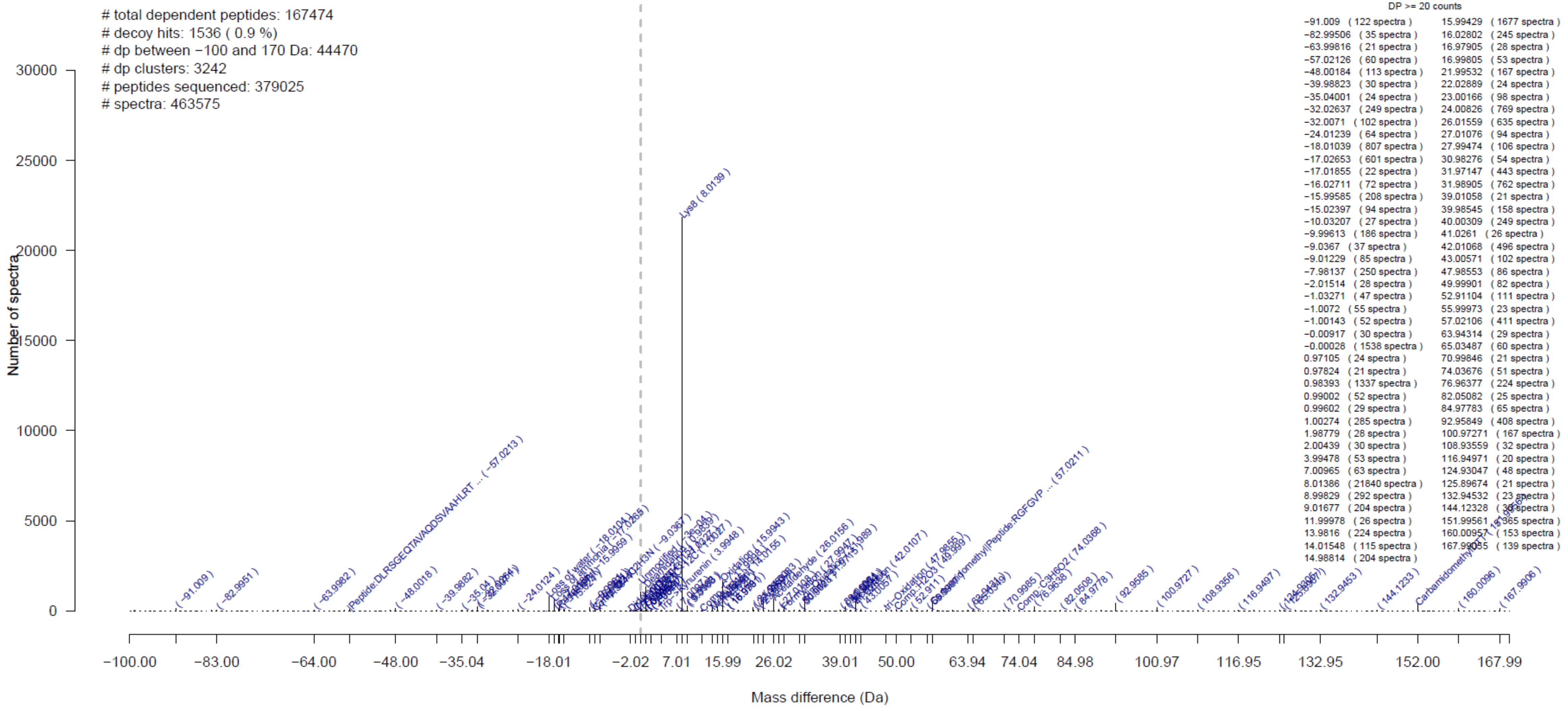

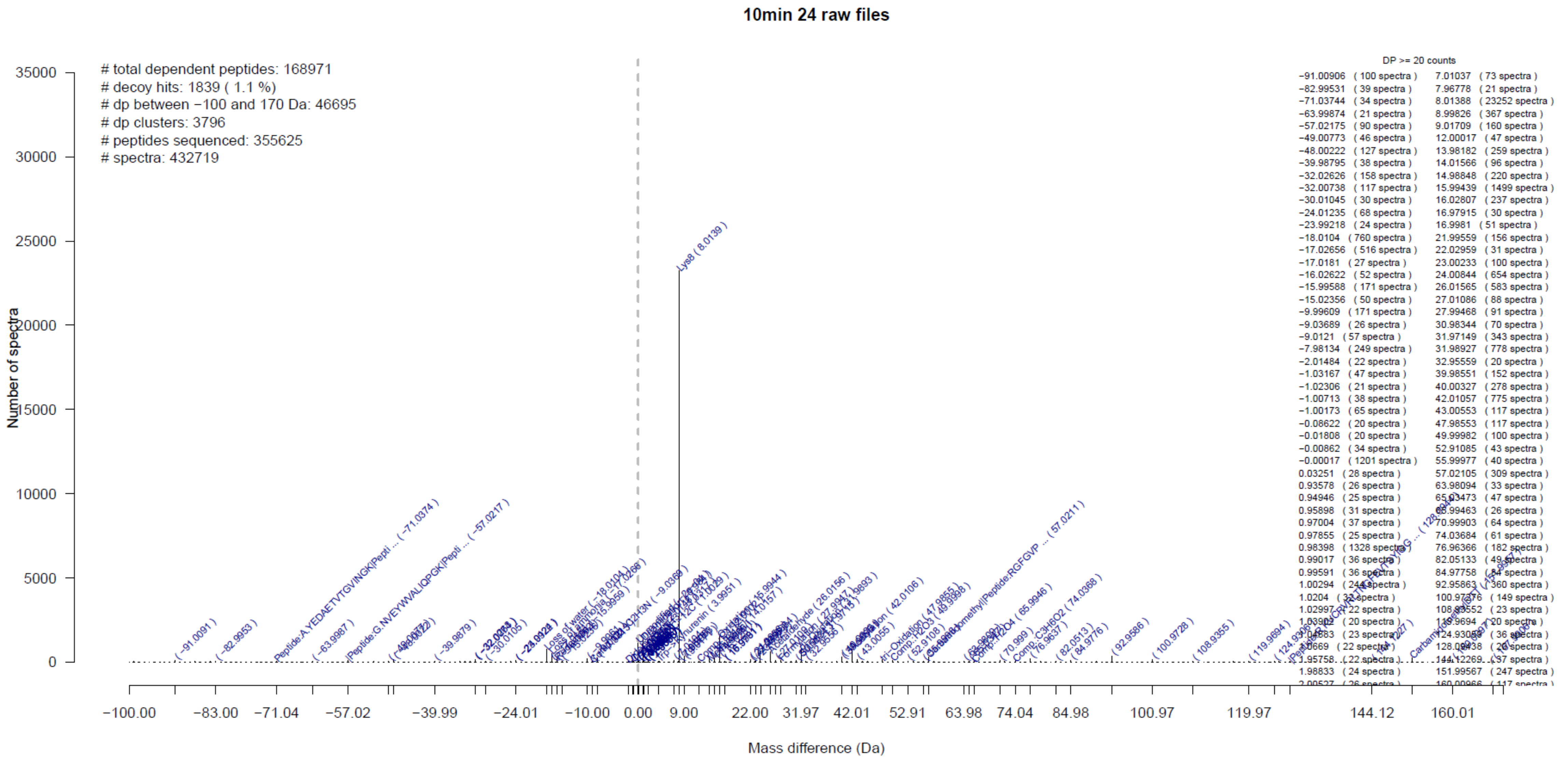
10min 24 raw files
total dependent peptides: 168971
decoy hits: 1839 (1.1 %)
dp between −100 and 170 Da: 46695
dp clusters: 3796
peptides sequenced: 355625
spectra: 432719
Number of spectra
35000
30000
25000
20000
15000
10000
5000
0
Mass difference (Da)
−100.00
−83.00
−71.04
−57.02
−39.99
−24.01
−10.00
0.00
9.00
22.00
31.97
42.01
52.91
63.98
74.04
84.98
100.97
119.97
144.12
160.01
Lys8 (8.0139)
Peptide:A.YEDAETVTGVINGK|Pepti ... (−71.0374)
Peptide:G.NVEYWVALIQPGK|Pepti ... (−57.0217)
DP >= 20 counts
−91.00906 (100 spectra) 7.01037 (73 spectra)
−82.99531 (39 spectra) 7.96778 (21 spectra)
−71.03744 (34 spectra) 8.01388 (23252 spectra)
−63.99874 (21 spectra) 8.99826 (367 spectra)
−57.02175 (90 spectra) 9.01709 (160 spectra)
−49.00773 (46 spectra) 12.00017 (47 spectra)
−48.00222 (127 spectra) 13.98182 (259 spectra)
−39.98795 (38 spectra) 14.01566 (96 spectra)
−32.02626 (158 spectra) 14.98848 (220 spectra)
−32.00738 (117 spectra) 15.99439 (1499 spectra)
−30.01045 (30 spectra) 16.02807 (237 spectra)
−24.01235 (68 spectra) 16.97915 (30 spectra)
−23.99218 (24 spectra) 16.9981 (51 spectra)
−18.0104 (760 spectra) 21.99559 (156 spectra)
−17.02656 (516 spectra) 22.02959 (31 spectra)
−17.0181 (27 spectra) 23.00233 (100 spectra)
−16.02622 (52 spectra) 24.00844 (654 spectra)
−15.99588 (171 spectra) 26.01565 (583 spectra)
−15.02356 (50 spectra) 27.01086 (88 spectra)
−9.99609 (171 spectra) 27.99468 (91 spectra)
−9.03689 (26 spectra) 30.98344 (70 spectra)
−9.0121 (57 spectra) 31.97149 (343 spectra)
−7.98134 (249 spectra) 31.98927 (778 spectra)
−2.01484 (22 spectra) 32.95559 (20 spectra)
−1.03167 (47 spectra) 39.98551 (152 spectra)
−1.02306 (21 spectra) 40.00327 (278 spectra)
−1.00713 (38 spectra) 42.01057 (775 spectra)
−1.00173 (65 spectra) 43.00553 (117 spectra)
−0.08622 (20 spectra) 47.98553 (117 spectra)
−0.01808 (20 spectra) 49.99982 (100 spectra)
−0.00862 (34 spectra) 52.91085 (43 spectra)
−0.00017 (1201 spectra) 55.99977 (40 spectra)
0.03251 (28 spectra) 57.02105 (309 spectra)
0.93578 (26 spectra) 63.98094 (33 spectra)
0.94946 (25 spectra) 65.93473 (47 spectra)
0.95898 (31 spectra) 65.99463 (26 spectra)
0.97004 (37 spectra) 70.99903 (64 spectra)
0.97855 (25 spectra) 74.03684 (61 spectra)
0.98398 (1328 spectra) 76.96366 (182 spectra)
0.99017 (36 spectra) 82.05133 (49 spectra)
0.99591 (36 spectra) 84.97758 (84 spectra)
1.00294 (244 spectra) 92.95863 (360 spectra)
1.0204 (32 spectra) 100.97376 (149 spectra)
1.02997 (22 spectra) 108.93552 (23 spectra)
1.03902 (20 spectra) 119.9694 (20 spectra)
1.04883 (23 spectra) 124.93059 (36 spectra)
1.0669 (22 spectra) 128.09438 (20 spectra)
1.95758 (22 spectra) 144.12269 (37 spectra)
1.98833 (24 spectra) 151.99567 (247 spectra)
2.00527 (26 spectra) 160.00966 (117 spectra)

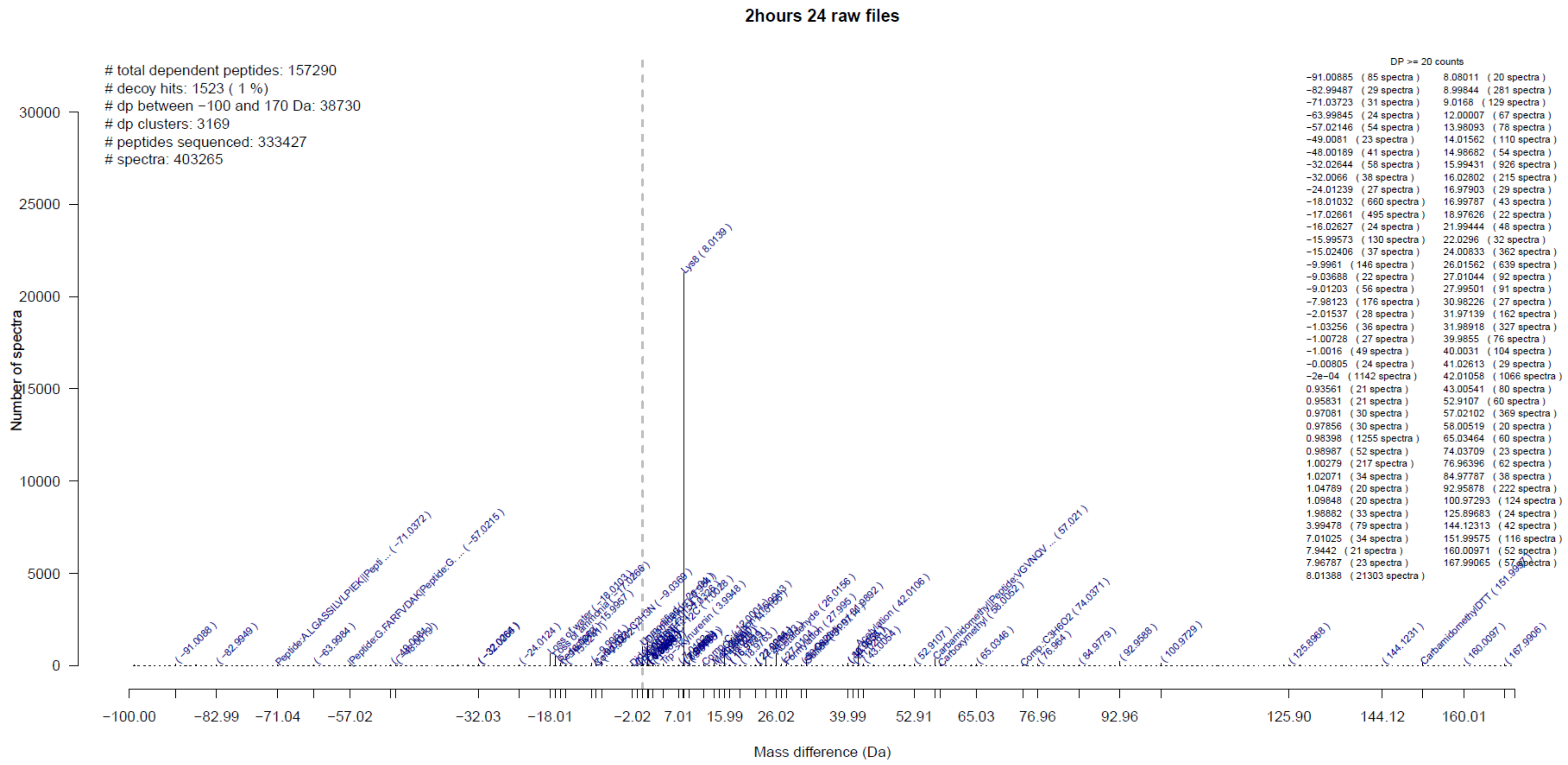
2hours 24 raw files
total dependent peptides: 157290
decoy hits: 1523 (1 %)
dp between −100 and 170 Da: 38730
dp clusters: 3169
peptides sequenced: 333427
spectra: 403265
DP >= 20 counts
−91.00885 (85 spectra)
−82.99487 (29 spectra)
−71.03723 (31 spectra)
−63.99845 (24 spectra)
−57.02146 (54 spectra)
−49.0081 (23 spectra)
−48.00189 (41 spectra)
−32.02644 (58 spectra)
−32.0066 (38 spectra)
−24.01239 (27 spectra)
−18.01032 (660 spectra)
−17.02661 (495 spectra)
−16.02627 (24 spectra)
−15.99573 (130 spectra)
−15.02406 (37 spectra)
−9.9961 (146 spectra)
−9.03688 (22 spectra)
−9.01203 (56 spectra)
−7.98123 (176 spectra)
−2.01537 (28 spectra)
−1.03256 (36 spectra)
−1.00728 (27 spectra)
−1.0016 (49 spectra)
−0.00805 (24 spectra)
−2e−04 (1142 spectra)
0.93561 (21 spectra)
0.95831 (21 spectra)
0.97081 (30 spectra)
0.97856 (30 spectra)
0.98398 (1255 spectra)
0.98987 (52 spectra)
1.00279 (217 spectra)
1.02071 (34 spectra)
1.04789 (20 spectra)
1.09848 (20 spectra)
1.98882 (33 spectra)
3.99478 (79 spectra)
7.01025 (34 spectra)
7.9442 (21 spectra)
7.96787 (23 spectra)
8.01388 (21303 spectra)
8.08011 (20 spectra)
8.99844 (281 spectra)
9.0168 (129 spectra)
12.00007 (67 spectra)
13.98093 (78 spectra)
14.01562 (110 spectra)
14.98682 (54 spectra)
15.99431 (926 spectra)
16.02802 (215 spectra)
16.97903 (29 spectra)
16.99787 (43 spectra)
18.97626 (22 spectra)
21.99444 (48 spectra)
22.0296 (32 spectra)
24.00833 (362 spectra)
26.01562 (639 spectra)
27.01044 (92 spectra)
27.99501 (91 spectra)
30.98226 (27 spectra)
31.97139 (162 spectra)
31.98918 (327 spectra)
39.9855 (76 spectra)
40.0031 (104 spectra)
41.02613 (29 spectra)
42.01058 (1066 spectra)
43.00541 (80 spectra)
52.9107 (60 spectra)
57.02102 (369 spectra)
58.00519 (20 spectra)
65.03464 (60 spectra)
74.03709 (23 spectra)
76.96396 (62 spectra)
84.97787 (38 spectra)
92.95878 (222 spectra)
100.97293 (124 spectra)
125.89683 (24 spectra)
144.12313 (42 spectra)
151.99575 (116 spectra)
160.00971 (52 spectra)
167.99065 (57 spectra)
Lys8 (8.0139)
Number of spectra
30000
25000
20000
15000
10000
5000
0
−100.00
−82.99
−71.04
−57.02
−32.03
−18.01
−2.02
7.01
15.99
26.02
39.99
52.91
65.03
76.96
92.96
125.90
144.12
160.01
Mass difference (Da)
