# Supplementary material for: Characterization of the E. coli proteome and its modifications during growth and ethanol stress
Source: Front Microbiol. 2015 Feb 18;6:103. doi: 10.3389/fmicb.2015.00103 (PMC4332353; doi:10.3389/fmicb.2015.00103)
Supplement: Supplementary file 1 [file DataSheet1.ZIP › Supplementary text.pdf]

Supplementary text

Protein Identification

We employed the super-SILAC approach in order to assess the relative and absolute dynamics of the proteome during seven different growth stages in *E.coli* grown in minimal medium: TP1: pre-exponential growth, TP2: transition into exponential growth, TP3: exponential growth; TP4: transition to stationary phase; TP5: early stationary phase; TP6: late stationary phase; TP7: extended late stationary phase (4 days after inoculation) (**Supplementary Fig.9A**). We extended the Super-SILAC methodology towards *E.coli* response to ethanol stress at three distinct timepoints: T1) pre-stress, T2) ten minutes post stress, T3) two hours post stress (**Supplementary Fig.9B**). Specific information with regards to growth and harvesting can be found in the Experimental Procedures section. Two biological replicates were performed for both growth and ethanol stress experiments. All biological samples had a high level of SILAC amino acid incorporation (**Supplementary Tab.S3**). In each biological replicate, orthogonal methods of peptide/protein separation (OffGel isoelectric focusing and GeLC-MS) and peptide fragmentation were employed (CID and HCD fragmentation). All peptide samples were analyzed on a nano-LC-MS system: LTQ Orbitrap Elite mass spectrometer. For Super-SILAC growth experiments, a total of 168 data files were acquired. For Super-SILAC ethanol stress experiments, a total of 72 data files were acquired. All proteins identified in both datasets as well as the relative and absolute quantitation and GO enrichment analysis can be seen in supplementary tables **S1, S2, S6 and S7.**

Biological Reproducibility

One critical aspect of the robustness and validity of data acquired in this experiment is the biological reproducibility of replicates. To this end, we performed Pearson correlation between experimental replicates in both growth and ethanol stress Super-SILAC experiments **(Supplementary Fig.10A, B).** In the Super-SILAC growth experiments, the Pearson correlation coefficients ranged from 0.70-0.83. In the Super-SILAC ethanol stress experiments, the Pearson correlation coefficients ranged from 0.73-0.76. Overall, the biological replicates quantified using the Super-SILAC method of quantitation produced very good reproducibility in all phases of growth, and ethanol stress time points. Protein profiles of GAPDH, MalE, and OmpX obtained from this study correlated well with recently published MS/Western blot data from a previous study (Soares et al., 2013) (**Supplementary Fig.11**). Since good reproducibility was obtained between all biological replicates, the replicate that contained the iBAQ standard required for absolute quantitation was chosen for further analysis with regards to absolute quantitation. This corresponds to biological replicate 2 (BR2) for both growth which identified and quantified more proteins and represents a more comprehensive dataset between the two biological replicates.

Further Insights into ethanol stress dataset

Recently, an ATP-independent chaperone protein known as Spy was found to play an important role in decreasing protein aggregation while simultaneously assisting in protein folding (Quan et al., 2011). Interestingly, we observed an induction of this protein during ethanol stress in both biological replicates with protein copy numbers estimated at approximately 80-500 copies per cell extending from pre-stress to 2 hours post ethanol stress. This observation could suggest that upon ethanol stress, the *E.coli* cells employ the use of periplasmic chaperones to dispose of aggregated proteins resulting from exposure to ethanol that would otherwise create imbalances to the homeostasis of the bacterial cell. Moreover, further evidence of periplasmic chaperone activity during ethanol stress was observed. For example, CpxP which has a 29% sequence homology with Spy (Quan et al., 2011) was found to be induced upon ethanol stress in biological

replicate 1. Unfortunately protein copy numbers could not be estimated for CpxP. Both CpxP and Spy were previously shown to be highly up regulated during butanol stress (Rutherford et al., 2010) suggesting that these targets play a role in general alcohol stress response.

E.coli employs mechanosensitive channels which are active during osmotic stress conditions (Edwards et al., 2012). These channels open rapidly when exposed to hypoosmotic shock which results in a large amount of water entering the bacterial cell inducing increased tension levels on the membrane (Edwards et al., 2012).There are two major groups of mechanosensitive proteins in *E.coli:* 1. MscL (Sukharev et al., 1994) and 2. MscS which has 6 homologs: MscS, MscK, YbdG, YbiO, YnaI, and YjeP (Levina et al., 1999). Thus far, mechanosensitive channels have only been described during osmotic stress, and in some mechanosensitive proteins such as YbiO reported to be specifically induced by NaCl only (Edwards et al., 2012). Interestingly our dataset does show an induction of certain mechanosensitive proteins during ethanol stress. Certain evidences both in this dataset (lack of increase in unsaturated fatty acids) and previously reported, suggests that membrane fluidity in *E.coli* increases upon ethanol stress. However during osmotic stress, these mechanosensitive channels are known to become activated and opened immediately after encountering an increase in membrane tension. Based on these reports, one would expect that these Msc proteins should not change since an increase in membrane fluidity as opposed to membrane tension occurs. This for the most part is true with MscS, MscK, MscL, Yjep, and YbdG showing overall minor expression level changes upon exposure to ethanol stress. However, YbiO appears to be present (induced) only after the addition of ethanol (both biological replicates) and YnaI is induced after being exposed to ethanol for 10 minutes (biological replicate 2). This raises interesting questions as to whether or not these specific mechanosensitive channel proteins have other functions other than responding to membrane tension caused from osmotic stress, and could suggest that they are important in circumventing stresses caused from alcohols and possible other types of solvents. Further experimental validation on the potential roles and functions of these proteins upon ethanol stress is required in order to draw any final conclusions.

Supplemental text references

Edwards, M.D., Black, S., Rasmussen, T., Rasmussen, A., Stokes, N.R., Stephen, T.L., Miller, S., and Booth, I.R. (2012). Characterization of three novel mechanosensitive channel activities in Escherichia coli. *Channels (Austin)* 6**,** 272-281. doi: 10.4161/chan.20998.

Levina, N., Totemeyer, S., Stokes, N.R., Louis, P., Jones, M.A., and Booth, I.R. (1999). Protection of Escherichia coli cells against extreme turgor by activation of MscS and MscL mechanosensitive channels: identification of genes required for MscS activity. *EMBO J* 18**,** 1730-1737. doi: 10.1093/emboj/18.7.1730.

Quan, S., Koldewey, P., Tapley, T., Kirsch, N., Ruane, K.M., Pfizenmaier, J., Shi, R., Hofmann, S., Foit, L., Ren, G., Jakob, U., Xu, Z., Cygler, M., and Bardwell, J.C. (2011). Genetic selection designed to stabilize proteins uncovers a chaperone called Spy. *Nat Struct Mol Biol* 18**,** 262-269. doi: 10.1038/nsmb.2016.

Rutherford, B.J., Dahl, R.H., Price, R.E., Szmidt, H.L., Benke, P.I., Mukhopadhyay, A., and Keasling, J.D. (2010). Functional genomic study of exogenous n-butanol stress in Escherichia coli. *Appl Environ Microbiol* 76**,** 1935-1945. doi: 10.1128/AEM.02323-09.

Soares, N.C., Spat, P., Krug, K., and Macek, B. (2013). Global dynamics of the Escherichia coli proteome and phosphoproteome during growth in minimal medium. *J Proteome Res* 12**,** 2611-2621. doi: 10.1021/pr3011843.

Sukharev, S.I., Blount, P., Martinac, B., Blattner, F.R., and Kung, C. (1994). A large-conductance mechanosensitive channel in E. coli encoded by mscL alone. *Nature* 368**,** 265-268. doi: 10.1038/368265a0.
